# Loss contexts enhance dorsolateral prefrontal interpersonal neural synchrony during successful human deceptive recommendations

**DOI:** 10.1038/s42003-026-10577-2

**Published:** 2026-06-30

**Authors:** Yaozhong Liu, Rui Huang, Xiaowei Gao, Jingao Zhang, Yuhao Jia, Jingyao Zheng, He Wang, Yingjie Liu

**Affiliations:** 1 Guangdong Province Key Laboratory of Philosophy, Social Sciences-Huashang Key Laboratory of Neuroeconomics, Guangdong, China; 2 School of Psychology and Mental Health, North China University of Science and Technology, Hebei, China; 3 Hebei Key Laboratory of Mental Health and Brain Science, Hebei, China

**Keywords:** Psychology, Human behaviour

## Abstract

Deception is an important social behavior because it can shape another person’s choices while concealing the sender’s true interests. Although people often respond differently when trying to gain rewards or avoid losses, it remains unclear how these contexts influence the shared brain processes that support deception during real interaction. Here we show that loss contexts make deception more frequent and more successful in human face-to-face communication. We study same-gender stranger dyads (18 female dyads) while sender gives recommendations that can either help or mislead the other under gain and loss contexts. Using functional near-infrared spectroscopy to record brain activity from both people at the same time, we find that misleading recommendations in gain contexts are associated with stronger activity in the dorsolateral prefrontal cortex, a frontal brain region involved in control and planning. In contrast, successful deception in loss contexts is associated with higher interpersonal brain synchrony in this region, defined as a greater increase in similarity between the two people’s brain activity during interaction compared with rest. This synchrony predicts deception success, suggesting that deception depends on context-sensitive alignment between interacting brains.

## Abstract (web summary)

fNIRS hyperscanning shows that loss contexts enhance interpersonal neural synchrony in the dorsolateral prefrontal cortex during successful deception, revealing how shared neural dynamics support strategic social interaction.

## Introduction

In real-world social interactions, information is often conveyed under conflicting interests or uncertain outcomes, making strategic communication a powerful means of shaping others’ judgments^1,2^. In such contexts, deception can be understood as a goal-directed regulatory behavior, whereby senders intentionally mislead receivers through verbal or non-verbal cues to obtain gains or avoid losses^3,4^. Because deceptive behavior depends not only on information veracity but also on how information is framed within a decision context^5^, identifying how contextual frames influence deception and its underlying neural dynamics is essential for advancing mechanistic models of social influence.

Framing effects offer a key account of how identical outcomes can guide behavior differently when presented as gains or losses^6,7^. Rooted in loss aversion and asymmetric value encoding, this framework posits that identical outcomes can elicit distinct psychological utilities when framed as gains versus losses^8^. According to Prospect Theory, potential losses carry greater psychological weight than equivalent gains, prompting individuals to recalibrate the value of available behavioral options^9^. This asymmetry is particularly relevant to deception: behavioral evidence suggests that potential losses increase dishonest^10,11^ or unethical actions^12^, primarily through loss avoidance rather than the pursuit of additional gains. Thus, loss contexts may make deception a more acceptable strategic option. We therefore hypothesized that individuals would deceive more frequently in loss contexts than in gain contexts.

Deceptive behavior involves not only evaluating outcome value^13^ but also dynamically integrating the detector’s anticipated responses^6^. Although behavioral studies can reveal contextual differences in deception frequency, they provide limited insight into how individuals evaluate and adjust deception as a strategy under different frames. Neurocognitive research therefore offers a complementary approach by identifying the computational and regulatory mechanisms underlying deceptive choices^14^. Existing evidence suggests that deception relies on distributed valuation and decision-making networks rather than a single brain region^15^. Within these networks, the prefrontal cortex (PFC) plays a central regulatory role by flexibly modulating value signals according to contextual demands^16,17^, particularly when social decisions require response inhibition^18,19^, strategic planning^20,21^, or behavioral adjustment^22,23^.

As a key node of the prefrontal cortex network, the dorsolateral prefrontal cortex (DLPFC) is commonly implicated in strategic planning^18^, response inhibition^21^, and contextual regulation^24^, including in studies of deceptive behavior^6,25^. However, DLPFC activity is not consistently observed across all decision-making tasks involving value evaluation, suggesting that its functional contribution is contingent on context-specific demands for cognitive control and strategic monitoring^26^. Gain-loss contexts provide a critical framework for understanding this context dependency. Whereas loss contexts are often dominated by urgency and threat-related processing^27^, gain contexts may require more fine-grained comparisons among competing options^28^. Thus, deception in gain contexts may place greater demands on strategic control, response inhibition, and value-based behavioral selection. We therefore hypothesized that deceptive behavior would elicit higher DLPFC activation in gain contexts than in loss contexts.

Interpersonal Deception Theory characterizes deception as a dynamic communicative process that requires continuous mutual adaptation between cheaters and detectors^29^. Such reciprocal adaptation necessarily engages multiple neurocognitive systems, including mentalizing, affective evaluation, and executive control. Consistent with this view, hyperscanning studies have identified interpersonal neural synchrony (INS) across a distributed set of regions during socially strategic interactions. For example, temporoparietal and superior temporal regions—implicated in mental state attribution—have shown increased inter-brain coupling during competitive and deceptive exchanges^25,30,31^, while anterior cingulate and orbitofrontal regions—associated with conflict monitoring and outcome evaluation—also exhibit context-sensitive synchrony during interactive decision-making^32^. These findings suggest that deception-related coordination reflects large-scale interpersonal network engagement rather than activity within a single cortical locus. Because deceptive interactions require both cheaters and detectors to monitor ongoing exchanges, anticipate each other’s responses, and update their strategies, control-related PFC regions are especially relevant at the dyadic level. In particular, DLPFC synchrony has been linked to behavioral coordination and shared strategic adjustment across joint action^33^, negotiation^34^, and deception paradigms^6,25^. Thus, increased DLPFC synchrony may reflect the alignment of control-related processes between interaction partners, rather than deception-specific activation per se.

Neuroimage research on gain-loss framing has consistently shown that loss contexts preferentially recruit limbic and salience-related circuitry, including the amygdala and anterior insula, and are associated with faster, more automatic response tendencies^35,36^. In contrast, gain contexts more reliably engage prefrontal regulatory regions, particularly the DLPFC supporting sustained goal maintenance, deliberative evaluation, and strategic control^37,38^. This neural dissociation suggests that framing context does not merely alter subjective valuation, but differentially constrains the degree of executive coordination required during decision-making. Extending this framework to interpersonal deception, we propose that gain contexts impose higher requirements for coordinated regulation during deceptive interaction. Because successful deception requires continuous monitoring, prediction, and adjustment, contexts that amplify prefrontal engagement at the individual level should likewise enhance interpersonal alignment within control-related networks. Accordingly, we proposed Hypothesize 3 that deceptive interactions in gain contexts will elicit higher INS increment (ΔINS) in the DLPFC, which will be positively associated with successful deception.

Conventional correlational^39^ or regression-based analyses^40^ can identify brain–behavior associations within specific contexts, but they provide limited evidence for whether these associations generalize across contexts or remain robust under alternative modeling assumptions. Addressing these questions requires analytical approaches that move beyond descriptive fitting toward prediction-oriented validation^41^. Machine-learning (ML) methods offer a suitable framework by treating neural measures as predictive features and behavioral outcomes as targets, allowing direct tests of whether brain-based signals contain reliable information about deception performance^34,42^. In the present study, we used a cross-validated, multi-model framework (refer to Huang et al.) to examine whether ΔINS predicted individual differences in cheaters’ successful deception rates under gain-loss contexts^6^. By comparing models with different inductive biases and evaluating performance [including Logistic Regression, least-squares regression (LSR), Decision Trees (DT), Naive Bayes, and Support Vector Regression (SVR)] using leave-one-subject-out cross-validation (LOSO-CV), this method assesses the generalizability and robustness of neural–behavioral associations beyond a single regression model.

The present study conceptualizes deception as a context-sensitive and interaction-dependent decision process. By integrating gain-loss framing with interpersonal neuroscience, we examined how contextual value structures shape deception through both intra-brain regulation and inter-brain alignment. Using functional near-infrared spectroscopy (fNIRS) hyperscanning, we focused on the interaction phase to test whether control-related prefrontal dynamics at the individual and dyadic levels were modulated by gain-loss context during receiver-disadvantageous recommendations, and whether these neural dynamics predicted successful deception. Finally, by combining hyperscanning with cross-validated machine-learning models, we assessed the robustness and generalizability of neural markers of deception beyond descriptive brain–behavior associations. Our work aims to inform the development of evidence-based counter-deception strategies.

## Results

### Manipulation check result

All participants endorsed the realism of the gain-loss context manipulation (9-point Likert scale, *M*_gain_ = 7.35, *SD* = 1.02; *M*_loss_ = 7.40, *SD* = 1.07), and no participants were excluded due to insufficient perceived realism.

### Subjective report results

Baseline analyses revealed no significant differences between male and female dyads in any pre-experimental demographic measures (education background, age, Monthly disposable income, Inclusion of Other in the Self Scale (IOS) score; *p*s > 0.063; Supplementary Table 1), indicating comparable baseline characteristics across dyads. To examine whether individual differences in detector-related personality traits could confound susceptibility to deception, we compared detectors’ scores on the Social Vulnerability Scale (SVS) and the Hyperarousal Scale (HAS) between gender dyads using independent-samples *t* tests. The results revealed no significant differences between groups on either deception-relevant personality dimension (*p*s > 0.234, Supplementary Table 1). These findings indicate that detectors in male and female dyads were comparable in baseline personality traits. Paired-samples *t* tests showed no significant pre–post changes in IOS scores under either gain or loss contexts (Pretest=1.04 ± 0.21; Post test: gain vs. loss: 1.09 ± 0.29 vs. 1.07 ± 0.26, *p*s > 0.321). These results indicate that the experimental manipulation did not systematically alter perceived interpersonal closeness between dyad members.

### Behavioral results: deception rate

This study examined how cheaters weighed potential outcomes when deciding whether to deceive or act honestly under conflicting interests. Dyads that rarely made objectively detector-disadvantageous recommendations were therefore excluded from data analyses, because such patterns provide insufficient behavioral variability for modeling deception-related processes. Specifically, two dyads with honesty rates exceeding 85% across the experiment were removed. This exclusion criterion was predefined before data analysis and applied uniformly across all conditions.

**Fig. 1:**
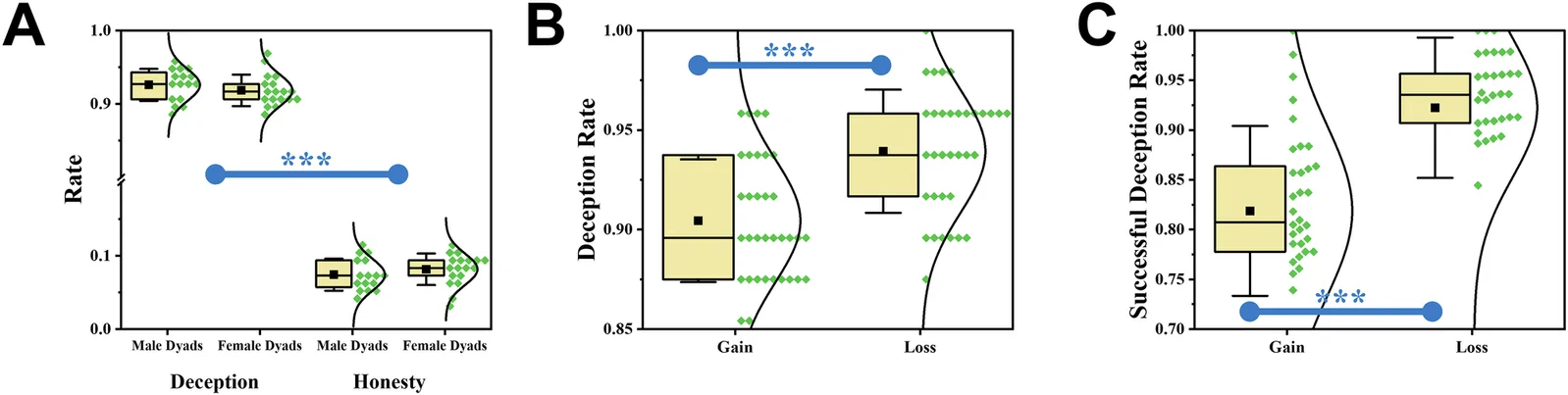
Behavioral results of deception (*n* = 34 healthy adults). **A** Proportion of behavioral occurrence (honesty vs. deception). **B** Deception rate across gain and loss contexts. **C** Successful deception rate across gain and loss contexts. Green diamonds represent individual data points; black squares denote condition means; lines within boxes indicate medians; error bars represent ±1 SD; outer contours depict normal distribution curves. *** *p* < 0.001. The same conventions apply in subsequent figures. *n* = 34 biologically independent samples.

To examine potential gender effects on cheater behavior, we conducted a 2 (Gender: male vs. female) ×2 (Behavior type: honesty vs. deception) mixed-design ANOVA. A significant main effect of behavior type was observed[*F*_(1,32)_ = 12878.747, *p* < 0.001, ${\eta }_{p}^{2}$ = 0.998, Fig. 1A], indicating that deception (0.92 ± 0.02) occurred substantially more frequently than honesty (0.08 ± 0.02). No significant main effects of gender or interaction effects were detected (*p*s > 0.329). These findings suggest that the experimental paradigm effectively elicited deception behavior without gender differences. Subsequent analyses focused exclusively on deceptive trials for two reasons: (1) the primary objective of this study was to investigate the behavioral and neural mechanisms underlying active deception interaction; (2) honesty trials were too sparse to support reliable statistical modeling (female: 8.16%; male: 7.42%). A paired-samples *t* test comparing deception rates across contexts revealed that cheaters engaged in deception more frequently under loss than gain contexts [loss vs. gain: 0.94 ± 0.03 vs. 0.90 ± 0.03, *t*_(33)_ =  470.626; *p* < 0.001, *Cohen’s d* = –0.793, Fig. 1B].

Beyond sender behavior, we examined detectors’ trial-level acceptance of the cheater’s recommendation. Detector acceptance was operationally defined as selecting the recommended option during the final decision phase (i.e., successful deception). A paired-samples *t* test showed that successful deception rates were significantly higher under loss than gain contexts [loss vs. gain: 0.92 ± 0.07 vs. 0.82 ± 0.09, *t*_(33)_ = –5.304; *p* < 0.001, *Cohen’s d* = –0.910, Fig. 1C], indicating that detectors were more likely to adopt the cheater’s recommendation in loss contexts.

### Behavioral results: reaction time (RT)

**Fig. 2:**
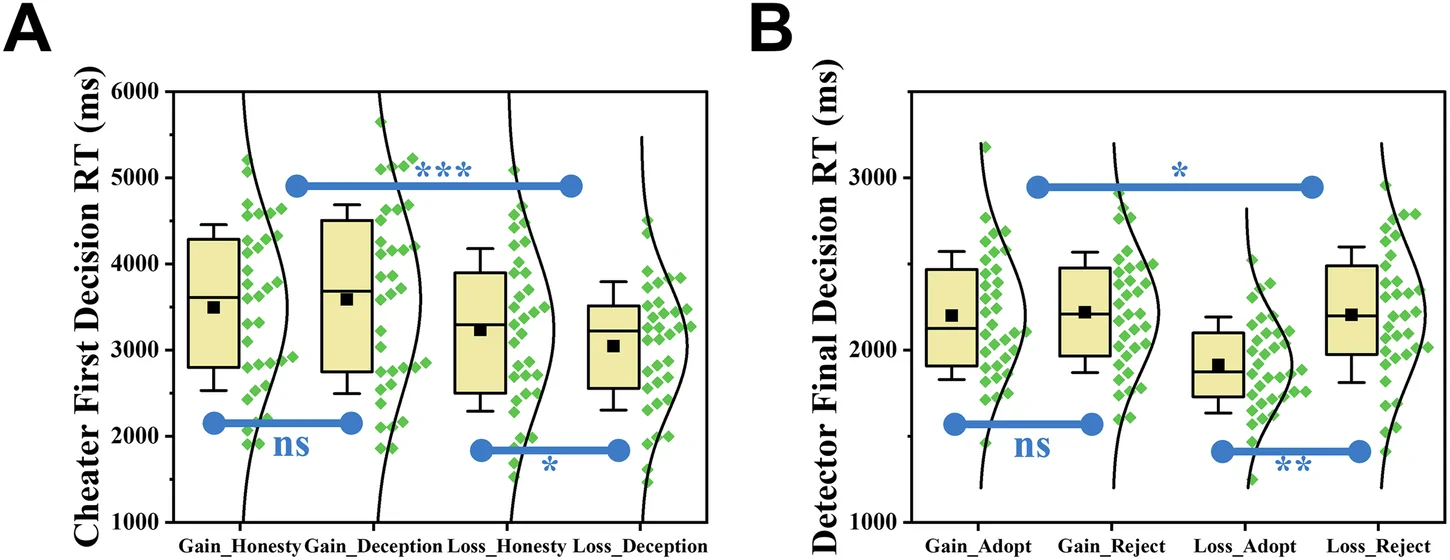
RT results (*n* = 34 healthy adults). **A** Cheater RT at the first decision phase. **B** Detector RT at the final decision phase. **p < 0.01, *p < 0.05, ns non-significant. The same conventions apply in subsequent figures.

To examine the RT underlying cheaters’ first decisions (i.e., the first behavioral expression of deception versus honesty), a 2 (Context: gain vs. loss) ×2 (Behavior type: honesty vs. deception) repeated-measures ANOVA was conducted. A significant main effect of contextual framing was observed [loss vs. gain: 3144.11 ± 139.16 vs. 3541.29 ± 174.11, *F*_(1,33)_ = 35.132, *p* < 0.001, ${\eta }_{p}^{2}$ = 0.516], indicating that RT were significantly shorter under loss compared to gain contexts. A significant interaction between context and behavior type also emerged [*F*_(1,33)_ = 5.0422, *p* = 0.032, ${\eta }_{p}^{2}$ = 0.133]. Simple-effects analyses revealed that under loss contexts, deception were executed significantly faster than honesty (deception vs. honesty: 3047.40 ± 127.74 vs. 3240.82 ± 159.51; post hoc *p* = 0.018, *95% CI* [ − 351.727, −35.111]). Contrastly, under gain contexts, no significant difference was observed between deceptive and honest responses (deception vs. honesty: 3589.68 ± 187.97 vs. 3492.91 ± 165.10; post hoc *p* = 0.132, *95% CI* [ − 30.630, 224.161]). No significant main effect of behavior type was detected [deception vs. honesty: 3318.54 ± 151.49 vs. 3366.87 ± 157.77, *F*_(1,33)_ = 2.876, *p* = 0.099, ${\eta }_{p}^{2}$ = 0.080, Fig. 2A].

We further examined detectors’ RT during the final decision phase to characterize their decision-making dynamics under different contextual and recommendation conditions. A 2 (context: gain vs. loss) ×2 (recommendation adoption: adopted vs. rejected) repeated-measures ANOVA revealed a significant main effect of context, with overall shorter RT in loss compared to gain contexts [loss vs. gain: 2058.96 ± 38.67 vs. 2209.68 ± 46.18, *F*_(1,33)_ = 5.125, *p* = 0.030, ${\eta }_{p}^{2}$ = 0.134]. A significant main effect of recommendation adoption was also observed, indicating that RT were shorter when detectors adopted the cheater’s recommendation (2056.82 ± 39.34) than when they rejected (2211.82 ± 43.87) it [*F*_(1,33)_ = 5.829, *p* = 0.021, ${\eta }_{p}^{2}$ = 0.150]. Importantly, the interaction between context and recommendation adoption was significant [*F*_(1,33)_ = 6.024, *p* = 0.020, ${\eta }_{p}^{2}$ = 0.154]. Simple-effects analyses showed that in loss contexts, RT were significantly shorter when recommendations were adopted compared to rejected (adopted vs. rejected: 1912.96 ± 47.80 vs. 2204.96 ± 67.52, post hoc *p* = 0.002, *95% CI* [113.406, 470.599]). No significant difference between adoption (2200.68 ± 63.69) and rejection (2218.68 ± 59.93) was observed in gain contexts (post hoc *p* = 0.828, *95% CI* [ − 149.387, 185.398]). These results are illustrated in Fig. 2B.

### Intra-brain activation results

To ensure the reliability of neural signals, a trial-level quality control procedure was implemented prior to Generalized Linear Model estimation. In accordance with the predefined data-cleaning criteria, trials with atypical speaking durations during the verbal statement were excluded. 79 trials were removed due to insufficient quality, including 26 trials in which the sender exceeded the allowed speaking duration. In addition, one dyad was excluded at the data-analysis stage due to excessive fNIRS artifacts. During preprocessing, three bad channels was removed from all participants, resulting in an overall channel retention rate of 99.60%. Importantly, exclusion rates did not differ between gain and loss contexts (*p* = 0.869), minimizing the likelihood of condition-specific bias.

**Fig. 3:**
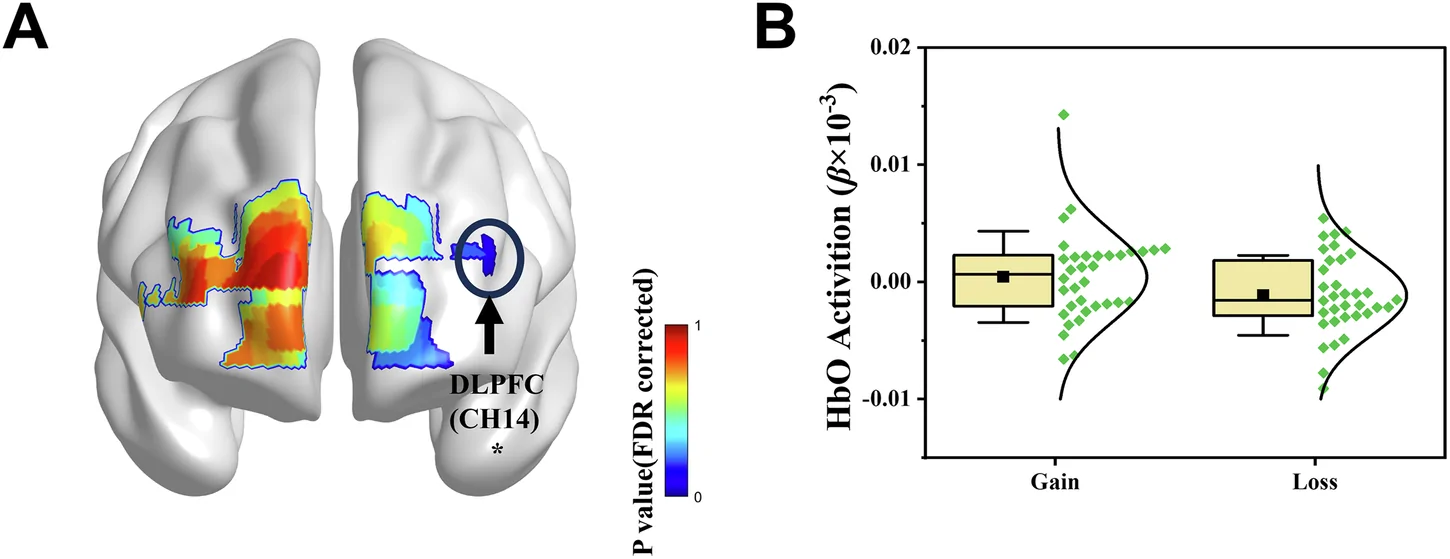
Context-dependent modulation of HbO responses in the PFC during deceptive trials. **A** Heatmap illustrating the statistical comparison of HbO *β* values across prefrontal channels between gain and loss contexts. The channel exhibiting a significant contextual difference is highlighted with a black circular outline and arrow. **B** Boxplot displaying the distribution of HbO *β* values in the identified channel under gain and loss contexts (***n*** = **34 healthy adults**). In the boxplots, green diamonds indicate individual data points, whereas black rectangles and black horizontal lines within the boxes indicate condition means. The curves adjacent to the boxes represent normal distribution curves. The same boxplot configuration is used in subsequent figures.

We next examined whether PFC hemodynamic responses during receiver-disadvantageous recommendations were modulated by gain-loss context. Given the low number of honesty trials, the primary analysis focused on deceptive recommendation trials, with oxygenated hemoglobin (HbO) responses extracted from the 15-s verbal statement phase. A significant contextual difference was observed only in the DLPFC (CH14, Fig. 3A). Specifically, the activation intensity of DLPFC was significantly higher in the gain context than in the loss context [×10⁻⁵; loss vs. gain: −1.14 ± 3.40 vs. 0.43 ± 3.89, *t*_(33)_ = 2.565, *p* = 0.015, *Cohen’s d* = 0.440, Fig. 3B]. As an exploratory check, we also examined contextual differences during honesty trials. No significant contextual differences were observed in any channel (*p*s > 0.162; Supplementary Table 2). However, given the small number of honesty trials, this analysis should be interpreted cautiously and was not treated as a sufficiently powered deception > honesty comparison.

### INS results

To control for the potential confounding effect of direct eye contact on ΔINS, we examined whether eye-contact frequency differed across contexts. Three sets paired-samples *t* tests on the mean number of direct eye-contact episodes during the verbal statement phase revealed no significant contextual differences (*p*s > 0.067; see Table 1). This pattern is consistent with prior findings^6^, suggesting that the experimental manipulation did not alter the frequency of direct eye contact.

**Table 1:** Contextual differences in direct eye contact during the verbal statement phase

Condition	Gain	Loss	*t*	*p*	*Cohen’s d*
Honesty	13.26 ± 6.86	14.71 ± 6.72	–0.928	0.360	–0.159
Deception	46.47 ± 18.05	42.56 ± 15.12	1.039	0.306	0.178
Successful Deception	35.65 ± 16.38	42.56 ± 15.12	–1.893	0.067	−0.325

A data-driven analysis was conducted across the 0.01–0.5 Hz frequency range by comparing task-phase INS (verbal statement) with resting-state INS^43^. After FDR correction, significant task-related increases were identified in the 0.0202-0.0539 Hz band under the gain context (Supplementary Fig. 1A) and in the 0.0404–0.0719 Hz band under the loss context (Supplementary Fig. 1C). These bands were therefore selected for subsequent analyses. Landing frequency clustering across dyads further confirmed aggregation within these task-sensitive bands (Supplementary Fig. 1B, D).

**Fig. 4:**
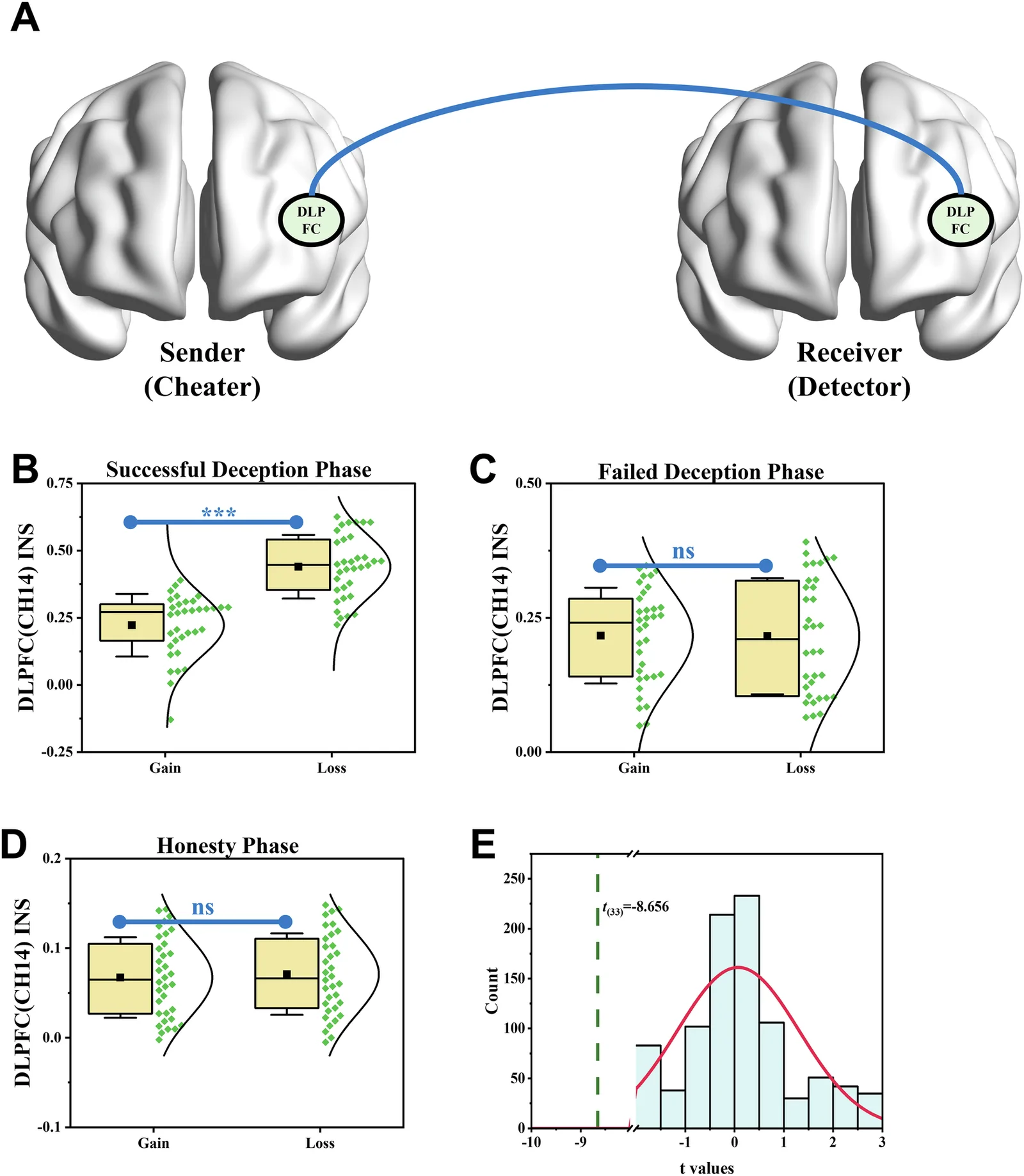
Task-level ΔINS results. **A** Visualization of channel pairs exhibiting significant contextual differences in ΔINS. **B** Comparison of ΔINS in channel pairs showing significant contextual differences during successful deception trials (*n* = 34). **C** Comparison of ΔINS in the same channel pair during failed deception trials across contexts (*n* = 34). **D** Comparison of ΔINS in the same channel pair during honesty trials across contexts (*n* = 34). **E** Results of the permutation test with 1000 label shuffles. The bars indicate the frequency distribution of t values obtained from the pseudo-dyad permutation test, the red curve represents the normal distribution curve, and the dashed line indicates the t value obtained from the real dyad test.

During successful deception trials, ΔINS between bilateral DLPFC channels (CH14–CH14) was significantly modulated by context. Specifically, ΔINS was significantly higher in the loss context compared to the gain context [loss vs. gain: 0.44 ± 0.12 vs. 0.22 ± 0.12, *t*_(33)_ = -8.656, *p* < 0.001, *Cohen’s d* = -1.485, Fig. 4A, B]. Importantly, no significant contextual differences were observed in the same channel pair during honesty trials [loss vs. gain: 0.07 ± 0.05 vs. 0.07 ± 0.04, *t*_(33)_ = 0.054, *p* = 0.957, *Cohen’s d* = -0.055, Fig. 4D] or failed deception trials [loss vs. gain: 0.22 ± 0.11 vs. 0.22 ± 0.09, *t*_(33)_ = -0.320, *p* = 0.751, *Cohen’s d* = 0.009, Fig. 4C]. These results indicate that gain–loss framing modulated ΔINS during successful receiver-disadvantageous recommendations. However, given the limited number of honesty trials and the absence of a sufficiently powered deception > honesty contrast, this pattern should not be interpreted as demonstrating deception-specificity. Permutation testing (1,000 iterations) confirmed that the empirical t-value exceeded the null distribution (*p*s < 0.001,Fig. 4E), indicating that the effect was not driven by random pairing.

### Trial-level ΔINS predicts the outcomes of deceptive recommendations

To evaluate whether trial-level ΔINS carried information about the outcome of deceptive recommendations, we applied two classification algorithms—Logistic Regression and SVM—to examine whether ΔINS predicted whether a receiver-disadvantageous recommendation was adopted or rejected by the receiver. Results indicated that, under both gain and loss contexts, the Logistic Regression model performed significantly above chance. Model performance was best in the loss context (Accuracy=0.884, AUC = 0.611, F1 score = 0.638). Performance metrics for all conditions and models are summarized in Table 2 and Supplementary Fig. 2. To further assess the statistical robustness of model performance, we conducted 1,000 permutation tests. The Logistic Regression model significantly outperformed the null distribution generated by label shuffling (*p*s < 0.001), confirming that the observed predictive accuracy was unlikely to arise by chance.

**Table 2:** Performance evaluation of two ML algorithms across context

Model	Condition	ROC-AUC	Accuracy	Precision	Recall	F1 Score
Logistic Regression	Gain	0.607	0.795	0.487	0.918	0.636
	Loss	0.611	0.884	0.476	0.971	0.638
SVM	Gain	0.404	0.471	0.211	0.621	0.315
	Loss	0.391	0.446	0.104	0.556	0.175

### Trial-by-trial ΔINS time-series dynamics

**Fig. 5:**
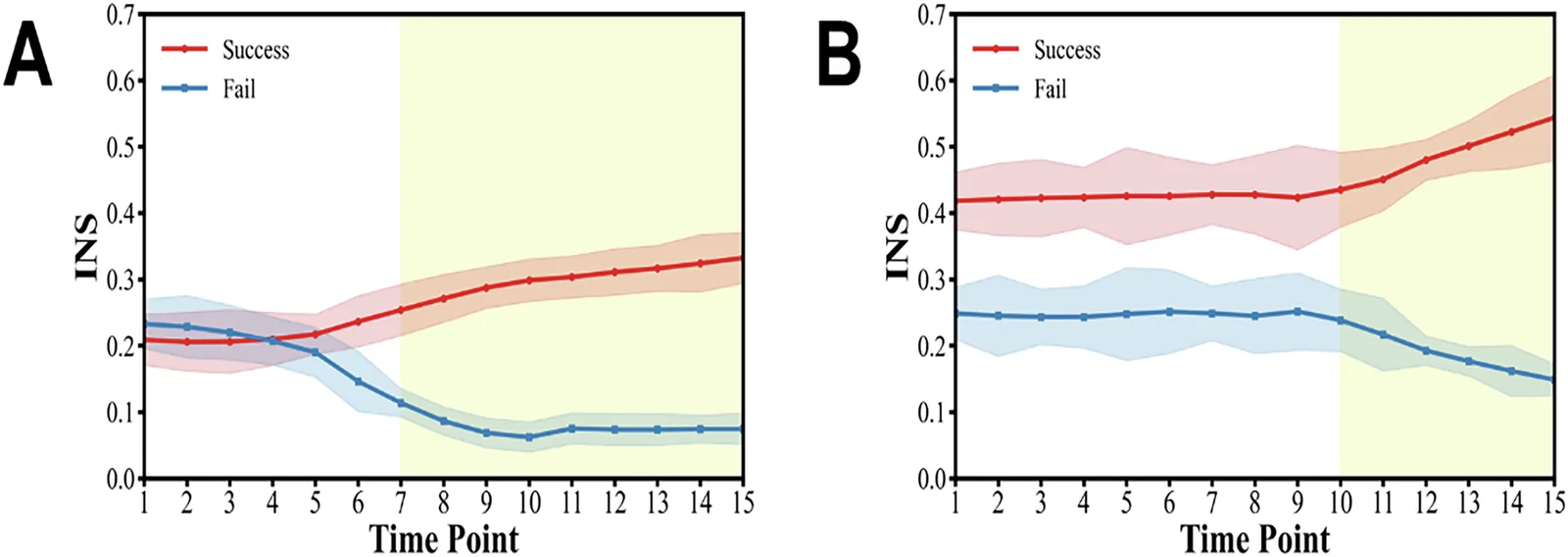
Trial-by-trial ΔINS time-series dynamics during successful and failed deception across contexts. **A** Gain context; **B** loss context. The red line represents ΔINS during successful deception trials, and the blue line represents ΔINS during failed deception trials. Shaded regions indicate 95% confidence intervals. Yellow-highlighted regions indicate significant ΔINS differences.

To further characterize the dynamic neural mechanisms underlying evaluative processes during deception, we conducted time-series analyses of ΔINS for successful and failed deception trials (Fig. 5). Results revealed that: (1) In the DLPFC, significant difference in INS between successful and failed deception trials emerged 7 seconds after the onset of the verbal interaction in the gain context. This suggests that INS begins to differentiate deception outcomes relatively early during interpersonal interaction. (2) In the loss context, significant ΔINS differences emerged later, around the 10th second of the verbal statement, and persisted through the end of the task.

### Brain-behavior correlation

To examine whether ΔINS was associated with deception behavioral performance, Pearson correlation analyses were conducted separately for gain and loss contexts. Results revealed that ΔINS during successful deception was significantly associated with successful deception rate in both contexts. In the gain context, a negative correlation was observed (*r* = -0.934, *p* < 0.001). In the loss context, ΔINS was also significantly correlated with successful deception rate (*r* = -0.442, *p* = 0.009). In contrast, ΔINS during failed deception trials was not significantly associated with failed deception rate in either context (gain: *r* = 0.264, *p* = 0.131; loss: *r* = 0.074, *p* = 0.676).

We further directly compared the two context-specific brain–behavior correlations. Because the two correlations were obtained from the same dyads but did not share variables, they were compared as dependent, non-overlapping correlations using the cocor procedure. The gain-context association was significantly stronger than the loss-context association, Pearson and Filon’s *z* = –3.487, *p* < 0.001, with Zou’s 95% confidence interval for the difference excluding zero, 95% CI [–0.816, -0.243]. This result indicates that the DLPFC ΔINS–successful deception rate association was significantly stronger in the gain context than in the loss context.

Because the zero-order correlation in the gain context was unusually large, we conducted additional robustness analyses to examine whether this association was driven by methodological or behavioral confounds. After controlling for the number of valid trials, mean verbal-statement duration, and variability in verbal-statement duration, the association between DLPFC ΔINS during successful receiver-disadvantageous recommendations and successful deception rate remained significant (partial *r* = –0.731, *p* < 0.001). This association was further supported by Spearman rank correlation (*ρ* = –0.774, *p* < 0.001), bootstrap resampling (95% CI [–0.888, –0.571]), and robust regression (*β* = -0.491, *p* < 0.001). Leave-one-dyad-out sensitivity analysis further showed that the correlation remained stable after each dyad was excluded in turn, with r values ranging from –0.952 to –0.929.

### Regression analyses: unique predictive contribution of ΔINS

To determine whether ΔINS provides predictive value beyond single-brain indices and demographic factors, we conducted hierarchical linear regression analyses separately for gain and loss contexts. Demographic variables, single-brain activation measures, and ΔINS were entered sequentially to evaluate their incremental explanatory power. As shown in Supplementary Table 3, significant improvements in model fit were observed only when ΔINS during successful deception was entered into the model (Δ*R*²_gain_ = 0.576, Δ*F*_gain_ = 54.093, *p* < 0.001; Δ*R*²_loss_ = 0.138, Δ*F*_loss_ = 6.248, *p* = 0.018). In contrast, neither single-brain activation measures (Δ*F* < 0.116, *p*s > 0.736) nor ΔINS during failed deception (Δ*F* < 2.410, *p*s > 0.132) significantly increased explained variance.

To further verify the robustness of this effect, we conducted confirmatory analyses by reversing the order of predictor entry. ΔINS during successful deception was entered first, followed by ΔINS during failed deception, single-brain activation measures, eye-contact frequency during successful deception, and finally demographic variables. As presented in Supplementary Table 4, none of the subsequently entered variables significantly improved model fit (failed-deception ΔINS: Δ*F* < 1.390, *p*s > 0.247; single-brain measures: Δ*F* < 0.159, *p*s > 0.693; eye contact: Δ*F* < 3.842, *p*s > 0.059; demographics: Δ*F* < 1.292, *p*s > 0.290).

### Predicting successful deception from ΔINS: comparative ML analyses

To further characterize the brain–behavior relationship, we implemented exploratory ML approaches. Based on the preceding findings, ΔINS during failed deception and single-brain activation measures neither correlated significantly with deception outcomes nor contributed incremental explanatory power in hierarchical regression models. We therefore hypothesized that ΔINS during successful deception constitutes the critical neural predictor of deceptive performance.

Separate predictive models were constructed for gain and loss contexts, with ΔINS during successful deception entered as input features and successful deception rate as the target variable. To identify the optimal predictive framework, we systematically compared multiple ML algorithms encompassing both linear and non-linear approaches. Across contexts, LSR demonstrated superior predictive performance[gain context: ROC-AUC = 0.929, Accuracy=0.727, Supplementary Fig. 3A; loss context: ROC-AUC = 0.867, Accuracy=0.727, Supplementary Fig. 3B]. Detailed performance metrics for all models are reported in Table 3. Using the LSR model, ΔINS within the significant channel pair reliably predicted successful deception across contexts (gain: *R*² = 0.870, *MSE* = 0.001; loss: *R*² = 0.266, *MSE* = 0.004). To assess statistical significance, we conducted 5,000 label-shuffled permutation tests. In both contexts, the observed predictive performance exceeded chance levels (gain: *r* = 0.933, *p* < 0.001, Supplementary Fig. 3C; loss: *r* = 0.516, *p* < 0.001, Supplementary Fig. 3D).

**Table 3:** Performance evaluation of different ML models

Model	Condition	ROC-AUC	Accuracy	Precision	Recall	F1 Score	MCC
LSR	Gain	0.929	0.727	0.750	0.857	0.800	0.386
	Loss	0.867	0.727	0.800	0.667	0.727	0.467
Logistic Regression	Gain	0.893	0.909	0.875	0.796	0.733	0.810
	Loss	0.800	0.636	0.667	0.667	0.667	0.817
Naive Bayes	Gain	0.768	0.727	0.650	0.757	0.700	0.386
	Loss	0.733	0.727	0.800	0.667	0.727	0.467
DT	Gain	0.732	0.677	0.833	0.714	0.669	0.449
	Loss	0.817	0.818	0.833	0.833	0.833	0.633
SVR	Gain	0.821	0.818	0.857	0.857	0.610	0.607
	Loss	0.667	0.545	0.600	0.500	0.545	0.100

## Discussion

The present study provides evidence that deception-related behavior, as operationalized by receiver-disadvantageous recommendations in the present task, is modulated by gain-loss contexts during social interaction. Behaviorally, loss contexts increased the frequency and success rate of such recommendations and were associated with shorter response times, suggesting that potential losses may facilitate more rapid selection of detector-disadvantageous options. At the single-brain level, DLPFC activation was higher during receiver-disadvantageous recommendation trials in gain contexts than in loss contexts, indicating context-dependent modulation of prefrontal control processes. However, this effect should not be interpreted as deception-specific activation, because the limited number of honesty trials precluded a sufficiently powered deception-versus-honesty comparison. More critically, the inter-brain results showed that contextual modulation of DLPFC ΔINS emerged selectively during successful deception, but not during honest or failed deception trials. Trial-level analyses further showed that DLPFC ΔINS distinguished successful from failed deception in both gain and loss contexts, with earlier differentiation in gain contexts and more sustained differentiation in loss contexts. Finally, hierarchical regression and cross-validated model comparison indicated that DLPFC ΔINS explained unique variance in deception success beyond single-brain activation and direct eye contact. Together, these findings suggest that gain-loss contexts shape deception-related outcomes not only through individual prefrontal regulation, but also through the alignment of control-related processes between interaction partners.

Behaviorally, gain-loss contexts modulated deception-related choices during interpersonal interaction. Senders made more receiver-disadvantageous recommendations in loss contexts than in gain contexts, consistent with Prospect Theory’s claim that losses carry greater psychological weight than equivalent gains^8^ and with evidence that potential losses can increase dishonest or norm-violating behavior aimed at avoiding negative outcomes^12^. Response-time results further showed that loss contexts were associated with faster decision implementation: senders made receiver-disadvantageous recommendations faster than honest recommendations, and receivers accepted such recommendations faster than they rejected them. This pattern accords with the view that loss contexts can promote rapid, threat-sensitive processing^35^, whereas gain frames often involve more deliberative value comparison^7^. Nevertheless, these effects should be interpreted within the task incentive structure. Because the payoff matrices created direct conflicts of interest and only one trial per context was selected for payment, senders may have developed relatively stable strategic tendencies across trials. Thus, the results indicate that gain-loss framing modulated the frequency, speed, and success of receiver-disadvantageous recommendations, rather than providing evidence for fully independent trial-wise decisions to deceive.

The neural findings suggest that gain-loss contexts shaped deception-related outcomes through both individual prefrontal control and dyadic control alignment. At the individual level, DLPFC activation during receiver-disadvantageous recommendation trials was stronger in gain contexts than in loss contexts. Given the limited number of honesty trials, this effect should not be interpreted as deception-specific activation. Rather, it suggests that gain contexts increased the control demands required to implement receiver-disadvantageous recommendations. This interpretation is consistent with evidence that the DLPFC is preferentially recruited when decisions require goal maintenance, response inhibition, strategic monitoring, and contextual regulation^21,24,26^, as well as with accounts of prefrontal control in multi-cue tradeoffs and norm–benefit conflicts^17,22^. Thus, whereas loss contexts promoted more frequent and faster receiver-disadvantageous recommendations, gain contexts appeared to require greater individual-level control.

Inter-brain results extend this account by showing that deception success was associated with the alignment of control-related processes between partners. DLPFC ΔINS showed contextual modulation selectively during successful deception, but not during honest or failed deception trials, arguing against a simple explanation based on general interaction intensity or verbal engagement. Instead, this pattern suggests that gain-loss contexts modulated the degree to which control-related processes became synchronized when receiver-disadvantageous recommendations successfully influenced the receiver’s choice. This interpretation is consistent with hyperscanning evidence linking DLPFC synchrony to strategic coordination, negotiation, competition, and shared behavioral adjustment^6,34^, and with the view that DLPFC-related INS reflects shared regulation of rules, goals, and strategies rather than emotional resonance alone^32^. Because speech recordings were unavailable, however, we cannot determine whether enhanced DLPFC ΔINS reflected specific verbal tactics, stable dyadic strategies, or moment-to-moment persuasive adjustments. Therefore, DLPFC ΔINS should be interpreted as evidence for dyadic control alignment during successful receiver-disadvantageous influence, rather than as a marker of a specific communicative strategy.

Furthermore, ΔINS not only distinguished successful from failed deception statistically but also demonstrated significant predictive power in both contexts, with higher classification accuracy in loss contexts. This suggests that INS is not a post-hoc averaged group effect but carries real-time information about decision outcomes within single interactions^44,45^. More importantly, time-series analysis revealed distinct temporal dynamics: in gain contexts, INS differences between success and failure emerged early (approximately 7 s), suggesting that in context requiring higher strategic monitoring, interacting partners enter a state of control alignment earlier^46^. In contrast, in loss contexts, differences appeared later (approximately 10 s) but persisted until the end of the trial, indicating that synchronization may not be a prerequisite for the initiation phase but rather strengthens progressively to consolidate decisions and maintain influence^47,48^. Taken together, the task-level and trial-level INS results jointly indicate that successful deception relies on the cross-brain synergy of control networks between interacting partners rather than merely individual control resource investment^49^. Gain and loss contexts shape the timing and intensity of this synergy by modulating decision rhythms and motivations, reflecting differentiated interactive regulation mechanisms at the neural level.

ML analysis advanced these associations from “explanation” to “generalizable prediction.” Cross-algorithm comparisons showed that the LSR model performed robustly in both contexts, indicating that ΔINS contains reproducible, cross-model robust behavioral information rather than accidental fitting. Simultaneously, the higher explanatory power and stronger negative correlation observed in gain contexts align with the aforementioned dynamic evidence that “deception in gain frames relies more on high-cost strategic monitoring and earlier synchronization differentiation.” In gain contexts, INS appears to be a core variable determining individual differences, whereas in loss contexts, where behavior is driven more by approach-avoidance motivation, ΔINS remains predictive but has more limited explanatory power. This confirms that gain-loss contexts modulate not only the pathway of deception execution but also the efficiency of interactive alignment that influences successful deception.

Several limitations should be noted. First, the 15-s open verbal interaction phase enhanced ecological validity but introduced semantic uncertainty. Because speech recordings and verbatim coding were unavailable, deception was defined operationally by the payoff structure—that is, receiver-disadvantageous recommendations—rather than by confirmed linguistic falsehoods. Second, although multi-step preprocessing and trial-level quality screening were applied, fNIRS signals during naturalistic speech may still be influenced by head motion, facial movement, articulation, and superficial blood flow. Accordingly, prefrontal activation and INS effects should be interpreted as ecological functional patterns rather than fine-grained localization of deception-specific neural circuits. Third, the incentive structure may have encouraged relatively stable response tendencies, and the high rate of receiver-disadvantageous recommendations left too few honesty trials for reliable deception-versus-honesty neural comparisons. Therefore, this study should not be interpreted as identifying the neural basis of deception per se, but as showing how gain-loss contexts modulate prefrontal activity and INS during receiver-disadvantageous recommendations in a high-conflict interaction task. Future studies should incorporate speech recording, semantic coding, trial-wise feedback, and more balanced honest/deceptive choices to test deception-specific mechanisms more directly.

In summary, by integrating behavioral measures, single-brain activation, and inter-brain synchronization within an ecologically valid interaction paradigm, this study provides evidence for a context-sensitive interpersonal neural account of receiver-disadvantageous recommendations. The findings suggest that gain–loss framing shapes both the behavioral implementation of such recommendations and the prefrontal interpersonal synchrony associated with their success. More broadly, this work advances the use of INS as a core metric for explaining and predicting interactive social influence, supporting a shift in social neuroscience from correlational description toward mechanistic modeling and behavioral prediction.

## Methods

### Participants

Participants were healthy, right-handed adults with normal or corrected-to-normal vision and were native Chinese speakers. All participants were recruited online to enhance sample representativeness. A priori power analysis was conducted using G*Power 3.1.9.7^50^ (medium effect size *f* = 0.25, *α* = 0.01, 1-*β* = 0.80, paired-samples *t* test), indicating a minimum required sample size of 34 dyads. Initially, 37 same-gender dyads composed of previously unacquainted individuals were formed from eligible participants. One dyad was excluded due to insufficient fNIRS data quality, and two dyads were excluded based on a predefined high-honesty criterion, defined as an honesty rate exceeding 85% across the experiment, equivalent to a deception rate below 15%^25^. The final sample therefore consisted of 34 same-gender stranger dyads, including 18 female dyads (age: *M* = 21.33, *SD* = 2.07) and 16 male dyads (age: *M* = 20.56, *SD* = 1.29). Prior to the experiment, experimenters confirmed that members within each dyad had no prior acquaintance and were meeting for the first time. To further validate the effectiveness of the stranger-dyad manipulation, participants completed the single-item Inclusion of Other in the Self (IOS) scale to assess perceived interpersonal closeness (7-point Likert scale, range from 1 to 7). All participants reported IOS scores below 2 (*M* = 1.04, *SD* = 0.21), indicating successful manipulation of stranger relationships. The study employed a sender–receiver spontaneous deception paradigm adapted from Chen et al.^25^. Roles (sender/cheater vs. receiver/detector) were randomly assigned within each dyad and remained fixed throughout the experiment. The protocol was approved by the Medical Ethics Committee of North China University of Science and Technology (No. 2023159), and written informed consent was obtained from all participants. All ethical regulations relevant to human research participants were followed.

Participant compensation consisted of two components. Each participant received a fixed base payment of 15 RMB upon completion of the experiment. To enhance the ecological validity of gain and loss contexts while avoiding excessive variability in participant compensation, one trial was randomly selected from each context at the end of the task to determine the performance-based bonus. This payment rule preserved incentive compatibility at the task level but did not accumulate payoffs across all trials. Therefore, the present task was not designed to assess learning-based adjustment of deception strategies across repeated decisions. The performance-based bonus was defined as *R*_gain_-*R*_loss_. If this value was less than or equal to 0, participants received a uniform minimum bonus of 1 RMB; otherwise, they received the corresponding positive bonus. Under this rule, all participants received compensation for their time in addition to the fixed base payment of 15 RMB, and no participant was required to repay or forfeit any part of the base payment. In the present study, the actual task bonus ranged from 1 to 20 RMB, with a mean of 10.79 RMB and a median of 10.00 RMB. Accordingly, no participant received 0 RMB, and the extreme total payment of 45 RMB implied by the original theoretical range did not occur; the highest actual total payment was 35 RMB, received by 12 participants. All experimental procedures and payment rules were clearly explained to participants prior to the experiment, and no participant was required to incur any additional costs. The performance-based compensation structure followed the protocol established in previous studies^6,25^.

### Experimental setup, task procedure and materials evaluation

The study employed a single-factor within-subject experimental design, with contextual framing (gain vs. loss) as the within-subject independent variable. All participants completed the task under both gain and loss conditions. To minimize potential order and fatigue effects associated with repeated task exposure, an ABBA block structure was implemented at the within-subject level. In addition, the initial context was counterbalanced across participants, such that half of the participants first completed the task in the gain context, whereas the other half began in the loss context. This control strategy was adopted to reduce potential confounding effects of contextual order on both behavioral and neural measures.

#### Setup

**Fig. 6:**
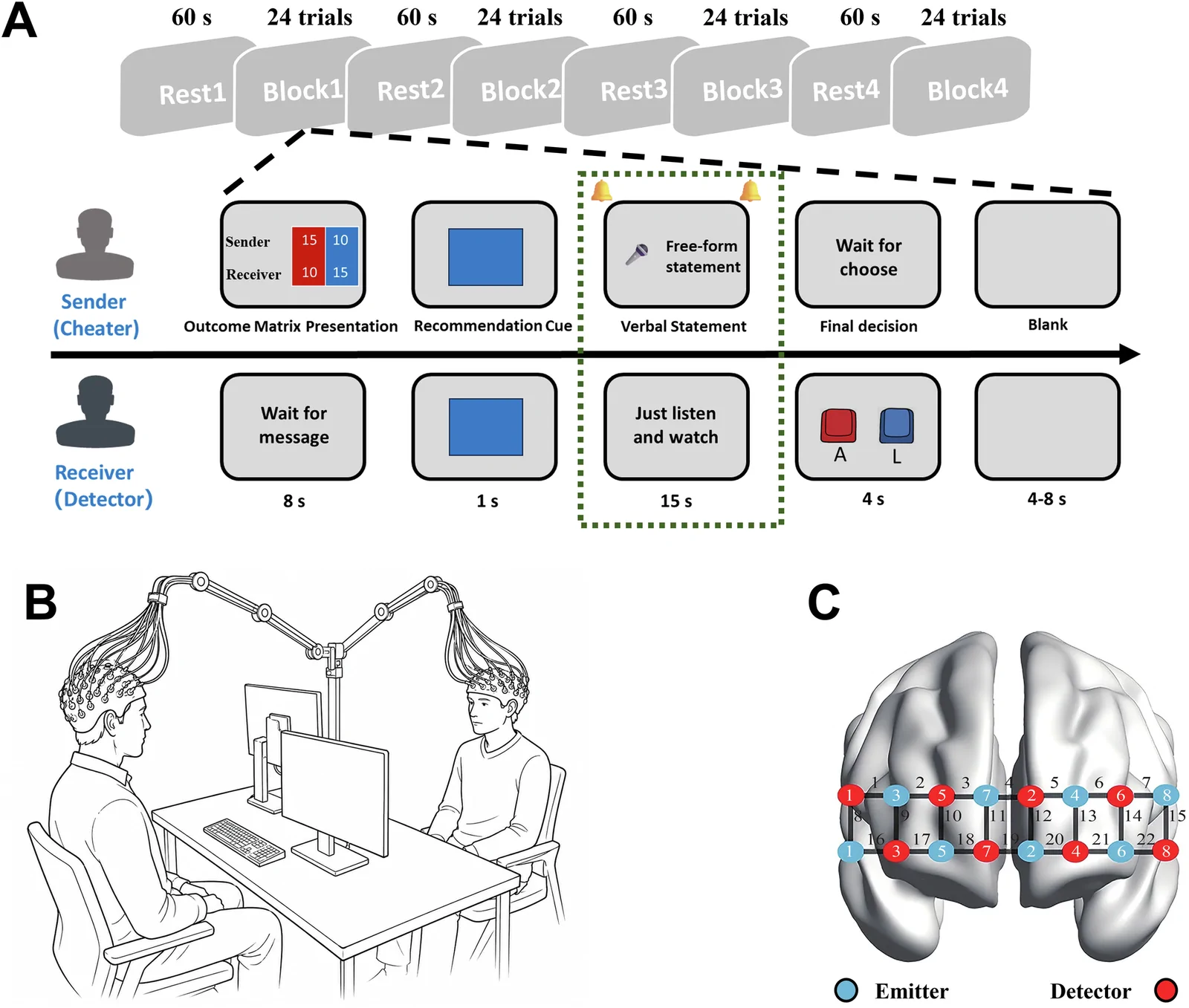
Experimental design and setup. **A** Task structure used in the present study. Each trial consisted of four sequential stages: outcome matrix presentation, recommendation cue, verbal statement, and final decision. The green dashed box highlights the task time window of interest (15 s) that was analyzed in the current study. **B** Experimental setup. Schematic illustration of the seating arrangement and diagonally positioned monitors (Hand-drawn line illustration by the authors of this article.). Participants maintained fixed roles throughout the experiment. This arrangement minimized unnecessary eye contact during non-task period. **C** fNIRS data acquisition setup.

The experiment was conducted in a fNIRS laboratory. Dyads were seated face-to-face at a diagonal angle, with monitors positioned diagonally between them. This configuration allowed participants to naturally access each other’s facial and nonverbal cues (e.g., facial expressions and gaze direction) while performing the task, without inducing prolonged, fixed, or socially meaningless eye contact. Importantly, the monitors did not fully occlude the participants’ view of one another. Therefore, the interaction retained face-to-face characteristics, while minimizing excessive social arousal that arises from direct gaze. This setup was designed to preserve ecological validity at the interpersonal level while maintaining experimental control (see Fig. 6B).

#### Subjective measures

(1) IOS. Perceived interpersonal closeness toward the interaction partner was assessed and administered both before and after the experiment. Given that all dyads consisted of previously unacquainted individuals, IOS scores were expected to be low prior to the experiment (i.e., below 2), an expectation that was confirmed by the present data. (2) Measures for detectors. Previous research has identified higher gullibility^51^ and lower vigilance^52^ as key personality traits associated with increased susceptibility to deception. Accordingly, detectors completed two questionnaires prior to the experiment. (a) Gullibility and susceptibility to deception were assessed using the Social Vulnerability Scale (SVS-15)^53^, a 0–4 Likert-type scale in which higher scores indicate a higher frequency of vulnerable behaviors. (b) Vigilance-related traits were assessed using the introspection and reactivity subscales of the Hyperarousal Scale (HAS)^54^. Items were rated on a 0–3 scale, with higher scores indicating a higher tendency toward heightened vigilance. (3) Perceived realism of contextual manipulation. After completing the task, participants rated the perceived realism of the contexts on separate 9-point Likert scales, with higher scores indicating higher subjective realism.

#### Task procedure

The experimental task was adapted from the sender–receiver spontaneous deception paradigm developed^25^. Following completion of the pre-task subjective measures, participants entered a preparation phase, which began with a 60-s baseline recording. During this period, participants were instructed to fixate on a central “+” symbol, remain relaxed, and minimize head movements^55^. Participants then completed a practice phase comprising four trials, including two randomly selected outcome matrices from the gain context and two from the loss context. The formal experiment commenced only after both members of the dyad demonstrated a clear understanding of the task rules, role assignments, and payoff structure. The procedure of the formal experiment was identical to that of the practice phase and consisted of the following stages (see Fig. 6A): (1) Outcome matrix presentation (8 s). The sender (potential information manipulator) viewed an outcome matrix displaying the potential outcomes for both players. The first and second rows corresponded to the sender’s and receiver’s outcomes, respectively. Red and blue colors denoted Option A and Option L. (2) Recommendation cue (1 s). The sender conveyed a brief nonverbal recommendation to the receiver using a color cue (red or blue), semantically equivalent to “the option associated with this color is more beneficial for you in this round.” (3) Verbal statement (15 s). Following an auditory cue (1000 Hz, 500 ms), the sender delivered a one-way verbal statement. The first sentence was required to reiterate that the recommended option was “more beneficial” for the receiver, after which the sender could freely provide task-relevant information. During this phase, the receiver remained silent and was not permitted to ask questions. A second auditory cue signaled the sender to immediately stop speaking. (4) Final decision (within 4 s). The receiver made the final choice, which determined the monetary outcome for both players in that trial. The experiment comprised four blocks of 24 trials each, with gain and loss contexts alternating in an ABBA order. A 1-min rest period was provided between blocks, and the inter-trial interval varied randomly between 4 and 8 s. The total duration of the experiment was approximately 45 min. Stimulus presentation and data acquisition were implemented using custom Python scripts. Trials in which participants failed to respond within the prescribed time window were automatically terminated and excluded from subsequent analyses. After completing the task, both participants completed the IOS scale and a post-task manipulation check assessing perceived contextual realism.

In each trial, the payoff structure was explicitly designed to induce a direct conflict of interest between the sender and receiver: a higher payoff for the sender necessarily corresponded to a lower payoff for the receiver, and vice versa. This structure was identical in both gain and loss contexts. Importantly, only one option in each trial was objectively advantageous for the receiver (i.e., higher gain or lower loss), whereas the alternative option was disadvantageous. This design has been shown to effectively elicit spontaneous information manipulation based on immediate payoff trade-offs^25^. Participants were explicitly informed that deceptive or misleading communication was permitted in the task. Receivers could not view the numerical values in the outcome matrix and relied solely on the sender’s recommendation cues (color signals and verbal statements), rendering direct verification of information impossible^6^. Senders received no feedback during or after the experiment regarding whether their recommendations influenced the receiver’s decision. This design minimized explicit punishment, reputation management, and feedback-based learning. Accordingly, the present task was intended to capture how receiver-disadvantageous recommendations were implemented under controlled gain-loss contexts, rather than to model adaptive trial-by-trial strategy updating based on outcome feedback. Because the semantic content of verbal statements was not constrained, deception could not be conclusively identified at the single-trial level. Accordingly, deception was operationally defined based on whether the sender’s recommendation was objectively disadvantageous for the receiver given the payoff structure, rather than on direct inference of the sender’s subjective intent.

#### Materials evaluation

Based on prior evidence indicating that monetary payoff configurations within outcome matrices significantly modulate information manipulation behavior^25^, the present study extended the original task materials accordingly. Specifically, loss-context matrices were constructed by introducing negative values into the gain-context payoff matrices. Participants were explicitly informed prior to the experiment that negative values represented actual monetary losses and would directly affect their final compensation. This manipulation has been widely adopted in previous research to operationalize gain-loss framing effects^56–58^, with the primary goal of isolating differences in value representation during decision processing rather than simulating the full complexity of real-world economic environments in which gains and losses may co-occur. Accordingly, the present design focuses on contrasting framing-dependent valuation mechanisms under controlled conditions, while holding constant the underlying payoff structure across contexts.

### fNIRS data acquisition

The researchers used two sets of optodes to cover each participant’s PFC, an area associated with deception and detection^59,60^. For each participant, a 2×8 photovoltaic array (8 emitters and 8 detectors, spaced 3 cm apart) was placed on the PFC with a reference photodiode located at Fpz (Fig. 6C). The coherence of the photovoltaic array was checked and adjusted. In the experiment, the LIGHTNIRS fNIRS system (Shimadzu Corporation, Japan; wavelengths: 780, 805, and 830 nm) measured 22 channels on the PFC of each dyad at a sampling rate of 13.3 Hz. Using the modified Beer-Lambert’s law, the optical data for each channel were converted to changes in the concentrations of HbO and deoxy-hemoglobin (HbR). The focus of this study was on HbO concentration, which is the most sensitive indicator of changes in cerebral blood flow in fNIRS measurements^61^. Channel localization data were collected using a 3D digitizer, and spatial alignment of NIRS_SPM provided the cortical location of the channels (Table 4).

**Table 4:** Anatomical correspondence between fNIRS channels and PFC

Anatomical label	Channels
Dorsolateral prefrontal cortex	1、2、6、7、9、14
Frontopolar area	3、4、5、10、11、12、13
Ventrolateral prefrontal cortex	8、15、16、22
Orbitofrontal area	17、18、19、20、21

### Coding of direct eye contact

Direct eye contact was defined as fixation on the interaction partner’s eyes for more than 500 ms during the verbal statement phase^25^. Four independent coders (two females) annotated eye contact events following standardized training. Videos were temporally segmented, frame-rate normalized (25 fps), and synchronously presented for dual-participant observation. Inter-rater reliability for the mean frequency of direct eye contact was high, as indexed by the intraclass correlation coefficient (ICC = 0.826).

### Data analysis

#### Manipulation check

To ensure adequate task engagement and effective contextual manipulation, participants rated the realism of gain and loss contexts on a 9-point scale (range from 1 to 9). Those scoring below 5 in both contexts were excluded from analysis.

#### Subjective measures analysis

Given that the present study included dyads of different genders, chi-square tests and independent-samples *t* tests were conducted to examine whether male and female dyads were comparable at baseline. Baseline variables included demographic characteristics (e.g., age, educational background, and monthly disposable income), as well as detectors’ scores on the SVS and the HAS. Additionally, paired-samples t-tests were applied to assess changes in IOS scores from pre- to post-experiment within dyads of different genders.

#### Behavioral data analysis

In the present study, senders (i.e., cheaters) were free to decide whether to deceive, resulting in two behavioral categories: honesty and deception. A deceptive attempt was classified as successful deception if the detector’s final decision led to a higher gain or a smaller loss for the cheater. Conversely, deception was classified as failed deception if the detector’s decision resulted in a higher gain or a smaller loss for the detector.

Based on these definitions, four primary behavioral indices were computed: (1) Honesty rate. The proportion of trials in which the sender provided truthful and detector-beneficial information. To ensure that deception-related behavioral and neural analyses were based on a sufficient number of deceptive trials, dyads with an honesty rate exceeding 85% across the experiment were excluded according to the predefined high-honesty criterion^25^. This criterion was used only to remove extreme high-honesty cases and was not intended to select dyads with unusually high deception rates. (2) Deception rate. The proportion of trials in which the sender provided information that was objectively disadvantageous to the receiver. (3) Successful deception rate. The proportion of deceptive trials in which the receiver ultimately obtained a lower gain or incurred a higher loss. (4) Reaction time. Given the focus on the implementation of sender recommendations, senders’ reaction times for honest and deceptive recommendations were recorded separately under gain and loss contexts. Behavioral data were analyzed using paired-samples *t*tests. Statistical significance was set at *p* < 0.05, with p values corrected for multiple comparisons using the Bonferroni. Effect sizes are reported as partial eta squared (${\eta }_{p}^{2}$).

#### fNIRS data analysis

Statistical analyses of the fNIRS data largely followed the procedures used for behavioral analyses, with the exception that *p* values were corrected for multiple comparisons using the False Discovery Rate (FDR) procedure^62^. Because honesty trials were extremely sparse in the present paradigm, they did not provide sufficient observations for reliable estimation of fNIRS activation, task-level INS, or trial-level predictive models. Therefore, the primary fNIRS analyses focused on trials in which senders provided receiver-disadvantageous recommendations. These analyses were intended to examine context-dependent neural modulation within deceptive interactions, rather than to provide a direct deception> honesty contrast. Given the unconstrained nature of verbal statements during the speech phase, post hoc trial-level screening was performed. Specifically, trials were excluded if the sender’s effective speaking duration was abnormally short or prematurely terminated (defined as durations shorter than M-3×SD), or if the speaking duration exceeded the predefined 15-s limit (in which case the experimenter intervened). These trials were not included in subsequent neural analyses.

#### Preprocessing

fNIRS data were preprocessed in MATLAB using the Homer2 toolbox. Low-quality channels were identified and excluded using the enPruneChannels function. Dyads were removed from further analyses if the proportion of bad channels exceeded 50%, in accordance with established criteria^43^. The preprocessing steps were as follows: (1) it detects motion artifacts using the hmrMotionArtifactByChannel function^63^. (2) Remove the initial 60 seconds of the signal to eliminate environmental variations. (3) Apply principal component analysis (PCA) to separate task-specific fNIRS signals^64^. (4) Convert raw light intensity data to optical density (OD) data. (5) Kurtosis-based wavelet filtering to remove motion artifacts with a threshold of 3.3^65^. (6) A fifth-order Butterworth bandpass filter with cutoff frequencies of 0.01 Hz and 0.5 Hz was used to remove signal drift and noise^66^. (7) OD data were converted to hemoglobin oxygenation concentration using BeerLambert’s law for further analysis^67^.

#### Intra-brain activation analysis

Using the SPM-based fNIRS analysis framework^63^, HbO time series were extracted from all 34 senders. Analyses focused on neural response patterns during the verbal statement phase (15 s), which constituted the primary task window of interest. Trial start times and durations were extracted to generate stimulus designs and convolved with hemodynamic response functions using NIRS-SPM. These predictive signals were fitted to the data using a generalized linear model to generate beta estimates for each parameter in the design matrix for each participant.

#### Task-level INS analysis

After preprocessing, we identified task-related INS in the frequency bands of interest. HbO data from both participants were extracted during resting and task phases, using continuous wavelet transform coherence (WTC) to calculate INS across 0.01–0.5 Hz^68^. Fisher-z transformed values underwent single-sample *t* tests to identify significant channel-frequency bands (*p* < 0.05) and further analysis to determine adjacent frequency bands^69^. We recalculated task-related ΔINS for each channel and trial within the identified frequency bands. Fisher-z transformation was applied to the averaged trial values for each dyad. The time series for INS analysis matched those used in intra-brain activation analysis.

#### INS validation analysis

We performed a non-parametric permutation test to confirm that increased INS during deception resulted from actual interactions. This involved generating 1000 pseudo-dyads by randomly pairing the time series of different participants and calculating INS for these pseudo-dyads^70^. The average ΔINS for true dyads was compared to the null distribution from the pseudo-dyads. Each participant’s time series was randomly shuffled 1000 times, and the INS data were reanalyzed to confirm the findings.

#### Trial-level ΔINS prediction of deception outcomes

To systematically examine the dynamic predictive value of trial-level ΔINS during deceptive interactions, ΔINS was computed on a single-trial basis from fNIRS signals and used to predict deception outcomes in the corresponding trials. Given the susceptibility of single-trial fNIRS signals to noise, we followed established procedures to enhance signal quality and stabilize INS feature extraction^6^. Specifically, (1) raw optical density signals were band-pass filtered at 0.01–0.1 Hz to preserve task-relevant low-frequency neural oscillations while removing high-frequency physiological noise; (2) motion artifacts were corrected using the wavelet minimum description length algorithm; (3) baseline drift was corrected using a 5-s pre-stimulus baseline for each trial; and (4) global component removal was applied to eliminate system-level components shared across channels.

After preprocessing, fNIRS data were segmented according to event markers, and signals during the verbal interaction phase (1–15 s) of each trial were extracted for ΔINS computation. Given the substantial variability inherent in single-trial fNIRS data^71,72^, analyses were restricted to interpersonal channels that exhibited significant task-level INS enhancement, thereby improving the stability and interpretability of trial-level estimates. Trial-level ΔINS was computed using the same method as in the task-level analysis and was extracted separately for successful and failed deception trials. To mitigate overfitting due to redundant features, trial-level ΔINS values were first Fisher z-transformed and then subjected to PCA. Principal components explaining more than 85% of the cumulative variance were retained for subsequent modeling^73^. Deception outcome at each trial (binary: successful deception = 0, failed deception = 1) was used as the dependent variable, with trial-level ΔINS feature vectors serving as predictors. Logistic regression was employed as the primary model to assess the linear predictive relationship between ΔINS and deception outcomes. In addition, Support Vector Machine (SVM) models were implemented to evaluate potential nonlinear associations between ΔINS and deception outcome.

All models were evaluated using a LOSO-CV scheme to ensure robust generalization across participants^74^. Model performance was quantified using (1) accuracy, (2) area under the receiver operating characteristic curve (ROC-AUC), and (3) F1 score. To determine whether model performance exceeded chance levels, permutation testing with 1,000 label shuffles was conducted.

#### Trial-level ΔINS time-series analysis

To further characterize the temporal dynamics underlying evaluative and behavioral modulation during deceptive information processing, a time-series analysis of trial-level ΔINS was conducted for successful and unsuccessful deception trials. Following the approach described by Jiang et al.^30^, trial-level ΔINS coherence was computed at 15 consecutive time points under each condition. Paired-samples t-tests were then performed to compare ΔINS values between successful and failed deception trials at each time point. To control for multiple comparisons across time points, FDR correction was applied.

#### Brain-behavior correlation analysis

Two-tailed Pearson correlation analyses were conducted to examine the relationship between ΔINS and behavioral outcomes during deceptive interactions under gain and loss contexts. For each dyad and condition, we calculated the number of valid trials, mean verbal-statement duration, and within-condition variability in verbal-statement duration, and then repeated the brain–behavior analyses using partial correlations controlling for these variables. Because verbatim transcripts were unavailable, semantic variability in verbal content could not be directly quantified; therefore, verbal-duration variability and direct eye-contact frequency were used as behavioral proxies for interaction variability. Spearman correlations, bootstrap confidence intervals, robust regression, and leave-one-dyad-out analyses were further conducted to assess whether the associations were driven by distributional assumptions, outliers, or influential dyads.

To test whether the brain–behavior association differed between gain and loss contexts, we compared the two context-specific correlations using the cocor package in R. Because both correlations were derived from the same dyads, they were treated as dependent correlations. We therefore used the non-independent correlation comparison implemented in cocor to examine whether the correlation between DLPFC ΔINS and successful deception rate differed significantly between the gain and loss contexts.

#### Regression analysis

To evaluate whether ΔINS provides incremental predictive power beyond demographic and intra-brain neural measures, hierarchical linear regression analyses were conducted. This approach was used to test whether dyadic ΔINS significantly improved the prediction of deceptive outcomes above and beyond established predictors. Predictors were entered into the model in a block-wise manner. In Step 1, demographic variables (e.g., gender and educational background) were included. Step 2 added interaction-related behavioral variables, specifically the frequency of direct eye contact during the interaction. In Step 3, task-related activation coefficients (*β* values) from channels showing significant effects were entered to account for intra-brain neural contributions. In Steps 4 and 5, ΔINS during failed deception and successful deception, respectively, were sequentially introduced. At each step, changes in model fit were evaluated to determine whether ΔINS explained additional variance beyond demographic, behavioral, and intra-brain neural measures, thereby assessing its unique predictive value for deceptive outcomes.

### Predicting successful deception using ΔINS: an ML approach

Given the relatively small sample size of the present study (*N* = 34), conventional train–test splits may yield performance estimates that are highly sensitive to sample partitioning, thereby compromising the stability of generalization assessment. To address this issue, we adopted a leave-one-out cross-validation (LOOCV) strategy. LOOCV has been widely recommended in small-sample neuroimaging studies, as it maximizes the use of available training data while providing an approximately unbiased estimate of model generalization performance^75,76^. In each cross-validation iteration, data from one dyad were held out as the test set, whereas data from the remaining N-1 dyads were used for model training. This procedure was repeated N times, ensuring that each dyad served exactly once as an independent test sample.

#### Model construction

ΔINS values were extracted from fNIRS channel pairs that exhibited significant contextual differences in the preceding statistical analyses and were used as input features for model construction. The successful deception rate served as the prediction target, capturing individual differences in deceptive performance across gain and loss contexts.

#### Model evaluation

To avoid dependence on a single modeling assumption, multiple commonly used ML algorithms were systematically compared within a unified LOOCV framework. These algorithms included Logistic Regression, LSR, DT, Naive Bayes, and SVR. Linear models (e.g., Logistic Regression and LSR) were primarily used to assess linear predictive relationships between ΔINS and successful deception rates, whereas nonlinear models (e.g., DT and SVR) were employed to capture potential nonlinear patterns. We further evaluated classification-related performance metrics derived from model predictions, including Accuracy, Precision, Recall, and the F1 score. Accuracy reflects the overall proportion of correct predictions; Precision indexes the reliability of predicted deceptive outcomes; Recall quantifies sensitivity to true deceptive behavior; and the F1 score represents the harmonic mean of Precision and Recall, providing a balanced measure that is particularly suitable for imbalanced class distributions or asymmetric misclassification costs^77,78^.

#### Model robustness assessment

Model robustness was further quantified by computing Pearson’s r between LOOCV-based predicted values and observed successful deception rates. This metric captures the model’s ability to account for individual differences in deceptive behavior at the continuous level. To assess the statistical significance of the observed prediction performance, permutation testing was conducted to construct a null distribution. In each permutation, deception-rate labels were randomly shuffled while feature values were held constant, and the entire prediction procedure was repeated. This process was iterated 1000 times to generate a distribution of correlation coefficients expected under chance-level prediction. The empirical r obtained from the true model was then compared against this null distribution to determine whether predictive performance exceeded random levels^6^.

### Statistics and reproducibility

The sample size was determined by an a priori power analysis conducted in G*Power 3.1.9.7. Assuming a medium effect size, α = 0.01, and power = 0.80, the minimum required sample size was 34 dyads. Thirty-seven same-gender stranger dyads were initially recruited. One dyad was excluded because of insufficient fNIRS data quality, and two dyads were excluded according to a predefined high-honesty criterion, defined as an honesty rate exceeding 85% across the experiment. The final sample included 34 dyads. Each dyad was treated as one independent biological replicate for dyad-level behavioral, neural, and brain–behavior analyses.

Roles were randomly assigned within each dyad and remained fixed throughout the experiment. Gain and loss contexts were administered within participants using an ABBA block structure, with the initial context counterbalanced across dyads. Unless otherwise specified, analyses were conducted at the dyad level using valid trials after predefined exclusion criteria. No data imputation was performed.

Behavioral data were analyzed using repeated-measures ANOVA, paired-samples t tests, independent-samples *t* tests, or chi-square tests where appropriate. Multiple comparisons were controlled using Bonferroni correction for behavioral analyses and false discovery rate correction for fNIRS activation and INS analyses. HbO responses were estimated using a generalized linear model. Because honesty trials were sparse, primary neural analyses focused on receiver-disadvantageous recommendation trials; honesty-trial analyses were exploratory and were not treated as sufficiently powered deception-versus-honesty contrasts. INS was computed using wavelet transform coherence, and non-parametric pseudo-dyad permutation tests were used to assess whether observed INS effects exceeded chance-level coupling.

Brain–behavior associations were examined using two-tailed Pearson correlations, with robustness assessed using partial correlations, Spearman correlations, bootstrap confidence intervals, robust regression, and leave-one-dyad-out sensitivity analyses. Predictive analyses used leave-one-subject-out or leave-one-out cross-validation, depending on the analysis level. Cross-validation folds were used only to estimate generalization performance and were not treated as independent replicates. Statistical significance of predictive performance was evaluated using label-shuffling permutation tests. All analyses were implemented using standard MATLAB, Python, and R procedures. Exact *p* values, effect sizes, correction methods, and model performance metrics are reported in the main text, tables, and supplementary materials.

### Reporting summary

Further information on research design is available in the Nature Portfolio Reporting Summary linked to this article.

## Supplementary information

Supplementary Information

Reporting Summary

Transparent Peer Review file

## Data Availability

All data in this study have been provided in an Excel file, in accordance with journal requirements. The complete raw data are available to researchers upon approval via the following repository: https://www.scidb.cn/s/IN7NFr^79^.

## Code availability

The custom analysis scripts used for fNIRS preprocessing, interpersonal neural synchrony calculation, statistical analyses, and predictive modeling are available from the corresponding authors upon reasonable request. The code is publicly available on Scidb at https://www.scidb.cn/s/IN7NFr^79^.
