# Supplementary material for: Loss contexts enhance dorsolateral prefrontal interpersonal neural synchrony during successful human deceptive recommendations
Source: Commun Biol. 2026 Jun 30;9:1376. doi: 10.1038/s42003-026-10577-2 (PMC13683375; doi:10.1038/s42003-026-10577-2)
Supplement: Supplementary file 1 [file 42003_2026_10577_MOESM1_ESM.pdf]

Supplementary materials

Supplementary Table 1. Baseline homogeneity tests of demographic variables across gender dyads.

Variable	Male dyads (n=32)	Female dyads (n=36)	$\chi^2_{(1)}/t_{(66)}$	*p*	*φ*/*Cohen*'s *d*
Educational Background (Undergraduate/Graduate)	30/2	28/8	3.446	0.063	0.225
Age (years)	20.56±1.29	21.33±2.07	-1.814	0.074	-0.441
Monthly Disposable Income (RMB)	1237.50±300.27	1216.67±292.28	0.290	0.773	0.070
IOS Score (pretest)	1.09±0.30	1.00±0.00	1.791	0.083	0.462

Only the receiver in each dyad was included in the analysis.

Variable	Male (n=16)	Female (n=18)	$t_{(66)}$	*p*	*Cohen*'s *d*
SVS-Gullibility	1.80±0.54	1.99±0.36	-1.214	0.234	-0.417
SVS-Credulity	1.84±0.56	1.87±0.42	-0.200	0.843	-0.069
HAS-Introspection	1.44±0.39	1.30±0.31	1.138	0.264	0.391
HAS-Reactivity	1.53±0.46	1.36±0.48	1.049	0.302	0.360

Supplementary Table 2. Contextual differences in intra-brain activation (sender) and dyadic ΔINS within the task-relevant time window.

Analysis Level	Channel	Brain Region	Gain Context	Loss Context	$t(33)$	p	*Cohen’s d*
Intra-brain activation during deception trials (sender)	CH1	DLPFC	0.0018±0.0080	0.0034±0.0071	-0.831	0.412	-0.143
	CH2	DLPFC	0.0020±0.0070	0.0034±0.0074	-0.805	0.427	-0.138
	CH3	FPA	0.0029±0.0074	0.0001±0.0070	1.453	0.156	0.249
	CH4	FPA	0.0026±0.0070	0.0033±0.0070	-0.372	0.712	-0.064
	CH5	FPA	0.0022±0.0071	0.0032±0.0078	-0.569	0.573	-0.098
	CH6	DLPFC	0.0010±0.0081	0.0028±0.0071	-1.179	0.247	-0.202
	CH7	DLPFC	0.0016±0.0069	0.0046±0.0060	-1.787	0.083	-0.306
	CH8	VLPFC	0.0011±0.0075	0.0018±0.0072	-0.421	0.676	-0.072
	CH9	DLPFC	0.0026±0.0071	0.0022±0.0074	0.202	0.841	0.035
	CH10	FPA	0.0014±0.0061	0.0037±0.0067	-1.389	0.174	-0.238
	CH11	FPA	0.0023±0.0084	0.0023±0.0070	-0.015	0.988	-0.003
	CH12	FPA	0.0044±0.0076	0.0027±0.0074	1.064	0.295	0.183
	CH13	FPA	0.0009±0.0073	0.0034±0.0074	-1.557	0.129	-0.267
	CH14	DLPFC	0.0004±0.0039	-0.0011±0.0034	2.565	0.015	0.440
	CH15	VLPFC	0.0032±0.0066	0.0030±0.0068	0.126	0.900	0.022
	CH16	VLPFC	0.0027±0.0079	0.0017±0.0062	0.616	0.542	0.106
	CH17	OFC	0.0035±0.0072	0.0052±0.0065	-0.931	0.358	-0.160
	CH18	OFC	0.0012±0.0074	0.0024±0.0072	-0.713	0.481	-0.122
	CH19	OFC	0.0029±0.0063	0.0028±0.0063	0.084	0.934	0.014
	CH20	OFC	0.0048±0.0070	0.0036±0.0057	0.766	0.449	0.131
	CH21	OFC	0.0018±0.0077	0.0048±0.0061	-1.807	0.080	-0.310
	CH22	VLPFC	0.0035±0.0075	0.0029±0.0079	0.343	0.733	0.059
Sender (cheater)-Receiver (detector) ΔINS during successful deception	CH1-CH1	DLPFC	0.2079±0.1365	0.2447±0.1706	-0.881	0.385	-0.151
	CH2-CH2	DLPFC	0.2624±0.1203	0.2117±0.1618	1.496	0.144	0.257
	CH3-CH3	FPA	0.2346±0.1208	0.1881±0.1373	1.465	0.152	0.251
	CH4-CH4	FPA	0.1966±0.0919	0.1750±0.1595	0.679	0.502	0.116
	CH5-CH5	FPA	0.2425±0.1080	0.1909±0.1433	1.558	0.129	0.267
	CH6-CH6	DLPFC	0.2062±0.0923	0.1966±0.1261	0.310	0.758	0.053
	CH7-CH7	DLPFC	0.2072±0.1255	0.1861±0.1191	0.690	0.495	0.118
	CH8-CH8	VLPFC	0.1726±0.1307	0.2276±0.1273	-1.687	0.101	-0.289
	CH9-CH9	DLPFC	0.2857±0.1490	0.2725±0.1518	0.376	0.709	0.065
	CH10-CH10	FPA	0.2499±0.0842	0.2336±0.1096	0.723	0.475	0.124
	CH11-CH11	FPA	0.2023±0.1130	0.1777±0.1149	0.751	0.458	0.129
	CH12-CH12	FPA	0.2263±0.1027	0.1947±0.1515	1.215	0.233	0.208
	CH13-CH13	FPA	0.2786±0.0981	0.2890±0.1078	-0.405	0.688	-0.070
	CH14-CH14	DLPFC	0.2220±0.1166	0.4396±0.1183	-8.656	<0.001	-1.485
	CH15-CH15	VLPFC	0.2008±0.1276	0.1972±0.1134	0.123	.903	0.021
	CH16-CH16	VLPFC	0.1876±0.1786	0.1882±0.1348	-0.015	.989	-0.002
	CH17-CH17	OFC	0.1953±0.1279	0.1637±0.0926	1.143	.261	0.196
	CH18-CH18	OFC	0.1880±0.1018	0.1961±0.1230	-0.295	.770	-0.051

	CH19-CH19	OFC	0.2078±0.1460	0.1671±0.1211	1.143	.261	0.196
	CH20-CH20	OFC	0.1806±0.0965	0.1935±0.1386	-0.431	.670	-0.074
	CH21-CH21	OFC	0.2539±0.1112	0.1947±0.1208	1.924	.063	0.330
	CH22-CH22	VLPFC	0.2452±0.1099	0.2148±0.1156	1.066	.294	0.183

Note: Intra-brain activation values should be multiplied by 10^{-3}.

Supplementary Table 3. Results of hierarchical linear regression model (context).

Context	Step	R^2	Adj. R^2	*F* values	Model sig.	ΔR^2	ΔF	ΔF sig.
Gain	1	0.013	-0.047	0.218	0.806			
	Demographic variables							
	2	0.059	-0.098	0.375	0.861	0.046	0.487	0.694
	Eye contact during successful deception							
	3	0.063	-0.131	0.323	0.920	0.004	0.116	0.736
	Single-brain activation (β)							
	4	0.137	-0.079	0.634	0.724	0.074	2.410	0.132
	INS during failed deception							
	5	0.713	0.627	8.369	<0.001	0.576	54.093	<0.001
	INS during successful deception							
Loss	1	0.166	0.115	3.275	0.051			
	Demographic variables							
	2	0.171	0.093	2.201	0.107	0.005	0.211	0.649
	Eye contact during successful deception							
	3	0.17	0.064	1.600	0.199	<0.001	<0.001	0.995
	Single-brain activation (β)							
	4	0.220	0.090	1.690	0.168	0.049	1.871	0.182
	INS during failed deception							
	5	0.358	0.225	2.696	0.033	0.138	6.248	0.018
	INS during successful deception							

Supplementary Table 4. Validation analysis results of hierarchical linear regression model (context).

Context	Step	R^2	Adj. R^2	F values	Model sig.	ΔR^2	ΔF	ΔF sig.
Gain	1	0.659	0.649	65.804	<0.001	0.659	65.804	<0.001
	INS during successful deception							
	2	0.662	0.642	32.379	<0.001	0.003	0.303	0.586
	INS during failed deception							
	3	0.664	0.633	21.089	<0.001	0.002	0.159	0.693
	Single-brain activation (β)							
	4	0.701	0.663	18.182	<0.001	0.037	3.842	0.059
	Eye contact during successful deception							
	5	0.702	0.640	11.391	<0.001	0.001	0.046	0.955
	Demographic variables							
Loss	1	0.262	0.240	12.059	0.001			
	INS during successful deception							
	2	0.292	0.249	6.793	0.003	0.030	1.390	0.247
	INS during failed deception							
	3	0.292	0.225	4.393	0.011	<0.001	0.002	0.961
	Single-brain activation (β)							
	4	0.301	0.211	3.335	0.022	0.009	0.406	0.529
	Eye contact during successful deception							
	5	0.358	0.225	2.696	0.033	0.057	1.292	0.290
	Demographic variables							

Supplementary Figure 1. Data-driven identification of task-sensitive frequency bands under different contexts.

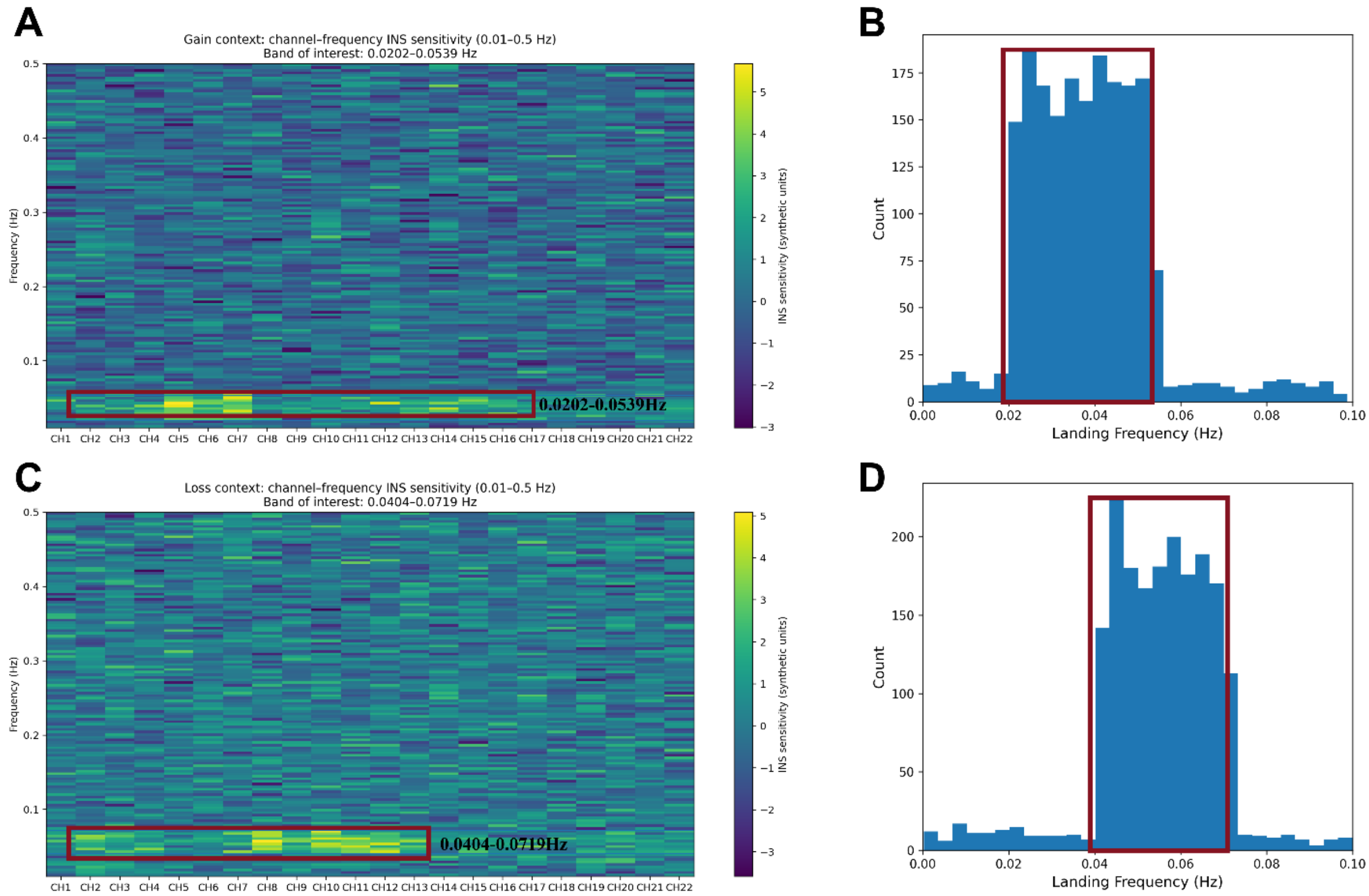

Note: Frequencies correspond to the peak ΔINS value in each trial (n=34 participants×48 trials). The distributions demonstrate consistent convergence within the identified bands, reinforcing that the synchronization effects are frequency-specific rather than driven by slow block-level trends or filtering artifacts.

(A) Data-driven identification of task-sensitive frequency bands in the gain context.

(B) LOOCV distribution within the data-driven frequency band under the gain context.

(C) Data-driven identification of task-sensitive frequency bands in the loss context. Frequency–channel heatmaps of task-related ΔINS were computed across the full 0-0.5Hz range without imposing predefined band-pass constraints during analysis. The horizontal axis represents the 22 fNIRS channels, and the vertical axis denotes frequency (Hz). Significant clusters emerged within restricted frequency ranges (gain: 0.0202-0.0539 Hz; loss: 0.0404-0.0719 Hz), rather than being uniformly distributed across the entire spectrum.

(D) LOOCV distribution within the data-driven frequency band under the loss context.

Supplementary Figure 2. ML performance in predicting deception outcomes based on trial-level ΔINS.

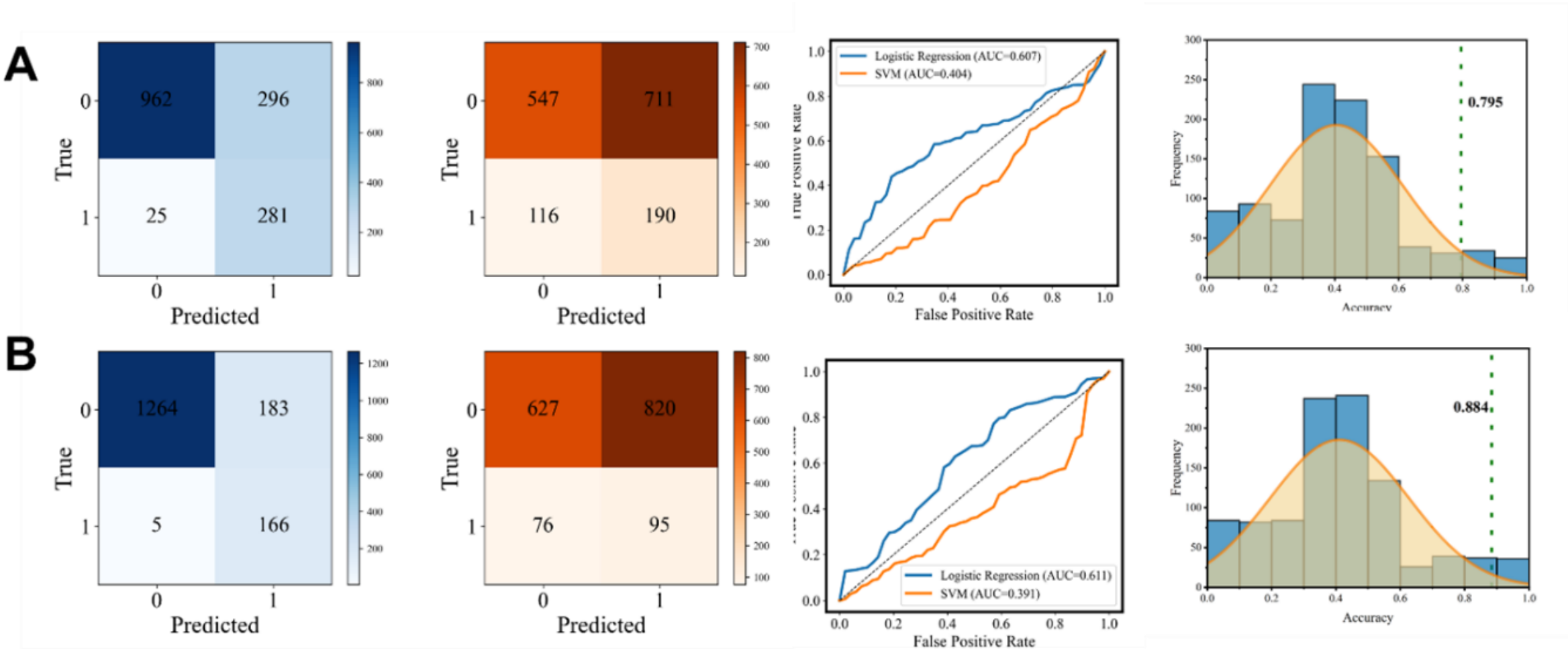

(A) Gain context (n=1564 trials);

(B) Loss context (n=1618 trials).

From left to right: **(1)** Confusion matrix for Logistic Regression (0=successful deception; 1=failed deception); **(2)** Confusion matrix for SVM; **(3)** ROC-AUC curves for both models (training phase), showing superior performance of Logistic Regression across contexts; **(4)** Permutation test results (1,000 iterations) for Logistic Regression, where the green dashed line indicates observed accuracy and the yellow curve represents the fitted null distribution.

Supplementary Figure 3. Comparative predictive performance of ML models across contexts.

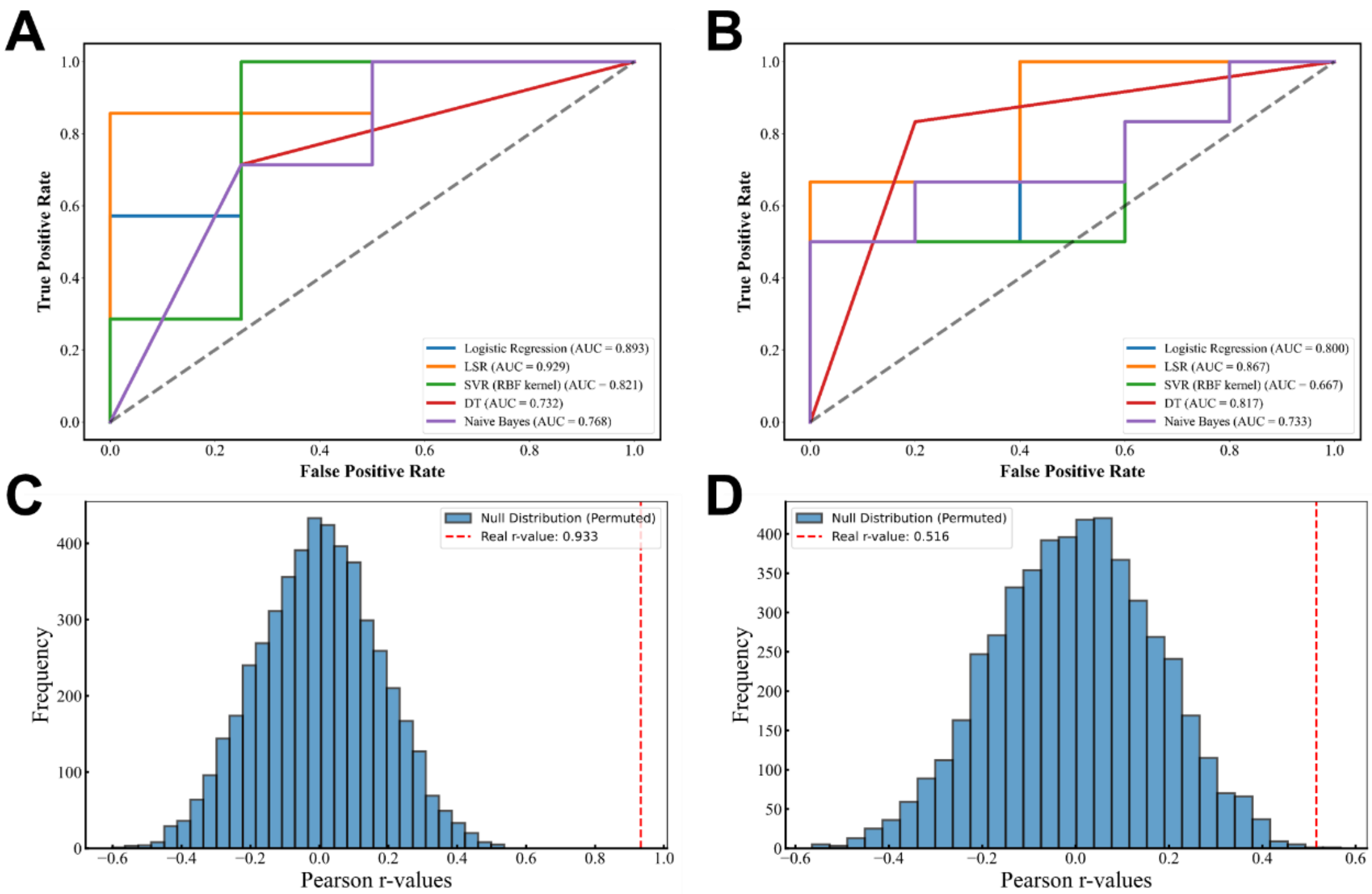

(A) ROC curves comparing ML models in the gain context.
(B) ROC curves comparing ML models in the loss context.
(C) Results of the 5,000-permutation test for predicting successful deception using ΔINS features in the gain context.
(D) Results of the 5,000-permutation test for predicting successful deception using ΔINS features in the loss context.
