# Supplementary material for: Loss contexts enhance dorsolateral prefrontal interpersonal neural synchrony during successful human deceptive recommendations
Source: Commun Biol. 2026 Jun 30;9:1376. doi: 10.1038/s42003-026-10577-2 (PMC13683375; doi:10.1038/s42003-026-10577-2)
Supplement: Supplementary file 2 [file 42003_2026_10577_MOESM2_ESM.pdf]

nature portfolio

Corresponding author(s): Yingjie, Liu

Last updated by author(s): Jun 4, 2026

Reporting Summary

Nature Portfolio wishes to improve the reproducibility of the work that we publish. This form provides structure for consistency and transparency in reporting. For further information on Nature Portfolio policies, see our Editorial Policies and the Editorial Policy Checklist.

Statistics

For all statistical analyses, confirm that the following items are present in the figure legend, table legend, main text, or Methods section.

n/a	Confirmed	
☐	☒	The exact sample size (*n*) for each experimental group/condition, given as a discrete number and unit of measurement
☐	☒	A statement on whether measurements were taken from distinct samples or whether the same sample was measured repeatedly
☐	☒	The statistical test(s) used AND whether they are one- or two-sided<br>*Only common tests should be described solely by name; describe more complex techniques in the Methods section.*
☐	☒	A description of all covariates tested
☐	☒	A description of any assumptions or corrections, such as tests of normality and adjustment for multiple comparisons
☐	☒	A full description of the statistical parameters including central tendency (e.g. means) or other basic estimates (e.g. regression coefficient) AND variation (e.g. standard deviation) or associated estimates of uncertainty (e.g. confidence intervals)
☐	☒	For null hypothesis testing, the test statistic (e.g. *F*, *t*, *r*) with confidence intervals, effect sizes, degrees of freedom and *P* value noted<br>*Give P values as exact values whenever suitable.*
☒	☐	For Bayesian analysis, information on the choice of priors and Markov chain Monte Carlo settings
☐	☒	For hierarchical and complex designs, identification of the appropriate level for tests and full reporting of outcomes
☐	☒	Estimates of effect sizes (e.g. Cohen's *d*, Pearson's *r*), indicating how they were calculated

Our web collection on statistics for biologists contains articles on many of the points above.

Software and code

Policy information about availability of computer code

Data collection

Data acquisition and task implementation were conducted using a combination of commercial hardware systems and custom-developed scripts. Functional near-infrared spectroscopy (fNIRS) signals were collected using the LIGHTNIRS system (Shimadzu Corporation, Japan), operating at wavelengths of 780, 805, and 830 nm with a sampling rate of 13.3 Hz. Optical signals were converted to concentration changes in oxy- and deoxy-hemoglobin based on the modified Beer–Lambert law implemented within the system software.

Experimental task presentation, stimulus timing, and event-marker synchronization were implemented using custom Python scripts developed by the authors (Python 3.9). These scripts controlled stimulus display, behavioral response recording, and trial structure, and were executed on laboratory computers running Windows 10.

No proprietary or undisclosed software was used beyond the commercial acquisition system and standard statistical software described above.

Data analysis

Data analyses were performed using commercial software, open-source libraries, and custom-developed scripts. Behavioral and questionnaire data were analyzed in SPSS (v26.0, IBM Corp.) and MATLAB (R2022a, MathWorks), using paired t-tests, repeated-measures ANOVA, hierarchical regression, and Pearson correlation, with Bonferroni or FDR correction where appropriate.

fNIRS preprocessing and intra-brain analyses were conducted in MATLAB (R2022a) using Homer2 (v2.8) and NIRS-SPM (v4.0). Procedures included channel pruning, motion artifact detection and wavelet correction, principal component analysis, band-pass filtering (0.01–0.5 Hz), and GLM estimation. Interpersonal neural synchrony (INS) was computed via continuous wavelet transform coherence implemented in custom MATLAB scripts. Non-parametric permutation testing (1,000 iterations) was used to validate INS effects.

Machine-learning analyses were conducted in Python (v3.9) using scikit-learn (v1.2.2) and associated libraries. Logistic regression, least-squares regression, decision trees, Naive Bayes, SVM, and SVR were evaluated using leave-one-out cross-validation. Predictive performance was assessed using ROC-AUC, accuracy, F1, MCC, R^2, and permutation testing (up to 5,000 iterations). No undisclosed proprietary analysis software was used.

For manuscripts utilizing custom algorithms or software that are central to the research but not yet described in published literature, software must be made available to editors and reviewers. We strongly encourage code deposition in a community repository (e.g. GitHub). See the Nature Portfolio guidelines for submitting code & software for further information.

Data

Policy information about availability of data

All manuscripts must include a data availability statement. This statement should provide the following information, where applicable:

- Accession codes, unique identifiers, or web links for publicly available datasets
- A description of any restrictions on data availability
- For clinical datasets or third party data, please ensure that the statement adheres to our policy

All data in this study have been provided in an Excel file, in accordance with journal requirements. The complete raw data and analysis scripts are available to researchers upon approval via the following repository: https://www.scidb.cn/s/n6fyqa.

Research involving human participants, their data, or biological material

Policy information about studies with human participants or human data. See also policy information about sex, gender (identity/presentation), and sexual orientation and race, ethnicity and racism.

Reporting on sex and gender

Sex (biological attribute) was recorded for all participants based on self-report at recruitment. Gender identity was not separately assessed. Participants were healthy young adults recruited from a university population. The final sample comprised 34 same-sex stranger dyads (N = 68 individuals), including 18 female dyads (36 individuals assigned female at birth) and 16 male dyads (32 individuals assigned male at birth). Dyads were formed as same-sex pairs to eliminate potential cross-sex interaction effects on interpersonal neural synchrony and deceptive behavior.

Sex was considered during study design and statistical analysis. Baseline comparability between male and female dyads was assessed using independent-samples t-tests and chi-square tests on demographic variables and personality measures relevant to deception susceptibility. No significant sex differences were observed in baseline characteristics or behavioral outcomes; therefore, subsequent primary analyses were conducted collapsing across sex. No interaction effects involving sex were detected in mixed-design analyses.

The study was not designed to test sex-specific hypotheses, and results are not restricted to one sex group. However, as gender identity beyond biological sex was not collected, conclusions are limited to biological sex categories as self-reported. Sex-disaggregated behavioral and neural data are provided in the raw dataset. Data sharing was approved by the institutional ethics committee, and all participants provided written informed consent permitting anonymized data sharing.

Reporting on race, ethnicity, or other socially relevant groupings

Race and ethnicity were not collected and were not included as analytical variables. The study was designed to examine context-dependent deception and interpersonal neural synchrony in controlled dyadic interactions, and no hypotheses concerned racial or ethnic variation. Race or ethnicity was therefore not used as a proxy for socioeconomic or cultural variables.

Participants were recruited from a university population and were native Chinese speakers, confirmed by self-report at recruitment to ensure task comprehension. Demographic variables including educational background and monthly disposable income were obtained via self-report to characterize the sample and were included in baseline comparisons and hierarchical regression models to control for potential socioeconomic confounding.

No race- or ethnicity-specific analyses were conducted. All demographic data were self-reported, and participants provided written informed consent for anonymized data sharing under institutional ethical approval.

Population characteristics

Participants were healthy young adults recruited from a university population. The final sample included 34 same-sex stranger dyads (N = 68 individuals; 36 females, mean age = 21.33 ± 2.07 years; 32 males, mean age = 20.56 ± 1.29 years). All participants were native Chinese speakers, right-handed, and reported normal or corrected-to-normal vision.

Exclusion criteria included prior acquaintance within dyads, excessive fNIRS artifacts (>50% poor-quality channels), and extreme honesty rates (>85%). No participants reported neurological or psychiatric disorders, and no genetic data were collected.

Age, educational background, and monthly disposable income were obtained via self-report and used to characterize the sample. Baseline analyses revealed no significant sex differences in demographic or deception-relevant personality measures. These variables were included as covariates in hierarchical regression analyses to control for potential confounding effects.

Recruitment

Participants were recruited through online advertisements and campus bulletin announcements at a university. Interested individuals contacted the research team voluntarily and were screened for eligibility based on predefined inclusion criteria (healthy young adults, native Chinese speakers, right-handed, normal or corrected-to-normal vision). Dyads were formed from previously unacquainted individuals to ensure stranger-pair interactions. Sex was self-reported during recruitment to facilitate same-sex dyad formation and to control for potential cross-sex interaction effects.

	Because recruitment relied on voluntary participation within a university population, the sample may reflect self-selection bias associated with interest in psychological or neuroscience research. Participants were predominantly young, educated adults, which may limit generalizability to broader age or socioeconomic groups. However, the experimental design focused on mechanistic neural processes under controlled laboratory conditions rather than population-level inference. Recruitment procedures were standardized across participants to minimize systematic selection bias.
Ethics oversight	The study protocol was reviewed and approved by the Medical Ethics Committee of the authors' institution (Approval No. 2023159). All procedures were conducted in accordance with institutional guidelines and the Declaration of Helsinki. Written informed consent was obtained from all participants prior to participation, including consent for anonymized data collection and sharing for research purposes.

Note that full information on the approval of the study protocol must also be provided in the manuscript.

Field-specific reporting

Please select the one below that is the best fit for your research. If you are not sure, read the appropriate sections before making your selection.

☐ Life sciences ☒ Behavioural & social sciences ☐ Ecological, evolutionary & environmental sciences

For a reference copy of the document with all sections, see nature.com/documents/nr-reporting-summary-flat.pdf

Behavioural & social sciences study design

All studies must disclose on these points even when the disclosure is negative.

Study description	This study employed a quantitative, within-subject laboratory experiment to examine context-dependent deception and interpersonal neural synchrony during face-to-face dyadic interaction. Same-sex stranger dyads completed a sender–receiver paradigm under gain and loss framing conditions. Behavioral outcomes (deception rate, successful deception rate, and reaction time) and neurophysiological measures (intra-brain activation and inter-brain synchrony derived from fNIRS hyperscanning) were recorded simultaneously. Contextual framing was experimentally manipulated, and neural–behavioral associations were further evaluated using regression and cross-validated machine-learning analyses. No qualitative data were collected. The design is best characterized as a controlled quantitative dyadic repeated-measures experiment.
Research sample	The research sample consisted of 68 healthy young adults recruited from a university population and organized into 34 same-sex stranger dyads. The final sample included 36 females (mean age = 21.33 years, SD = 2.07) and 32 males (mean age = 20.56 years, SD = 1.29). All participants were native Chinese speakers, right-handed, and reported normal or corrected-to-normal vision. No participants reported neurological or psychiatric disorders.<br><br>The sample was selected to ensure homogeneity in age, language background, and cognitive functioning, thereby reducing variability unrelated to the experimental manipulation. Same-sex stranger dyads were formed to control for cross-sex interaction effects and prior relational familiarity.<br><br>No pre-existing datasets were used; all data were collected prospectively for the present study.
Sampling strategy	Participants were recruited using convenience sampling from a university population through online advertisements and campus postings. Interested individuals voluntarily contacted the research team and were screened for eligibility according to predefined inclusion criteria. Dyads were formed from previously unacquainted individuals and assigned as same-sex pairs to control for relational familiarity and cross-sex interaction effects.<br><br>An a priori power analysis was conducted using G*Power (version 3.1.9.7) to determine the required sample size. Assuming a medium effect size ($f = 0.25$), $\alpha = 0.01$, and power ($1 - \beta$) = 0.80 for paired-samples comparisons, the analysis indicated a minimum of 34 dyads. To account for potential data loss due to fNIRS artifacts and behavioral exclusion criteria, 37 dyads were initially recruited. Following predefined exclusion criteria, the final analyzed sample comprised 34 dyads, meeting the planned statistical power.<br><br>No qualitative data were collected; therefore, considerations of data saturation were not applicable.
Data collection	Data were collected in a controlled laboratory setting using computer-based task presentation and functional near-infrared spectroscopy (fNIRS) hyperscanning. Stimuli were presented on monitors positioned diagonally between dyad members to preserve face-to-face interaction. Behavioral responses (choices and reaction times) were recorded automatically via custom Python scripts. Direct eye contact during verbal interaction was coded from synchronized video recordings following standardized procedures.<br><br>Neurophysiological data were acquired simultaneously from both participants using the LIGHTNIRS system (Shimadzu Corporation, Japan), with optodes positioned over the prefrontal cortex. A baseline recording period preceded task execution. All stimulus timing and event markers were computer-controlled to ensure synchronization between behavioral and neural data streams.<br><br>Only the two participants and trained experimenters were present during data collection. No additional observers were present. Experimenters were aware of the within-subject framing manipulation (gain vs. loss), as task administration required condition control; however, they were not informed of specific hypotheses regarding expected neural or behavioral differences between conditions. Data preprocessing and statistical analyses were conducted according to predefined criteria to minimize potential bias.
Timing	Data collection was conducted between October 2023 and December 2023. Recruitment and testing proceeded continuously during this period without interruption. Participants were tested individually as dyads upon successful eligibility screening, and no separate

sampling cohorts were defined. All experimental sessions followed an identical protocol and equipment configuration throughout the entire data collection window. No procedural modifications were introduced during this period.

Data exclusions

Data exclusions were predefined prior to analysis. A total of 37 dyads were initially recruited. Three dyads were excluded before final analysis: two dyads were removed due to excessive fNIRS signal artifacts (defined a priori as >50% poor-quality channels after preprocessing), and one dyad was excluded due to extreme honesty rates (>85%), which precluded meaningful modeling of deceptive behavior. These criteria were established before statistical analysis to ensure data quality and model validity.

At the trial level, trials containing missing behavioral responses or invalid reaction times (±3 SD from each participant's mean) were excluded according to predefined thresholds. No additional exclusions were applied after inspection of the results. The final analyzed sample comprised 34 dyads (N = 68 participants).

Non-participation

All individuals who met the predefined eligibility criteria and provided written informed consent completed the experimental session. No participants withdrew during data collection, and no eligible individuals declined participation after enrollment. Of the 37 dyads initially recruited and tested, three dyads were excluded based on predefined data-quality and behavioral criteria (as described under Data exclusions), rather than participant withdrawal.

Because recruitment was based on voluntary response to advertisements within a university population, the exact number of individuals who viewed the recruitment materials but did not respond cannot be determined. Among those screened and deemed eligible, the participation rate was 100%, and no attrition occurred during the experimental procedure.

Randomization

The study employed a within-subject design in which all dyads completed both gain and loss framing conditions. Contextual framing (gain vs. loss) was manipulated within participants, and the order of conditions was counterbalanced across dyads to control for potential order effects.

Dyads were formed as same-sex stranger pairs to control for cross-sex interaction and prior familiarity. No random assignment to independent groups was required, as each dyad served as its own control. Potential covariates, including age, educational background, and monthly disposable income, were measured and included in regression analyses where appropriate to account for possible confounding influences.

Reporting for specific materials, systems and methods

We require information from authors about some types of materials, experimental systems and methods used in many studies. Here, indicate whether each material, system or method listed is relevant to your study. If you are not sure if a list item applies to your research, read the appropriate section before selecting a response.

Materials & experimental systems

n/a	Involved in the study
☒	☐ Antibodies
☒	☐ Eukaryotic cell lines
☒	☐ Palaeontology and archaeology
☒	☐ Animals and other organisms
☒	☐ Clinical data
☒	☐ Dual use research of concern
☒	☐ Plants

Methods

n/a	Involved in the study
☒	☐ ChIP-seq
☒	☐ Flow cytometry
☒	☐ MRI-based neuroimaging

Plants

Seed stocks

Report on the source of all seed stocks or other plant material used. If applicable, state the seed stock centre and catalogue number. If plant specimens were collected from the field, describe the collection location, date and sampling procedures.

Novel plant genotypes

Describe the methods by which all novel plant genotypes were produced. This includes those generated by transgenic approaches, gene editing, chemical/radiation-based mutagenesis and hybridization. For transgenic lines, describe the transformation method, the number of independent lines analyzed and the generation upon which experiments were performed. For gene-edited lines, describe the editor used, the endogenous sequence targeted for editing, the targeting guide RNA sequence (if applicable) and how the editor was applied.

Authentication

Describe any authentication procedures for each seed stock used or novel genotype generated. Describe any experiments used to assess the effect of a mutation and, where applicable, how potential secondary effects (e.g. second site T-DNA insertions, mosiacism, off-target gene editing) were examined.
