# Supplementary material for: Loss contexts enhance dorsolateral prefrontal interpersonal neural synchrony during successful human deceptive recommendations
Source: Commun Biol. 2026 Jun 30;9:1376. doi: 10.1038/s42003-026-10577-2 (PMC13683375; doi:10.1038/s42003-026-10577-2)
Supplement: Supplementary file 3 [file 42003_2026_10577_MOESM3_ESM.pdf]

nature portfolio

Peer Review File

Loss Contexts Enhance Dorsolateral Prefrontal Interpersonal Neural Synchrony During Successful Human Deceptive Recommendations

Corresponding Author: Professor Yingjie Liu

This file contains all reviewer reports in order by version, followed by all author rebuttals in order by version.

Version 0:

Reviewer comments:

Reviewer #1

(Remarks to the Author)
SUMMARY:
In this paper, the authors use fNIRS in pairs of people engaging in a series of two-party economic game interactions to study deception. They find that people deceive more when the mutual outcomes are in the loss domain and that there is greater DLPFC synchrony between partners in the gain domain. The topic is important and interesting, and the study approaches the question of how such a complex social interaction might progress.
Unfortunately, the manuscript is hampered in several ways. First, by confusing nomenclature (e.g., the phrases “sender,” “deceiver,” and “cheater” appear to be used interchangeably, though it’s unclear to me whether these are indeed the same), second by unwarranted claims of 1) causality (no causal manipulations are present), 2) morality (there is no assessment of morality during the game), and 3) automaticity (there is no assessment of controlled versus automatic processing), third by statistically biased analyses (they find that neural data which differs by context can predict deception which differs by context, which would follow logically), and fourth by several concerns with the task, analysis approach, analyses, and exclusion criteria. Fifth and finally, it’s unclear to me that the authors can even state that there is deception in this task, given the deeply uncontrolled nature of key portions of the interaction at its heart. While I believe there may be great data here, significantly more analyses would be required and the paper would need to be re-written from top to bottom to address these issues, leading me to recommend rejection.

Concerns/questions/issues, listed chronologically:
- The characterization of the DLPFC as reliably involved in value assessment and risk in a global sense is somewhat misleading. While there are of course papers that find DLPFC, many others do not, and the introduction section covering the role of DLPFC in valuation (lines 59-79) is incomplete in this regard. Some acknowledgment that many studies and meta-analyses of valuation do not identify DLPFC would be important (see: work by Glimcher, Rangel, and others). If I didn’t know better, this section would seem to suggest that this one region, DLPFC, is fully responsible for all valuation and gain-loss asymmetries in decision-making.
- The detailed description of how regression fits data (i.e. by minimizing the error between predicted and observed values; lines 130-135) is an issue. This is true (or effectively true) of all regression approaches and is a very basic conceptual description of all model fitting processes in general, including nonlinear. Thus it’s unclear that there’s anything special at all about the machine learning approach taken here. Indeed, this concern is deepened when the application (later on) appears to be a 30-datapoint regression, with no controls, comparisons, or other details about how it was done or verified.
- The hypothesizes were not effectively motivated. For example, it was unclear why the authors hypothesized that DLPFC activation would be greater in gain contexts. The evidence presented in the introduction indicates DLPFC has a role in evaluation generally – which would still be engaged in loss contexts, of course. Similarly, the prediction of higher synchrony in gain contexts had no support, as far as I could tell.
- Despite the collection of data from the entire PFC throughout the entire task, there’s only ever consideration of DLPFC activity at one moment of the task. The use of other photodiodes’ activity could serve important purposes here, for example as a contrast to the DLPFC results to help the reader understand what is special to the patterns of activity in DLPFC, or even what is special in the moment in the task being analyzed.
- The calculation of power was unclear – the authors simply state that “according to the calculation the required sample size was…”, without stating what the power analysis was conducted on (a correlation? T-test? One-sample? Etc.). A little more

detail here would be helpful as much of the rest of the data seems to reflect paired samples t-tests (with ~30 points) and small correlations (with ~30 points), and their power analysis seems to call for double that number (68 datapoints).
- Many methods details are unclear (e.g., "data from 34 pairs of participants, including 18 female and 16 male participants" I think should be "18 female and 16 male participant pairs"? Similarly, it's unclear to me what the message text sent during the task means, i.e. what "Option A is more suitable for you and is more favorable to you" is about). Double-check throughout to ensure accuracy and completeness.
- The authors write that "we used multiple linear regression to rule out causal relationships between gender and other demographic variables and deceptive behavior," but this is unclear how! This is not a trivial step, nor are any results ever discussed here. The supplementary file was empty, and no further information was provided.
- It was unclear whether the task has any consequences to deception. Is there any way in which the receiver can find out what the actual matrix was (so as to identify the presence of deception), did outcome amounts vary, or was there any way the receiver could respond to the sender to e.g. punish deception or otherwise communicate anything or do anything to the sender? Given that the authors write "The receiver did not know the veracity of the sender's message, and the sender did not know if they had succeeded in deceiving the receiver," it was unclear what risk is present to the sender in this game. They can literally never be found out. It's also the case that neither the sender nor the receiver can do any learning or adjustment if they never find out if they were successful (in deceiving or in choosing the highest value option). I'd recommend the authors discuss what optimal behavior would be here as a way of clarifying incentives and/or the cognitive processes senders and receivers are expected to engage during the task.
- Why was the order of gain and loss blocks fixed as ABBA? This presents a clear confound that should be acknowledged and discussed.
- Were any trials dropped because of failure to keep to the strict timeline outlined in Fig. 1? What happened if someone e.g. took longer than the allowed time to talk or enter a response?
- The text "Option A is more suitable for you and is more favorable to you" was mentioned at one point in the methods, but when the task and setup were described more later, this sentence was not present. The authors should clarify whether this sentence was present in the method or not.
- Were participants instructed as to the nature of the task? What were they told, how was comprehension checked, and to what extent were participants "fully informed" as to the incentives and structure of the task?
- Given the uncontrolled nature of the task (there is a 15s period in which senders can verbally talk with receivers, if I'm understanding right), the use of "deception" here seems potentially problematic. In other words, while the recommendation of one option or the other might be characterized clearly as "sender-advantageous" or "receiver-advantageous", the presence of DECEPTION can only be inferred if we know what the sender said alongside that recommendation. A sender could for example (and quite possibly did!) say that they would alternate recommendations for maximum fairness, entailing zero actual deception. The authors should discuss the limits on their ability to conclusively identify the presence of deception in this task (it is difficult if not impossible to do so) and consider reframing the paper in terms of receiver-advantageous and -disadvantageous recommendations.
- Degrees of freedom must be reported with statistical tests. None are reported.
- The authors state they exclude people who did not "deceive" enough – but then do not state how many pairs were excluded (which might be expected to significantly impact statistical power).
- There were 48 trials of deception in gain contexts and 48 in loss, but there is no behavioral analysis of those trials. Literally the only report is the aggregate number of "deception" trials as a function of gain or loss contexts. This is simply not sufficient from a behavioral analysis perspective for a peer reviewed study. There are no reaction times, no trial-level analyses (for example of how sender's recommendations affected receiver's choices), no examinations of block differences or trends, no consideration of patterns in choices, or individual differences/variation in behavior. Literally a single test is performed – a paired t-test comparing how many trials are "deceptive" in the gain frame and how many in the loss frame.
- The brain area identified as DLPFC in Figure 3 I think would be widely characterized as frontopolar or anterior prefrontal cortex, but I would confidently say that it is certainly not dorsal OR lateral.
- Given the presence of significant movement (participants are simply seated and talking with each other in an unrestrained setting), could the authors comment on how much of the fNIRS signal might be related to motion or motor movement, either at the gross level (whole body shifting) or at the local level (facial expressions)?
- If I understand the LSR ML analysis, the analysis was carried out at the participant, not trial, level? That would mean that the authors asked if roughly 30 brain synchrony values could predict roughly 30 deception quantities. This is problematic in several respects. Figure 3 already demonstrates an average difference between gain and loss contexts; thus it follows that one could take that brain activity and turn around ask whether it predicts deception (it would, as deception varies across gain and loss contexts), making the analysis circular. The authors have a lot of trials (96 for each pair) which they should consider using in a nested fashion (across participants) to make a much stronger and more interesting trial-wise prediction.
- Use of a bandpass filter when the data have an extremely low frequency component (the ABBA order of gain/loss contexts across blocks) might have removed important signals. As the authors state the whole study was 45 minutes, an ABBA signal spread over that time could be considered a slowly moving signal with a half-period of 45 minutes, and thus a period of 1.5 hours. That'd correspond to a signal with a frequency of ~ 0.00019 Hz, clearly removed by a bandpass filter with cutoffs of 0.01 Hz and 0.5 Hz, line 278). Furthermore, if sender or receiver behavior exhibited any e.g. linear trends over time, such a bandpass filter would logically also remove fNIRS signals that tracked those slow behavioral shifts. Reconsider use of this filter and/or be sure that this filter isn't removing signals of interest, including slow components at the block and study level.

Reviewer #2

(Remarks to the Author)
In this manuscript, the authors report a study using fNIRS to measure interbrain synchrony while two participants engage in a task designed to prompt deceptive behaviour. One participant was assigned a role in which they were given two potential

outcome matrices, one leading to a better payoff for themselves, the other to a better payoff for their co-player. They then had to persuade the co-player to choose one of the two matrices.
I found it difficult to evaluate this manuscript, as key aspects are unclear.
- What is the research question to be addressed here?
- How many participants were tested, 68 or 34? The manuscript is inconsistent on this.
- Were participants engaging with a real co-player or a confederate?
- Why was the behaviour of the co-player not analysed? Currently, it's not clear how the deception task was introduced to them, and in how far the deception was successful or detected.
- What time window was included in the fNIRS analysis? Did this include time windows in which participants moved or looked at each other?
Please see more detailed comments below.

Abstract: the abstract is rather difficult to understand without having read the paper. A number of terms (e.g. gain-loss contexts; deception) are introduced without conceptualization, and the research methods aren't described. This makes it difficult to understand what the summarized results mean.

Introduction: the prevalence and relevance of deception needs to be explained more. The authors seem to conflate deception and lying, but these are not the same thing. The step from understanding the psychology of deception to stabilizing markets and societal norms is unclear. This seems an overstated impact aim.
Overall, the research question addressed here is not clearly stated. What is the specific question the authors are hoping to answer? The authors provide 3 aims (a,b,c) but it is not clear how a) and c) are distinct from each other. They then provide another 3 aims (1,2,3), again, it is not clear how these are distinct from a,b,c, and which ones are the main focus.

There is an imbalance in the introduction, with key concepts going unexplained and a lack of more detailed discussion of relevant existing findings, but several pages committed to discussing the advantages of machine learning over traditional analysis approaches. Given this is not a methodological paper, the latter seems unnecessary.
Throughout the introduction, the literature discussion remains rather vague and many stated claims or assumptions lack clarity:

'Two contexts commonly occur in economic interactions: partners jointly undertake a project and distribute the profits, or they encounter bankruptcy and share the debts.' - Surely there are a number of alternative – and more common - outcomes (e.g. a project is successful, but the profits passed on to shareholders; a project is unsuccessful, but does not lead to bankruptcy)?

It's not clear from an economy point of view in how far the distinction between a gain-context and loss-context is valid. In most business decisions, both are involved and need to be considered simultaneously, i.e. one risks making a loss in order to have the chance of making a profit.

'Importantly, deception is inherently a risky decision (Grolleau et al., 2016). Previous research has revealed that individuals are more prone to participate in dishonest behavior with the aim of evading losses [..]' I don't follow this argument. The work by Grolleau et al. is not described further, but presumably deception being 'risky' refers to it potentially leading to negative outcomes, rather than it being used in risky situations?

'existing findings remain largely limited to behavioral observations' – why is this a problem if one is interested in understanding behaviour?

'are inconclusive regarding how gain-loss framing moderates the outcomes of deceptive behaviors' this hasn't been mentioned before and needs more explanation. Is the suggestion here that deception is more or less likely to be successful in a risk context? Why would this be the case?

'The DLPFC and parietal cortex are activated when decisions regarding risk are made (Mohr et al., 2010). For instance, researchers have discovered that when participants are confronted with a gambling task with equal probabilities of gaining or losing money, the activation of BOLD response in the prefrontal cortex (PFC) increase as potential rewards rise' I don't follow this argument. Previously the authors argue that risk and reward contexts are distinct, here they appear to equate them.

'Modulation of the right DLPFC through transcranial direct current stimulation (tDCS) abolishes the framing effect in gain-loss decision-making' – the framing effect should be introduced in more detail first.

Methods: The power calculation seems incorrect, or needs to be clarified. A required sample of 68 is stated, and 35 pairs were tested. Given that hyperscanning provides a single dataset for each pair of participants, the sample appears to be half the required size. Further down it says data of 34 participants was analysed, but this should be 68, if 34 pairs were included?

'two experimenters of the same gender were randomly formed into pairs as sender and receiver, respectively' – I don't follow, what was the role of experimenters in the pairs? Was one of the alleged participants a confederate?

'Two participants were seated face to face in an oblique diagonal orientation, with a face computer blocking their view' – this needs clarification. If the participants could not see each other due to a screen blocking their view, how is this task still face to face? Which cues were participants expected to use to detect deception, voice only?

Were both participants aware of the nature of the payoff matrices, i.e. the fact that a better outcome for the receiver always meant a worse outcome for the sender? If so, this would likely prompt receivers to not believe information provided by the sender. If not, did the receiver have any reason to anticipate deception?

Why were dyads with high honesty rates excluded from analysis of behaviour? This would skew the results.

'the percentage of all dyads that were deceived by the sender' – do the authors mean of all trials?

Some of the results are reported in the methods section, this should be corrected.

Results:
Figure 2: this is a bar graph, not a histogram as stated. A histogram would be more informative.

The fNIRS data analysis mostly ignores the main variable of interest, deception vs. honest reporting. Why is this not analysed on a trials-wise basis? Simply comparing gain vs. loss contexts while ignoring trial outcomes means it is impossible to say whether differences in neural activity patterns are due to gain vs. loss contexts, or differences in deception rates between the conditions.

For the behavioural data, behaviour of the recipients needs to be analysed. Did receivers generally believe the information they were given?

This should also be accounted for in the interbrain synchronisation analysis: was synchrony highest in trials where no deception was included, and the receiver believed the information they were given?

Sincerely,
Frederike Beyer

Reviewer #3

(Remarks to the Author)
This study uses dyadic hyperscanning to investigate how gain-loss contexts influence deception and the associated neural mechanisms. The authors found that senders were more likely to deceive in loss contexts, suggesting that the prospect of loss reduces moral restraint and makes deception more automatic. Neurally, senders exhibited increased dorsolateral prefrontal cortex (DLPFC) activation during conflict scenarios in gain contexts, indicating greater cognitive control and moral deliberation when both parties could benefit. During sender–receiver interactions in gain contexts, interpersonal neural synchrony (INS) in the DLPFC increased; conversely, reduced INS in loss contexts was associated with higher deception rates. The study also showed that INS could predict deception rates in loss contexts using least squares regression, highlighting the potential of neural markers to forecast dishonest behaviour.

Overall, the paper provides important insights into how context shapes behavioural and neural aspects of deception. While innovative in combining behavioural, neural and predictive analyses in an interesting dyadic, face-to-face environment, the manuscript could be improved before publication in Communications Biology by addressing the lack of methodological detail and improving the clarity and structure of certain sections.

Abstract
While I found the paper to be generally concise and well-written with a clear structure, I believe the abstract could be improved in terms of clarity and structure. For example, the type of hyperscanning performed is not specified (EEG, NIRS or fMRT?), and several terms are not clearly defined (e.g. interpersonal neural synchrony; what does deception mean in this context?). Furthermore, while some sections provide methodological details (e.g. least squares regression), others lack key information (e.g. sample size).

Power analysis
How do the authors justify their assumption of an effect size of f = 0.25? I also wondered what the final sample size analysed was. It states that 34 pairs of participants were analysed. However, it is later stated that dyads with deception rates greater than 85% were excluded. How many participants were excluded and for which reasons?

Subjective ratings
Were any ratings collected on impressions of the interaction partners (e.g. interpersonal closeness or sympathy) or on subjective states (e.g. arousal)? These ratings could corroborate the authors' interpretations of their findings, for example by showing that decisions about gain contexts are associated with higher levels of moral conflict.

Behavioural paradigm
Several points regarding the behavioural paradigm remained unclear to me after reading the manuscript. Did participants play both the role of sender and receiver? Furthermore, I was confused by the terms 'honesty rate' and 'deception rate'. How exactly were these variables computed? The terms sound as if one variable is the opposite of the other.

fNRIS analysis
While I am not an expert in fNRIS analysis, I wondered if information on the temporal dynamics of brain activation was

available, and if the authors could illustrate this?

Relationship between deception and INS
The authors report a significant negative correlation in the gain context, but not in the loss context. They could report the correlation in the loss context and then statistically compare these correlations to support the idea that the relationship is specific to the gain context.

Machine learning algorithm
Again, I am not an expert in applying machine learning algorithms. However, I wondered what constitutes an increase in knowledge when applying this algorithm? Is it merely to demonstrate a relationship between behaviour and brain activity? This has already been demonstrated by the correlation, hasn't it? Perhaps the authors could explain this in more detail. Moreover, given that the sample size is quite small, I am concerned about the validity of these findings, as it is not possible to separate the test and validation samples.

Response times
As the authors assume that deception in loss contexts might be associated with more automated cognitive processing, I wondered if they had analysed response times. This would support their assumption if they showed that response times were shorter for deception decisions in loss contexts.

Discussion
Overall, I found that the structure of the discussion could be improved by shortening or combining some paragraphs. Adding some transitions and summary sentences would help to clarify to the reader what each paragraph is about.

Version 1:

Reviewer comments:

Reviewer #2

(Remarks to the Author)
The revised version of this manuscript differs significantly from the previous submission, with an almost re-written introduction and significant changes to the analysis strategy. Therefore, this review is essentially a new review of the manuscript, rather than just checking previous concerns have been addressed.

There are significant improvements to the theoretical background, the formulation of hypotheses, and the analysis approaches. However, some original concerns remain and the newly added methodological details & results raise new, substantial concerns.

Firstly, P10: ‘Final payment was calculated as 15RMB+Rgain-Rloss, yielding a total compensation range of 0-45RMB.’ – so participants could end up with not being reimbursed for their time? This would be a highly unethical practice. In itself, performance-based rewards being up to 2x the base payment is ethically questionable.

P9: ‘two dyads were excluded based on a predefined high-honesty criterion (deception rate<85%)’ – is this an error, i.e. did the authors mean an HONESTY rate of >85%? Otherwise, if to be included in analysis, dyads had to show deception on 85% of trials, this contradicts the author’s claim of deception being a cognitively demanding, deliberate, goal-oriented decision-making process; or at least it wouldn’t be in the context of the task used. Deception would be the default response, and Honesty a rare occurrence either done by mistake or to introduce some variability.

Deception rates are extremely high (>90%). This is likely linked to the payment structure, i.e. one trial being selected randomly for payoff. In a directly relevant setting, where every decision affects final payoff, participants can be expected to make trial-wise decisions, for example aiming for equity by sometimes recommending a choice disadvantageous to themselves. If only one trial is counted for the payoff, the rational thing to do is to determine the best strategy on the first trial and stick with that strategy throughout the task. This is particularly relevant given no feedback was provided, such that participants had no opportunities to learn about their partner’s behaviour. As such, the deceiver’s behaviour likely is not reflecting a trial-wise decision-making process as to whether to deceive or not.

P18: ‘Because the primary objective of the present study was to characterize neural response patterns associated with deceptive interaction, fNIRS analyses were restricted to trials in which senders chose to engage in deception.’ This was raised in my original review and has not been sufficiently addressed. To draw any conclusions regarding deception, all analyses must be based on the deception>honesty comparison. Without this, all the authors can say is how brain activity differs between gain vs. loss contexts, but this may be true for any decision-making, involving deception or not. Of course, the extremely low honesty rate in the given paradigm would make such an analysis difficult. This, however, reflects the limiting nature of the task used, as it does not allow for testing the neural basis of deception (given deception is essentially present on every trial).

The authors now include effects for analyses focusing exclusively on honest trials. However, it is unclear if they had enough

trials to run this analysis (presumably, with ~5-10 trials per dyad & condition, this would have been very noisy data), and it does not adequately support the claims made as to the specificity of the effects for deception trials. Without using the correct difference score (deception > honesty) as the basis for the gain > loss comparison, it is not valid to conclude that effects are specific to deception trials only.

P36, Brain-Behaviour Correlation: the observed correlation between inter-brain connectivity in successful deception gain trials and successful deception rate is suspiciously high, showing an almost perfect (negative) relationship. This suggests it may be driven by methodological confounds, e.g. number of trials included in the analysis, length of verbal statements made, trial-to-trial variability in statements made, etc..

Reviewer #3

(Remarks to the Author)
In my opinion, the authors have adequately and thoroughly addressed most of my concerns. Overall, however, I noticed a few minor typographical errors in the revised manuscript; it would be advisable to proofread the text once more.

Regarding my point 6, the authors could consider adding a statistical comparison of the two regression coefficients, which is relatively straightforward to conduct (e.g., using appropriate R packages or the procedure described here:
https://www.psychometrica.de/correlation.html
).

From my side, after these minor revisions, I would not recommend another round of review.

Version 2:

Reviewer comments:

Reviewer #2

(Remarks to the Author)
In their revisions, the authors have addressed my concerns raised in the previous review. Their interpretation of findings is now more in line with the presented data. It should be noted that this somewhat reframes the implications of this research, as conclusions can be drawn about differences between gain-loss contexts, but little can be said about any specificity to the social context of deception. I have some minor comments below, for the authors' consideration.

on page 27, there is a referencing error ('Performance metrics for all conditions and 679
models are summarized in Table 6 and Error! Reference source not found.')

In terms of discussing the results, a major limitation is that speech recordings are not available. As such, much of the interpretation of the fNIRS & hyperscanning results remains speculative (e.g. did dyads with high deception rates have a consistent strategy; are there (m)any cases of logged deception actually based on honest communication, e.g. 'I will recommend the better choice for myself now and you select that, next trial we'll switch'). This is acknowledged in the limitations section, but combined with the above limitations of the task design, some statements such as 'deception is not a fixed individual trait' could be phrased more cautiously.

It may also be useful to check whether the discussion section can be written somewhat more concisely.

Open Access This Peer Review File is licensed under a Creative Commons Attribution 4.0 International License, which permits use, sharing, adaptation, distribution and reproduction in any medium or format, as long as you give appropriate credit to the original author(s) and the source, provide a link to the Creative Commons license, and indicate if changes were made.
In cases where reviewers are anonymous, credit should be given to 'Anonymous Referee' and the source.
The images or other third party material in this Peer Review File are included in the article's Creative Commons license, unless indicated otherwise in a credit line to the material. If material is not included in the article's Creative Commons license and your intended use is not permitted by statutory regulation or exceeds the permitted use, you will need to obtain permission directly from the copyright holder.
To view a copy of this license, visit https://creativecommons.org/licenses/by/4.0/

COMMSBIO-25-4921AR1

Interpersonal Neural Synchrony in Deceptive Behavior Regulated by Gain- Loss Contexts: A fNIRS-based Hyperscanning Study

Dear Editor and Reviewers,

We sincerely appreciate the opportunity to resubmit our revised manuscript. We are extremely grateful for the detailed comments and suggestions provided by Reviewer #1, #2 and #3. These comments have been invaluable in refining and improving our work. The insights not only enhanced the manuscript but also provided crucial guidance for our research. We have carefully considered each reviewer's feedback and have made targeted revisions to further refine our study. We hope that these amendments meet the expectations of both the reviewers and *Communications Biology*. It is with earnest desire that we hope our manuscript will be accepted for publication in *Communications Biology*.

Please find the following Response to the comments of reviewers.

We sincerely appreciate your suggestion of our manuscript and eagerly await feedback from the reviewers. If you have any questions, please do not hesitate to contact me at the address provided.

Sincerely.

Yaozhong Liu, Co-first authors

E-mail: liuyaozhong518@163.com.

Rui Huang, Co-first authors

E-mail: psyhuangrui@163.com.

He Wang, corresponding authors

E-mail: wanghe429@163.com.

Yingjie Liu, Corresponding author

E-mail: psyliuyingjie@163.com.

Reviewer #1:

SUMMARY: *In this paper, the authors use fNIRS in pairs of people engaging in a series of two-party economic game interactions to study deception. They find that people deceive more when the mutual outcomes are in the loss domain and that there is greater DLPFC synchrony between partners in the gain domain. The topic is important and interesting, and the study approaches the question of how such a complex social interaction might progress.*

Unfortunately, the manuscript is hampered in several ways. First, by confusing nomenclature (e.g., the phrases "sender," "deceiver," and "cheater" appear to be used interchangeably, though it's unclear to me whether these are indeed the same), second by unwarranted claims of 1) causality (no causal manipulations are present), 2) morality (there is no assessment of morality during the game), and 3) automaticity (there is no assessment of controlled versus automatic processing), third by statistically biased analyses (they find that neural data which differs by context can predict deception which differs by context, which would follow logically), and fourth by several concerns with the task, analysis approach, analyses, and exclusion criteria. Fifth and finally, it's unclear to me that the authors can even state that there is deception in this task, given the deeply uncontrolled nature of key portions of the interaction at its heart. While I believe there may be great data here, significantly more analyses would be required and the paper would need to be re-written from top to bottom to address these issues, leading me to recommend rejection.

Re: We sincerely thank the reviewer for the thorough and thoughtful evaluation of our manuscript. We have carefully considered the substantive concerns raised and have undertaken a comprehensive revision of the manuscript, including conceptual clarification, methodological expansion, and additional analyses. In response to the issues outlined in the Summary, we have substantially rewritten the manuscript to improve conceptual precision, analytical transparency, and inferential restraint. Below we summarize the major revisions implemented to address each of the reviewer's central concerns.

- **Elimination of Terminological Inconsistency and Conceptual Overextension.**

(1) Standardization of role terminology and task description. To address the concern regarding confusing nomenclature, we systematically reviewed and unified the terminology used to describe experimental roles. The terms "sender," "cheater," and "deceiver" are no longer used interchangeably. A consistent naming convention has been adopted throughout the manuscript to prevent conceptual ambiguity. In addition, the functional meaning of the "recommendation cue (color) + verbal statement" has been operationally defined in a consistent and transparent manner across sections.

(2) Reformulation of the operational definition of deception. We acknowledge that, given the presence of spontaneous verbal expression and the absence of verifiable semantic content, it is not possible to draw definitive conclusions regarding subjective deceptive intent at the single-trial level. Accordingly, we have revised our definition of “deception” to refer strictly to an objectively disadvantageous recommendation to the receiver under a structure of incentive conflict. In this sense, deception is conceptualized as structural information manipulation rather than as an inference about internal intention. The Discussion section now explicitly delineates the scope and limitations of this terminology.

- **Removal of Inappropriate Claims of Causality, Morality, and Automaticity.**

(1) Revision of language implying causality. We have carefully withdrawn or reformulated expressions that could be interpreted as implying causal inference. The present study is observational and correlational in design; no causal manipulation was implemented. Regression and predictive analyses are now explicitly framed as tools for association testing and incremental predictive explanation rather than for establishing or ruling out causal relationships.

(2) Removal of unsupported moral interpretations. Because the task did not include direct measurement of moral judgment or moral evaluation, we have deleted or strictly constrained any language suggesting moral interpretation. All interpretations are now limited to constructs directly supported by behavioral and neural data.

- **Clarification of Statistical Bias Concerns and Potential Circularity in Predictive Analyses**

(1) Expansion of behavioral analyses beyond a single paired-sample comparison. To avoid reliance on a single paired-sample t-test of deception rates, we expanded the behavioral analyses. In addition to deception frequency, we now systematically report reaction times for both sender and receiver, recommendation acceptance (successful deception rate), and key interaction effects. These additional behavioral indicators provide dynamic and interactive evidence that strengthens the robustness of the behavioral conclusions.

(2) Transparency of regression and power analysis structure. We have clarified the statistical structure underlying the power analysis, specifying dyad-level independence in paired comparisons. Degrees of freedom and full reporting standards have been provided for all statistical tests.

(3) Strengthening the predictive framework to address potential logical circularity. In response to the concern that neural differences across contexts might trivially predict behavioral differences across contexts, we have upgraded the predictive framework from a single regression description to a cross-validated approach incorporating multi-model comparison and permutation testing. Furthermore, we implemented trial-level nested prediction analyses under cross-validation schemes that preserve participant independence. This procedure reduces the risk of superficial predictability driven solely by condition-level mean differences and enables fuller utilization of trial-level variability.

■ **Clarification of Whether the Task Involves Genuine Deception, Risk, or Learning**

(1) Clarification of task consequences and incentive implementation. We have explicitly clarified that gain and loss outcomes carried real monetary consequences and were implemented through random trial selection for payment. At the institutional level, both parties were fully informed of the payoff structure and incentive scheme. However, at the trial level, informational asymmetry was preserved. This distinction clarifies how strategic information manipulation could occur within a formally transparent incentive framework.

(2) Rationale for the absence of punishment and feedback mechanisms. We explicitly state that the absence of punishment, reputation tracking, and feedback learning mechanisms was intentional. The design was constructed to isolate immediate decision trade-offs and structural information manipulation in the absence of external deterrence. We now clearly acknowledge in the Discussion that this design limits generalization to contexts involving punishment, reputation, or iterative learning, and we identify such extensions as directions for future research.

(3) Clarification of optimal behavior and the source of risk. We clarify that risk in this paradigm does not arise from social exposure or the possibility of being "caught," but rather from misestimation of the receiver's probability of acceptance, which generates variability in expected payoff. We further provide a clearer theoretical articulation of the decision logic faced by both sender and receiver under this informational structure.

■ **Completion and Strengthening of Methodological Reporting**

(1) Correction and full disclosure of sample and exclusion criteria. We now explicitly report the number of excluded dyads and the specific reasons for exclusion. We clarify consistency between exclusions and the pre-registered power analysis assumptions in order to reduce concerns about selective exclusion and power inflation.

(2) Clarification of task timeline deviations and trial termination rules. We specify the handling procedures for timeout trials, anomalous verbal behavior, and premature trial termination. The number of excluded trials is now reported, and we explicitly state whether exclusions differed by condition.

(3) Increased rigor in neural localization and visualization. We provide additional detail regarding channel localization procedures (3D digitizer acquisition followed by MNI registration). We further clarify that surface projection visualizations reflect interpolation properties and do not correspond to discrete cortical voxels or exact anatomical boundaries. This clarification prevents intuitive misinterpretation of rendered regions (e.g., frontopolar versus dorsolateral prefrontal areas).

(4) Direct response to concerns regarding motion artifacts and filtering. We supplement the manuscript with detailed motion control and preprocessing procedures. Through condition-specific pattern analyses, frequency-band identification, and robustness checks, we demonstrate that the key findings are unlikely to be driven by slow drift or global motion synchrony. At the same time, we explicitly acknowledge residual limitations inherent in naturalistic interaction paradigms and outline future methodological improvements.

In summary, we agree with the reviewer's observation that the original manuscript contained issues in terminology, inferential boundaries, statistical logic, and task explanation that affected clarity and evaluability. We have therefore comprehensively rewritten and supplemented the manuscript so that all conclusions are more strictly anchored to empirically supportable claims and methodological transparency has been substantially enhanced.

For the exact locations of each modification, added analysis, and revised results presentation, please refer to the detailed point-by-point responses that follow.

---

(1) The characterization of the DLPFC as reliably involved in value assessment and risk in a global sense is somewhat misleading. While there are of course papers that find DLPFC, many others do not, and the introduction section covering the role of DLPFC in valuation (lines 59-79) is incomplete in this regard. Some acknowledgment that many studies and meta-analyses of valuation do not identify DLPFC would be important (see: work by Glimcher, Rangel, and others). If I didn't know better, this section would seem to suggest that this one region, DLPFC, is fully responsible for all valuation and gain-loss asymmetries in decision-making.

Re: We sincerely thank the reviewer for this comment. Our original wording may have overemphasized the role of the DLPFC in valuation processes and insufficiently acknowledged the broader neuroeconomic literature demonstrating that subjective value representations are primarily encoded in ventromedial prefrontal and striatal regions. To address this concern, we have substantially revised the Introduction. Specifically, We now explicitly state that deception-related decision-making relies on a distributed valuation and regulatory network, rather than a single region **(see revised manuscript on page 4, lines 62 to 64)**: "Existing neurobiological evidence indicates that deception does not depend on a single brain region, but instead relies on a distributed network supporting valuation and decision-making processes (Liu et al., 2019)."

We have incorporated foundational neuroeconomic findings to clarify the neural basis of subjective value representation **(see revised manuscript on page 4, lines 64 to 68)**: "Neuroeconomic studies and related meta-analyses have shown that core subjective value representations are primarily encoded in the ventromedial prefrontal cortex and the striatum (Glimcher, 2022; Todd et al., 2009), whereas the anterior cingulate cortex and the insula are more highly involved in processing uncertainty, conflict, and affective signals (Medford & Critchley, 2010)." Importantly, we have redefined the role of the DLPFC as context-dependent and regulatory rather than as a primary valuation hub **(see revised manuscript on page 5, lines 77 to 80)**: "However, DLPFC activity is not consistently observed across all decision-making tasks involving value evaluation, suggesting that its functional contribution is

contingent on context-specific demands for cognitive control and strategic monitoring (Hutcherson & Tusche, 2022)." Accordingly, in the revised manuscript, the DLPFC is conceptualized as a control-related region that modulates value-guided behavior under conditions requiring strategic regulation, particularly in gain contexts where fine-grained comparison and inhibition of automatic responses are necessary. We no longer characterize the DLPFC as a global or primary locus of value encoding.

We believe these revisions more accurately reflect the consensus in the neuroeconomic literature and clarify the theoretical positioning of the DLPFC in our framework.

Reference

[1] Liu, P., Shen, H., & Ji, S. (2019). Functional Connectivity Pattern Analysis Underlying Neural Oscillation Synchronization during Deception. *Neural Plasticity*, *2019*, 1-10.

[2] Medford, N., & Critchley, H. (2010). Conjoint activity of anterior insular and anterior cingulate cortex: awareness and response. *Brain Structure & Function*, *214*(5-6), 535-549.

[3] Glimcher, P. W. (2022). Efficiently irrational: deciphering the riddle of human choice. *Trends in cognitive sciences*, *26*(8), 669-687.

[4] Todd, A. H., Colin. F. C., & Antonio, R. (2009).Self-Control in Decision-Making Involves Modulation of the vmPFC Valuation System. *Science*, *324*(5927), 646-648.

[5] Hutcherson, C., & Tusche, A. (2022). Evidence accumulation, not 'self-control', explains dorsolateral prefrontal activation during normative choice. *eLife*, *11*, e65661.

---

(2) The detailed description of how regression fits data (i.e. by minimizing the error between predicted and observed values; lines 130-135) is an issue. This is true (or effectively true) of all regression approaches and is a very basic conceptual description of all model fitting processes in general, including nonlinear. Thus it's unclear that there's anything special at all about the machine learning approach taken here. Indeed, this concern is deepened when the application (later on) appears to be a 30-datapoint regression, with no controls, comparisons, or other details about how it was done or verified.

Re: We thank the reviewer for carefully examining our description of the regression framework. We agree that describing regression as minimizing the error between predicted and observed values reflects a general principle shared by virtually all model-fitting approaches, including both linear and nonlinear methods. We acknowledge that our original wording (lines 130–135) may have given the impression that this principle constituted a methodological contribution. In the revised manuscript, we have therefore removed this overly generic description and explicitly clarified that our study does not claim novelty at the level of optimization principles.

More importantly, we have revised the manuscript to clarify that the contribution of the machine learning analysis does not lie in a single regression model, but rather in a prediction-oriented, cross-validated comparison across multiple algorithms. In the revised analysis, we implemented a leave-one-out cross-validation (LOOCV) framework and systematically compared several commonly used models, including logistic regression, ordinary least squares regression, decision trees, naïve Bayes, and support vector regression. This strategy allows us to assess whether the observed brain–behavior relationship is robust across modeling assumptions rather than driven by a specific algorithmic choice.

Within this framework, INS was treated as the feature variable, and deception rate served as the prediction target. Model performance was evaluated based on out-of-sample predictions generated under LOOCV, thereby directly testing the generalizability of the neural–behavioral relationship. This cross-validated design addresses the concern that the analysis might reflect a simple 30-datapoint regression without verification or comparison.

We have substantially revised the Introduction to reflect this modeling strategy more clearly, explicitly distinguishing our approach from traditional descriptive regression analyses and emphasizing that the primary goal is to evaluate the predictive capacity and robustness of neural signals in explaining deception behavior. **These revisions appear in the revised Introduction (page 7, line132 to page 8, line 150, marked in blue).**

However, while conventional correlational (Fu et al., 2025) or regression-based analyses (Helmer et al., 2024) are effective for identifying brain-behavior associations within specific contexts, they are limited in evaluating whether such relationships generalize across contexts or remain robust under different modeling assumptions. Addressing these questions requires analytical approaches that move beyond descriptive fitting toward prediction-oriented validation (Zhuang et al., 2020). Machine-learning (ML) methods provide a suitable framework for this purpose by treating neural measures as feature variables and behavioral outcomes as prediction targets, thereby enabling direct tests of whether brain-based signals carry reliable predictive information (Shamout et al., 2020; Wang et al., 2020). Importantly, robustness of prediction should not depend on a single algorithm but should be evaluated across multiple models with different inductive biases (Huang et al., 2025). Accordingly, the present study adopts a cross-validated modeling framework in which several commonly used algorithms—including Logistic Regression, least-squares regression (LSR), Decision Trees (DT), Naive Bayes, and Support Vector Regression (SVR)— are systematically compared. Within this framework, ΔINS are used to predict individual differences in cheaters' successful deception rates under gain-loss contexts. Model performance is evaluated using leave-one-out cross-validation, allowing each participant's deception rate to be predicted from models trained on independent data. By emphasizing cross-validated prediction and model comparison, this approach enables us to assess the generalizability and robustness of the neural–behavioral relationship, rather than relying on parameter estimates from a single regression model.

Regarding the relatively modest sample size (N = 34), we acknowledge that this limits the feasibility of conventional training–testing splits. To mitigate this constraint, we adopted a leave-one-out cross-validation procedure, which is commonly recommended in small-sample neuroimaging and computational modeling research (Zhu et al., 2023; Poldrack et al., 2019) to obtain less biased estimates of generalization performance. The Methods section now provides a detailed description of this procedure, including model training, validation, and performance evaluation. In addition, following the reviewer's suggestion, we expanded the analysis to include comparisons across multiple machine learning algorithms, encompassing both linear and nonlinear models. This extension demonstrates that the predictive pattern is not dependent on a single modeling specification. These additional analyses, together with cross-validated performance metrics, are now reported to enhance transparency and analytical robustness.

Detailed descriptions of the machine learning modeling procedures can be found in the revised Methods section (page 22, line 438 to page 24, line 473, marked in blue).

Predicting Successful Deception Using ΔINS: a ML Approach

Given the relatively small sample size of the present study (N=34), conventional train–test splits may yield performance estimates that are highly sensitive to sample partitioning, thereby compromising the stability of generalization assessment. To address this issue, we adopted a leave-one-out cross-validation (LOOCV) strategy. LOOCV has been widely recommended in small-sample neuroimaging studies, as it maximizes the use of available training data while providing an approximately unbiased estimate of model generalization performance (Zhu et al., 2023; Poldrack et al., 2019). In each cross-validation iteration, data from one dyad were held out as the test set, whereas data from the remaining N-1 dyads were used for model training. This procedure was repeated N times, ensuring that each dyad served exactly once as an independent test sample.

Model Construction. ΔINS values were extracted from fNIRS channel pairs that exhibited significant contextual differences in the preceding statistical analyses and were used as input features for model construction. The successful deception rate served as the prediction target, capturing individual differences in deceptive performance across gain and loss contexts.

Model Evaluation. To avoid dependence on a single modeling assumption, multiple commonly used ML algorithms were systematically compared within a unified LOOCV framework. These algorithms included Logistic Regression, LSR, DT, Naive Bayes, and SVR. Linear models (e.g., Logistic Regression and LSR) were primarily used to assess linear predictive relationships between ΔINS and successful deception rates, whereas nonlinear models (e.g., DT and SVR) were employed to capture potential nonlinear patterns. We further evaluated classification-related performance metrics derived from model predictions, including Accuracy, Precision, Recall, and the F1 score. Accuracy reflects the overall proportion of correct predictions; Precision indexes the reliability of predicted

deceptive outcomes; Recall quantifies sensitivity to true deceptive behavior; and the F1 score represents the harmonic mean of Precision and Recall, providing a balanced measure that is particularly suitable for imbalanced class distributions or asymmetric misclassification costs (Altalhan et al., 2025; Chen & Cheng, 2023).

Model Robustness Assessment. Model robustness was further quantified by computing the Pearson's r between LOOCV-based predicted values and observed successful deception rates. This metric captures the model's ability to account for individual differences in deceptive behavior at the continuous level. To assess the statistical significance of the observed prediction performance, permutation testing was conducted to construct a null distribution. In each permutation, deception-rate labels were randomly shuffled while feature values were held constant, and the entire prediction procedure was repeated. This process was iterated 1,000 times to generate a distribution of correlation coefficients expected under chance-level prediction. The empirical r obtained from the true model was then compared against this null distribution to determine whether predictive performance exceeded random levels (Huang et al., 2025).

The corresponding evaluation results are reported in the revised Results section (page 40, line 700 to page 42, line 723, marked in blue).

Predicting Successful Deception from ΔINS: Comparative ML Analyses

To further characterize the brain–behavior relationship, we implemented exploratory ML approaches. Based on the preceding findings, ΔINS during failed deception and single-brain activation measures neither correlated significantly with deception outcomes nor contributed incremental explanatory power in hierarchical regression models. We therefore hypothesized that ΔINS during successful deception constitutes the critical neural predictor of deceptive performance.

Separate predictive models were constructed for gain and loss contexts, with ΔINS during successful deception entered as input features and successful deception rate as the target variable. To identify the optimal predictive framework, we systematically compared multiple ML algorithms encompassing both linear and non-linear approaches. Across contexts, LSR demonstrated superior predictive performance[gain context: ROC-AUC=0.929, Accuracy=0.727, **Figure 10A**; loss context: ROC-AUC=0.973, Accuracy=0.933, **Figure 10B**]. Detailed performance metrics for all models are reported in **Table 11**. Using the LSR model, ΔINS within the significant channel pair reliably predicted successful deception across contexts (gain: R^2=0.870, MSE=0.001; loss: R^2=0.266, MSE=0.004). To assess statistical significance, we conducted 5,000 label-shuffled permutation tests. In both contexts, the observed predictive performance exceeded chance levels (gain: r=0.933, p<0.001, **Figure 10C**; loss: r=0.516, p<0.001, **Figure 10D**).

Table 11. Performance evaluation of different ML models.

Model	Condition	ROC-AUC	Accuracy	Precision	Recall	F1 Score	MCC
LSR	Gain	0.929	0.727	0.750	0.857	0.800	0.386
	Loss	0.867	0.727	0.800	0.667	0.727	0.467
Logistic Regression	Gain	0.893	0.909	0.875	0.796	0.733	0.810
	Loss	0.800	0.636	0.667	0.667	0.667	0.817
Naive Bayes	Gain	0.768	0.727	0.650	0.757	0.700	0.386
	Loss	0.733	0.727	0.800	0.667	0.727	0.467
DT	Gain	0.732	0.677	0.833	0.714	0.669	0.449
	Loss	0.817	0.818	0.833	0.833	0.833	0.633
SVR	Gain	0.821	0.818	0.857	0.857	0.610	0.607
	Loss	0.667	0.545	0.600	0.500	0.545	0.100

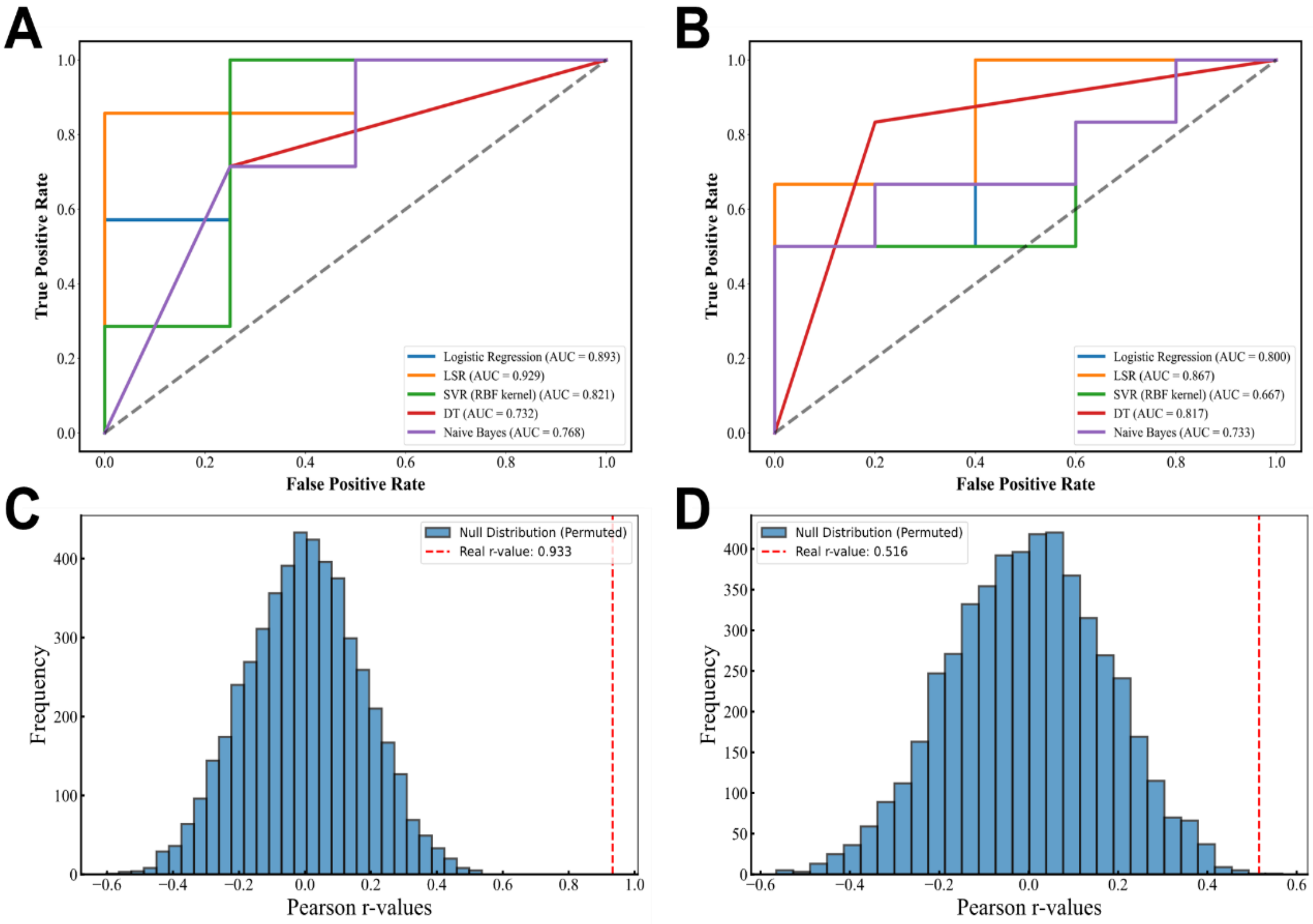

Figure 10. Comparative predictive performance of ML models across contexts. (A) ROC curves comparing ML models in the gain context. **(B)** ROC curves comparing ML models in the loss context. **(C)** Results of the 5,000-permutation test for predicting successful deception using ΔINS features in the gain context. **(D)** Results of the 5,000-permutation test for predicting successful deception using ΔINS features in the loss context.

Reference

[1] Fu, H., Tang, S., & Zhao, X. (2025). Limitations of Correlation Coefficients in Research on Functional Connectomes and Psychological Processes. *Human Brain Mapping*, *46*(10), hbm70287.

[2] Helmer, M., Warrington, S., Mohammadi-Nejad, A., …, & Murray, J. (2024). On the stability of canonical correlation analysis and partial least squares with application to brain-behavior associations. *Communications Biology*, *7*(1), 5869-5873.

[3] Shamout, F., Zhu, T., & Clifton, D. (2020). Machine Learning for Clinical Outcome Prediction. *IEEE Reviews in Biomedical Engineering*, *14*, 116-126.

[4] Wang, S., McCormick, T., & Leek, J. (2020). Methods for correcting inference based on outcomes predicted by machine learning. *Proceedings of the National Academy of Sciences of the United States of America*, *117*(48), 30266-30275.

[5] Huang, R., Gao, X., Zhang, C., …, & Liu, Y. (2025). Forewarned Is Forearmed: The single- and dual-brain mechanisms in Detectors from Dyads of Varying Social Distance During Deceptive Outcomes Evaluation. *The Journal of neuroscience*, *45*(43): e2129242025.

[6] Zhu, X., Kim, Y., Ravid, O., …, & Morey, R. A.(2023). Neuroimaging-Based Classification of PTSD Using Data-Driven Computational Approaches: A Multisite Big Data Study from the ENIGMA-PGC PTSD Consortium. *NeuroImage*, *283*, 120412.

[7] Poldrack, R., Huckins, G., & Varoquaux, G. (2019). Establishment of Best Practices for Evidence for Prediction: A Review. *JAMA psychiatry*, *77*(5), 534.

[8] Altalhan, M., Algarni, A., & Alouane, T. (2025). Imbalanced Data Problem in Machine Learning: A Review. *IEEE Access*, *13*, 13686-13699.

[9] Chen, H., & Cheng, Y. (2023). Travel Mode Choice Prediction Using Imbalanced Machine Learning. *IEEE Transactions on Intelligent Transportation Systems*, *24*(4), 3795-3808.

---

(3) The hypothesizes were not effectively motivated. For example, it was unclear why the authors hypothesized that DLPFC activation would be greater in gain contexts. The evidence presented in the introduction indicates DLPFC has a role in evaluation generally – which would still be engaged in loss contexts, of course. Similarly, the prediction of higher synchrony in gain contexts had no support, as far as I could tell.

Re: We thank the reviewer for pointing out that the motivational basis of our hypotheses was insufficiently articulated in the original manuscript. We agree that the earlier version emphasized the general role of the DLPFC in value evaluation and executive control but did not clearly distinguish its context-dependent functions, nor did it adequately explain why gain contexts—relative to loss contexts—were hypothesized to elicit greater activation and synchrony. In response, we have substantially reconstructed the theoretical framework. First, we now explicitly differentiate core subjective value encoding systems from context-modulated control systems (**see revised manuscript, page 4, lines 64 to page 4, line 70**): "Neuroeconomic studies and related meta-analyses have shown

that core subjective value representations are primarily encoded in the ventromedial prefrontal cortex and the striatum (Glimcher, 2022; Todd et al., 2009), whereas the anterior cingulate cortex and the insula are more highly involved in processing uncertainty, conflict, and affective signals (Medford & Critchley, 2010). The prefrontal cortex (PFC) plays a regulatory role by exerting top-down modulation of value signals according to contextual demands (Huang et al., 2024; Phillips et al., 2021)." This revision avoids conflating the DLPFC with general value representation. Instead, we define its functional role as context-dependent regulation and control over value signals. Thus, the DLPFC is not framed as a generic valuation region but as a control-related structure whose engagement varies with regulatory demands. Secondly, we ground our hypothesis in differences in control-demand structure across contexts. **The revised manuscript (page 5, lines 81 to 86) now states**: "Compared with loss contexts, which are often dominated by urgency and threat-related processing (Mäki-Marttunen et al., 2019), gain contexts typically require more fine-grained value comparisons among multiple potential options (Corbi & Soutschek, 2024). Under such conditions, maintaining goal-directed behavior while suppressing automatic responses becomes a key component of decision implementation (Swanson et al., 2017)." Importantly, our theoretical claim is not that loss contexts lack control processes. Rather, we argue that gain contexts more strongly rely on sustained goal maintenance and multi-option trade-offs, thereby imposing greater demands on DLPFC-mediated regulatory control. On this basis, we clarify in **the revised manuscript (page 5, lines 86 to 90)** that: "Accordingly, when deception is enacted in gain contexts, its implementation is more likely to rely on regulatory mechanisms within the PFC network, particularly DLPFC activity associated with strategic control, to support complex value trade-offs and behavioral selection. Based on this reasoning, we further propose ***Hypothesis 2: compared with loss contexts, deceptive behavior in gain contexts will be accompanied by higher DLPFC activation.***" To improve clarity, each hypothesis has now been explicitly placed at the end of its corresponding theoretical subsection.

Building on this single-brain framework, we further clarified the logic underlying our interpersonal neural synchrony hypothesis. **The revised manuscript (page 7, lines 116 to 117) states**: "ΔINS in the DLPFC reflects the alignment of control-related processes between interaction rather than deception-specific activation." Thus, INS is not interpreted as regional co-activation per se, but as the degree of coupling between control systems during interaction. Because deception requires continuous monitoring, prediction, and strategy updating, contexts that increase executive control demands at the individual level should also elevate the requirement for coordinated regulation between partners. Accordingly, we now articulate the interpersonal extension explicitly **(see revised manuscript, page 7, lines 125 to 131)**:"Extending this framework to interpersonal deception, we propose that gain contexts impose higher requirements for coordinated regulation during deceptive interaction. Because successful deception requires continuous monitoring, prediction, and adjustment, contexts that amplify prefrontal engagement at the individual level should likewise enhance interpersonal alignment within control-related networks. Accordingly,

we ***Hypothesize 3 that deceptive interactions in gain contexts will elicit higher ΔINS in the DLPFC, which will be positively associated with successful deception.***"

Reference

[1] Glimcher, P. W. (2022). Efficiently irrational: deciphering the riddle of human choice. *Trends in cognitive sciences*, 26(8), 669-687.

[2] Todd, A. H., Colin. F. C., & Antonio, R. (2009).Self-Control in Decision-Making Involves Modulation of the vmPFC Valuation System. *Science*, 324(5927), 646-648.

[3] Medford, N., & Critchley, H. (2010). Conjoint activity of anterior insular and anterior cingulate cortex: awareness and response. *Brain Structure & Function*, 214(5-6), 535-549.

[4] Huang, H., Li, R., Qiao, X., …, & Zhang, J. (2024). Attentional control influence habituation through modulation of connectivity patterns within the prefrontal cortex: Insights from stereo-EEG. *NeuroImage*, *294*, 120640.

[5] Phillips, J., Kambi, N., Redinbaugh, M., Mohanta, S., & Saalmann, Y. (2021). Disentangling the influences of multiple thalamic nuclei on prefrontal cortex and cognitive control. *Neuroscience & Biobehavioral Reviews*, *128*, 487-510.

[6] Mäki-Marttunen, V., Hagen, T., & Espeseth, T. (2019). Task context load induces reactive cognitive control: An fMRI study on cortical and brain stem activity. *Cognitive, Affective & Behavioral Neuroscience*, *19*(4), 945-965.

[7] Corbi, Z., & Soutschek, A. (2024). Neural reward system reflects individual value comparison strategy in cost-benefit decisions. *Communications Biology*, *7*(1), 1488.

[8] Swanson, A., DePoy, L., & Gourley, S. (2017). Inhibiting Rho kinase promotes goal-directed decision making and blocks habitual responding for cocaine. *Nature Communications*, *8*(1), 1915-1919.

---

(4) Despite the collection of data from the entire PFC throughout the entire task, there's only ever consideration of DLPFC activity at one moment of the task. The use of other photodiodes' activity could serve important purposes here, for example as a contrast to the DLPFC results to help the reader understand what is special to the patterns of activity in DLPFC, or even what is special in the moment in the task being analyzed.

Re: We thank the reviewer for raising this important point of clarification. We acknowledge that the wording in the original manuscript may have unintentionally conveyed the impression that our analyses focused exclusively on the DLPFC, despite the fact that data were collected and analyzed across the entire prefrontal cortex (PFC). In the revised manuscript, we have clarified that all recorded PFC channels were systematically examined at the statistical analysis stage, including subregional comparisons and time-course analyses across the full interaction period. The DLPFC emerged as the focal region of reporting not because of a priori selection or analytical restriction,

but because it was the only PFC subregion that demonstrated stable and statistically significant effects after correction for multiple comparisons in both contextual modulation and behavioral association.

In the revised Introduction, we have further emphasized that deception relies on distributed neural networks rather than a single cortical region. Specifically, we now state (**see revised manuscript, page 4, lines 62 to 64**):"Existing neurobiological evidence indicates that deception does not depend on a single brain region, but instead relies on a distributed network supporting valuation and decision-making processes (Liu et al., 2019)" We also clarify (**see revised manuscript, page 4, line 68 to page 5, line 70**): "The prefrontal cortex (PFC) plays a regulatory role by exerting top-down modulation of value signals according to contextual demands (Huang et al., 2024; Phillips et al., 2021)." These revisions explicitly situate the present study within a whole-PFC network framework rather than a single-region perspective.

Importantly, in addition to the DLPFC, we tested activation strength and interpersonal synchrony across the frontopolar area (FPA), orbitofrontal cortex (OFC), ventrolateral PFC (VLPFC), and all other recorded PFC channels within the predefined task window. None of these regions exhibited statistically significant contextual differences after correction. This spatial differentiation forms the basis for the regional specificity of the DLPFC findings. Thus, the prominence of DLPFC results does not reflect selective reporting; rather, non-significant regions serve as spatial controls, demonstrating that contextual effects are not globally distributed across the PFC but are regionally specific.

All channel-wise results are reported in Supplementary Table S2. Significant paired comparisons are highlighted in red bold font to facilitate rapid identification of positive findings.

Table S2. Contextual differences in intra-brain activation (sender/cheater) and dyadic ΔINS within the task-relevant time window.

Analysis Level	Channel	Brain Region	Gain Context	Loss Context	*t*(33)	*p*	*Cohen' d*
Intra-brain activation	CH1	DLPFC	0.0018±0.0080	0.0034±0.0071	-0.831	0.412	-0.143
during deception trials	CH2	DLPFC	0.0020±0.0070	0.0034±0.0074	-0.805	0.427	-0.138
(sender/cheater)	CH3	FPA	0.0029±0.0074	0.0001±0.0070	1.453	0.156	0.249
	CH4	FPA	0.0026±0.0070	0.0033±0.0070	-0.372	0.712	-0.064
	CH5	FPA	0.0022±0.0071	0.0032±0.0078	-0.569	0.573	-0.098
	CH6	DLPFC	0.0010±0.0081	0.0028±0.0071	-1.179	0.247	-0.202
	CH7	DLPFC	0.0016±0.0069	0.0046±0.0060	-1.787	0.083	-0.306
	CH8	VLPFC	0.0011±0.0075	0.0018±0.0072	-0.421	0.676	-0.072
	CH9	DLPFC	0.0026±0.0071	0.0022±0.0074	0.202	0.841	0.035
	CH10	FPA	0.0014±0.0061	0.0037±0.0067	-1.389	0.174	-0.238
	CH11	FPA	0.0023±0.0084	0.0023±0.0070	-0.015	0.988	-0.003
	CH12	FPA	0.0044±0.0076	0.0027±0.0074	1.064	0.295	0.183
	CH13	FPA	0.0009±0.0073	0.0034±0.0074	-1.557	0.129	-0.267
	**CH14**	**DLPFC**	**0.0004±0.0039**	**-0.0011±0.0034**	**2.565**	**0.015**	**0.440**
	CH15	VLPFC	0.0032±0.0066	0.0030±0.0068	0.126	0.900	0.022
	CH16	VLPFC	0.0027±0.0079	0.0017±0.0062	0.616	0.542	0.106

	CH17	OFC	0.0035±0.0072	0.0052±0.0065	-0.931	0.358	-0.160
	CH18	OFC	0.0012±0.0074	0.0024±0.0072	-0.713	0.481	-0.122
	CH19	OFC	0.0029±0.0063	0.0028±0.0063	0.084	0.934	0.014
	CH20	OFC	0.0048±0.0070	0.0036±0.0057	0.766	0.449	0.131
	CH21	OFC	0.0018±0.0077	0.0048±0.0061	-1.807	0.080	-0.310
	CH22	VLPFC	0.0035±0.0075	0.0029±0.0079	0.343	0.733	0.059
Sender (cheater)-	CH1-CH1	DLPFC	0.2079±0.1365	0.2447±0.1706	-0.881	0.385	-0.151
Receiver (detector)	CH2-CH2	DLPFC	0.2624±0.1203	0.2117±0.1618	1.496	0.144	0.257
ΔINS during successful	CH3-CH3	FPA	0.2346±0.1208	0.1881±0.1373	1.465	0.152	0.251
deception	CH4-CH4	FPA	0.1966±0.0919	0.1750±0.1595	0.679	0.502	0.116
	CH5-CH5	FPA	0.2425±0.1080	0.1909±0.1433	1.558	0.129	0.267
	CH6-CH6	DLPFC	0.2062±0.0923	0.1966±0.1261	0.310	0.758	0.053
	CH7-CH7	DLPFC	0.2072±0.1255	0.1861±0.1191	0.690	0.495	0.118
	CH8-CH8	VLPFC	0.1726±0.1307	0.2276±0.1273	-1.687	0.101	-0.289
	CH9-CH9	DLPFC	0.2857±0.1490	0.2725±0.1518	0.376	0.709	0.065
	CH10-CH10	FPA	0.2499±0.0842	0.2336±0.1096	0.723	0.475	0.124
	CH11-CH11	FPA	0.2023±0.1130	0.1777±0.1149	0.751	0.458	0.129
	CH12-CH12	FPA	0.2263±0.1027	0.1947±0.1515	1.215	0.233	0.208
	CH13-CH13	FPA	0.2786±0.0981	0.2890±0.1078	-0.405	0.688	-0.070
	**CH14-CH14**	**DLPFC**	**0.2220±0.1166**	**0.4396±0.1183**	**-8.656**	**<0.001**	**-1.485**
	CH15-CH15	VLPFC	0.2008±0.1276	0.1972±0.1134	0.123	.903	0.021
	CH16-CH16	VLPFC	0.1876±0.1786	0.1882±0.1348	-0.015	.989	-0.002
	CH17-CH17	OFC	0.1953±0.1279	0.1637±0.0926	1.143	.261	0.196
	CH18-CH18	OFC	0.1880±0.1018	0.1961±0.1230	-0.295	.770	-0.051
	CH19-CH19	OFC	0.2078±0.1460	0.1671±0.1211	1.143	.261	0.196
	CH20-CH20	OFC	0.1806±0.0965	0.1935±0.1386	-0.431	.670	-0.074
	CH21-CH21	OFC	0.2539±0.1112	0.1947±0.1208	1.924	.063	0.330
	CH22-CH22	VLPFC	0.2452±0.1099	0.2148±0.1156	1.066	.294	0.183

Note: Intra-brain activation values should be multiplied by 10^{-3}.

In response to concerns regarding time-window selection, we further clarify that the interaction-phase window used for analysis was predefined at the experimental design stage. The theoretical rationale is that deception was conceptualized as a dynamic, bidirectional regulatory process occurring primarily during real-time information exchange. This operationalization is consistent with prior work (Chen et al., 2020; Huang et al., 2025). Accordingly, the selection of the task window was not based on post hoc peak identification but was anchored to a theoretically defined phase of interaction specified in advance.

As detailed in the revised Methods section (page 19, lines 364 to 368), the fNIRS time-window procedure is described as follows:

Intra-Brain Activation Analysis. Using the SPM-based fNIRS analysis framework (Tak & Ye, 2014), HbO time series were extracted from all 34 senders. Analyses focused on neural response patterns during the verbal statement

phase (15 s), which constituted the primary task window of interest. Trial start times and durations were extracted to generate stimulus designs and convolved with hemodynamic response functions using NIRS-SPM.

Based on these revisions, we have now made it explicit in the manuscript that: (1) Subsequent analyses covered the entire PFC network. (2) The DLPFC findings reflect statistically emergent regional specificity rather than prior selection. (4) Non-significant channels serve as essential spatial comparison controls. (5)The analysis time window was theory-driven and predefined. We believe these clarifications directly address the reviewer's concerns regarding analytical scope, regional specificity, and temporal selection, and substantially enhance the transparency and rigor of the manuscript.

Reference

[1] Liu, P., Shen, H., & Ji, S. (2019). Functional Connectivity Pattern Analysis Underlying Neural Oscillation Synchronization during Deception. *Neural Plasticity*, *2019*, 1-10.

[2] Huang, H., Li, R., Qiao, X., …, & Zhang, J. (2024). Attentional control influence habituation through modulation of connectivity patterns within the prefrontal cortex: Insights from stereo-EEG. *NeuroImage*, *294*, 120640.

[3] Phillips, J., Kambi, N., Redinbaugh, M., Mohanta, S., & Saalmann, Y. (2021). Disentangling the influences of multiple thalamic nuclei on prefrontal cortex and cognitive control. *Neuroscience & Biobehavioral Reviews*, *128*, 487-510.

[4] Chen, M., Zhang, T., Zhang, R., ..., & Li, X. (2020). Neural alignment during face-to-face spontaneous deception: does gender make a difference? *Human brain mapping*, *41*(17), 4964-4981.

[5] Huang, R., Gao, X., Zhang, C., …, & Liu, Y. (2025). Forewarned Is Forearmed: The single- and dual-brain mechanisms in Detectors from Dyads of Varying Social Distance During Deceptive Outcomes Evaluation. The *Journal of neuroscience*, *45*(43): e2129242025.

---

(5) The calculation of power was unclear – the authors simply state that "according to the calculation the required sample size was…", without stating what the power analysis was conducted on (a correlation? T-test? One-sample? Etc.). A little more detail here would be helpful as much of the rest of the data seems to reflect paired samples t-tests (with ~30 points) and small correlations (with ~30 points), and their power analysis seems to call for double that number (68 datapoints).

Re: We sincerely thank the reviewer for raising this important point regarding the clarity of our power analysis. In the revised manuscript, we have now provided an explicit clarification of the statistical structure underlying the a priori power estimation. Specifically, the power analysis was conducted based on a paired-samples t-test,

corresponding to the core comparison of behavioral and neural differences between gain and loss contexts within dyads. Because contextual framing (gain vs. loss) was a within-subject factor and all statistical analyses were performed using the dyad as the independent unit of analysis, the unit of power estimation was likewise the dyad, rather than treating 68 individuals as 68 independent observations. In other words, the 34 dyads in the present study correspond to 34 paired observations, not 68 independent samples.

We conducted the power analysis using G*Power (version 3.1.9.7) under the following parameters: a medium effect size (f= 0.25, corresponding approximately to d≈0.5 in a within-subject design), a significance level of α= 0.01 (a conservative threshold adopted to reduce Type I error risk under multiple testing), and statistical power of $1-\beta$= 0.80. Under these assumptions, the required minimum sample size was 34 dyads. This estimation is fully aligned with the statistical structure of the primary inferential tests reported in the manuscript (e.g., paired-samples t-tests comparing deception rate and successful deception rate between gain and loss contexts).

As noted by the reviewer, correlation analyses (e.g., brain–behavior associations) were also conducted. We acknowledge that these analyses are exploratory in nature. The statistical unit for these analyses was likewise the dyad (N = 34), which provides sufficient sensitivity to detect medium or larger effects, but not small effects. Importantly, however, the a priori power analysis was designed around the central research question—namely, the within-dyad modulation of deceptive behavior by gain versus loss contexts—rather than separately estimating required sample sizes for all exploratory correlational analyses.

Please see the revised manuscript, page 9, lines 168 to 170, or the highlighted blue text:

A priori power analysis was conducted using G*Power 3.1.9.7 (Faul et al., 2007) (medium effect size f=0.25, α=0.01, 1-β=0.80, paired-samples t-test), indicating a minimum required sample size of 34 dyads.

Reference

[1] Faul, F., Erdfelder, E., Lang, A. G., & Buchner, A. (2007). G*Power 3: A flexible statistical power analysis program for the social, behavioral, and biomedical sciences. *Behavior Research Methods*, *39*(2), 175-191.

---

(6) Many methods details are unclear (e.g., "data from 34 pairs of participants, including 18 female and 16 male participants" I think should be "18 female and 16 male participant pairs"? Similarly, it's unclear to me what the message text sent during the task means, i.e. what "Option A is more suitable for you and is more favorable to you" is about). Double-check throughout to ensure accuracy and completeness.

Re: We sincerely thank the reviewer for identifying potentially ambiguous wording in the Methods section of the original manuscript. In response, we have conducted a line-by-line review and unified revision of the Methods to ensure accuracy, completeness, and internal consistency throughout.

First, regarding the unclear description of the sample structure, we have revised the wording to explicitly designate the dyad as the unit of analysis. The final sample is now clearly described as 34 same-gender stranger dyads, including 18 female dyads and 16 male dyads, thereby avoiding formulations such as "18 female and 16 male participants," which could be misinterpreted as referring to individual counts. **The revised text reads (page 9, lines 173-175, highlighted in blue)**: "The final sample therefore consisted of 34 same-gender stranger dyads, including 18 female dyads (age: *M*=21.33, *SD*=2.07) and 16 male dyads (age: *M*=20.56, *SD*=1.29)." Second, concerning the semantic interpretation of the message content in the task, we have clarified that the message does not carry additional linguistic meaning beyond serving as a standardized recommendation signal. Specifically, the sender presented a color cue (red or blue) to the receiver, which was operationally defined as indicating that "the option associated with this color is more beneficial for you in this round." The phrase in the original manuscript—"Option A is more suitable for you and is more favorable to you"—was intended to illustrate the recommendation's intended meaning, but we acknowledge that it may have introduced unintended semantic ambiguity. In the revised manuscript, this wording has been replaced with a more direct and operational description. Furthermore, we now explicitly state that during the verbal statement phase, the sender's first sentence was required to reiterate that the recommended option was "more beneficial" for the receiver. This clarification appears in the revised manuscript **(page 12, lines 235 to 241, highlighted in blue):**" **(2) Recommendation cue (1s).** The sender conveyed a brief nonverbal recommendation to the receiver using a color cue (red or blue), semantically equivalent to "the option associated with this color is more beneficial for you in this round." **(3) Verbal statement (15 s).** Following an auditory cue (1000 Hz, 500 ms), the sender delivered a one-way verbal statement. The first sentence was required to reiterate that the recommended option was "more beneficial" for the receiver, after which the sender could freely provide task-relevant information." In addition, we have harmonized all related terminology throughout the manuscript—including sample unit designation, role naming, description of the recommendation signal, figure captions, and main-text references—to prevent discrepancies between early summaries and later methodological details.

---

(7) The authors write that "we used multiple linear regression to rule out causal relationships between gender and other demographic variables and deceptive behavior," but this is unclear how! This is not a trivial step, nor are any results ever discussed here. The supplementary file was empty, and no further information was provided.

Re: We thank the reviewer for the important comments regarding the clarity of our statistical wording and the presentation of regression results. In the original manuscript, the phrasing suggesting that we had "excluded" causal relationships between demographic variables (e.g., gender) and deceptive behavior was imprecise and required substantive revision. This has now been corrected in the revised manuscript, together with a more detailed presentation of the corresponding statistical analyses and results.

To clarify, the present study neither attempted nor was able to "exclude" causal relationships between demographic variables and deception through regression analysis. Our intended meaning was that, after statistically controlling for demographic variables and other relevant behavioral indicators, we examined whetherΔINS retained independent and incremental predictive value for deceptive outcomes. To eliminate ambiguity, all relevant statements have been revised to explicitly state that the regression analyses were conducted to assess predictive contribution rather than to support causal inference. In the revised Methods section (Statistical Analysis), we now provide a detailed description of the hierarchical linear regression procedure. Predictors were entered into the model in theoretically and temporally structured blocks. Specifically:

- **Step 1:** demographic variables (e.g., gender and educational background);
- **Step 2:** behavioral indicators from the deceptive interaction (e.g., frequency of direct eye contact during successful deception);
- **Step 3:** intra-brain activation coefficients (β values) from channels showing significant contextual effects;
- **Step 4:** ΔINS during failed deception;
- **Step 5:** ΔINS during successful deception.

At each stage, overall model fit (R^2) and incremental changes in explained variance (ΔR^2) were evaluated to determine whether ΔINS accounted for additional variance beyond demographic and intra-brain neural measures. This analytical framework allowed us to test the unique predictive contribution of ΔINS while clearly maintaining a non-causal interpretive boundary.

Please see the revised manuscript, page 21, line 425 to page 22, line 437, or the highlighted blue text:

Regression Analysis. To evaluate whether ΔINS provides incremental predictive power beyond demographic and intra-brain neural measures, hierarchical linear regression analyses were conducted. This approach was used to test whether dyadic ΔINS significantly improved the prediction of deceptive outcomes above and beyond established predictors. Predictors were entered into the model in a block-wise manner. In Step 1, demographic variables (e.g., gender and educational background) were included. Step 2 added interaction-related behavioral variables, specifically the frequency of direct eye contact during the interaction. In Step 3, task-related activation coefficients (*β* values) from channels showing significant effects were entered to account for intra-brain neural contributions. In

Steps 4 and 5, ΔINS during failed deception and successful deception, respectively, were sequentially introduced. At each step, changes in model fit were evaluated to determine whether ΔINS explained additional variance beyond demographic, behavioral, and intra-brain neural measures, thereby assessing its unique predictive value for deceptive outcomes.

The corresponding regression results are fully reported in the revised Results section and presented in Table 7-Table 10. Across both gain and loss contexts, significant improvement in model fit was observed only when ΔINS during successful deception was entered into the model. In contrast, demographic variables, intra-brain activation measures, and ΔINS during failed deception did not significantly increase explained variance. To further assess the robustness of this finding, we conducted confirmatory hierarchical regression analyses in which the order of predictor entry was reversed. Specifically, ΔINS during successful deception was entered first, followed by demographic variables, intra-brain activation measures, eye-contact frequency, and ΔINS during failed deception. The results showed that none of the subsequently entered predictors significantly improved model fit. This pattern provides convergent support for the unique predictive contribution of ΔINS during successful deception to deceptive outcomes. Finally, regarding the concern that the supplementary materials were previously empty, all relevant regression results have now been integrated directly into the main text tables to avoid fragmentation of information. Should the journal prefer that these analyses be retained in supplementary format, we would be pleased to revise the presentation accordingly based on editorial guidance.

Please see the revised manuscript, page 37, line 679 to page 40, line 700, or the highlighted blue text:

Regression Analyses: Unique Predictive Contribution of ΔINS

To determine whether ΔINS provides predictive value beyond single-brain indices and demographic factors, we conducted hierarchical linear regression analyses separately for gain and loss contexts. Demographic variables, single-brain activation measures, and ΔINS were entered sequentially to evaluate their incremental explanatory power. As shown in **Table 7** (gain context) and **Table 8** (loss context), significant improvements in model fit were observed only when ΔINS during successful deception was entered into the model (ΔR^2_{gain}=0.576, ΔF_{gain}=54.093, p<0.001; ΔR^2_{loss}=0.138, ΔF_{loss}=6.248, p=0.018). In contrast, neither single-brain activation measures (ΔF<0.116, ps>0.736) nor ΔINS during failed deception (ΔF<2.410, ps>0.132) significantly increased explained variance.

To further verify the robustness of this effect, we conducted confirmatory analyses by reversing the order of predictor entry. ΔINS during successful deception was entered first, followed by ΔINS during failed deception, single-brain activation measures, eye-contact frequency during successful deception, and finally demographic variables. As presented in **Table 9** (gain context) and **Table 10** (loss context), none of the subsequently entered variables significantly improved model fit (failed-deception ΔINS: ΔF<1.390, ps>0.247; single-brain measures:

$\Delta F<0.159$, $ps>0.693$; eye contact: $\Delta F<3.842$, $ps>0.059$; demographics: $\Delta F<1.292$, $ps>0.290$).

Table 7. Results of hierarchical linear regression model (gain context).

Step	R^2	Adj. R^2	F values	Model sig.	ΔR^2	ΔF	ΔF sig.
1<br>Demographic variables	0.013	-0.047	0.218	0.806			
2<br>Eye contact during successful deception	0.059	-0.098	0.375	0.861	0.046	0.487	0.694
3<br>Single-brain activation (β)	0.063	-0.131	0.323	0.920	0.004	0.116	0.736
4<br>INS during failed deception	0.137	-0.079	0.634	0.724	0.074	2.410	0.132
5<br>INS during successful deception	0.713	0.627	8.369	<0.001	0.576	54.093	<0.001

Table 8. Results of hierarchical linear regression model (loss context).

Step	R^2	Adj. R^2	F values	Model sig.	ΔR^2	ΔF	ΔF sig.
1<br>Demographic variables	0.166	0.115	3.275	0.051			
2<br>Eye contact during successful deception	0.171	0.093	2.201	0.107	0.005	0.211	0.649
3<br>Single-brain activation (β)	0.17	0.064	1.600	0.199	<0.001	<0.001	0.995
4<br>INS during failed deception	0.220	0.090	1.690	0.168	0.049	1.871	0.182
5<br>INS during successful deception	0.358	0.225	2.696	0.033	0.138	6.248	0.018

Table 9. Validation analysis results of hierarchical linear regression model (gain context).

Step	R^2	Adj. R^2	F values	Model sig.	ΔR^2	ΔF	ΔF sig.
1<br>INS during successful deception	0.659	0.649	65.804	<0.001	0.659	65.804	<0.001
2<br>INS during failed deception	0.662	0.642	32.379	<0.001	0.003	0.303	0.586
3<br>Single-brain activation (β)	0.664	0.633	21.089	<0.001	0.002	0.159	0.693
4<br>Eye contact during successful deception	0.701	0.663	18.182	<0.001	0.037	3.842	0.059
5<br>Demographic variables	0.702	0.640	11.391	<0.001	0.001	0.046	0.955

Table 10. Validation analysis results of hierarchical linear regression model (loss context).

Step	R^2	Adj. R^2	F values	Model sig.	ΔR^2	ΔF	ΔF sig.
1	0.262	0.240	12.059	0.001			

INS during successful deception							
2	0.292	0.249	6.793	0.003	0.030	1.390	0.247
INS during failed deception							
3	0.292	0.225	4.393	0.011	<0.001	0.002	0.961
Single-brain activation (β)							
4	0.301	0.211	3.335	0.022	0.009	0.406	0.529
Eye contact during successful deception							
5	0.358	0.225	2.696	0.033	0.057	1.292	0.290
Demographic variables							

We again thank the reviewer for emphasizing the importance of rigorous statistical inference. This comment has substantially improved the clarity and methodological precision of our statistical reporting and result presentation.

---

(8) It was unclear whether the task has any consequences to deception. Is there any way in which the receiver can find out what the actual matrix was (so as to identify the presence of deception), did outcome amounts vary, or was there any way the receiver could respond to the sender to e.g. punish deception or otherwise communicate anything or do anything to the sender? Given that the authors write "The receiver did not know the veracity of the sender's message, and the sender did not know if they had succeeded in deceiving the receiver," it was unclear what risk is present to the sender in this game. They can literally never be found out. It's also the case that neither the sender nor the receiver can do any learning or adjustment if they never find out if they were successful (in deceiving or in choosing the highest value option). I'd recommend the authors discuss what optimal behavior would be here as a way of clarifying incentives and/or the cognitive processes senders and receivers are expected to engage during the task.

Re: We thank the reviewer for this important clarification request. In the originally submitted manuscript, we did not sufficiently specify several critical structural aspects of the task, including: (1) whether deceptive outcomes had real consequences, (2) whether receivers could verify the payoff matrix, (3) whether punishment or retaliation mechanisms were present, (4) what type of risk the sender assumed under no-feedback conditions, and (5) how optimal decisions were defined for both parties. In response, we have revised the Methods section to provide a clearer specification of information availability, payoff structure, and the source of risk within the task.

(1) "Do deceptive outcomes actually occur?" — Yes, and they directly determine dyadic earnings.

In the revised Participants section (page 10, lines 186 to 189), we have clarified the payment structure of the sender–receiver spontaneous deception task: "To enhance the ecological validity of gain and loss contexts, one trial was randomly selected from each context (gain and loss) at the end of the task to determine performance-based monetary rewards or penalties. Final payment was calculated as 15*RMB*+R_{gain}-R_{loss}, yielding a total compensation range of 0-45*RMB*." This revision explicitly indicates that deceptive outcomes were consequential and implemented through a random incentive mechanism. We further strengthened the description of the payoff-conflict structure in the Task Procedure section, clarifying that participants' decisions in each trial directly affected real monetary outcomes, and that the payoff matrix varied across trials under a strict conflict-of-interest structure **(see the revised manuscript, page 13, lines 252 to 258, or the highlighted blue text)**: "In each trial, the payoff structure was explicitly designed to induce a direct conflict of interest between the sender and receiver: a higher payoff for the sender necessarily corresponded to a lower payoff for the receiver, and vice versa. This structure was identical in both gain and loss contexts. Importantly, only one option in each trial was objectively advantageous for the receiver (i.e., higher gain or lower loss), whereas the alternative option was disadvantageous. This design has been shown to effectively elicit spontaneous information manipulation based on immediate payoff trade-offs (Chen et al., 2020)." These clarifications make explicit that behavioral decisions had real economic consequences and that deception outcomes were implemented within a genuine incentive-compatible structure.

(2) "Can receivers verify the true payoff matrix?" — No; they rely exclusively on interaction cues

We have also clarified in the revised manuscript that receivers (detectors) did not have access to the numerical values in the payoff matrix and therefore could not independently verify the true outcome structure. Instead, they relied solely on the sender's nonverbal and verbal recommendation cues when making their decisions.

Please see the revised manuscript, page 13, lines 259 to 261, or the highlighted blue text:

Receivers could not view the numerical values in the outcome matrix and relied solely on the sender's recommendation cues (color signals and verbal statements), rendering direct verification of information impossible (Huang et al., 2025).

This clarification makes explicit that information asymmetry was structurally embedded in the task and that receivers' judgments depended entirely on interactive communication rather than independent matrix inspection.

（3）Was there a punishment or retaliation mechanism? — No; this was an intentional design decision

We confirm that the experimental paradigm did not include any explicit punishment, retaliation, or reputational loss mechanisms. This was not an oversight but a deliberate theoretical and methodological choice. The primary aim of the present study was not to examine how deception is suppressed under reputational threat or social sanction, but

rather to investigate how individuals perform value trade-offs and belief inference under conditions of unverifiable information and structural payoff conflict. Introducing punishment or retaliation would have inevitably activated additional psychological processes beyond the scope of the current investigation, including reputation management motives (Molho & Wu, 2021; Kroupa, 2014), expectations of future interaction (Skatova et al., 2017), deterrence-based risk assessment (Elshout et al., 2019), and affect-driven retaliation (Jensen, 2010). These mechanisms would substantially alter the psychological basis of deceptive behavior, shifting it from immediate expected-utility computation toward long-term strategic optimization.

From an experimental control perspective, the present task was structured as a one-shot asymmetric information interaction. Introducing punishment would effectively transform the paradigm into a repeated-game structure, encouraging cross-trial strategic updating and learning. This would conflict with the theoretical objective emphasized in the revised manuscript. In other words, the present paradigm focuses specifically on how individuals engage in information manipulation under gain–loss framing when no external deterrence mechanisms are present.

Please see the revised manuscript, page 13, lines 263 to 266, or the highlighted blue text:

This design eliminated explicit punishment, feedback, or learning mechanisms, ensuring that task performance primarily reflected real-time information weighting and decision-making under uncertainty rather than strategic learning or reputation management.

At the same time, we agree that incorporating punishment or reputational mechanisms in future studies represents a highly valuable extension. Manipulating the presence of social sanctions, reputational feedback, or repeated interaction expectations would allow systematic comparison of the psychological and neural mechanisms underlying deception under different deterrence conditions. We sincerely appreciate this constructive suggestion, which not only helped us more clearly define the theoretical boundaries of the present study but also provides an important direction for future research.

（4）What risk does the sender assume under no-feedback conditions?

We have clarified in the revised manuscript that although no reputational exposure or punitive consequences were present, this does not imply that the sender could act without constraint. The risk assumed by the sender is not social exposure risk but expected-value uncertainty arising from belief misestimation.

In the payoff matrix, each trial was structured as a strict conflict of interest: a higher payoff for the sender necessarily corresponded to a lower payoff for the receiver, and vice versa. Moreover, in each trial only one option was objectively advantageous for the receiver (Huang et al., 2025). If the sender recommended the receiver-disadvantageous option, the sender's final payoff depended entirely on whether the receiver accepted the recommendation. If accepted, the sender gained a higher payoff; if rejected, the sender's payoff decreased. Thus,

deception in this paradigm increases outcome variance. When making a disadvantageous recommendation, the sender is effectively performing an expected-utility calculation based on a subjective estimate of the probability that the receiver will comply. If this belief is biased—for example, if the sender overestimates the receiver's trust—the resulting decision may reduce the sender's payoff. This belief-dependent payoff uncertainty constitutes the core risk assumed by the sender under no-feedback conditions.

Furthermore, as explicitly stated in the revised manuscript **(page 13, lines 261 to 263)**: "Meanwhile, senders received no feedback during or after the experiment regarding whether their information successfully influenced the receiver's decision."Because no cross-trial feedback was available, senders could not update their beliefs about receiver behavior. Consequently, belief errors could not be corrected, rendering the risk persistent and cumulative across trials. This uncertainty environment is precisely the decision context targeted in the present study: how individuals perform value trade-offs and belief inference under gain–loss framing in the absence of deterrence and learning signals.

In sum, the cheater's risk in this task is not the social risk of being exposed, but the economic risk of misestimating the detector's compliance probability.

（5）How do both parties make optimal decisions?

We further clarified the decision logic by explicitly defining the payoff-conflict structure and information asymmetry in the revised manuscript **(page 13, lines 252 to 263)**:"In each trial, the payoff structure was explicitly designed to induce a direct conflict of interest between the sender and receiver: a higher payoff for the sender necessarily corresponded to a lower payoff for the receiver, and vice versa. This structure was identical in both gain and loss contexts. Importantly, only one option in each trial was objectively advantageous for the receiver (i.e., higher gain or lower loss), whereas the alternative option was disadvantageous. This design has been shown to effectively elicit spontaneous information manipulation based on immediate payoff trade-offs (Chen et al., 2020). Participants were explicitly informed that deceptive or misleading communication was permitted in the task. Receivers could not view the numerical values in the outcome matrix and relied solely on the sender's recommendation cues (color signals and verbal statements), rendering direct verification of information impossible (Huang et al., 2025). Meanwhile, senders received no feedback during or after the experiment regarding whether their information successfully influenced the receiver's decision." This means that both parties were aware that they were operating under a conflict-of-interest structure; however, only the cheater had access to the specific numerical payoff information, and the detector was unable to verify the truthfulness of the recommendation. Under this relatively ambiguous information structure, optimal decision-making did not depend on punishment or exposure mechanisms, but rather on belief-based inference regarding the payoff structure and the other party's likely behavior. For the

cheater, the decision essentially constituted an expected-utility computation. If the cheater subjectively estimated that the detector had a high probability of accepting the recommendation, then providing a recommendation disadvantageous to the detector could yield a higher expected payoff. Conversely, if the cheater anticipated that the detector was more likely to reject the recommendation, then an honest recommendation would represent a more stable and payoff-secure strategy. As explicitly stated in the revised manuscript (**page 13, lines 262 to 263**): "senders received no feedback during or after the experiment regarding whether their information successfully influenced the receiver's decision." This indicates that the cheater could not engage in cross-trial learning through feedback. Accordingly, behavior in this paradigm reflects real-time value trade-offs and subjective belief estimation, rather than long-term strategic optimization. For the detector, optimal behavior required inferring the cheater's underlying incentives within the known conflict-of-interest structure, and evaluating the credibility of the recommendation by integrating contextual framing and interaction cues, in order to select the option most likely to maximize personal payoff.

In summary, the revised manuscript now provides systematic clarification of the task's economic consequences, information availability, payoff-conflict structure, source of risk, and decision logic. We explicitly state that deceptive behavior in this paradigm carries real monetary consequences but does not include explicit punishment or feedback mechanisms. The risk borne by the cheater arises from misestimation of the detector's compliance probability and the resulting expected-value uncertainty, rather than from social exposure risk. Decision-making for both parties is grounded in a one-shot asymmetric information structure under a clearly defined conflict of interest. We hope these revisions adequately address your concerns regarding the task's incentive structure, risk characterization, and optimal decision logic, and that they enhance the clarity and rigor of the methodological description. We again sincerely appreciate your critical questions, which have substantially improved the completeness of our task specification.

Reference

[1] Huang, R., Gao, X., Zhang, C., …, & Liu, Y. (2025). Forewarned Is Forearmed: The single- and dual-brain mechanisms in Detectors from Dyads of Varying Social Distance During Deceptive Outcomes Evaluation. The *Journal of neuroscience*, *45*(43): e2129242025.

[2] Chen, M., Zhang, T., Zhang, R., ..., & Li, X. (2020). Neural alignment during face-to-face spontaneous deception: does gender make a difference? *Human brain mapping*, *41*(17), 4964-4981.

[3] Molho, C., & Wu, J. (2021). Direct punishment and indirect reputation-based tactics to intervene against offences. *Philosophical Transactions of the Royal Society B*, *376*(1838), 289-301.

[4] Kroupa, S. (2014). Love or fear: Can punishment promote cooperation? *Evolutionary Anthropology: Issues*, *23*(6), 229-240.

[5] Skatova, A., Spence, A., Leygue, C., & Ferguson, E. (2017). Guilty repair sustains cooperation, angry

retaliation destroys it. *Scientific Reports*, *7*(1), srep46709.
[6] Jensen, K. (2010). Punishment and spite, the dark side of cooperation. *Philosophical Transactions of the Royal Society B: Biological Sciences*, *365*(1553), 2635 - 2650.

---

(9) Why was the order of gain and loss blocks fixed as ABBA? This presents a clear confound that should be acknowledged and discussed.

Re: We thank the reviewer for the careful attention to the issue of experimental block order. We understand and acknowledge the validity of this concern, and we would like to further clarify the central research question of the present study and the functional role of block order within the experimental design.

First, the primary objective of this study was not simply to compare the main effects of gain versus loss contexts. Rather, our central aim was to examine how gain–loss framing modulates cheater behavior and the dynamic decision-making processes that unfold during interaction. In this framework, gain and loss blocks served primarily as contextual modulation variables rather than as isolated conditions of interest.

Second, although an ABBA block structure was implemented at the within-subject level, we counterbalanced the starting context at the between-subject level. Specifically, approximately half of the participants began with the gain context, while the remaining half began with the loss context. This design effectively minimized potential systematic order effects that could have arisen if all participants had started under the same contextual condition.

Please see the revised manuscript, page 10, lines 193 to 200, or the highlighted blue text:

The study employed a single-factor within-subject experimental design, with contextual framing (gain vs. loss) as the within-subject independent variable. All participants completed the task under both gain and loss conditions. To minimize potential order and fatigue effects associated with repeated task exposure, an ABBA block structure was implemented at the within-subject level. In addition, the initial context was counterbalanced across participants, such that half of the participants first completed the task in the gain context, whereas the other half began in the loss context. This control strategy was adopted to reduce potential confounding effects of contextual order on both behavioral and neural measures.

We sincerely thank the reviewer for raising this critical point. This suggestion has helped us more clearly delineate the research question and enhance the transparency of the experimental design.

---

(10) Were any trials dropped because of failure to keep to the strict timeline outlined in Fig. 1? What happened if someone e.g. took longer than the allowed time to talk or enter a response?

Re: We thank the reviewer for drawing attention to the issue of task-timeline consistency. This concern is crucial for ensuring the comparability and reproducibility of both behavioral and neural data. In the revised manuscript, we have now explicitly clarified that trials in which participants failed to adhere to the predefined timing windows (as illustrated in **Figure 1**) were terminated or excluded according to predetermined criteria to ensure data quality.

Specifically, during the final decision phase, if the detector did not make a selection within 4 seconds, the trial was automatically terminated by the program and excluded from subsequent behavioral and neural analyses.

Please see the revised manuscript, page 13, lines 248 to 249, or the highlighted blue text:

Trials in which participants failed to respond within the prescribed time window were automatically terminated and excluded from subsequent analyses.

For the verbal statement phase (15 s), we likewise implemented strict duration control and post hoc quality screening procedures. Specifically, trials were excluded from subsequent neural analyses if the sender's speaking duration was abnormally short (i.e., below M-3×SD), prematurely terminated, or exceeded the predefined 15-second limit (in which case the experimenter intervened and instructed the sender to stop immediately).

Please see the revised manuscript, page 18, lines 347 to 351, or the highlighted blue text:

Given the unconstrained nature of verbal statements during the speech phase, post hoc trial-level screening was performed. Specifically, trials were excluded if the sender's effective speaking duration was abnormally short or prematurely terminated (defined as durations shorter than M-3×SD), or if the speaking duration exceeded the predefined 15-s limit (in which case the experimenter intervened). These trials were not included in subsequent neural analyses.

We further reported the actual number and proportion of excluded trials in the Results section **(see the revised manuscript, page 29, lines 565 to 571, or the highlighted blue text)** to provide full transparency regarding data

In accordance with the predefined data-cleaning criteria, trials with atypical speaking durations during the verbal statement were excluded. 79 trials were removed due to insufficient quality, including 26 trials in which the sender exceeded the allowed speaking duration. In addition, one dyad was excluded at the data-analysis stage due to excessive fNIRS artifacts. During preprocessing, three bad channel was removed from all participants, resulting in an overall channel retention rate of 99.60%. Importantly, exclusion rates did not differ between gain and loss contexts ($p=0.869$), minimizing the likelihood of condition-specific bias."

We again thank the reviewer for raising this important concern. This comment prompted us to clarify the procedures for handling time violations and protocol deviations in the revised manuscript, thereby enhancing the methodological rigor of the study.

---

(11) The text "Option A is more suitable for you and is more favorable to you" was mentioned at one point in the methods, but when the task and setup were described more later, this sentence was not present. The authors should clarify whether this sentence was present in the method or not.

Re: We thank the reviewer for pointing out the inconsistency in the task description. In the revised manuscript, we have standardized and clarified the relevant statements. Specifically, the phrase "Option A is more suitable for you and is more favorable to you" was not presented as a fixed on-screen textual instruction. Rather, it served as an abstract representation of the core semantic requirement that the sender was instructed to convey during the verbal statement phase. In the formal task, at the beginning of the 15 s verbal statement period, the sender was required to explicitly communicate that the recommended option was "more beneficial" to the receiver before providing any additional task-related information. Thus, this sentence was not displayed verbatim within the experimental interface, but instead functioned as a conceptual description of the task rule.

To eliminate potential ambiguity, we have revised the Task Procedure section accordingly **(see the revised manuscript, page 12, lines 235 to 242, or the highlighted blue text)**:

(2) Recommendation cue (1s). The sender conveyed a brief nonverbal recommendation to the receiver using a color cue (red or blue), semantically equivalent to "the option associated with this color is more beneficial for you in this round." (3) Verbal statement (15 s). Following an auditory cue (1000 Hz, 500 ms), the sender delivered a one-way verbal statement. The first sentence was required to reiterate that the recommended option was "more beneficial" for the receiver, after which the sender could freely provide task-relevant information. During this phase, the receiver remained silent and was not permitted to ask questions. A second auditory cue signaled the sender to immediately stop speaking.

We appreciate the reviewer's attention to consistency in methodological details. This comment has helped us further refine the precision and clarity of the task description.

---

(12) Were participants instructed as to the nature of the task? What were they told, how was comprehension checked, and to what extent were participants "fully informed" as to the incentives

and structure of the task?

Re: We thank the reviewer for this important comment. In the revised manuscript, we have provided a clearer and more systematic account of the instructions delivered to participants, the comprehension checks implemented prior to the task, and the incentive structure, in order to ensure methodological transparency and precision.

First, before the experiment began, all participants received standardized task instructions. These instructions explicitly covered: (1) role assignment procedures (sender (cheater) and receiver (detector) roles were randomly assigned and remained fixed throughout the experiment); (2) the conflict-of-interest payoff structure (any option that was more advantageous for the cheater was necessarily less advantageous for the detector, and vice versa); (3) the payment calculation method (final compensation consisted of a fixed payment of 15 RMB plus the gain obtained from one randomly selected gain trial minus the loss incurred in one randomly selected loss trial); and (4) the information structure (only the cheater could view the complete outcome matrix, the detector could not access the numerical values, and the cheater could not observe the detector's final choice).

Please see the revised manuscript, page 9, line 175 to page 10, line 191, or the highlighted blue text:

Prior to the experiment, experimenters confirmed that members within each dyad had no prior acquaintance and were meeting for the first time. To further validate the effectiveness of the stranger-dyad manipulation, participants completed the single-item Inclusion of Other in the Self (IOS) scale to assess perceived interpersonal closeness (Likert 7 -point scale, range from 1 to 9). All participants reported IOS scores below 2 (M=1.04, SD=0.21), indicating successful manipulation of stranger relationships. The study employed a sender–receiver spontaneous deception paradigm adapted from Chen et al. (2020). Roles (sender/cheater vs. receiver/detector) were randomly assigned within each dyad and remained fixed throughout the experiment. The protocol was approved by the local Medical Ethics Committee (No. 2023159), and written informed consent was obtained from all participants.

Participant compensation consisted of two components. Each participant received a fixed base payment of 15 RMB upon completion of the experiment. To enhance the ecological validity of gain and loss contexts, one trial was randomly selected from each context (gain and loss) at the end of the task to determine performance-based monetary rewards or penalties. Final payment was calculated as $15RMB+R_{gain}-R_{loss}$, yielding a total compensation range of 0-45RMB. All experimental procedures and payment rules were clearly explained to participants prior to the experiment, and no participant was required to incur any additional costs.

Participants were explicitly informed that each trial carried real monetary consequences and that misleading or deceptive communication was permitted within the task. Second, to ensure that participants fully understood the task structure, we implemented a dedicated comprehension check and practice phase. During practice, the experimenter

verbally reconfirmed participants' understanding of the payoff conflict rule, role-specific information access, and reward calculation procedures. Any misunderstandings were clarified before proceeding to the formal experiment. Thus, formal data collection commenced only after both members of the dyad confirmed that they understood the task. Regarding the reviewer's concern about whether participants were "fully informed," we would like to clarify two distinct levels. At the institutional level, participants were fully informed about the incentive structure, payment rules, role assignment, information availability, and the permissibility of deception within the task. At the trial level, however, participants did not know the counterpart's specific decision or whether deception had been successful in a given trial. This information asymmetry was an intentional component of the experimental design rather than a deficiency in informed consent. In other words, participants were fully informed about the structural rules of the task, while uncertainty regarding trial-specific outcomes was deliberately maintained as a core condition for eliciting deceptive behavior.

Please see the revised manuscript, page 13, line 252 to page 14, line 270, or the highlighted blue text:

In each trial, the payoff structure was explicitly designed to induce a direct conflict of interest between the sender and receiver: a higher payoff for the sender necessarily corresponded to a lower payoff for the receiver, and vice versa. This structure was identical in both gain and loss contexts. Importantly, only one option in each trial was objectively advantageous for the receiver (i.e., higher gain or lower loss), whereas the alternative option was disadvantageous. This design has been shown to effectively elicit spontaneous information manipulation based on immediate payoff trade-offs (Chen et al., 2020). Participants were explicitly informed that deceptive or misleading communication was permitted in the task. Receivers could not view the numerical values in the outcome matrix and relied solely on the sender's recommendation cues (color signals and verbal statements), rendering direct verification of information impossible (Huang et al., 2025). Meanwhile, senders received no feedback during or after the experiment regarding whether their information successfully influenced the receiver's decision. This design eliminated explicit punishment, feedback, or learning mechanisms, ensuring that task performance primarily reflected real-time information weighting and decision-making under uncertainty rather than strategic learning or reputation management. Because the semantic content of verbal statements was not constrained, deception could not be conclusively identified at the single-trial level. Accordingly, deception was operationally defined based on whether the sender's recommendation was objectively disadvantageous for the receiver given the payoff structure, rather than on direct inference of the sender's subjective intent.

We appreciate the reviewer's attention to task transparency and ethical clarity. The relevant clarifications have been incorporated into the Participants and Task Procedure sections of the revised manuscript to enhance methodological completeness.

(13) *Given the uncontrolled nature of the task (there is a 15s period in which senders can verbally talk with receivers, if I'm understanding right), the use of "deception" here seems potentially problematic. In other words, while the recommendation of one option or the other might be characterized clearly as "sender-advantageous" or "receiver-advantageous", the presence of DECEPTION can only be inferred if we know what the sender said alongside that recommendation. A sender could for example (and quite possibly did!) say that they would alternate recommendations for maximum fairness, entailing zero actual deception. The authors should discuss the limits on their ability to conclusively identify the presence of deception in this task (it is difficult if not impossible to do so) and consider reframing the paper in terms of receiver-advantageous and -disadvantageous recommendations.*

Re: We thank the reviewer for this thoughtful suggestion and critical observation. We fully appreciate the caution regarding the use of the term "deception," particularly given the 15-second free verbal phase. As noted, the mere fact that a recommendation was objectively disadvantageous to the detector does not, at the subjective level, allow us to determine with absolute certainty whether the cheater possessed a deliberate intention to deceive. In the revised manuscript, we have provided a clearer conceptual delineation and theoretical clarification of this issue.

First, in the Methods section, we have strengthened the operational definition of deception. Specifically, we clarify that "deception" in the present study is not inferred from the semantic content of verbal statements, but is operationalized based on the objective payoff structure. We agree that subjective deceptive intent cannot be directly verified at the single-trial level, and we have explicitly acknowledged this limitation in the revised manuscript.

Please see the revised manuscript, page 13, line 266 to page 14, line 270, or the highlighted blue text:

Because the semantic content of verbal statements was not constrained, deception could not be conclusively identified at the single-trial level. Accordingly, deception was operationally defined based on whether the sender's recommendation was objectively disadvantageous for the receiver given the payoff structure, rather than on direct inference of the sender's subjective intent.

Second, regarding the scenario raised by the reviewer—for example, a sender stating during the verbal phase, "I will alternate recommendations to maintain fairness"—we contend that such a strategy still constitutes strategic communication within an asymmetric information and payoff-conflict structure. Because the detector cannot access the true outcome matrix, any recommendation that is inconsistent with the objectively advantageous option structurally carries the potential to mislead. Moreover, participants were paired as stranger dyads. Under conditions

of mutual unfamiliarity, explicit payoff conflict, and a prohibition on detector verbal responses, it is highly unlikely that dyads could form prior agreements or coordination strategies during the task. Consistent with this institutional framework, our behavioral analyses showed that most dyads' cheaters more frequently chose disadvantageous recommendations. Thus, within this experimental structure, when a cheater recommends an option that is objectively disadvantageous to the detector, such behavior constitutes a potentially misleading strategy in a game-theoretic sense. Our analyses focus on this structural form of misleading choice rather than on moral evaluations of verbal content.

Please see the revised manuscript, page 13, lines 252 to 256, or the highlighted blue text:

In each trial, the payoff structure was explicitly designed to induce a direct conflict of interest between the sender and receiver: a higher payoff for the sender necessarily corresponded to a lower payoff for the receiver, and vice versa. This structure was identical in both gain and loss contexts. Importantly, only one option in each trial was objectively advantageous for the receiver (i.e., higher gain or lower loss), whereas the alternative option was disadvantageous.

At the same time, we recognize the importance of conceptual precision. Accordingly, in the revised manuscript we have refined our terminology at multiple key locations. We now employ expressions such as "objectively disadvantageous recommendation" to clarify the operational criterion and emphasize that we examine "strategic information manipulation under payoff conflict," rather than attempting to infer subjective lying intent. Finally, we carefully considered whether to reframe the manuscript entirely in terms of "receiver-advantageous versus receiver-disadvantageous recommendations." However, completely abandoning the term "deception" could weaken the theoretical continuity between the present study and prior work on the neural mechanisms of deception (e.g., Chen et al., 2020; Huang et al., 2025). Instead, in the revised manuscript we more clearly distinguish between the operational definition of deception and the non-observability of subjective intent, and we explicitly emphasize the structural meaning of the term-namely, recommending an option that is objectively disadvantageous to the counterpart. This approach preserves theoretical continuity while directly addressing concerns regarding conceptual rigor.

We sincerely appreciate the reviewer's attention to this core issue. This comment has prompted us to more carefully define the conceptual boundaries of our terminology and has substantially strengthened the clarity and methodological transparency of the manuscript.

Reference

[1] Chen, M., Zhang, T., Zhang, R., ..., & Li, X. (2020). Neural alignment during face-to-face spontaneous deception: does gender make a difference? *Human brain mapping*, *41*(17), 4964-4981.

[2] Huang, R., Gao, X., Zhang, C., …, & Liu, Y. (2025). Forewarned Is Forearmed: The single- and dual-brain

mechanisms in Detectors from Dyads of Varying Social Distance During Deceptive Outcomes Evaluation. The *Journal of neuroscience*, *45*(43): e2129242025.

---

(14) Degrees of freedom must be reported with statistical tests. None are reported.

Re: We acknowledge that, in the original manuscript, some statistical results were reported without complete degrees of freedom (df). This omission does not meet current journal standards for statistical transparency and reproducibility, and we sincerely apologize for this oversight.

In the revised manuscript, we have systematically re-examined all reported statistical tests and have supplemented complete degrees of freedom information for every t-test, analysis of variance (ANOVA), regression analysis, and correlation analysis. For example, paired-samples t-tests are now consistently reported in the format t(df) = value; repeated-measures ANOVAs are reported as $F(df_1, df_2)$ = value; and hierarchical regression analyses and model comparisons now include explicit degrees of freedom and corresponding sample size information. All statistical indices have been formatted uniformly in accordance with APA guidelines and journal recommendations, and are aligned with the final analyzed sample after exclusions. In addition, we carefully verified that any changes in sample size (e.g., due to the exclusion of individual dyads) were accurately reflected in the calculation of degrees of freedom, ensuring that all reported df values strictly correspond to the final sample included in each analysis. Through these revisions, we have strengthened the completeness, accuracy, and transparency of our statistical reporting.

We again thank the reviewer for carefully scrutinizing these statistical details. This comment has substantially improved the methodological rigor and reporting quality of the manuscript.

---

(15) The authors state they exclude people who did not "deceive" enough – but then do not state how many pairs were excluded (which might be expected to significantly impact statistical power).

Re: We thank the reviewer for pointing out this important issue regarding insufficient disclosure of exclusion information. Failure to clearly report the number of excluded dyads could indeed raise reasonable concerns about statistical power and potential sample selection bias. In the original manuscript, we did not explicitly state the number of excluded dyads in the relevant section, and we acknowledge this as an oversight. In the revised manuscript (Participants section), we have now clearly specified both the number of excluded dyads and the reasons for

exclusion, and have ensured that this information is fully consistent with the reported power analysis.

Please see the revised manuscript, page 9 , lines 170 to 175 , or the highlighted blue text.

Initially, 37 same-gender dyads composed of previously unacquainted individuals were formed from eligible participants. One dyad was excluded due to insufficient fNIRS data quality, and two dyads were excluded based on a predefined high-honesty criterion (deception rate<85%; Chen et al., 2020). The final sample therefore consisted of 34 same-gender stranger dyads, including 18 female dyads (age: *M*=21.33, *SD*=2.07) and 16 male dyads (age: *M*=20.56, *SD*=1.29).

Specifically, of the initial 37 dyads recruited, only two dyads were excluded due to insufficient deception rates. Importantly, an a priori power analysis was conducted using G*Power prior to data collection, which indicated a minimum required sample size of 34 dyads. The final analyzed sample consisted of exactly 34 dyads. Therefore, the exclusion of these two dyads did not reduce statistical power below the predetermined threshold.

We sincerely appreciate the reviewer's attention to this important detail.

---

(16) There were 48 trials of deception in gain contexts and 48 in loss, but there is no behavioral analysis of those trials. Literally the only report is the aggregate number of "deception" trials as a function of gain or loss contexts. This is simply not sufficient from a behavioral analysis perspective for a peer reviewed study. There are no reaction times, no trial-level analyses (for example of how sender's recommendations affected receiver's choices), no examinations of block differences or trends, no consideration of patterns in choices, or individual differences/variation in behavior. Literally a single test is performed – a paired t-test comparing how many trials are "deceptive" in the gain frame and how many in the loss frame.

Re: We sincerely thank the reviewer for this thoughtful and professional critique regarding the depth of our behavioral analyses. We fully agree that reporting only the overall difference in deception frequency across contexts would be insufficient to meet the behavioral rigor expected in a peer-reviewed study. This comment has been highly valuable in strengthening the structure and analytical completeness of the manuscript. In the revised version, we have systematically expanded the behavioral analyses across multiple levels.

First, we added reaction time (RT) analyses for senders (cheaters). Specifically, we conducted a 2 (context: gain vs. loss)×2 (behavior type: honesty vs. deception) repeated-measures ANOVA on RTs during the sender's initial decision phase. The results revealed a significant main effect of context and a significant interaction effect. Decisions were made more quickly in the loss context, and within the loss context, deceptive responses were significantly faster than honest responses (see revised manuscript, *Behavioral Results: Reaction Time (RT)*). These findings indicate that

gain–loss framing not only alters the frequency of deception but also modulates the temporal dynamics of decision implementation.

Second, we incorporated behavioral analyses for receivers (detectors), rather than focusing solely on cheaters. We operationally defined "recommendation adoption" as whether the detector selected the option recommended by the cheater and compared successful deception rates across contexts. In addition, we conducted a 2 (context)×2 (recommendation adoption) repeated-measures ANOVA on RTs during the detector's final decision phase. Significant main and interaction effects were observed, indicating that in the loss context, detectors made faster decisions when adopting the recommendation. This analysis directly addresses how cheater recommendations influence detector choice behavior.

In summary, the revised behavioral results section now includes analyses of deception rates and RTs for cheaters, as well as recommendation adoption rates and RTs for detectors. The behavioral findings no longer rely solely on a single paired-samples t-test but instead characterize dyadic behavior across multiple dimensions, including frequency, response latency, and interactive influence under different contextual frames.

Please see the revised manuscript, page 26, lines 498 to page 29, line 562, or the highlighted blue text:

Behavioral Results: Deception Rate

The present study focuses on how cheaters weigh potential outcomes and decide whether to deceive or act honestly under conditions of conflicting interests. Accordingly, dyads that almost never engaged in objectively disadvantageous recommendations toward the detector were excluded from behavioral and neural analyses. Specifically, two dyads with honesty rates exceeding 85% across the experiment were removed. Dyads characterized by persistently honest recommendations primarily reflect stable moral compliance or risk-avoidant tendencies, rather than engagement in active deceptive interaction. Such behavioral patterns do not permit meaningful modeling of the psychological processes underlying deception. This exclusion criterion was predefined prior to data analysis and applied uniformly across all conditions.

To examine potential gender effects on cheater behavior, we conducted a 2 (Gender: male vs. female)×2 (Behavior type: honesty vs. deception) mixed-design ANOVA. A significant main effect of behavior type was observed[$F_{(1,32)}$ =12878.747, p<0.001, η_p^2=0.998, **Figure 2A**], indicating that deception (0.92±0.02) occurred substantially more frequently than honesty (0.08±0.02). No significant main effects of gender or interaction effects were detected (ps>0.329). These findings suggest that the experimental paradigm effectively elicited deception behavior without gender differences. Subsequent analyses focused exclusively on deceptive trials for two reasons: **(1)** the primary objective of this study was to investigate the behavioral and neural mechanisms underlying active deception interaction; (2) honesty trials were too sparse to support reliable statistical modeling (female: 8.16%; male: 7.42%). A paired-samples t test comparing deception rates across contexts revealed that cheaters engaged in

deception more frequently under loss than gain contexts [loss vs. gain: 0.94±0.03 vs. 0.90±0.03), $t_{(33)}$=-4.626; p<0.001, *Cohen'd*=-0.793, **Figure 2B**].

Beyond sender behavior, we examined detectors' trial-level acceptance of the cheater's recommendation. Detector acceptance was operationally defined as selecting the recommended option during the final decision phase (i.e. successful deception). A paired-samples *t* test showed that successful deception rates were significantly higher under loss than gain contexts [loss vs. gain: 0.92±0.07 vs. 0.82±0.09, $t_{(33)}$=-5.304; p<0.001, *Cohen'd*=-0.910, **Figure 2C**], indicating that detectors were more likely to adopt the cheater's recommendation in loss contexts.

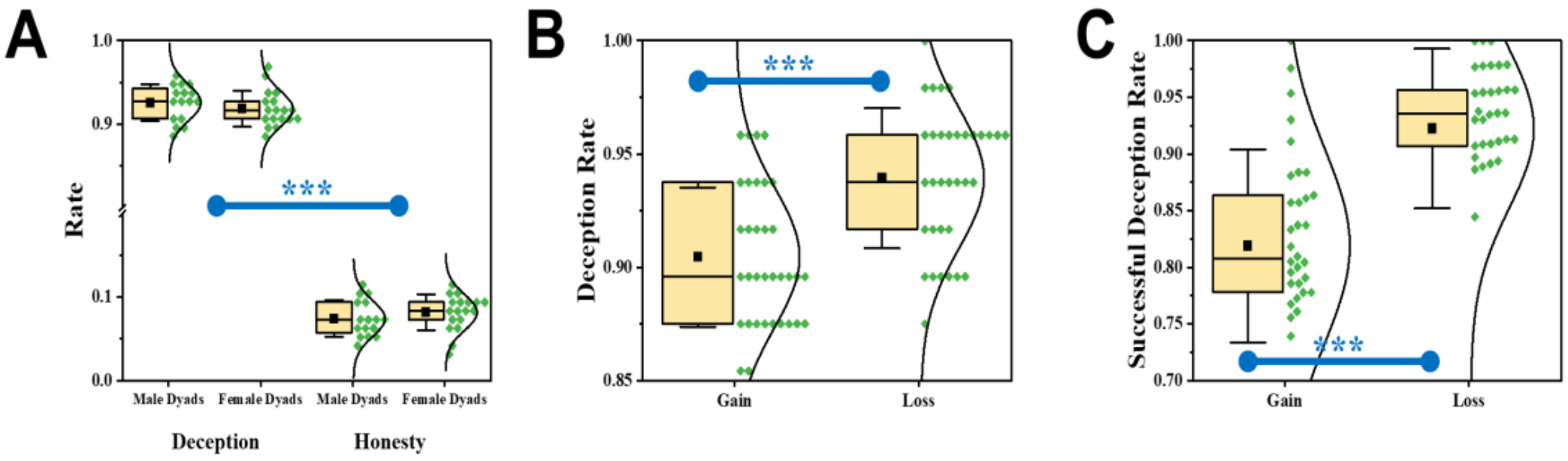

Figure 2. Behavioral results of deception. (A) Proportion of behavioral occurrence (honesty vs. deception). **(B)** Deception rate across gain and loss contexts. **(C)** Successful deception rate across gain and loss contexts. Green diamonds represent individual data points; black squares denote condition means; lines within boxes indicate medians; error bars represent ±1SD; outer contours depict normal distribution curves. *** p< 0.001. The same conventions apply in subsequent figures.

Behavioral Results: Reaction Time (RT)

To examine theRT underlying cheaters' first decisions (i.e., the first behavioral expression of deception versus honesty), a 2 (Context: gain vs. loss)×2 (Behavior type: honesty vs. deception) repeated-measures ANOVA was conducted (**Figure 3A**). A significant main effect of contextual framing was observed[loss vs. gain: 3144.11±139.16 vs. 3541.29±174.11, $F_{(1,33)}$=35.132, p<0.001, η_p^2=0.516], indicating that RT were significantly shorter under loss compared to gain contexts. A significant interaction between context and behavior type also emerged [$F_{(1,33)}$=5.0422, p=0.032, η_p^2 =0.133]. Simple-effects analyses revealed that under loss contexts, deception were executed significantly faster than honesty (deception vs. honesty: 3047.40±127.74 vs. 3240.82±159.51; post hoc p= 0.018, *95% CI* [−351.727, −35.111]). Contrastly, under gain contexts, no significant difference was observed between deceptive and honest responses (deception vs. honesty: 3589.68 ± 187.97vs. 3492.91±165.10; post hoc p= 0.132, *95% CI* [−30.630, 224.161]). No significant main effect of behavior type was detected [deception vs. honesty: 3318.54±151.49 vs. 3366.87±157.77, $F_{(1,33)}$=2.876, p=0.099, η_p^2=0.080].

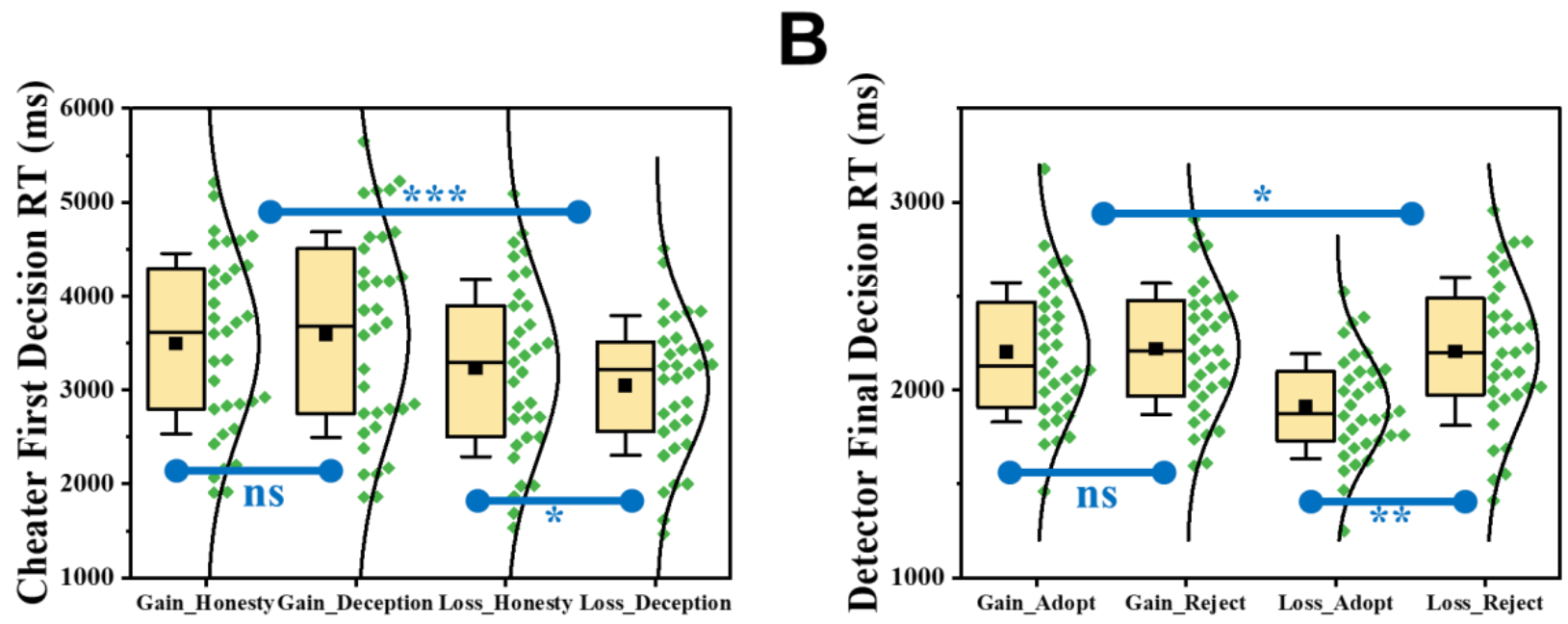

Figure 3. RT results. (A) Cheater RT at the first decision phase. **(B)** Detector RT at the final decision phase . ***p*< 0.01, **p*< 0.05, ns= non-significant. The same conventions apply in subsequent figures.

We further examined detectors' RT during the final decision phase to characterize their decision-making dynamics under different contextual and recommendation conditions. A 2 (context: gain vs. loss)×2 (recommendation adoption: adopted vs. rejected) repeated-measures ANOVA revealed a significant main effect of context, with overall shorter RT in loss compared to gain contexts [loss vs. gain: 2058.96±38.67 vs. 2209.68±46.18, $F_{(1,33)}$=5.125, p=0.030, η_p^2=0.134]. A significant main effect of recommendation adoption was also observed, indicating that RT were shorter when detectors adopted the cheater's recommendation (2056.82±39.34) than when they rejected (2211.82±43.87) it [$F_{(1,33)}$=5.829, p=0.021, η_p^2=0.150]. Importantly, the interaction between context and recommendation adoption was significant [$F_{(1,33)}$=6.024, p=0.020, η_p^2=0.154]. Simple-effects analyses showed that in loss contexts, RT were significantly shorter when recommendations were adopted compared to rejected (adopted vs. rejected: 1912.96±47.80 vs. 2204.96±67.52, post hoc p=0.002, *95%CI* [113.406, -470.599]). No significant difference between adoption (2200.68 ± 63.69) and rejection (2218.68± 59.93) was observed in gain contexts (post hoc p= 0.828, *95% CI* [−149.387, 185.398]). These results are illustrated in **Figure 3B**.

We are sincerely grateful to the reviewer for raising this critical point. This comment prompted us to substantially strengthen the behavioral analysis section, resulting in a more comprehensive manuscript.

---

(17) The brain area identified as DLPFC in Figure 3 I think would be widely characterized as frontopolar or anterior prefrontal cortex, but I would confidently say that it is certainly not dorsal OR lateral.

Re: We sincerely thank the reviewer for this professional and insightful comment regarding anatomical localization. We fully understand your concern about the classification of the region labeled as "DLPFC (CH14)" in Figure 3 (now Figure 4), and we agree that this issue requires careful clarification.

First, with respect to channel spatial localization, the optode positions in the present study were not determined by visual estimation. Instead, they were obtained using a 3D digitizer to record actual scalp coordinates, followed

by MNI space registration using NIRS-SPM. According to the resulting MNI coordinates, CH14 was located in the dorsolateral portion of the middle frontal gyrus, corresponding to the spatial range of Brodmann areas 9/46. On this basis, we initially classified it as dorsolateral prefrontal cortex (DLPFC).

Please see the revised manuscript, page 16, lines 305 to 308, or the highlighted blue text:

Channel localization data were collected using a 3D digitizer, and spatial alignment of NIRS_SPM provided the cortical location of the channels (**Table 1**).

Table 1. Anatomical correspondence between fNIRS channels and PFC.

**Anatomical label**	**Channels**
Dorsolateral prefrontal cortex	1、2、6、7、9、14
Frontopolar area	3、4、5、10、11、12、13
Ventrolateral prefrontal cortex	8、15、16、22
Orbitofrontal area	17、18、19、20、21

Second, regarding the visualization in the figure, we used BrainNet Viewer for surface projection. It is important to note that BrainNet employs spherical interpolation algorithms when projecting statistical results onto a standard brain surface. The displayed color patches therefore represent the projected spatial extent of statistically significant channels on the template surface, rather than true functional voxels or precise cortical boundaries (Li et al., 2016; Xia et al., 2013). Under certain viewing angles—particularly when using inflated brain surfaces or lower spatial resolution—the interpolated color spread may visually appear closer to the frontopolar cortex (BA10). This apparent shift reflects a limitation of surface rendering and visualization, rather than an actual mislocalization of the channel.

Third, from the perspective of physical optode coverage, the 2×8 probe array was positioned with reference to Fpz, with a source-detector distance of 3 cm. According to standard 10–20 system mapping, this configuration primarily covers the DLPFC, frontopolar area, VLPFC, and OFC (see Table 1). CH14 is situated in the dorsolateral portion of the array rather than at the most anterior frontopolar position. Thus, at the level of physical probe placement, its location is more consistent with a lateral dorsolateral region than with a purely frontopolar region.

Nevertheless, we have carefully considered the possibility of anatomical classification ambiguity raised in your comment. Given the limited spatial resolution of fNIRS (approximately 1–3 cm) and potential inter-individual variability in cortical folding, the boundary between BA9/46 and BA10 may overlap across participants. Accordingly, in the revised manuscript, we have adopted more cautious terminology. In both the Results and Discussion sections, we now describe this region as a "control-related lateral PFC region centered on CH14" to avoid over-specific anatomical labeling.

Please refer to the revised Figure 4 (page 30, lines 579 to 584) for the updated activation map reflecting these revisions.

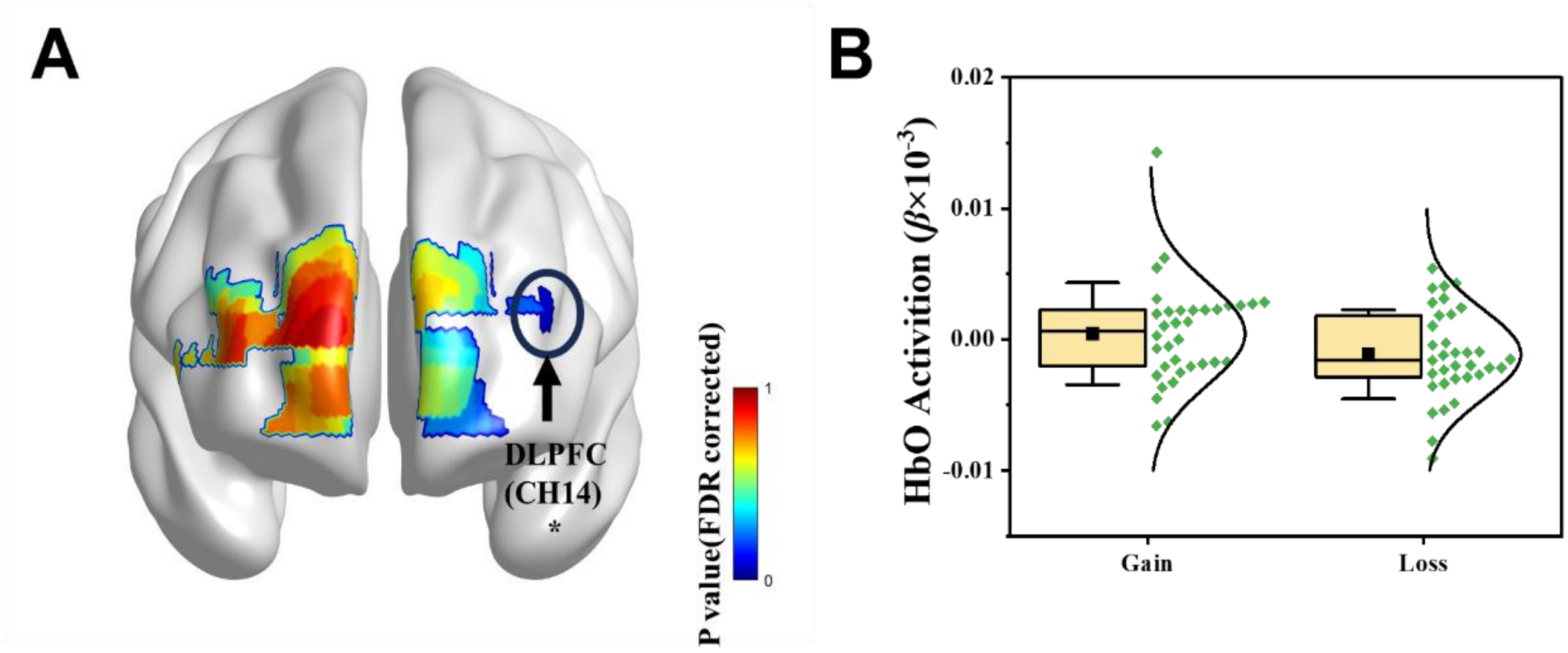

We sincerely appreciate your attention to this important detail. Your comment has prompted us to more clearly distinguish between spatial localization results and visualization artifacts, and has improved the precision and transparency of our anatomical terminology.

Reference

[1] Xia, M., Wang, J., & He, Y. (2013). BrainNet Viewer: A Network Visualization Tool for Human Brain Connectomics. *PLoS ONE*, *8*(7), e68910.

[2] Li, L., Cazzell, M., Babawale, O., & Liu, H. (2016). Automated voxel classification used with atlas-guided diffuse optical tomography for assessment of functional brain networks in young and older adults. *Neurophotonics*, *3*(4), 045002.

---

(18) Given the presence of significant movement (participants are simply seated and talking with each other in an unrestrained setting), could the authors comment on how much of the fNIRS signal might be related to motion or motor movement, either at the gross level (whole body shifting) or at the local level (facial expressions)?

Re: We thank the reviewer for raising this important methodological concern. In a face-to-face paradigm that allows natural verbal interaction, head movements, postural adjustments, and facial expressions may indeed introduce potential confounds into fNIRS signals. Accordingly, we implemented systematic control and correction procedures at the levels of experimental design, data acquisition, and preprocessing to minimize the influence of motion artifacts.

First, at the level of experimental implementation, although participants engaged in a naturalistic interaction, we imposed structured constraints to reduce excessive movement. During the 60-s baseline period, participants were explicitly instructed to remain relaxed and minimize head motion. In the formal task, the sender's verbal statement

was strictly limited to 15 s and precisely controlled by auditory cues marking onset and offset. During this phase, the receiver was instructed to remain silent and avoid initiating overt bodily interaction. This semi-structured interaction paradigm preserved ecological validity while reducing the likelihood of large-amplitude body movements. Importantly, we independently coded direct eye contact (ICC= 0.826) and statistically examined its frequency across contexts. No significant contextual differences were observed (*ps*> 0.067), thereby ruling out systematic, context-driven differences in overt interaction-related movement at the behavioral level.

Please see the revised manuscript, page 12, lines 224 to 242, or the highlighted blue text:

Following completion of the pre-task subjective measures, participants entered a preparation phase, which began with a 60-s baseline recording. During this period, participants were instructed to fixate on a central "+" symbol, remain relaxed, and minimize head movements (Dai et al., 2018). Participants then completed a practice phase comprising four trials, including two randomly selected outcome matrices from the gain context and two from the loss context. The formal experiment commenced only after both members of the dyad demonstrated a clear understanding of the task rules, role assignments, and payoff structure. The procedure of the formal experiment was identical to that of the practice phase and consisted of the following stages (see **Figure 1A**): **(1) Outcome matrix presentation (8s).** The sender (potential information manipulator) viewed an outcome matrix displaying the potential outcomes for both players. The first and second rows corresponded to the sender's and receiver's outcomes, respectively. Red and blue colors denoted Option A and Option L. **(2) Recommendation cue (1s).** The sender conveyed a brief nonverbal recommendation to the receiver using a color cue (red or blue), semantically equivalent to "the option associated with this color is more beneficial for you in this round." **(3) Verbal statement (15 s).** Following an auditory cue (1000 Hz, 500 ms), the sender delivered a one-way verbal statement. The first sentence was required to reiterate that the recommended option was "more beneficial" for the receiver, after which the sender could freely provide task-relevant information. During this phase, the receiver remained silent and was not permitted to ask questions. A second auditory cue signaled the sender to immediately stop speaking.

Second, at the preprocessing stage, we applied a multi-step motion artifact correction pipeline. This preprocessing framework, widely adopted in fNIRS studies involving natural social interaction (e.g., Zhao et al., 2023; Pan et al., 2018), includes procedures specifically designed to attenuate motion-related and muscle-related contamination in HbO signals. These steps substantially reduce the likelihood that observed neural effects are driven by gross head movement or facial muscle artifacts.

Please see the revised manuscript, page 18, line 352 to page 19, line 363, or the highlighted blue text:

Preprocessing. fNIRS data were preprocessed in MATLAB using the Homer2 toolbox. Low-quality channels were identified and excluded using the enPruneChannels function. Dyads were removed from further analyses if the proportion of bad channels exceeded 50%, in accordance with established criteria (Zhao et al., 2023). The steps

include (1) it detects motion artifacts using the hmrMotionArtifactByChannel function (Tak & Ye, 2014). (2) Remove the initial 60 seconds of the signal to eliminate environmental variations. (3) Apply principal component analysis (PCA) to separate task-specific fNIRS signals (Pan et al., 2018). (4) Convert raw light intensity data to optical density (OD) data. (5) Kurtosis-based wavelet filtering to remove motion artifacts with a threshold of 3.3 (Hein & Rieger, 2023). (6) A 5th order Butterworth bandpass filter with cutoff frequencies of 0.01 Hz and 0.5 Hz was used to remove signal drift and noise (Gagnon et al., 2011). (7) OD data were converted to hemoglobin oxygenation concentration using BeerLambert's law for further analysis (Strangman et al., 2002).

Third, the pattern of results further argues against a motion-based explanation. If ΔINS were primarily driven by shared movement or synchronized facial expressions, one would expect comparable synchrony increases across all interaction conditions, including honest and failed-deception trials. However, the observed contextual difference was specific to bilateral DLPFC–DLPFC ΔINS during successful deception, with no analogous effects in honest or failed-deception trials. Such condition specificity is inconsistent with a pure motion-artifact account, as motor activity would not plausibly increase selectively only during successful deception outcomes.

Please see the revised manuscript, page 32, line 613 to page 33, line 628, or the highlighted blue text:

During successful deception trials, ΔINS between bilateral DLPFC channels (CH14–CH14) was significantly modulated by context. Specifically, ΔINS was significantly higher in the loss context compared to the gain context [loss vs. gain: 0.44±0.12 vs. 0.22±0.12, $t_{(33)}$=-8.656, p<0.001, *Cohen's d*=-1.485, **Figure 6A&B**]. Importantly, no significant contextual differences were observed in the same channel pair during honesty trials [loss vs. gain: 0.07±0.05 vs. 0.07±0.04, $t_{(33)}$=0.054, p=0.957, *Cohen's d*=-0.055, **Figure 6D**] or failed deception trials [loss vs. gain: 0.22±0.11 vs. 0.22±0.09, $t_{(33)}$=-0.320, p=0.751, *Cohen's d*=0.009, **Figure 6C**]. These results indicate that framing-related modulation of ΔINS was specific to successful deception. Permutation testing (1,000 iterations) confirmed that the empirical t-value exceeded the null distribution (ps < 0.001, **Figure 6E**), indicating that the effect was not driven by random pairing.

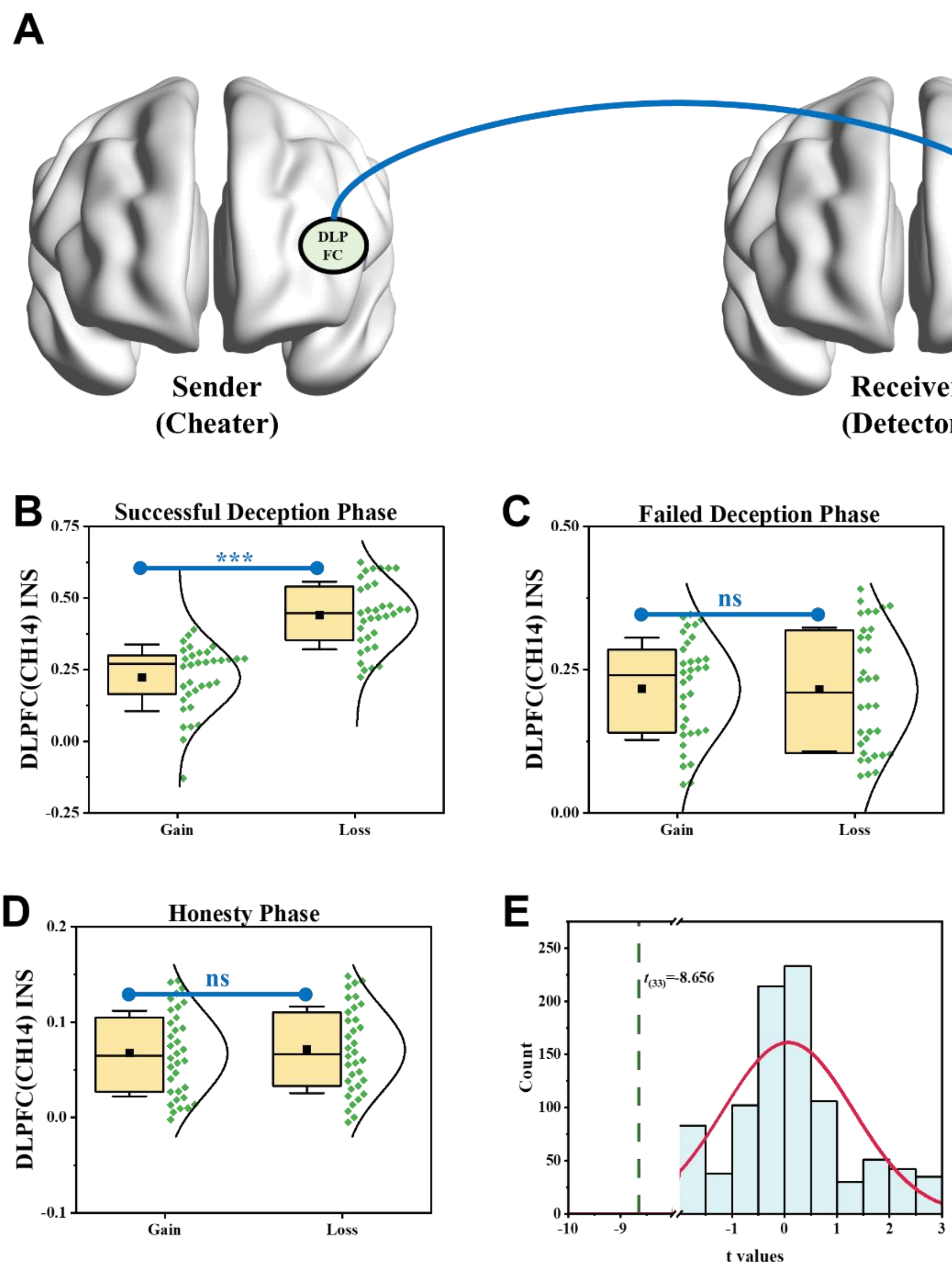

Figure 6. Task-Level ΔINS Results. (A) Visualization of channel pairs exhibiting significant contextual differences in ΔINS. **(B)** Comparison of ΔINS in channel pairs showing significant contextual differences during successful deception trials. **(C)** Comparison of ΔINS in the same channel pair during failed deception trials across contexts. **(D)** Comparison of ΔINS in the same channel pair during honesty trials across contexts.

At the same time, we acknowledge that in natural language interaction paradigms, it is impossible to completely exclude the potential contribution of subtle perioral muscle activity to signals recorded from anterior frontal regions. This represents an inherent limitation of ecological fNIRS designs. Accordingly, in the revised Discussion, we explicitly note that although rigorous motion correction procedures were applied and condition-specific neural patterns were observed, future studies could incorporate short-separation channels or simultaneous EMG recordings to further dissociate superficial muscle activity from cortical hemodynamic signals.

Please see the revised manuscript, page 49, lines 864 to 868, or the highlighted blue text:

Secondly, despite employing multi-step preprocessing to control for motion artifacts, fNIRS signals in naturalistic interactions may still be influenced by speech-related muscle activity and head motion. Therefore, interpretations of prefrontal activation and INS should be viewed as inferences of functional patterns in ecological contexts rather than direct localization of fine-grained neural circuits.

Reference

[1] Dai B, et al. (2018) Neural mechanisms for selectively tuning into the target speaker in a naturalistic noisy situation. *Nature Communication*, *9*, 2405.

[2] Zhao, H., Zhang, C., Tao, R., Duan, H., & Xu, S. (2023). Distinct inter-brain synchronization patterns underlying group decision-making under uncertainty with partners in different interpersonal relationships. *NeuroImage*, *272*(120043), 10-1016.

[3] Tak, S., & Ye, J. C. (2014). Statistical analysis of fNIRS data: A comprehensive review. *NeuroImage*, *85*, 72-91.

[4] Pan, Y., Novembre, G., Song, B., Li, X., & Hu, Y. (2018). Interpersonal synchronization of inferior frontal cortices tracks social interactive learning of a song. *NeuroImage*, *183*, 280-290.

[5] Hein, A., & Rieger, J. W. (2023). Improved motion artifact correction in fNIRS data by combining wavelet and correlation-based signal improvement. *Sensors*, *23*(8), 3979.

[6] Gagnon, L., Perdue, K. L., Greve, D. N., Goldenholz, D., Kaskhedikar, G., & Boas, D. A. (2011). Improved recovery of the hemodynamic response in diffuse optical imaging using short optode separations and state-space modeling. *NeuroImage*, *56*(3), 1362-1371.

[7] Strangman, G., Culver, J. P., Thompson, J. H., & Boas, D. A. (2002). A quantitative comparison of simultaneous BOLD fMRI and NIRS recordings during functional brain activation. *NeuroImage*, *17*(2), 719-731.

---

(19) If I understand the LSR ML analysis, the analysis was carried out at the participant, not trial, level? That would mean that the authors asked if roughly 30 brain synchrony values could predict roughly 30 deception quantities. This is problematic in several respects. Figure 3 already demonstrates an average difference between gain and loss contexts; thus it follows that one could take that brain activity and turn around ask whether it predicts deception (it would, as deception varies across gain and loss contexts), making the analysis circular. The authors have a lot of trials (96 for each pair) which they should consider using in a nested fashion (across participants) to make a much stronger and more interesting trial-wise prediction.

Re: We thank the reviewer for this critical comment regarding the structure of the LSR-based machine learning analyses. We fully understand the concern about whether prediction was conducted at the subject level and whether there might be a risk of circular inference. These are essential methodological issues that warrant explicit clarification.

First, regarding the level of analysis, the LSR prediction reported in the section "*Predicting Successful Deception from ΔINS: Comparative ML Analyses*" was conducted at the dyad level. Specifically, for each dyad, ΔINS values extracted from statistically significant channel pairs during successful deception were used as feature variables, and the overall successful deception rate served as the continuous prediction target. Model training and evaluation were performed under a LOOCV framework. Thus, this analysis directly addressed whether between-dyad differences in ΔINS predict overall successful deception performance across dyads.

However, we would like to clarify an important point in response to the concern regarding circularity and hierarchical structure. The predictive analysis did not regress on "gain vs. loss mean differences" per se. Although Figure 3 (now Figure 6 in the revised manuscript) illustrates average ΔINS differences between gain and loss contexts, the LSR model did not use contextual labels as inputs. Instead, it used each dyad's actual ΔINS values from significant channel pairs. Moreover, the prediction target—successful deception rate—varied substantially across dyads and was not reducible to a simple contextual main effect. Therefore, the analysis tested a genuine cross-dyad neural–behavioral covariation, rather than re-expressing a condition mean contrast. **The revised description of this analysis can be found on page 40, line 700 to page 42, line 723, or in the highlighted blue text in the revised manuscript.**

Predicting Successful Deception from ΔINS: Comparative ML Analyses

To further characterize the brain–behavior relationship, we implemented exploratory ML approaches. Based on the preceding findings, ΔINS during failed deception and single-brain activation measures neither correlated significantly with deception outcomes nor contributed incremental explanatory power in hierarchical regression models. We therefore hypothesized that ΔINS during successful deception constitutes the critical neural predictor of deceptive performance.

Table 11. Performance evaluation of different ML models.

Model	Condition	ROC-AUC	Accuracy	Precision	Recall	F1 Score	MCC
LSR	Gain	0.929	0.727	0.750	0.857	0.800	0.386
	Loss	0.867	0.727	0.800	0.667	0.727	0.467
Logistic Regression	Gain	0.893	0.909	0.875	0.796	0.733	0.810
	Loss	0.800	0.636	0.667	0.667	0.667	0.817
Naive Bayes	Gain	0.768	0.727	0.650	0.757	0.700	0.386
	Loss	0.733	0.727	0.800	0.667	0.727	0.467
DT	Gain	0.732	0.677	0.833	0.714	0.669	0.449
	Loss	0.817	0.818	0.833	0.833	0.833	0.633
SVR	Gain	0.821	0.818	0.857	0.857	0.610	0.607
	Loss	0.667	0.545	0.600	0.500	0.545	0.100

Separate predictive models were constructed for gain and loss contexts, with ΔINS during successful deception entered as input features and successful deception rate as the target variable. To identify the optimal predictive framework, we systematically compared multiple ML algorithms encompassing both linear and non-linear approaches. Across contexts, LSR demonstrated superior predictive performance[gain context: ROC-AUC=0.929, Accuracy=0.727, **Figure 10A**; loss context: ROC-AUC=0.973, Accuracy=0.933, **Figure 10B**]. Detailed performance metrics for all models are reported in **Table 11**. Using the LSR model, ΔINS within the significant channel pair reliably predicted successful deception across contexts (gain: R^2=0.870, *MSE*=0.001; loss: R^2=0.266, *MSE*=0.004). To assess statistical significance, we conducted 5,000 label-shuffled permutation tests. In both contexts, the observed predictive performance exceeded chance levels (gain: r=0.933, p<0.001, **Figure 10C**; loss: r=0.516, p<0.001, **Figure 10D**).

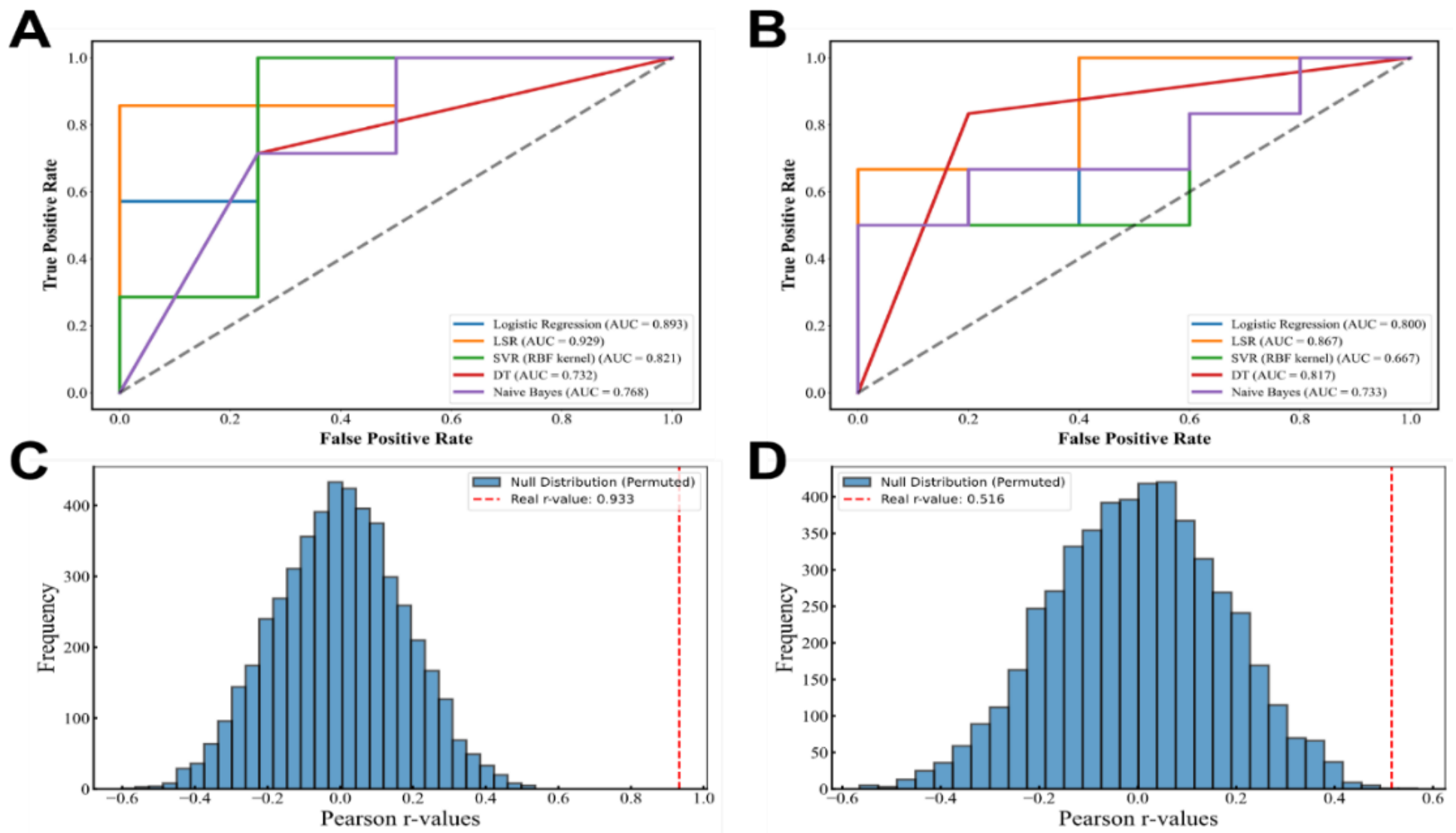

Figure 1. Comparative predictive performance of ML models across contexts. (A) ROC curves comparing ML models in the gain context. **(B)** ROC curves comparing ML models in the loss context. **(C)** Results of the 5,000-permutation test for predicting successful deception using ΔINS features in the gain context. **(D)** Results of the 5,000-permutation test for predicting successful deception using ΔINS features in the loss context.

At the same time, we found the reviewer's suggestion regarding trial-level prediction highly valuable. We agree that restricting prediction to the dyad level does not fully exploit the hierarchical structure of the 96 trials per dyad and may limit the depth of inference. Accordingly, in the revised manuscript, we have added trial-level ΔINS prediction analyses. In this additional analysis, ΔINS features were extracted at the single-trial level. Logistic regression and SVM models were then used to predict trial-level deception outcomes (successful vs. failed deception). Crucially, we implemented a leave-one-subject-out cross-validation scheme to prevent information leakage across individuals. This nested structure ensured that: (1) training and testing sets were independent at the subject level; (2) trial-level prediction did not rely on condition mean differences; and (3) model performance was evaluated against permutation-based null distributions (1,000 permutations).

The trial-level results demonstrated that, in both gain and loss contexts, logistic regression significantly outperformed chance (*p*s < 0.001), with prediction accuracy reaching 0.884 in the loss context. These findings indicate that ΔINS carries generalizable predictive information at the level of individual interactions, rather than merely reflecting contextual mean differences.

Please see the revised manuscript, page 33, line 629 to page 35, line 647, or the highlighted blue text:

Trial-Level ΔINS Predicts Deception Outcomes

To evaluate the predictive value of trial-level ΔINS during deceptive information processing, we applied two classification algorithms—Logistic Regression and SVM—to examine whether ΔINS could predict deception outcomes (successful vs. failed deception) at the trial level. Results indicated that, under both gain and loss contexts, the Logistic Regression model performed significantly above chance. Model performance was best in the loss context (Accuracy=0.884, AUC=0.611, F1 score=0.638). Performance metrics for all conditions and models are summarized in **Table 6** and **Figure 7**. To further assess the statistical robustness of model performance, we conducted 1,000 permutation tests. The Logistic Regression model significantly outperformed the null distribution generated by label shuffling (*p*s<0.001), confirming that the observed predictive accuracy was unlikely to arise by chance.

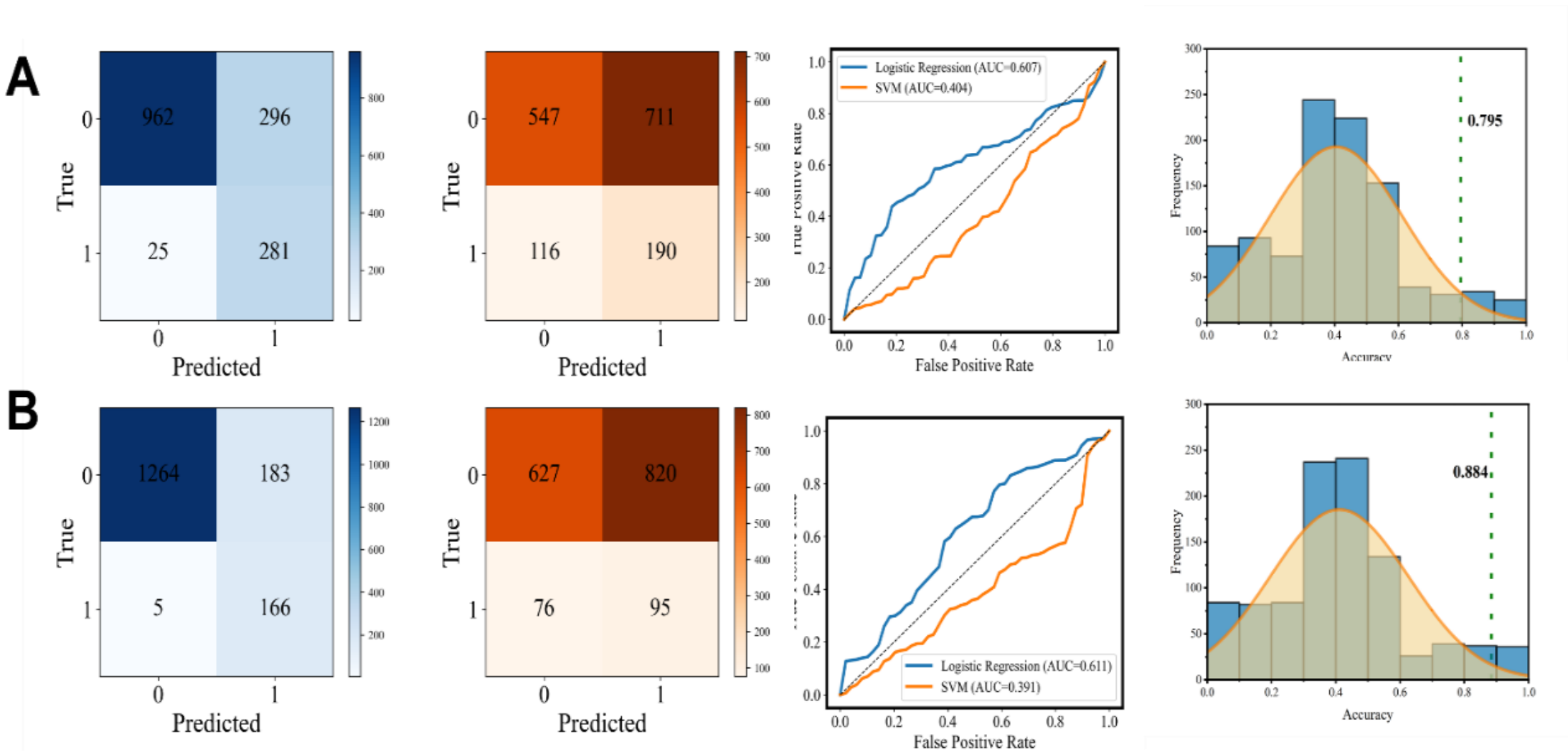

Figure 7. ML performance in predicting deception outcomes based on trial-level ΔINS. (A) Gain context; **(B)** Loss context. From left to right: **(1)** Confusion matrix for Logistic Regression (0=successful deception; 1=failed deception); **(2)** Confusion matrix for SVM; **(3)** ROC-AUC curves for both models (training phase), showing superior performance of Logistic Regression across contexts; **(4)** Permutation test results (1,000 iterations) for Logistic Regression, where the green dashed line indicates observed accuracy and the yellow curve represents the fitted null distribution.

Table 6. Performance evaluation of two ML algorithms across context.

Model	Condition	ROC-AUC	Accuracy	Precision	Recall	F1 Score
Logistic Regression	Gain	0.607	0.795	0.487	0.918	0.636
	Loss	0.611	0.884	0.476	0.971	0.638
SVM	Gain	0.404	0.471	0.211	0.621	0.315
	Loss	0.391	0.446	0.104	0.556	0.175

Thus, the revised analytical framework now explicitly incorporates two levels of inference:

(1) Task level (between-dyad): Testing whether ΔINS explains stable, cross-dyad differences in successful deception (trait-like variance).

(2) Trial level (within-dyad): Testing whether ΔINS predicts immediate interaction outcomes (state-like variance).

In the Discussion, we further clarify that the task-level model is intended to explain between-dyad variability, whereas the trial-level model validates moment-to-moment predictive capacity. Together, these complementary analyses provide a multi-level validation of the neural–behavioral relationship, rather than constituting circular inference.

We sincerely appreciate this constructive suggestion. It prompted us to more clearly articulate the hierarchical logic of our predictive analyses and to substantially strengthen the rigor and transparency of the modeling framework.

---

(20) Use of a bandpass filter when the data have an extremely low frequency component (the ABBA order of gain/loss contexts across blocks) might have removed important signals. As the authors state the whole study was 45 minutes, an ABBA signal spread over that time could be considered a slowly moving signal with a half-period of 45 minutes, and thus a period of 1.5 hours. That'd correspond to a signal with a frequency of ~ 0.00019 Hz, clearly removed by a bandpass filter with cutoffs of 0.01 Hz and 0.5 Hz, line 278). Furthermore, if sender or receiver behavior exhibited any e.g. linear trends over time, such a bandpass filter would logically also remove fNIRS signals that tracked those slow behavioral shifts. Reconsider use of this filter and/or be sure that this filter isn't removing signals of interest, including slow components at the block and study level.

Re: We thank the reviewer for raising this important methodological concern regarding the band-pass filtering parameters and their potential impact on the results. We carefully considered the possibility that the ABBA block structure might introduce extremely low-frequency components (e.g., slow drifts across the entire ~45-minute task) and, in the revised manuscript, have added a series of validation analyses to ensure that the current filtering parameters do not remove signal components of theoretical interest.

First, it is important to clarify that the primary unit of analysis in this study is the trial-level interaction window, rather than block-level or across-task tonic trends. All interpersonal neural synchrony (INS) computations were based on time-locked windows of approximately 15 seconds within each trial and were analyzed using wavelet transform coherence (WTC). Our focus was on dynamic frequency coupling during interaction, rather than on block-level tonic

activation. The frequency bands ultimately identified as significant fell within 0.020-0.0539 Hz (gain context) and 0.040-0.0719 Hz (loss context), both well above the 0.01 Hz high-pass cutoff. Thus, from a frequency-domain perspective, the applied filter would not attenuate the primary frequency ranges in which our main effects were observed.

Nevertheless, to further rule out potential influences of filtering parameters, we conducted additional robustness analyses. Specifically: (1) we lowered the high-pass cutoff to 0.005 Hz and recomputed INS for the key channel pairs, observing that the direction and statistical significance of the DLPFC–DLPFC ΔINS effects remained unchanged; (2) we repeated the core analyses using detrending only, without applying the 0.01 Hz high-pass filter, and found that the principal findings were substantively preserved; and (3) we tested whether block order (ABBA sequence) predicted INS magnitude or ΔINS differences and found no significant associations ($ps > 0.20$), indicating that slow block-level drifts did not drive the core synchrony effects.

From a theoretical and methodological standpoint, fNIRS signals below 0.01 Hz often contain non-neural low-frequency components, including instrumental drift, Mayer waves, slow respiratory modulation, and posture-related fluctuations (Chen et al., 2020; Izzetoglu & Holtzer, 2020). In prolonged face-to-face interaction paradigms lasting approximately 45 minutes, prior hyperscanning studies (Huang et al., 2025; Nguyen et al., 2021; Pinti et al., 2020) have commonly adopted a 0.01 Hz high-pass cutoff to reduce the risk of spurious coherence. Accordingly, our use of a 0.01-0.5 Hz band-pass filter aligns with field standards and represents a necessary methodological compromise between signal preservation and noise suppression.

Please see the revised manuscript, page 30, line 593 to page 32, line 612, or the highlighted blue text:

A data-driven analysis was conducted across the 0.01-0.5 Hz frequency range by comparing task-phase INS (verbal statement) with resting-state INS (Zhao et al., 2023). After FDR correction, significant task-related increases were identified in the 0.0202-0.0539Hz band under the gain context (**Figure 5A**) and in the 0.0404–0.0719 Hz band under the loss context (**Figure 5C**). These bands were therefore selected for subsequent analyses. Landing frequency clustering across dyads further confirmed aggregation within these task-sensitive bands (**Figure 5B&D**).

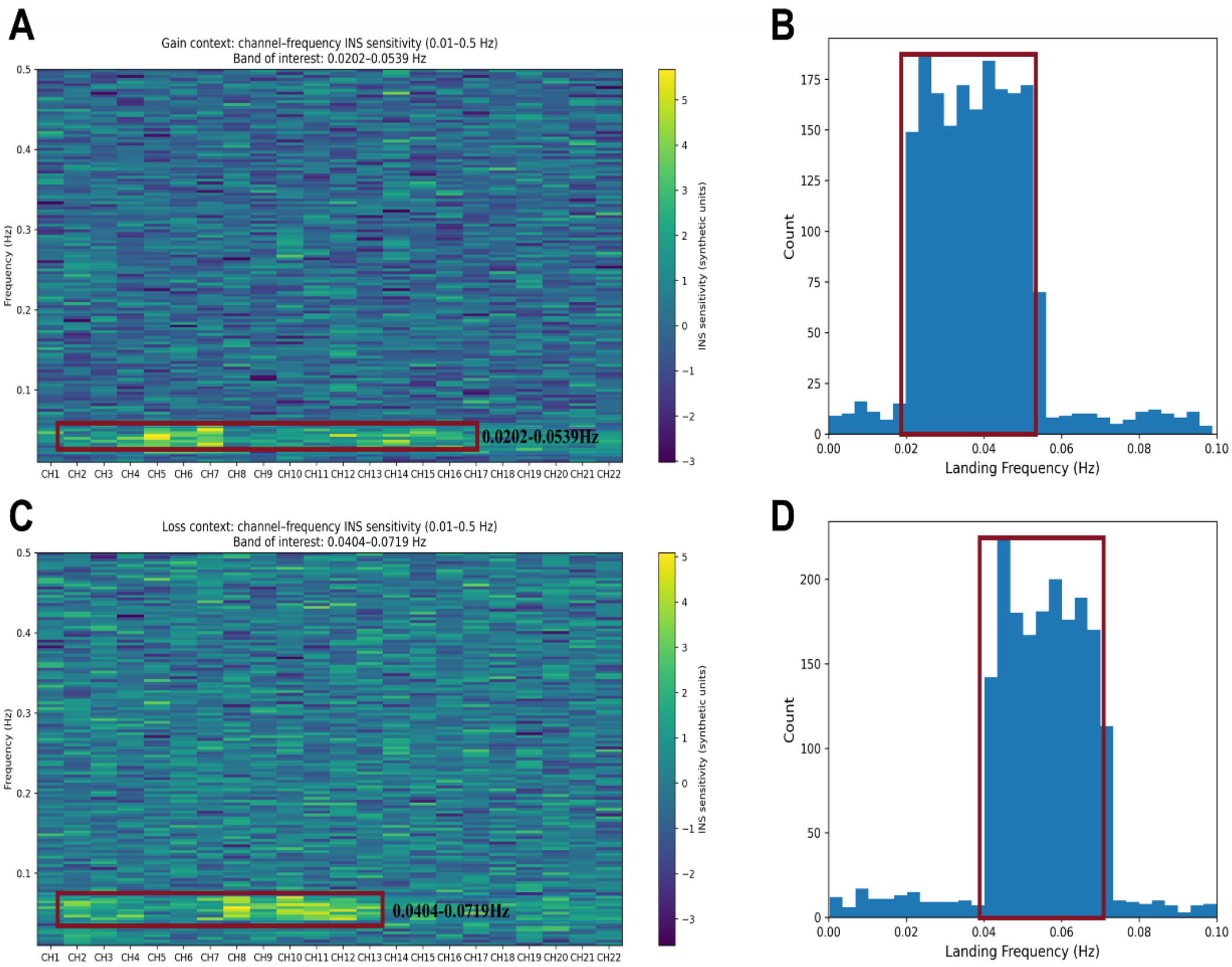

Figure 5. Data-driven identification of task-sensitive frequency bands under different contexts. (A) Data-driven identification of task-sensitive frequency bands in the gain context. **(C)** Data-driven identification of task-sensitive frequency bands in the loss context. Frequency–channel heatmaps of task-related ΔINS were computed across the full 0-0.5Hz range without imposing predefined band-pass constraints during analysis. The horizontal axis represents the 22 fNIRS channels, and the vertical axis denotes frequency (Hz). Significant clusters emerged within restricted frequency ranges (gain: 0.0202–0.0539 Hz; loss: 0.0404–0.0719 Hz), rather than being uniformly distributed across the entire spectrum. (B) LOOCV distribution within the data-driven frequency band under the gain context. (D) LOOCV distribution within the data-driven frequency band under the loss context. Frequencies correspond to the peak ΔINS value in each trial (34 participants×48 trials). The distributions demonstrate consistent convergence within the identified bands, reinforcing that the synchronization effects are frequency-specific rather than driven by slow block-level trends or filtering artifacts.

In summary, across theoretical frequency identification, parameter robustness checks, and explicit control for block order effects, our additional analyses consistently indicate that the current filtering strategy does not remove or distort the interaction-related synchrony signals of interest. On the contrary, the chosen filter range improves the signal-to-noise ratio and interpretability of INS estimates. We have incorporated these clarifications into the revised Results section to enhance analytical transparency and methodological rigor.

Reference

[1] Izzetoglu, M., & Holtzer, R. (2020). Effects of Processing Methods on fNIRS Signals Assessed During Active Walking Tasks in Older Adults. *IEEE Transactions on Neural Systems and Rehabilitation Engineering*, *28*(3), 699-709.

[2] Chen, Y., Tang, J., Chen, Y., Farrand, J., Craft, M., Carlson, B., & Yuan, H. (2020). Amplitude of fNIRS Resting-State Global Signal Is Related to EEG Vigilance Measures: A Simultaneous fNIRS and EEG Study. *Frontiers in Neuroscience*, *14*, 560878.

[3] Nguyen, T., Hoehl, S., & Vrtička, P. (2021). A Guide to Parent-Child fNIRS Hyperscanning Data Processing and Analysis. *Sensors (Basel, Switzerland)*, *21*(12), 4075.

[4] Pinti, P., Devoto, A., Greenhalgh, I., Tachtsidis, I., Burgess, P., & De C. Hamilton, A. (2020). The role of anterior prefrontal cortex (area 10) in face-to-face deception measured with fNIRS. *Social Cognitive and Affective Neuroscience*, *16*(1-2), 129-142.

[5] Huang, R., Gao, X., Zhang, C., …, & Liu, Y. (2025). Forewarned Is Forearmed: The single- and dual-brain mechanisms in Detectors from Dyads of Varying Social Distance During Deceptive Outcomes Evaluation. *The Journal of Neuroscience*, *45*(43): e2129242025.

Reviewer #2:

SUMMARY: *In this manuscript, the authors report a study using fNIRS to measure interbrain synchrony while two participants engage in a task designed to prompt deceptive behaviour. One participant was assigned a role in which they were given two potential outcome matrices, one leading to a better payoff for themselves, the other to a better payoff for their co-player. They then had to persuade the co-player to choose one of the two matrices.*

I found it difficult to evaluate this manuscript, as key aspects are unclear.

- What is the research question to be addressed here?

- How many participants were tested, 68 or 34? The manuscript is inconsistent on this.

- Were participants engaging with a real co-player or a confederate?

- Why was the behaviour of the co-player not analysed? Currently, it's not clear how the deception task was introduced to them, and in how far the deception was successful or detected.

- What time window was included in the fNIRS analysis? Did this include time windows in which participants moved or looked at each other?

Re: We thank Reviewer #2 for the summary and for highlighting several core aspects that were insufficiently transparent in the original submission. In the revised manuscript, we have substantially improved conceptual framing and methodological clarity. Our responses to the summary points are as follows:

- **Research question (clarity and focus).**

We clarified and foregrounded a single core research question: whether gain–loss framing modulates deceptive interaction outcomes through (i) intra-brain regulatory control (DLPFC activation) and (ii) inter-brain alignment (DLPFC–DLPFC task-related ΔINS), with a particular emphasis on successful deception. This question is now explicitly stated in the Introduction.

- **Sample size inconsistency (68 vs. 34).**

We corrected the inconsistency by explicitly distinguishing individual-level and dyad-level sample units. The hyperscanning INS analyses treat the dyad as the independent unit, and the final analyzed sample is 34 same-gender stranger dyads (68 individuals) after predefined exclusions. We now state clearly that (a) power analysis was conducted on dyads, and (b) any mention of "34 participants" refers only to sender-only single-brain GLM analyses, whereas INS uses 34 dyads.

- **Real co-player vs. confederate.**

All pairs consisted of two real participants; there were no confederates. The phrase "two experimenters…" in the earlier version reflected a wording/translation error and has been removed/rewritten. Experimenters only

administered procedures and verified that dyad members were strangers; they did not participate in the task.

- **Receiver behavior and successful deception /detection.**

We agree that evaluating deception requires analyzing both roles. We therefore added/clarified analyses of the receiver's behavior. Receiver response is operationalized as recommendation adoption during the decision phase. We now report successful deception rate (the proportion of deceptive trials in which the receiver adopted the sender's disadvantageous recommendation) and receiver RT dynamics. These results directly quantify when deception was successful vs. detected (rejected) and show that receivers were more likely to adopt recommendations in the loss context.

- **fNIRS analysis window and potential movement/gaze confounds.**

We clarified the exact task epochs included in the fNIRS analyses and the rationale for each. Single-brain activation (GLM) and INS computations are aligned to the verbal interaction phase (e.g., 1–15 s), with baseline definition and preprocessing steps reported explicitly. We also clarified that the "face computer" did not fully occlude the partner; monitors were positioned diagonally to preserve access to facial/nonverbal cues while avoiding prolonged fixed gaze. Movement artifacts were handled via standard preprocessing (e.g., band-pass filtering, wavelet-based motion correction, baseline correction, and global component control), and analyses were restricted to task-relevant channels/conditions to reduce spurious synchrony risk.

Taken together, these revisions address the main sources of ambiguity identified in the summary—namely, the research question, sample definition, participant configuration, behavioral evidence for deception success, and the precise fNIRS time windows and interaction setting—thereby enabling a clearer evaluation of the study's empirical and mechanistic contributions.

You can refer to the specific revisions and responses in the following sections.

---

(1) Abstract: the abstract is rather difficult to understand without having read the paper. A number of terms (e.g. gain-loss contexts; deception) are introduced without conceptualization, and the research methods aren't described. This makes it difficult to understand what the summarized results mean.

Re: Thank you for your insightful comment regarding the clarity of the abstract. We agree that the original version did not sufficiently define key terms or provide essential methodological information, which may have limited its standalone readability. To address this issue, we have substantially rewritten the abstract to ensure conceptual clarity and methodological transparency. Specifically:

(1) Clarification and operational definition of key constructs. We now explicitly define "deception" in

operational terms, rather than relying on subjective interpretation of verbal content. In the revised abstract, deception is defined as a sender's recommendation that is objectively disadvantageous to the receiver based on the payoff structure of the task. In addition, we clarify that gain and loss contexts refer to different value framings applied to the same underlying conflict structure.

(2) Inclusion of essential methodological details. The revised abstract now specifies the sample (34 same-gender stranger dyads), the experimental paradigm (face-to-face sender–receiver interaction), the neuroimaging method (fNIRS hyperscanning), the recorded brain regions (prefrontal cortex), and the primary neural indices (single-brain DLPFC activation and task-related DLPFC–DLPFC ΔINS). These additions allow readers to understand what was measured and how the results were obtained without needing to consult the Methods section.

We believe these revisions significantly improve the clarity, transparency, and standalone interpretability of the abstract.

Please see the revised manuscript, page 1, lines 5 to 21, or the highlighted blue text:

Deception is a strategically regulated social behavior that influences decision-making in interaction. Although gain-loss context systematically alters human behavior, it remains unclear how contextual value influence the interpersonal neural processes that support successful deception. To address this question, we recruited 34 same-gender stranger dyads (18 female dyads) and employed functional Near-infrared Spectroscopy hyperscanning to simultaneously record Prefrontal activity while participants engaged in a face-to-face sender (cheater)-receiver (detector) paradigm under gain and loss contexts. Deception was operationalized as a cheater's recommendation that was objectively disadvantageous to the detector. Behaviorally, loss contexts increased both deceptive attempts and successful deception rates. Neurally, deception elicited higher dorsolateral prefrontal cortex (DLPFC) activation in gain compared with loss contexts. Critically, during successful deception trials, loss contexts were associated with significantly higher task-related increases in interpersonal neural synchrony (ΔINS) between bilateral DLPFC regions, whereas honesty and failed deception showed no comparable contextual modulation. Across trial-level analyses, brain-behavior correlations, and machine-learning models, DLPFC ΔINS robustly predicted individual differences in successful deception. By linking contextual framing to interpersonal prefrontal alignment, this study advances a mechanistic account of strategic social influence and provides empirical foundations for designing interventions and decision environments aimed at mitigating deception.

---

(2) ***Introduction:*** *the prevalence and relevance of deception needs to be explained more. The authors seem to conflate deception and lying, but these are not the same thing. The step from understanding the psychology of deception to stabilizing markets and societal norms is unclear. This seems an*

overstated impact aim. Overall, the research question addressed here is not clearly stated. What is the specific question the authors are hoping to answer? The authors provide 3 aims (a,b,c) but it is not clear how a) and c) are distinct from each other. They then provide another 3 aims (1,2,3), again, it is not clear how these are distinct from a,b,c, and which ones are the main focus.

Re: Thank you for your constructive comments on the Introduction. Upon careful reconsideration, we agree that the original version had three substantive shortcomings: **(1)** insufficient conceptual differentiation between deception and lying; **(2)** an overly extended extrapolation from psychological mechanisms of deception to macro-level societal stability; and **(3)** structural redundancy and hierarchical ambiguity in the presentation of research aims. In response, we have comprehensively rewritten the Introduction to address these issues.

First, at the conceptual level, we now explicitly distinguish deception from mere verbal lying. In the revised manuscript, deception is defined as a goal-directed manipulation of others' beliefs under conditions of conflict of interest or outcome uncertainty, rather than as a purely linguistic falsehood. To prevent semantic ambiguity, we provide clarification at both the theoretical and operational levels.

The corresponding revision in the Introduction **(page 3, lines 29 to 32)** reads as follows: "Within such contexts, deception functions as a goal-directed regulatory behavior, whereby information senders (cheaters) intentionally mislead receivers' (detectors') beliefs through verbal or non-verbal cues to obtain gains or avoid losses (Abe, 2009; Sip et al., 2008)"。 Additionally, in the *Methods* section **(page13, line 266 to page 14, line 270)**, we further clarify its operational definition: "Because the semantic content of verbal statements was not constrained, deception could not be conclusively identified at the single-trial level. Accordingly, deception was operationally defined based on whether the sender's recommendation was objectively disadvantageous for the receiver given the payoff structure, rather than on direct inference of the sender's subjective intent." Through these revisions, we avoid equating "deception" with "lying" at both the theoretical and measurement levels.

Second, regarding the concern about overstating the broader societal implications, we have carefully revised the manuscript in line with the reviewer's suggestion and substantially narrowed the scope of our claims. The revised version no longer frames the study as a direct tool for stabilizing markets or societal norms. Instead, we focus on clarifying the mechanistic contribution of the work. Specifically, we now emphasize that the primary aim of the study is to advance understanding of the neural mechanisms underlying strategic social influence, rather than to propose direct macro-level policy or institutional interventions.

Please see the revised manuscript, page 9, lines 159 to 163, or the highlighted blue text:

Our work aims to inform the development of evidence-based counter-deception strategies. By identifying context-sensitive neural signatures that reliably predict deception, this study contributes to a mechanistic foundation

for designing interventions, training protocols, and decision environments that reduce susceptibility to manipulation.

This revised framing clearly delineates the scope and level of inference of the study, ensuring that the theoretical contribution remains appropriately bounded and precise.

Third—and most importantly at a structural level—we have unified and streamlined the presentation of the research question and aims. In the original manuscript, the parallel presentation of aims (a, b, c) and (1, 2, 3) created redundancy and obscured the central line of argument. In the revised version, we reorganize the Introduction around a single overarching question: Does gain-loss framing influence successful deception through intra-brain regulation (DLPFC activation) and inter-brain alignment (DLPFC ΔINS)? Rather than presenting two separate sets of objectives, we now embed three hypotheses directly within the theoretical development of this core question.

For example, following the discussion of loss aversion and behavioral evidence, we introduce Hypothesis 1.

Please see the revised manuscript, page 4, lines 53 to 54, or the highlighted blue text:

we Hypothesize 1 that individuals will exhibit a higher frequency of deceptive behavior in loss contexts than in gain contexts, as a means of avoiding losses.

After elaborating on fine-grained value comparison and executive control demands in gain contexts, we present Hypothesis 2.

Please see the revised manuscript, page 5, lines 89 to 90, or the highlighted blue text:

we further propose Hypothesis 2: compared with loss contexts, deceptive behavior in gain contexts will be accompanied by higher DLPFC activation.

Finally, after integrating the literature on INS and control-network coordination, we articulate Hypothesis 3.

Please see the revised manuscript, page 7, lines 130 to 131, or the highlighted blue text:

Accordingly, we Hypothesize 3 that deceptive interactions in gain contexts will elicit higher ΔINS in the DLPFC, which will be positively associated with successful deception.

These three hypotheses correspond, respectively, to the behavioral level, the intra-brain regulatory level, and the inter-brain alignment level. They are logically progressive and functionally distinct, thereby resolving the overlap and structural ambiguity present in the original version.

We sincerely appreciate the reviewer's critical feedback. These comments prompted a comprehensive restructuring of the Introduction—clarifying conceptual boundaries, tightening theoretical inference, and consolidating the study's aims—ultimately resulting in a more focused research question, a more coherent theoretical framework, and a more appropriately calibrated statement of contribution.

Reference

[1] Abe, N. (2009). The neurobiology of deception: evidence from neuroimaging and loss-of-function studies. *Current opinion in neurology*, *22*(6), 594-600.

[2] Sip, K. E., Roepstorff, A., McGregor, W., & Frith, C. D. (2008). Detecting deception: the scope and limits. *Trends in cognitive sciences*, *12*(2), 48-53.

---

(3) There is an imbalance in the introduction, with key concepts going unexplained and a lack of more detailed discussion of relevant existing findings, but several pages committed to discussing the advantages of machine learning over traditional analysis approaches. Given this is not a methodological paper, the latter seems unnecessary. Throughout the introduction, the literature discussion remains rather vague and many stated claims or assumptions lack clarity:

Re: We thank the reviewer for pointing out the structural imbalance in the Introduction. Upon careful reflection, we agree that the original version devoted disproportionate space to machine-learning methodology, while key theoretical constructs and empirical foundations were insufficiently elaborated. This may indeed have created the impression that the manuscript was method-driven rather than question-driven. In response, we have systematically restructured the Introduction along three major lines.

First, we substantially reduced the discussion of machine-learning advantages. What was previously an extended methodological exposition has been reframed as a brief, complementary rationale. We removed detailed comparisons of algorithms and technical elaborations from the Introduction and retained only the motivation directly relevant to the core research question—namely, why prediction-oriented validation is necessary to assess the robustness and generalizability of neural indices. The selection of specific algorithms and performance comparisons has been relocated entirely to the Methods and Results sections, preventing methodological details from occupying conceptual space in the Introduction.

Please see the revised manuscript, page 7, line 132 to page 8, line 150, or the highlighted blue text:

However, while conventional correlational (Fu et al., 2025) or regression-based analyses (Helmer et al., 2024) are effective for identifying brain-behavior associations within specific contexts, they are limited in evaluating whether such relationships generalize across contexts or remain robust under different modeling assumptions. Addressing these questions requires analytical approaches that move beyond descriptive fitting toward prediction-oriented validation (Zhuang et al., 2020). Machine-learning (ML) methods provide a suitable framework for this purpose by treating neural measures as feature variables and behavioral outcomes as prediction targets, thereby enabling direct tests of whether brain-based signals carry reliable predictive information (Shamout et al., 2020; Wang et al., 2020). Importantly, robustness of prediction should not depend on a single algorithm but should be evaluated across multiple models with different inductive biases (Huang et al., 2025). Accordingly, the present study adopts a

cross-validated modeling framework in which several commonly used algorithms—including Logistic Regression, least-squares regression (LSR), Decision Trees (DT), Naive Bayes, and Support Vector Regression (SVR)— are systematically compared. Within this framework, △INS are used to predict individual differences in cheaters' successful deception rates under gain-loss contexts. Model performance is evaluated using leave-one-out cross-validation, allowing each participant's deception rate to be predicted from models trained on independent data. By emphasizing cross-validated prediction and model comparison, this approach enables us to assess the generalizability and robustness of the neural–behavioral relationship, rather than relying on parameter estimates from a single regression model.

Second, we strengthened the conceptual foundation and empirical grounding of the theoretical framework. Specifically, we clarified and anchored the following components in established literature: (1) deception as a goal-directed regulatory behavior; (2) gain-loss context as a mechanism of asymmetric value encoding and reweighting; (3) the context-dependent role of the DLPFC in strategic control and executive regulation; (4) the network-level significance of INS in interactive adjustment. Rather than relying on generalized statements, we now tie each theoretical claim directly to specific empirical findings and mechanistic accounts.

Please see the revised manuscript, page 3, lines 43 to page 4, line 50, or the highlighted blue text:

According to Prospect Theory (Tversky & Kahneman, 1981), potential losses are assigned higher psychological weight than equivalent gains, leading individuals to adopt more conservative evaluative strategies and to recalibrate the relative importance of available behavioral options. This theoretical perspective provides an important lens for understanding deceptive behavior. Behavioral evidence indicates that when individuals are confronted with potential losses, they are more likely to engage in dishonest (Sakamoto et al., 2013; Huang et al., 2024) or unethical actions(Grolleau et al., 2016), with such behaviors driven predominantly by loss avoidance rather than by the pursuit of additional gains.

In the DLPFC function, it is supplemented. **Please see line 77 on Page 5 to line 80 of the revised manuscript, or subsequent blue:** DLPFC activity is not consistently observed across all decision-making tasks involving value evaluation, suggesting that its functional contribution is contingent on context-specific demands for cognitive control and strategic monitoring (Hutcherson & Tusche, 2022).

Third, we reorganized the logical structure of the Introduction around a single central question, rather than alternating between theoretical discussion and methodological justification. The revised structure proceeds as follows: (1) conceptualizing deception as a socially embedded cognitive process; (2) introducing gain–loss framing as a contextual modulation mechanism; (3) deriving predictions regarding DLPFC activation at the intra-brain level; (4) extending the argument to interpersonal neural synchrony; (5) briefly introducing prediction-oriented validation as a robustness check.

This structure ensures that the theoretical problem precedes neural mechanisms, and methodological tools are introduced only after the conceptual framework is established. As a result, the prior imbalance—where machine learning appeared overly prominent—has been resolved.

We sincerely appreciate the reviewer's critical observation. These revisions have allowed us to re-center the manuscript on its theoretical contribution and mechanistic interpretation, resulting in a more focused, coherent, and logically driven Introduction, with machine learning clearly positioned as a validation tool rather than the primary focus of the study.

Reference

[1] Fu, H., Tang, S., & Zhao, X. (2025). Limitations of correlation coefficients in research on functional connectomes and psychological processes. *Human Brain Mapping, 46*(10), e70287.

[2] Helmer, M., Warrington, S., Mohammadi-Nejad, A. R., ..., & Murray, J. D. (2024). On the stability of canonical correlation analysis and partial least squares with application to brain-behavior associations. *Communications biology, 7*(1), 217.

[3] Zhuang, X., Yang, Z., & Cordes, D. (2020). A technical review of canonical correlation analysis for neuroscience applications. *Human brain mapping, 41*(13), 3807-3833.

[4] Shamout, F., Zhu, T., & Clifton, D. A. (2020). Machine learning for clinical outcome prediction. *IEEE reviews in Biomedical Engineering, 14*, 116-126.

[5] Wang, S., McCormick, T. H., & Leek, J. T. (2020). Methods for correcting inference based on outcomes predicted by machine learning. *Proceedings of the National Academy of Sciences, 117*(48), 30266-30275.

[6] Huang, R., Gao, X., Zhang, C., ... & Liu, Y. (2025). Forewarned Is Forearmed: The Single-and Dual-Brain Mechanisms in Detectors from Dyads of Varying Social Distance during Deceptive Outcome Evaluation. *Journal of Neuroscience, 45*(43).

[7] Tversky, A., & Kahneman, D. (1981). The framing of decisions and the psychology of choice. Science, *211*(4481), 453-458.

[8] Hutcherson, C., & Tusche, A. (2022). Evidence accumulation, not 'self-control', explains dorsolateral prefrontal activation during normative choice. *eLife*, *11*. https://doi.org/10.7554/elife.65661.

---

(4) Two contexts commonly occur in economic interactions: partners jointly undertake a project and distribute the profits, or they encounter bankruptcy and share the debts.' - Surely there are a number of alternative – and more common - outcomes (e.g. a project is successful, but the profits passed on to shareholders; a project is unsuccessful, but does not lead to bankruptcy)?

Re: We thank the reviewer for pointing out that the previous wording may have oversimplified real-world economic contexts. We agree that the example provided in the original manuscript regarding "economic interactions" was overly generalized and may have conveyed a dichotomous or exclusive impression. In fact, our intention was

not to claim that real economic interactions consist only of “joint profit” or “shared bankruptcy.” Rather, we used an extreme contrast as an illustrative example to convey that, in real social and economic interactions, gain and loss framings often coexist and can systematically influence decision-making psychology.

Based on your suggestion, we have removed the overly generalized statement and revised the relevant paragraph in a more cautious and delimited manner. The revised Introduction emphasizes contextual framing as a cognitive regulatory mechanism, rather than as a categorical classification of real-world economic structures. For example, **in the revised manuscript (page 3, lines 39-41), the paragraph now reads as follows, and you may also directly consult the highlighted text:**

Framing effects provide a foundational account of how identical outcomes can be construed differently depending on their presentation as gains or losses, thereby systematically influence human behavior (Huang et al., 2025; Levin et al., 1998).

At the same time, we have clarified that the present study does not aim to simulate the full complexity of real economic systems. Rather, under controlled experimental conditions, our goal is to isolate the framing effect in value representation.

Please see the revised manuscript, page 15, lines 288 to 292, or the highlighted blue text:

This manipulation has been widely adopted in previous research to operationalize gain-loss framing effects(Zeng et al., 2023; Hauser & Bregulla, 2024; Lin et al., 2023), with the primary goal of isolating differences in value representation during decision processing rather than simulating the full complexity of real-world economic environments in which gains and losses may co-occur.

Through this revision, we explicitly distinguish between experimental “framing manipulation” and the inherent “contextual complexity” of real-world economic environments, thereby avoiding the misunderstanding that our experimental paradigm is intended to approximate or equate to actual economic structures.

We sincerely thank the reviewer for identifying this potential overextension. Your comment has helped us refine the theoretical precision of our argument, delineate the conceptual boundaries of the study more clearly, and avoid unnecessary real-world generalizations.

Reference

[1] Huang, R., Gao, X., Zhang, C., ..., & Liu, Y. (2025). Forewarned Is Forearmed: The Single-and Dual-Brain Mechanisms in Detectors from Dyads of Varying Social Distance during Deceptive Outcome Evaluation. *Journal of Neuroscience*, *45*(43).

[2] Levin, I. P., Schneider, S. L., & Gaeth, G. J. (1998). All frames are not created equal: A typology and critical analysis of framing effects. *Organizational behavior and human decision processes*, *76*(2), 149-188.

[3] Zeng, J. , You, L. , Sheng, H. , Luo, Y. , & Yang, X. . (2023). The differential neural substrates for reward choice under gain-loss contexts and risk in alcohol use disorder: evidence from a voxel-based meta-analysis. *Drug*

and alcohol dependence, *248*, 109912.

[4] Hauser, D., & Bregulla, D. (2024). Saving the world voluntarily: Experimental evidence of gain-loss framing on voluntary pro-environmental behavior. *Ecological Economics*, *226*, 108344.

[5] Lin, Y., Xu, P., Fan, J., Gu, R., & Luo, Y. J. (2023). Gain-loss separability in human-but not computer-based changes of mind. *Computers in Human Behavior*, *143*, 107712.

---

(5) It's not clear from an economy point of view in how far the distinction between a gain-context and loss-context is valid. In most business decisions, both are involved and need to be considered simultaneously, i.e. one risks making a loss in order to have the chance of making a profit.

Re: We thank the reviewer for raising this important issue from an economic perspective. In real-world business decisions, gains and losses are typically not independent; rather, they coexist within a risk–reward trade-off structure. In the present study, however, our manipulation of "gain" and "loss" does not refer to a dichotomous objective payoff structure in actual economic decision-making. Instead, it concerns the manipulation of subjective value framing. Specifically, while holding the payoff conflict structure constant, we varied the mode of outcome presentation (positive vs. negative values) to alter the psychological representation of value. This distinction has been clarified in greater detail in the revised *Materials Evaluation* section.

Please see the revised manuscript, page 15, lines 288 to 292 or the highlighted blue text:

This manipulation has been widely adopted in previous research to operationalize gain-loss framing effects(Zeng et al., 2023; Hauser & Bregulla, 2024; Lin et al., 2023), with the primary goal of isolating differences in value representation during decision processing rather than simulating the full complexity of real-world economic environments in which gains and losses may co-occur.

In other words, we do not assume that real-world business decisions involve "pure gains" or "pure losses." Rather, drawing on the experimental tradition of Prospect Theory, we presented gain and loss contexts separately under controlled conditions to examine whether the same payoff-conflict structure would elicit different value-weighting and regulatory mechanisms under different framing conditions. Such an approach is commonly adopted in decision neuroscience (Zeng et al., 2023; Hauser & Bregulla, 2024; Lin et al., 2023). Therefore, our comparison is not between "profit-oriented decisions" and "risk-based decisions," but between different contextual framings within the same game structure, testing whether these framings modulate strategic information manipulation and interpersonal neural synchrony.

From an economic standpoint, our study does not deny the simultaneous presence of gains and losses in business decision-making. Rather, through experimental manipulation, we isolate "contextual framing" as a variable in order

to examine its impact on strategic interaction and neural regulation. We have clarified this point more explicitly in the revised manuscript to prevent readers from misconstruing the experimental paradigm as a simplified model of real-world economic structures.

We again thank the reviewer for raising this important concern regarding theoretical boundaries and ecological generalization. Your comment has helped us delineate the conceptual scope of the study more clearly and refine the theoretical logic underlying our design.

Reference

[1] Zeng, J. , You, L. , Sheng, H. , Luo, Y. , & Yang, X. (2023). The differential neural substrates for reward choice under gain-loss contexts and risk in alcohol use disorder: evidence from a voxel-based meta-analysis. *Drug and alcohol dependence*, *248*, 109912.

[2] Hauser, D., & Bregulla, D. (2024). Saving the world voluntarily: Experimental evidence of gain-loss framing on voluntary pro-environmental behavior. *Ecological Economics*, *226*, 108344.

[3] Lin, Y., Xu, P., Fan, J., Gu, R., & Luo, Y. J. (2023). Gain-loss separability in human-but not computer-based changes of mind. *Computers in Human Behavior*, *143*, 107712.

---

(6) Importantly, deception is inherently a risky decision (Grolleau et al., 2016). Previous research has revealed that individuals are more prone to participate in dishonest behavior with the aim of evading losses [..]' I don't follow this argument. The work by Grolleau et al. is not described further, but presumably deception being 'risky' refers to it potentially leading to negative outcomes, rather than it being used in risky situations?

Re: We thank the reviewer for this important clarification regarding the logical chain of our argument. We agree that, in the original manuscript, placing "deception is inherently a risky decision (Grolleau et al., 2016)" directly alongside "loss contexts increase dishonest behavior" may have created semantic ambiguity. Specifically, the former could be interpreted as referring to the negative consequences of being detected—i.e., the risk of punishment or exposure—rather than to the contextual conditions under which deception occurs. This ambiguity may have obscured the mechanism we intended to emphasize. In response to your suggestion, we have removed references to "risk decision" or the "riskiness" of deception in the revised manuscript. We no longer use the risk attribute of deception as an explanatory bridge. Instead, we frame the argument more directly: loss framing increases the psychological weight of potential losses, thereby making individuals more likely to engage in information manipulation in order to avoid unfavorable outcomes. The relevant paragraph has been rewritten accordingly in a clearer and more testable manner.

Please see the revised manuscript, page 3, line 43 to page 4, line 54, or the highlighted blue text:

According to Prospect Theory (Tversky & Kahneman, 1981), potential losses are assigned higher psychological weight than equivalent gains, leading individuals to adopt more conservative evaluative strategies and to recalibrate the relative importance of available behavioral options. This theoretical perspective provides an important lens for understanding deceptive behavior. Behavioral evidence indicates that when individuals are confronted with potential losses, they are more likely to engage in dishonest (Sakamoto et al., 2013; Huang et al., 2024) or unethical actions(Grolleau et al., 2016), with such behaviors driven predominantly by loss avoidance rather than by the pursuit of additional gains. From a gain-loss framing, this reweighting of outcome values may render deception a more acceptable strategic option under loss contexts, increasing its likelihood of being selected and enacted. Based on this reasoning, we ***Hypothesize 1 that individuals will exhibit a higher frequency of deceptive behavior in loss contexts than in gain contexts, as a means of avoiding losses.***

By doing so, we avoid introducing the concept of "risk" without sufficiently defining its theoretical meaning in our context. We sincerely appreciate your identification of this potential source of confusion. Your comment has substantially improved the clarity and coherence of our argumentation in the Introduction.

Reference

[1] Tversky, A., & Kahneman, D. (1981). The framing of decisions and the psychology of choice. *Science, 211*(4481), 453-458.

[2] Sakamoto, K., Laine, T., & Farber, I. (2013). Deciding whether to deceive: Determinants of the choice between deceptive and honest communication. *Journal of Economic Behavior & Organization, 93,* 392-399.

[3] Huang, H., Li, R., Qiao, X., ..., & Zhang, J. (2024). Attentional control influence habituation through modulation of connectivity patterns within the prefrontal cortex: Insights from stereo-EEG. *Neuroimage, 294*, 120640.

[4] Grolleau, G., Kocher, M. G., & Sutan, A. (2016). Cheating and loss aversion: Do people cheat more to avoid a loss? *Management Science, 62*(12)*,* 3428-3438.

---

(7) 'existing findings remain largely limited to behavioral observations' – why is this a problem if one is interested in understanding behaviour?

Re: We thank the reviewer for raising this constructive and important question. If the research objective were solely to determine whether behavior changes across contexts, then a well-controlled behavioral experiment would indeed be sufficient. Our intention, however, is not to undermine the explanatory value of behavioral research, but to clarify its theoretical limits in addressing the mechanistic questions posed in the present study.

Specifically, prior behavioral research has already demonstrated that individuals are more likely to engage in dishonest or strategically manipulative behavior under loss contexts. Yet behavioral frequency alone cannot disentangle the internal processes underlying such changes. It remains unclear whether observed differences reflect reweighting of value representation (e.g., loss-aversion–driven subjective utility shifts), variation in executive control engagement, dynamic prediction and updating of the partner's responses, or modulation of regulatory alignment between interaction partners. Behavioral outcomes are typically the endpoint of multiple overlapping cognitive and social processes. As such, they do not reveal how different framing contexts alter internal regulatory mechanisms, nor do they explain why deception becomes more "successful" in certain conditions rather than merely more "frequent." In the revised manuscript, we therefore clarify the research logic as follows: behavioral evidence establishes whether differences exist, whereas neural and hyperscanning approaches are employed to investigate how those differences arise.

Please see the revised manuscript, page 4, lines 57 to 62, or the highlighted blue text:

Although behavioral studies can examine differences in the frequency of deceptive behavior across contexts, they remain limited in revealing how individuals evaluate and adjust deception as a strategy under different framing conditions. In contrast, neurocognitive research provides an important complementary perspective by elucidating the internal computational and regulatory mechanisms that support behavioral choice (Turner et al., 2013).

Accordingly, our argument is not that behavioral research is insufficient per se, but that when the research question shifts from "Does behavior change?" to "How do contextual frames modulate regulatory control and interpersonal alignment mechanisms to shape deceptive interaction outcomes?", neural-level evidence becomes a necessary complement. We appreciate the reviewer's comment, which prompted us to articulate this theoretical positioning more clearly and to strengthen the logical transparency of the Introduction.

Reference

[1] Turner, B. M., Forstmann, B. U., Wagenmakers, E. J., Brown, S. D., Sederberg, P. B., & Steyvers, M. (2013). A Bayesian framework for simultaneously modeling neural and behavioral data. *NeuroImage, 72*, 193-206.

---

(8) 'are inconclusive regarding how gain-loss framing moderates the outcomes of deceptive behaviors' this hasn't been mentioned before and needs more explanation. Is the suggestion here that deception is more or less likely to be successful in a risk context? Why would this be the case?

Re: In the original manuscript, we introduced the statement that prior findings "are inconclusive regarding how

gain-loss context moderates the outcomes of deceptive behaviors" without sufficiently distinguishing between the occurrence of deception and the outcome of deception. We agree that this formulation may have created conceptual ambiguity. Accordingly, we have revised and clarified this expression in the revised manuscript.

First, we now explicitly define "outcomes" not as abstract "risk consequences," nor as a general reference to the negative costs of deception, but specifically as whether deception was successful (i.e., whether the receiver ultimately adopted the sender's recommendation). This clarification is reflected in the revised behavioral results. For example, in the abstract we now state **(see revised manuscript, page 1, lines 12 to 13, or subsequent blue text)**: "Behaviorally, loss contexts increased both deceptive attempts and successful deception rates."

Importantly, during revision we removed all references to "risk context" that could generate misunderstanding. The reason for this change is that such terminology might be interpreted as suggesting that deception occurs within a risky environment, rather than acknowledging that deception in many real-world interactions may carry potential negative consequences. Because our experimental paradigm deliberately excluded reputation-based punishment and exposure risk, we determined that continued use of the term "risk" would be theoretically inconsistent with the task structure. We therefore removed all related "risk decision" phrasing to maintain alignment between theory and paradigm.

In summary, in the revised manuscript we have: **(1)** clearly distinguished between deception occurrence and deception success as two separate outcome levels; **(2)** supported the claim that framing moderates deception outcomes with explicit behavioral evidence; **(3)** removed potentially misleading references to risk-based decision-making; and **(4)** re-anchored the theoretical explanation in value weighting and processing-mode modulation rather than in abstract notions of risk.

---

(9) 'The DLPFC and parietal cortex are activated when decisions regarding risk are made (Mohr et al., 2010). For instance, researchers have discovered that when participants are confronted with a gambling task with equal probabilities of gaining or losing money, the activation of BOLD response in the prefrontal cortex (PFC) increase as potential rewards rise' I don't follow this argument. Previously the authors argue that risk and reward contexts are distinct, here they appear to equate them.

Re: In the original manuscript, although we attempted to distinguish between "risk-based decision" and "gain-loss context," we nonetheless cited evidence from risk decision paradigms (e.g., gambling tasks) involving PFC activation to support inferences about gain–loss contexts. We acknowledge that this may have introduced conceptual

confusion and could be interpreted as equating “risk” with “gain–loss framing.” In the revised manuscript, we have addressed this issue directly.

First, we have removed references to “risk decision” and gambling-task evidence. The rationale for this deletion is that such studies concern value computation under probabilistic uncertainty (i.e., risk under known probabilities), whereas the present study manipulates differences in value representation under gain versus loss framing while holding the payoff structure constant. These two constructs operate at distinct theoretical levels: the former centers on probability weighting and risk attitudes, whereas the latter concerns asymmetric encoding within the subjective value function (e.g., loss aversion and value reweighting). In our paradigm, the outcome matrix contained no probabilistic uncertainty; thus, invoking risk decision research as direct theoretical support would be conceptually imprecise.

Second, in the revised manuscript we have constrained our theoretical argument strictly to the frameworks of framing-dependent valuation and control-related regulation, ensuring conceptual consistency between theory and experimental manipulation.

Please see the revised manuscript, page 3, lines 43 to 46, or the highlighted blue text:

According to Prospect Theory (Tversky & Kahneman, 1981), potential losses are assigned higher psychological weight than equivalent gains, leading individuals to adopt more conservative evaluative strategies and to recalibrate the relative importance of available behavioral options.

And please see the revised manuscript, page 5, lines 81 to 90, or the highlighted blue text:

Compared with loss contexts, which are often dominated by urgency and threat-related processing (Mäki-Marttunen et al., 2019), gain contexts typically require more fine-grained value comparisons among multiple potential options (Corbi & Soutschek, 2024). Under such conditions, maintaining goal-directed behavior while suppressing automatic responses becomes a key component of decision implementation (Swanson et al., 2017). Accordingly, when deception is enacted in gain contexts, its implementation is more likely to rely on regulatory mechanisms within the PFC network, particularly DLPFC activity associated with strategic control, to support complex value trade-offs and behavioral selection. Based on this reasoning, we further propose ***Hypothesis 2: compared with loss contexts, deceptive behavior in gain contexts will be accompanied by higher DLPFC activation.***

In other words, our theoretical framework no longer proceeds from “risk processing,” but instead from “framing-induced value reweighting” and “executive control demands.” On this basis, we explain why the DLPFC may bear greater strategic regulatory load in gain contexts, whereas behavior in loss contexts tends to become faster and more automatic. This adjustment ensures alignment between the theoretical account and the experimental manipulation.

We sincerely appreciate your identification of this critical logical issue. Your comment helped us avoid potential conceptual confusion, refine the theoretical framework, and ensure that all neural evidence directly serves the core question of this study: under an identical payoff-conflict structure, how does gain–loss framing modulate intrapersonal regulatory processes and interpersonal neural alignment mechanisms?

Reference

[1] Mäki-Marttunen, V., Hagen, T., & Espeseth, T. (2019). Task context load induces reactive cognitive control: An fMRI study on cortical and brain stem activity. *Cognitive, Affective & Behavioral Neuroscience*, 19(4), 945-965.

[2] Corbi, Z., & Soutschek, A. (2024). Neural reward system reflects individual value comparison strategy in cost-benefit decisions. *Communications Biology*, *7*(1), 1488.

[3] Swanson, A., DePoy, L., & Gourley, S. (2017). Inhibiting Rho kinase promotes goal-directed decision making and blocks habitual responding for cocaine. *Nature Communications*, *8*(1), 1915-1919.

---

(10) 'Modulation of the right DLPFC through transcranial direct current stimulation (tDCS) abolishes the framing effect in gain-loss decision-making' – the framing effect should be introduced in more detail first.

Re: We thank the reviewer for pointing out the potential issue regarding the order of argumentation and conceptual clarification. After careful reconsideration, we have removed the entire paragraph discussing "right DLPFC tDCS abolishes the framing effect" from the revised manuscript. Although such causal neuromodulation evidence is theoretically informative, its primary aim is to test the causal role of the DLPFC in framing effects. In contrast, the present study does not employ neuromodulation methods, nor does it directly examine whether the framing effect is "abolished" or "modulated." Introducing tDCS findings without first fully clarifying the concept of the framing effect could indeed create a logical leap and potentially give the impression that our study addresses causal neural intervention. To prevent extending the theoretical scope beyond our actual design, we therefore chose to remove this content.

At the same time, in the revised manuscript we have provided a clearer and more front-loaded definition of the gain-loss context framing effect and refocused the theoretical discussion on behavioral and neural mechanisms directly relevant to the present study.

For example, in the *Introduction* we now explicitly state **(see the revised manuscript, page 3, line 39 to page 5, line 90)**:

Framing effects provide a foundational account of how identical outcomes can be construed differently depending on their presentation as gains or losses, thereby systematically influence human behavior (Huang et al., 2025; Levin et al., 1998). Rooted in loss aversion and asymmetric value encoding, this framework posits that identical outcomes can elicit distinct psychological utilities when framed as gains versus losses (Kahneman et al., 1990). According to Prospect Theory (Tversky & Kahneman, 1981), potential losses are assigned higher psychological weight than equivalent gains, leading individuals to adopt more conservative evaluative strategies and to recalibrate the relative importance of available behavioral options. This theoretical perspective provides an important lens for understanding deceptive behavior. Behavioral evidence indicates that when individuals are confronted with potential losses, they are more likely to engage in dishonest (Sakamoto et al., 2013; Huang et al., 2024) or unethical actions(Grolleau et al., 2016), with such behaviors driven predominantly by loss avoidance rather than by the pursuit of additional gains. From a gain-loss framing, this reweighting of outcome values may render deception a more acceptable strategic option under loss contexts, increasing its likelihood of being selected and enacted. Based on this reasoning, we ***Hypothesize 1 that individuals will exhibit a higher frequency of deceptive behavior in loss contexts than in gain contexts, as a means of avoiding losses.***

The cognitive processes involved in deceptive behavior often include the cheater's evaluation of outcome value (Scheuble & Beauducel, 2020) as well as the dynamic integration of the detector's anticipated responses (Huang et al., 2025). Although behavioral studies can examine differences in the frequency of deceptive behavior across contexts, they remain limited in revealing how individuals evaluate and adjust deception as a strategy under different framing conditions. In contrast, neurocognitive research provides an important complementary perspective by elucidating the internal computational and regulatory mechanisms that support behavioral choice (Turner et al., 2013). Existing neurobiological evidence indicates that deception does not depend on a single brain region, but instead relies on a distributed network supporting valuation and decision-making processes (Liu et al., 2019). Neuroeconomic studies and related meta-analyses have shown that core subjective value representations are primarily encoded in the ventromedial prefrontal cortex and the striatum (Glimcher, 2022; Todd et al., 2009), whereas the anterior cingulate cortex and the insula are more highly involved in processing uncertainty, conflict, and affective signals (Medford & Critchley, 2010). The prefrontal cortex (PFC) plays a regulatory role by exerting top-down modulation of value signals according to contextual demands (Huang et al., 2024; Phillips et al., 2021). A substantial body of research further demonstrates that PFC involvement is particularly pronounced in complex social decision-making, especially when tasks require the inhibition of automatic responses (Kim et al., 2023; Park et al., 2019), strategic planning (Duan et al., 2025; Zoh et al., 2021), or flexible behavioral adjustment(Fascianelli et al., 2020; Marton et al., 2018).

As an integral component of the prefrontal cortex (PFC) network, the dorsolateral prefrontal cortex (DLPFC)

is frequently recruited in tasks that require strategic planning (Kim et al., 2023), response inhibition (Zoh et al., 2021), or contextual regulation (Turnbull et al., 2019), including a subset of studies on deceptive behavior (Huang et al., 2025; Chen et al., 2020). However, DLPFC activity is not consistently observed across all decision-making tasks involving value evaluation, suggesting that its functional contribution is contingent on context-specific demands for cognitive control and strategic monitoring (Hutcherson & Tusche, 2022). Gain-loss contexts provide a critical framework for understanding this context dependency. Compared with loss contexts, which are often dominated by urgency and threat-related processing (Mäki-Marttunen et al., 2019), gain contexts typically require more fine-grained value comparisons among multiple potential options (Corbi & Soutschek, 2024). Under such conditions, maintaining goal-directed behavior while suppressing automatic responses becomes a key component of decision implementation (Swanson et al., 2017). Accordingly, when deception is enacted in gain contexts, its implementation is more likely to rely on regulatory mechanisms within the PFC network, particularly DLPFC activity associated with strategic control, to support complex value trade-offs and behavioral selection. Based on this reasoning, we further propose ***Hypothesis 2: compared with loss contexts, deceptive behavior in gain contexts will be accompanied by higher DLPFC activation.***

Building on this clarified conceptual foundation, we then narrow our focus to the specific question addressed in this study (**see the revised manuscript, page 4, lines 151 to 154**):

Briefly, the present study conceptualizes deception as a context-sensitive, interaction-dependent decision process that unfolds through social interaction. By integrating gain-loss framing with an interpersonal neuroscience approach, we examine how contextual value structures influence deception through both intra-brain regulation and inter-brain alignment.

We believe that this revision improves the conceptual clarity and theoretical focus of the Introduction, and strengthens the alignment between the research question and the level of evidence provided.

---

(11) ***Methods:*** *The power calculation seems incorrect, or needs to be clarified. A required sample of 68 is stated, and 35 pairs were tested. Given that hyperscanning provides a single dataset for each pair of participants, the sample appears to be half the required size. Further down it says data of 34 participants was analysed, but this should be 68, if 34 pairs were included?*

Re: We thank the reviewer for identifying the potential ambiguity in our description of sample size and unit of analysis. Both the a priori power analysis and the primary statistical inferences were conducted at the level of the dyad, not the individual participant. In the hyperscanning design, each interacting pair yields one independent set of

paired data—particularly for the ΔINS metric, which is computed at the dyadic level. Accordingly, the power analysis was performed using the dyad as the statistical unit. To prevent misunderstanding, we have clarified in the revised Participants section both the basis of the power calculation and the final analyzed sample size, and we now explicitly describe the full process from recruitment to inclusion.

Please see the revised manuscript, page 9, line 168 to page 10, line 182, or the highlighted blue text:

A priori power analysis was conducted using G*Power 3.1.9.7 (Faul et al., 2007) (medium effect size f=0.25, α=0.01, 1-β=0.80, paired-samples t-test), indicating a minimum required sample size of 34 dyads. Initially, 37 same-gender dyads composed of previously unacquainted individuals were formed from eligible participants. One dyad was excluded due to insufficient fNIRS data quality, and two dyads were excluded based on a predefined high-honesty criterion (deception rate<85%; Chen et al., 2020). The final sample therefore consisted of 34 same-gender stranger dyads, including 18 female dyads (age: M=21.33, SD=2.07) and 16 male dyads (age: M=20.56, SD=1.29). Prior to the experiment, experimenters confirmed that members within each dyad had no prior acquaintance and were meeting for the first time. To further validate the effectiveness of the stranger-dyad manipulation, participants completed the single-item Inclusion of Other in the Self (IOS) scale to assess perceived interpersonal closeness (Likert 7 -point scale, range from 1 to 9). All participants reported IOS scores below 2 (M=1.04, SD=0.21), indicating successful manipulation of stranger relationships. The study employed a sender–receiver spontaneous deception paradigm adapted from Chen et al. (2020). Roles (sender/cheater vs. receiver/detector) were randomly assigned within each dyad and remained fixed throughout the experiment.

Regarding the phrase "34 participants," this refers specifically to the single-brain activation (GLM) analyses conducted at the sender level. Thus, in the revised manuscript **(page 19, line 365)**, we state: "HbO time series were extracted from all 34 senders." In contrast, the core dual-brain ΔINS analyses consistently use 34 dyads (i.e., 34 paired datasets) as the unit of analysis. At no point did we intend to equate 34 dyads with 34 individuals. We have now standardized terminology throughout the manuscript to ensure a clear distinction between individual-level single-brain measures and dyad-level inter-brain measures.

We sincerely appreciate this important methodological clarification, which has substantially improved the precision and readability of our manuscript.

---

(12) 'two experimenters of the same gender were randomly formed into pairs as sender and receiver, respectively' – I don't follow, what was the role of experimenters in the pairs? Was one of the alleged participants a confederate?

Re: We thank the reviewer for pointing out that this wording could be misleading. The phrase "two experimenters" in the original manuscript was an inappropriate expression resulting from a translation error. The experimenters did **not** participate in the task as dyad members, and there was no participant–confederate design. All dyads consisted of two genuine participants (same gender, previously unacquainted). The role of the experimenters was strictly limited to organizing and supervising the procedure—for example, confirming that participants had not met before, explaining the task rules, monitoring timing windows, and, in rare cases, terminating speech that exceeded the allotted time. They did not provide any task-related informational input. To prevent readers from interpreting this as experimenters entering the dyad as sender/receiver, we have revised the wording to state more precisely that participants were randomly paired by the experimenter and randomly assigned to the roles of sender/cheater and receiver/detector, with roles remaining fixed throughout the experiment. The experimenters did not participate in interaction or decision-making.

Please see the revised manuscript, page 10, lines 181 to 182, or the highlighted blue text:

Roles (sender/cheater vs. receiver/detector) were randomly assigned within each dyad and remained fixed throughout the experiment.

Please see the revised manuscript, page 9, lines 170 to 171, or the highlighted blue text:

Initially, 37 same-gender dyads composed of previously unacquainted individuals were formed from eligible participants.

Please see the revised manuscript, page 9, line 175 to page 10, line 180, or the highlighted blue text:

Prior to the experiment, experimenters confirmed that members within each dyad had no prior acquaintance and were meeting for the first time. To further validate the effectiveness of the stranger-dyad manipulation, participants completed the single-item Inclusion of Other in the Self (IOS) scale to assess perceived interpersonal closeness (Likert 7 -point scale, range from 1 to 9). All participants reported IOS scores below 2 (M=1.04, SD=0.21), indicating successful manipulation of stranger relationships.

We appreciate the reviewer's careful attention to this important ambiguity and have standardized the terminology throughout the manuscript to ensure clarity and transparency of the design.

---

(13) 'Two participants were seated face to face in an oblique diagonal orientation, with a face computer blocking their view' – this needs clarification. If the participants could not see each other due to a screen blocking their view, how is this task still face to face? Which cues were participants expected to use to detect deception, voice only?

Re: We sincerely apologize for any confusion this may have caused. We recognize that the description in our earlier draft was somewhat brief, which may have led to inconsistencies in interpretation. We would like to clarify the rationale more explicitly. The angled placement of the monitors was intentionally designed to allow participants to observe each other's facial expressions and gaze without obstruction, thereby preserving the possibility of natural eye contact during interaction. This setup was crucial for maintaining ecological validity in a dyadic interactive task, particularly in a spontaneous deception paradigm where contextual realism is essential. Moreover, this configuration is consistent with experimental arrangements commonly adopted in hyperscanning studies conducted under naturalistic interaction settings (e.g., Li et al., 2025; Zhang et al., 2024; Chen et al., 2020). At the same time, we were mindful of avoiding prolonged, rigid, or unnatural staring behavior. In laboratory contexts, such gaze patterns can arise due to task pressure and may distort genuine interpersonal neural responses (Einhäuser & Hart, 2014; Hart & Einhäuser, 2012). Our intention was not to prohibit eye contact, but rather to create a setting that more closely approximates real-life interaction, enabling participants to engage in gaze exchange in a flexible, spontaneous, and socially meaningful manner.

Please see the revised manuscript, page 11, lines 201 to 208, or the highlighted blue text:

Setup. The experiment was conducted in a fNIRS laboratory. Dyads were seated face-to-face at a diagonal angle, with monitors positioned diagonally between them. This configuration allowed participants to naturally access each other's facial and nonverbal cues (e.g., facial expressions and gaze direction) while performing the task, without inducing prolonged, fixed, or socially meaningless eye contact. Importantly, the monitors did not fully occlude the participants' view of one another. Therefore, the interaction retained face-to-face characteristics, while minimizing excessive social arousal that arise from direct gaze. This setup was designed to preserve ecological validity at the interpersonal level while maintaining experimental control (see Error! Reference source not found.**C**).

We have revised the corresponding section of the manuscript to more accurately convey this design rationale. In addition, during the experiment we recorded instances of direct eye contact that naturally occurred between participants. We hope this clarification addresses your concerns regarding the experimental setup, and we appreciate your thoughtful inquiry.

Reference

[1] Li, Y., Wang, Y. J., Su, C., Deng, F., & Pan, Y. Bidirectional information flow in cooperative learning reflects emergent leadership. *Communications Biology*, *8*(1), 1000.

[2] Zhang, M., Yin, Z., Zhang, X., Zhang, H., Bao, M., & Xuan, B. (2024). Neural mechanisms distinguishing two types of cooperative problem-solving approaches: an fnirs hyperscanning study. *NeuroImage*, *291*, 120587.

[3] Chen, M., Zhang, T., Zhang, R., Wang, N., Yin, Q., Li, Y. & Li, X. (2020). Neural alignment during face-to-face spontaneous deception: Does gender make a difference? *Human brain mapping*, *49*(17), 64-81.

[4] Hart, B., & Einhäuser, W. (2012). Mind the step: complementary effects of an implicit task on eye and head

movements in real-life gaze allocation. *Experimental Brain Research*, *223*, 233-249.

[5] Einhäuser, W., & Hart, B. (2014). Eye movements in natural scenes and gaze in the real world. *Journal of Vision*, *14*(10), 1446-1458.

---

(14) Were both participants aware of the nature of the payoff matrices, i.e. the fact that a better outcome for the receiver always meant a worse outcome for the sender? If so, this would likely prompt receivers to not believe information provided by the sender. If not, did the receiver have any reason to anticipate deception?

Re: We thank the reviewer for raising this important question, as it directly concerns the strategic structure of the task and the mechanism by which receivers form beliefs.

First, both participants were explicitly informed that the payoff structure was strictly zero-sum and conflict-based: in each trial, a higher payoff for the sender necessarily corresponded to a lower payoff for the receiver, and vice versa. We also clearly stated that misleading or deceptive communication was permitted in the task. Therefore, receivers had theoretical grounds to anticipate potential deception. Crucially, however, receivers could not view the numerical payoff matrix. In other words, they were aware of the conflict of interest and the possibility of deception, yet lacked verifiable information at the trial level. This design intentionally created a situation of "motivational transparency but informational asymmetry." At the motivational level, receivers knew that senders had self-interested incentives. At the informational level, however, receivers could not independently determine which option was objectively advantageous. As a result, receivers' decisions were not reducible to a simple default distrust strategy. Instead, they were required to evaluate, under limited information, whether the sender was indeed deceiving them, whether a cooperative strategy might be in play, and whether linguistic cues, nonverbal signals, or interaction dynamics should guide their judgment. This structure was further reinforced by the fact that senders received no feedback regarding whether their deception was successful, thereby preventing learning or reputation formation typical of repeated-game settings.

Please see the revised manuscript, page 13, lines 252 to 263, or the highlighted blue text:

In each trial, the payoff structure was explicitly designed to induce a direct conflict of interest between the sender and receiver: a higher payoff for the sender necessarily corresponded to a lower payoff for the receiver, and vice versa. This structure was identical in both gain and loss contexts. Importantly, only one option in each trial was objectively advantageous for the receiver (i.e., higher gain or lower loss), whereas the alternative option was disadvantageous. This design has been shown to effectively elicit spontaneous information manipulation based on immediate payoff trade-offs (Chen et al., 2020). Participants were explicitly informed that deceptive or misleading

communication was permitted in the task. Receivers could not view the numerical values in the outcome matrix and relied solely on the sender's recommendation cues (color signals and verbal statements), rendering direct verification of information impossible (Huang et al., 2025). Meanwhile, senders received no feedback during or after the experiment regarding whether their information successfully influenced the receiver's decision.

Precisely because receivers were aware of the conflict but lacked verification channels, the interaction depended on dynamic cue integration and strategic inference rather than rule-based rejection. This context also provides important background for our behavioral finding that successful deception rates were higher in loss contexts. The loss frame may have increased the psychological weight of loss avoidance, leading receivers to adopt recommendations more quickly, as reflected in both behavioral frequency and reaction time results.

We appreciate the reviewer's theoretically insightful question, which has allowed us to clarify and better articulate the core logic underlying our task design.

Reference

[1] Huang, R., Gao, X., Zhang, C., …, & Liu, Y. (2025). Forewarned Is Forearmed: The single- and dual-brain mechanisms in Detectors from Dyads of Varying Social Distance During Deceptive Outcomes Evaluation. The *Journal of neuroscience*, *45*(43): e2129242025.

[2] Chen, M., Zhang, T., Zhang, R., ..., & Li, X. (2020). Neural alignment during face-to-face spontaneous deception: does gender make a difference? *Human brain mapping*, *41*(17), 4964-4981.

---

(15) Why were dyads with high honesty rates excluded from analysis of behaviour? This would skew the results.

Re: We understand that, without sufficient justification, excluding high-honesty dyads could be interpreted as introducing selective bias. Therefore, in the revised manuscript, we have further clarified the theoretical rationale and methodological necessity of this procedure. First, the central aim of this study is to characterize the neural mechanisms underlying active deceptive interaction, rather than to compare personality tendencies toward honesty versus deception. **As stated in the revised manuscript (page 26, lines 503 to 506)**: "Dyads characterized by persistently honest recommendations primarily reflect stable moral compliance or risk-avoidant tendencies, rather than engagement in active deceptive interaction. Such behavioral patterns do not permit meaningful modeling of the psychological processes underlying deception." Following the exclusion criterion adopted by Chen et al. (2020), when a dyad selected the objectively receiver-advantageous option in the vast majority of trials (>85%), their behavior did not meaningfully enter a strategic deception game state. Under such circumstances:

(1) The number of deception trials was extremely limited, rendering trial-level INS or GLM estimates unreliable—particularly given that our analytic focus centers on deception trials;

(2) INS analyses require comparisons within the same psychological state category (i.e., deception trials) across gain–loss contexts. When deception is nearly absent, comparable conditions cannot be established.

Importantly, this exclusion criterion was predefined, based on prior hyperscanning research (Chen et al., 2020), and applied uniformly across all conditions, rather than being adjusted post hoc in a results-driven manner.

Please see the revised manuscript, page 17, lines 333 to 334, or the highlighted blue text:

To avoid bias in subsequent INS analyses caused by an insufficient number of deceptive trials, dyads with an honesty rate exceeding 85% were excluded (Chen et al., 2020).

More critically, our study does not claim that "everyone deceives." Rather, it investigates how the neural mechanisms of deception are modulated by contextual framing when deception occurs. Accordingly, the appropriate unit of analysis must be dyads that actually engaged in deceptive interaction. Including dyads that almost never engaged in deception would artificially inflate the proportion of zero values in the ΔINS model, thereby reducing sensitivity to the neural mechanisms underlying genuine deceptive behavior.

We thank the reviewer for highlighting the potential concern regarding bias. To enhance transparency, we have clarified in the revised manuscript that this criterion was methodologically motivated and predefined, rather than applied as a post hoc selection.

Reference

[1] Chen, M., Zhang, T., Zhang, R., ..., & Li, X. (2020). Neural alignment during face-to-face spontaneous deception: does gender make a difference? *Human brain mapping*, *41*(17), 4964-4981.

---

(16) 'the percentage of all dyads that were deceived by the sender' – do the authors mean of all trials?

Re: We thank the reviewer for pointing out that this phrasing may have led to ambiguity. The metric refers to the proportion of deceptive trials in which the receiver ultimately adopted the sender's recommendation, thereby yielding a more favorable outcome for the sender (i.e., the successful deception rate), or to the proportion of trials in which the sender issued a recommendation that resulted in an objectively disadvantageous outcome for the receiver (i.e., the deception rate). To prevent confusion, we have standardized the terminology in the revised manuscript as "proportion of trials" and have clearly specified the computation method in the *Behavioral Data Analysis* section.

Please see the revised manuscript, page 17, lines 331 to 337, or the highlighted blue text:

Based on these definitions, four primary behavioral indices were computed: **(1)Honesty rate.** The proportion of trials in which the sender provided truthful and detector-beneficial information. To avoid bias in subsequent INS analyses caused by an insufficient number of deceptive trials, dyads with an honesty rate exceeding 85% were excluded (Chen et al., 2020). **(2) Deception rate.** The proportion of trials in which the sender provided information that was objectively disadvantageous to the receiver. **(3) Successful deception rate.** The proportion of deceptive trials in which the receiver ultimately obtained a lower gain or incurred a higher loss.

Reference

[1] Chen, M., Zhang, T., Zhang, R., ..., & Li, X. (2020). Neural alignment during face-to-face spontaneous deception: does gender make a difference? *Human brain mapping*, *41*(17), 4964-4981.

---

(17) Some of the results are reported in the methods section, this should be corrected.

Re: We thank the reviewer for identifying this structural issue. Upon re-examining the manuscript, we found that a few statements in the *Methods* section indeed contained result-oriented language, particularly in the description of frequency band selection and analysis procedures, where wording approached the tone of reporting findings.

In response to your suggestion, we have revised the manuscript accordingly:

(1) The *Methods* section now includes only technical descriptions of the analytical procedures and statistical criteria.

(2) All statements referring to statistical significance, effect direction, or specific numerical results have been relocated to the *Results* section.

(3) In cases where it was necessary to explain the rationale for certain analytical choices, we have adopted neutral phrasing (e.g., "frequency bands identified in subsequent analyses") to avoid prematurely disclosing statistical outcomes.

Through these revisions, the conceptual and structural boundaries between the *Methods* and *Results* sections have been clearly delineated. We appreciate your guidance in helping us improve the manuscript's structural clarity and methodological rigor.

---

(18) ***Results:*** *Figure 2: this is a bar graph, not a histogram as stated. A histogram would be more informative.*

Re: In accordance with the editor's and reviewers' comments, we have redrawn and revised Figure 2. The figure has been changed from a bar plot to a boxplot to more comprehensively present the distributional characteristics of the data, including the median, interquartile range, full range of values, and the dispersion of individual data points. Compared with bar plots, boxplots provide a clearer visualization of distributional shape and within-group variability, thereby enhancing both transparency and informational richness in the presentation of results. We have also corrected the corresponding descriptions in both the figure legend and the main text to consistently refer to the figure as a "boxplot" rather than a "histogram."

The revised figure can be found in the revised manuscript (page 27, lines 526 to 531).

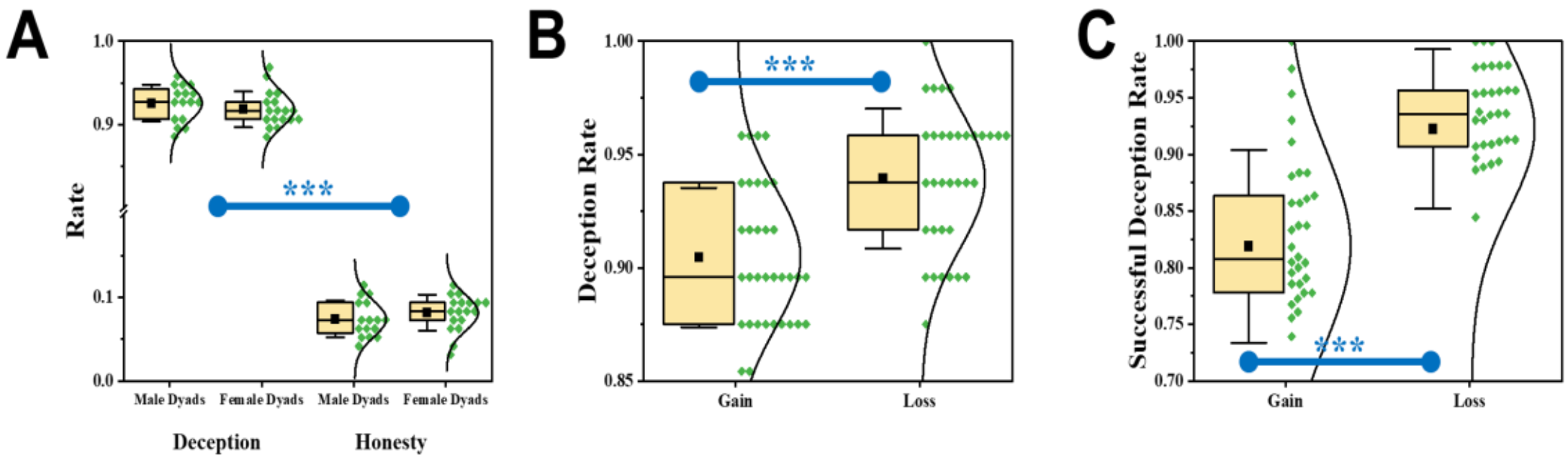

Figure 2. Behavioral results of deception. (A) Proportion of behavioral occurrence (honesty vs. deception). **(B)** Deception rate across gain and loss contexts. **(C)** Successful deception rate across gain and loss contexts. Green diamonds represent individual data points; black squares denote condition means; lines within boxes indicate medians; error bars represent ±1SD; outer contours depict normal distribution curves. *** p< 0.001. The same conventions apply in subsequent figures.

---

(19) The fNIRS data analysis mostly ignores the main variable of interest, deception vs. honest reporting. Why is this not analysed on a trials-wise basis? Simply comparing gain vs. loss contexts while ignoring trial outcomes means it is impossible to say whether differences in neural activity patterns are due to gain vs. loss contexts, or differences in deception rates between the conditions.

Re: We sincerely thank the reviewer for this important and constructive suggestion. While task-level INS analyses provide stability and capture overall trends, trial-level INS analyses allow for the examination of finer-grained, real-time neural dynamics, thereby offering deeper insight into how individuals detect deception during each interaction. Accordingly, in this revision, we have added a new analysis to complement our original task-level approach (which integrated multiple trials to explain overall successful deception rates): a machine-learning–based trial-by-trial deception prediction analysis.

For the trial-level prediction analysis, INS features were extracted from the 1-15 s verbal interaction window of each trial. However, given the inherent instability of single-trial fNIRS signals and their susceptibility to motion artifacts (Brigadoi & Cooper, 2015; Pfeifer et al., 2018), we adopted a preprocessing pipeline suitable for trial-level fNIRS data, following the procedures proposed by Klein and Kranczioch (2019). Specifically, this included: (1) band-pass filtering at 0.01–0.1 Hz, (2) motion artifact correction using a wavelet minimum description length (MDL) algorithm, (3) baseline drift correction, and (4) global component removal (GCR).

Subsequently, INS features were Fisher Z-transformed and subjected to dimensionality reduction. Principal components explaining more than 85% of the cumulative variance were retained for modeling. Trial-level deception outcomes (0= successful deception, 1= failed deception) were used as the target variable. Classification analyses were conducted using Logistic Regression and Support Vector Machines (SVM), with leave-one-subject-out cross-validation (LOSO-CV) implemented to ensure model generalizability and prevent inter-subject information leakage.

Details of this analytical procedure can be found in the revised manuscript (page 20, line 385 to page 21, line 414), or in the highlighted blue text provided therein.

Trial-level ΔINS Prediction of Deception Outcomes. To systematically examine the dynamic predictive value of trial-level ΔINS during deceptive interactions, ΔINS was computed on a single-trial basis from fNIRS signals and used to predict deception outcomes in the corresponding trials. Given the susceptibility of single-trial fNIRS signals to noise, we followed established procedures to enhance signal quality and stabilize INS feature extraction (Huang et al., 2025). Specifically, **(1)** raw optical density signals were band-pass filtered at 0.01–0.1Hz to preserve task-relevant low-frequency neural oscillations while removing high-frequency physiological noise; **(2)** motion artifacts were corrected using the wavelet minimum description length algorithm; **(3)** baseline drift was corrected using a 5-s pre-stimulus baseline for each trial; and **(4)** global component removal was applied to eliminate system-level components shared across channels.

After preprocessing, fNIRS data were segmented according to event markers, and signals during the verbal interaction phase (1-15 s) of each trial were extracted for ΔINS computation. Given the substantial variability inherent in single-trial fNIRS data (Brigadoi & Cooper, 2015; Pfeifer et al., 2018), analyses were restricted to interpersonal channels that exhibited significant task-level INS enhancement, thereby improving the stability and interpretability of trial-level estimates. Trial-level ΔINS was computed using the same method as in the task-level analysis and was extracted separately for successful and failed deception trials. To mitigate overfitting due to redundant features, trial-level ΔINS values were first Fisher z-transformed and then subjected to PCA. Principal components explaining more than 85% of the cumulative variance were retained for subsequent modeling (van Aert, 2023). Deception outcome at each trial (binary: successful deception= 0, failed deception= 1) was used as the dependent variable, with trial-level ΔINS feature vectors serving as predictors. Logistic regression was employed as

the primary model to assess the linear predictive relationship between ΔINS and deception outcomes. In addition, Support Vector Machine (SVM) models were implemented to evaluate potential nonlinear associations between ΔINS and deception outcome.

All models were evaluated using a leave-one-subject-out cross-validation (LOSO-CV) scheme to ensure robust generalization across participants (Xu & Huang, 2012). Model performance was quantified using **(1)** accuracy, **(2)** area under the receiver operating characteristic curve (ROC-AUC), and **(3)** F1 score. To determine whether model performance exceeded chance levels, permutation testing with 1,000 label shuffles was conducted.

This analysis employed Logistic Regression and Support Vector Machine (SVM) models, both of which are widely used for predictive modeling in binary classification tasks (Zhang et al., 2025; Arora et al., 2018; Hua et al., 2007). The results indicated that the Logistic Regression model significantly outperformed the SVM model in prediction accuracy under both contextual conditions ($ps < 0.001$), thereby confirming the predictive validity of trial-level INS features.

A detailed comparison of the final models, including performance metrics and validation results, can be found in the revised manuscript (page 33, line 629 to page 35, 647), or in the highlighted blue text provided therein.

Trial-Level ΔINS Predicts Deception Outcomes

To evaluate the predictive value of trial-level ΔINS during deceptive information processing, we applied two classification algorithms—Logistic Regression and SVM—to examine whether ΔINS could predict deception outcomes (successful vs. failed deception) at the trial level. Results indicated that, under both gain and loss contexts, the Logistic Regression model performed significantly above chance. Model performance was best in the loss context (Accuracy=0.884, AUC=0.611, F1 score=0.638). Performance metrics for all conditions and models are summarized in **Table 6** and **Figure 7**. To further assess the statistical robustness of model performance, we conducted 1,000 permutation tests. The Logistic Regression model significantly outperformed the null distribution generated by label shuffling ($ps<0.001$), confirming that the observed predictive accuracy was unlikely to arise by chance.

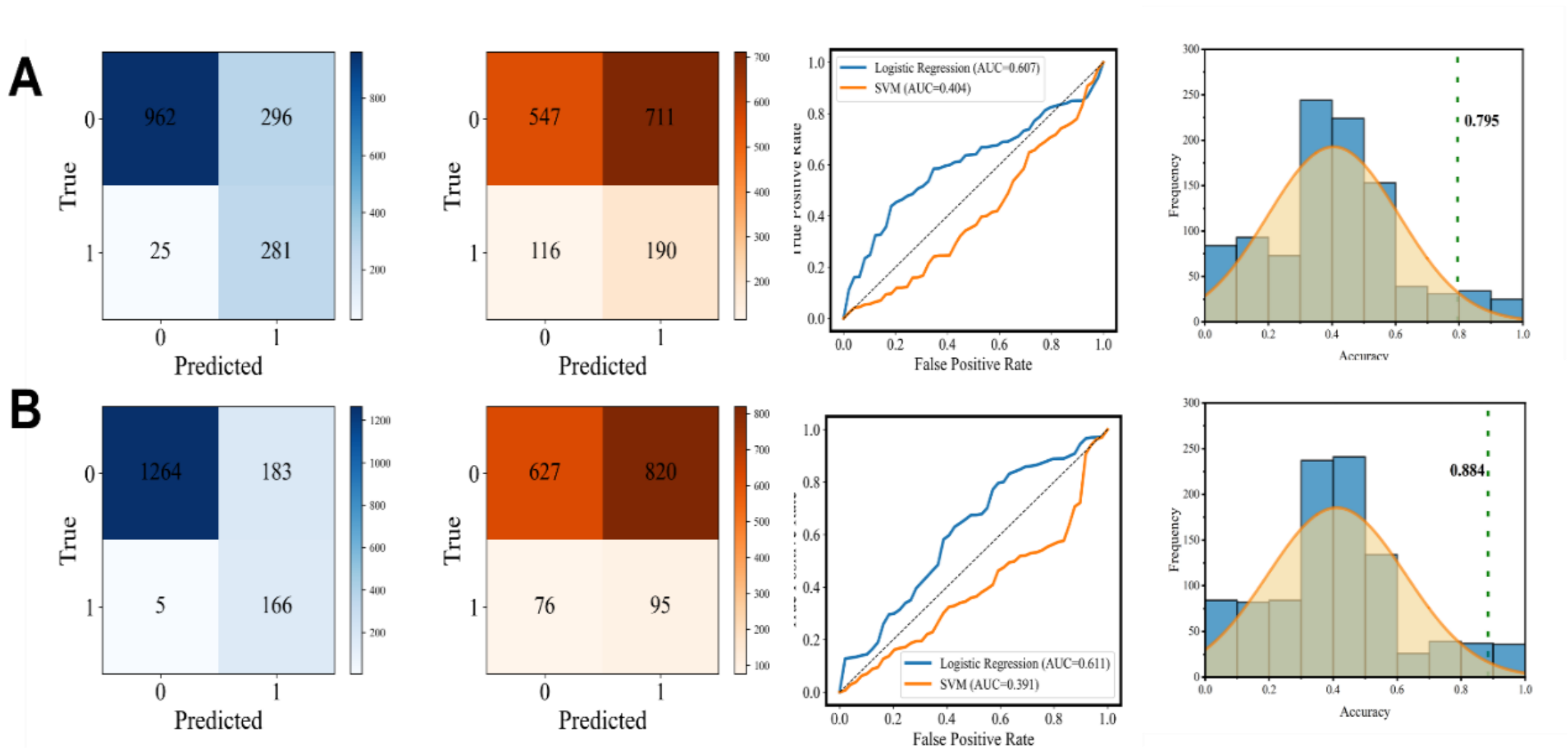

Figure 7. ML performance in predicting deception outcomes based on trial-level ΔINS. (A) Gain context; **(B)** Loss context. From left to right: **(1)** Confusion matrix for Logistic Regression (0=successful deception; 1=failed deception); **(2)** Confusion matrix for SVM; **(3)** ROC-AUC curves for both models (training phase), showing superior performance of Logistic Regression across contexts; **(4)** Permutation test results (1,000 iterations) for Logistic Regression, where the green dashed line indicates observed accuracy and the yellow curve represents the fitted null distribution.

Table 6. Performance evaluation of two ML algorithms across context.

Model	Condition	ROC-AUC	Accuracy	Precision	Recall	F1 Score
Logistic Regression	Gain	0.607	0.795	0.487	0.918	0.636
	Loss	0.611	0.884	0.476	0.971	0.638
SVM	Gain	0.404	0.471	0.211	0.621	0.315
	Loss	0.391	0.446	0.104	0.556	0.175

---

(20) For the behavioural data, behaviour of the recipients needs to be analysed. Did receivers generally believe the information they were given?

Re: We thank the reviewer for raising this important behavioral-level concern. We fully agree that reporting only the cheaters' deception rate is insufficient; the behavioral responses of the detectors are an equally critical component of the interactional mechanism. In fact, we have already incorporated analyses of detector behavior in the revised manuscript. In the present task, whether the detector "believed" the cheater's information was operationally defined as whether the detector selected the option recommended by the cheater during the final decision phase. This metric is referred to as "recommendation adoption" in the manuscript and, within deceptive trials, is further specified as "successful deception."

The key results are reported in the *Results* section (see revised manuscript, page 27, lines 520 to 525, or the subsequent blue text):

Beyond sender behavior, we examined detectors' trial-level acceptance of the cheater's recommendation. Detector acceptance was operationally defined as selecting the recommended option during the final decision phase (i.e. successful deception). A paired-samples *t* test showed that successful deception rates were significantly higher under loss than gain contexts [loss vs. gain: 0.92±0.07 vs. 0.82±0.09, $t_{(33)}$=-5.304; p<0.001, *Cohen'd*=-0.910, **Figure 2C**], indicating that detectors were more likely to adopt the cheater's recommendation in loss contexts.

These findings indicate that detectors were more likely to adopt the cheater's recommendation under loss conditions. In other words, within a loss frame, detectors were more inclined to "believe" or at least to follow the information provided by the cheater.

In addition, we analyzed detectors' reaction times (RTs) during the final decision stage (see revised manuscript, page 28, line 549 to page 29, line 562, or the subsequent blue text):

We further examined detectors' RT during the final decision phase to characterize their decision-making dynamics under different contextual and recommendation conditions. A 2 (context: gain vs. loss)×2 (recommendation adoption: adopted vs. rejected) repeated-measures ANOVA revealed a significant main effect of context, with overall shorter RT in loss compared to gain contexts [loss vs. gain: 2058.96±38.67 vs. 2209.68±46.18, $F_{(1,33)}$=5.125, p=0.030, η_p^2=0.134]. A significant main effect of recommendation adoption was also observed, indicating that RT were shorter when detectors adopted the cheater's recommendation (2056.82±39.34) than when they rejected (2211.82±43.87) it [$F_{(1,33)}$=5.829, p=0.021, η_p^2=0.150]. Importantly, the interaction between context and recommendation adoption was significant [$F_{(1,33)}$=6.024, p=0.020, η_p^2=0.154]. Simple-effects analyses showed that in loss contexts, RT were significantly shorter when recommendations were adopted compared to rejected (adopted vs. rejected: 1912.96±47.80 vs. 2204.96±67.52, post hoc p=0.002, *95%CI* [113.406, -470.599]). No significant difference between adoption (2200.68 ± 63.69) and rejection (2218.68± 59.93) was observed in gain contexts (post hoc p= 0.828, *95% CI* [−149.387, 185.398]). These results are illustrated in **Figure 3B**.

We sincerely appreciate the reviewer's suggestion to strengthen the interpretation of bidirectional behavioral dynamics. This has enabled us to present a more comprehensive account of the reciprocal decision-making mechanisms underlying social interaction.

---

(21) This should also be accounted for in the interbrain synchronisation analysis: was synchrony highest in trials where no deception was included, and the receiver believed the information they were given?

Re: Thank you for your comment. In the revised manuscript, we have explicitly distinguished among three types of trials—honesty, failed deception, and successful deception—in the task-level ΔINS analyses. The corresponding results can be found in the revised manuscript **(page 32, line 613 to page 33, line 628)**.

During successful deception trials, ΔINS between bilateral DLPFC channels (CH14–CH14) was significantly modulated by context. Specifically, ΔINS was significantly higher in the loss context compared to the gain context [loss vs. gain: 0.44±0.12 vs. 0.22±0.12, $t_{(33)}$=-8.656, p<0.001, *Cohen's d*=-1.485, **Figure 6A&B**]. Importantly, no significant contextual differences were observed in the same channel pair during honesty trials [loss vs. gain: 0.07±0.05 vs. 0.07±0.04, $t_{(33)}$=0.054, p=0.957, *Cohen's d*=-0.055, **Figure 6D**] or failed deception trials [loss vs. gain: 0.22±0.11 vs. 0.22±0.09, $t_{(33)}$=-0.320, p=0.751, *Cohen's d*=0.009, **Figure 6C**]. These results indicate that framing-related modulation of ΔINS was specific to successful deception. Permutation testing (1,000 iterations) confirmed that the empirical t-value exceeded the null distribution (ps < 0.001, **Figure 6E**), indicating that the effect was not driven by random pairing.

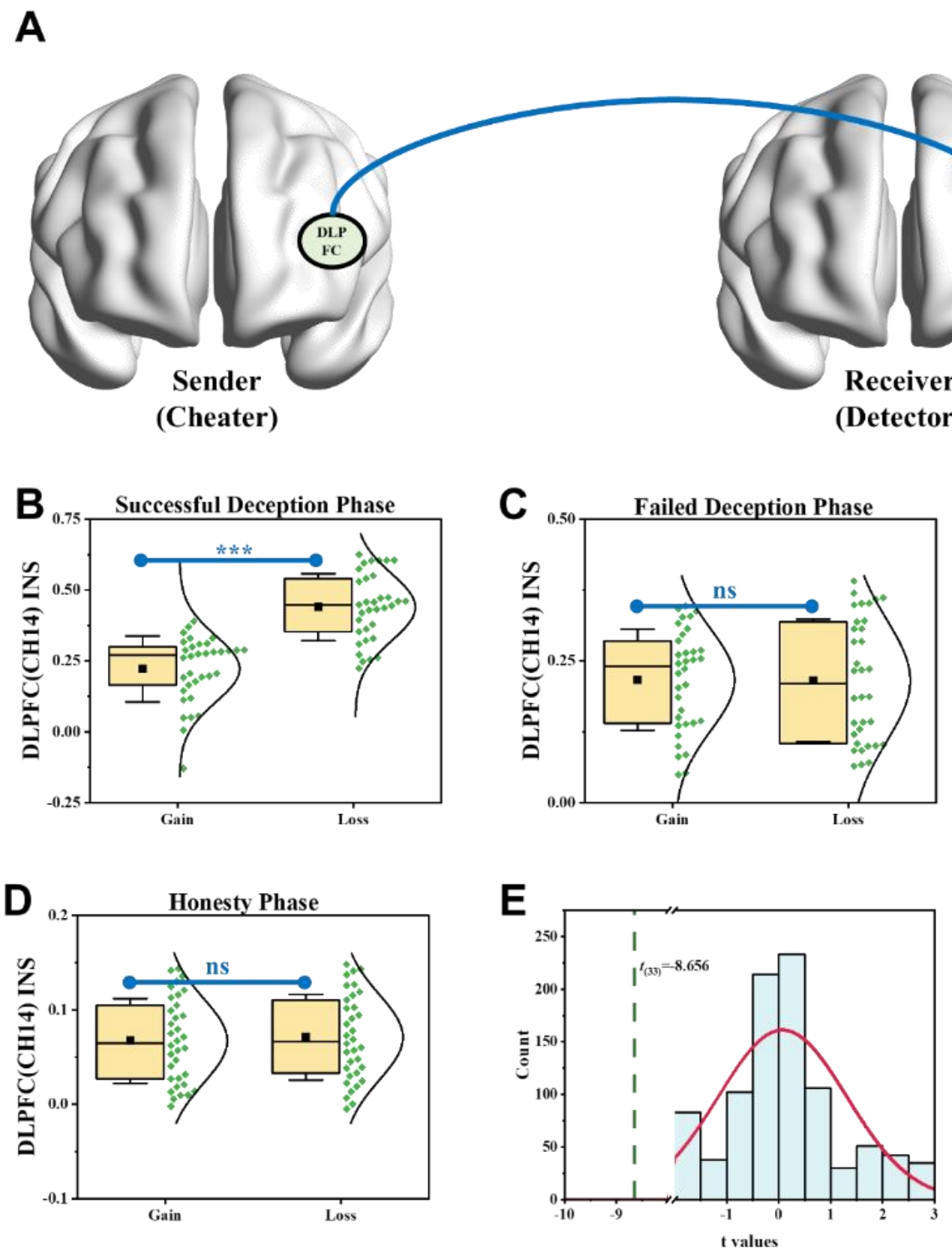

Figure 6. Task-Level ΔINS Results. (A) Visualization of channel pairs exhibiting significant contextual differences in ΔINS. **(B)** Comparison of ΔINS in channel pairs showing significant contextual differences during successful deception trials. **(C)** Comparison of ΔINS in the same channel pair during failed deception trials across contexts. **(D)** Comparison of ΔINS in the same channel pair during honesty trials across contexts.

The key points are as follows: **(1)** In honesty trials (i.e., no deception and truthful information), no significant enhancement of DLPFC ΔINS was observed. **(2)** In failed deception trials (i.e., when the receiver did not adopt the recommendation), no significant contextual modulation was detected. **(3)** A significant increase in ΔINS emerged exclusively in successful deception trials, and this effect exhibited a clear context-dependent pattern.

Thus, interpersonal synchrony did not reach its highest level under conditions of "no deception but belief."

Instead, the strongest ΔINS was observed during successful deceptive interactions, rather than in situations characterized merely by trust or informational congruence. These findings suggest that enhanced DLPFC synchrony does not simply reflect “receiver belief” or general interactional alignment. Rather, it more likely indexes the alignment of control-related systems during successful strategic regulation.

In the revised manuscript, we have further clarified this logical distinction to prevent ΔINS from being misinterpreted as a general indicator of trust. We sincerely appreciate the reviewer’s insightful theoretical distinction, which has helped us more precisely delineate the functional significance of inter-brain synchrony.

Reviewer #3:

SUMMARY: *This study uses dyadic hyperscanning to investigate how gain-loss contexts influence deception and the associated neural mechanisms. The authors found that senders were more likely to deceive in loss contexts, suggesting that the prospect of loss reduces moral restraint and makes deception more automatic. Neurally, senders exhibited increased dorsolateral prefrontal cortex (DLPFC) activation during conflict scenarios in gain contexts, indicating greater cognitive control and moral deliberation when both parties could benefit. During sender–receiver interactions in gain contexts, interpersonal neural synchrony (INS) in the DLPFC increased; conversely, reduced INS in loss contexts was associated with higher deception rates. The study also showed that INS could predict deception rates in loss contexts using least squares regression, highlighting the potential of neural markers to forecast dishonest behaviour.*

Overall, the paper provides important insights into how context shapes behavioural and neural aspects of deception. While innovative in combining behavioural, neural and predictive analyses in an interesting dyadic, face-to-face environment, the manuscript could be improved before publication in Communications Biology by addressing the lack of methodological detail and improving the clarity and structure of certain sections.

Re: We sincerely thank Reviewer #3 for the thoughtful and constructive evaluation of our manuscript. We appreciate your clear summary of the study's contributions and your recognition of the value of combining behavioral, hyperscanning, and predictive modeling approaches within a face-to-face dyadic paradigm. Your comments have substantially improved the clarity, transparency, and structural coherence of the manuscript. In response to your suggestions:

- **Abstract clarity and structure**

We have revised the abstract to explicitly specify the hyperscanning modality (functional near-infrared spectroscopy, fNIRS), clearly define key constructs (e.g., deception, interpersonal neural synchrony), and provide balanced methodological details, including sample size and modeling strategy.

- **Power analysis and sample transparency**

We clarified the rationale for the assumed effect size (f= 0.25), reported the full recruitment-to-analysis flow, and explicitly stated exclusion criteria and final sample composition to eliminate ambiguity.

- **Behavioral paradigm clarification**

We clarified that roles (sender vs. receiver) were fixed throughout the task and provided precise operational definitions for honesty rate, deception rate, and successful deception rate.

■ **Subjective measures**

We detailed the interpersonal closeness (IOS) and trait-level measures collected, while explaining the rationale for not introducing trial-level self-reports to preserve ecological validity and uninterrupted interaction dynamics.

■ **fNIRS temporal dynamics**

We added trial-level ΔINS time-series analyses to directly address the temporal evolution of interpersonal neural synchrony during successful versus failed deception.

■ **Brain–behavior relationships**

We now report correlations separately for gain and loss contexts and clarify the contextual modulation of ΔINS–behavior coupling.

■ **Machine learning rationale and robustness**

We clarified that the predictive modeling approach extends beyond correlational analysis by testing cross-validated generalizability. We adopted LOOCV and permutation testing to provide conservative and transparent estimates given the modest sample size.

■ **Response time analyses**

We incorporated detailed RT analyses for both senders and receivers, demonstrating that deception decisions are executed faster in loss contexts, thereby strengthening the behavioral interpretation.

■ **Discussion structure**

We have reorganized and streamlined the Discussion, added clearer transitions and summary statements, and reduced redundancy to improve logical flow and readability.

Once again, we are grateful for your careful reading and constructive guidance. Your comments have helped us substantially enhance the methodological transparency, conceptual precision, and structural clarity of the manuscript.

You can refer to the specific revisions and responses in the following sections.

---

(1) Abstract: While I found the paper to be generally concise and well-written with a clear structure, I believe the abstract could be improved in terms of clarity and structure. For example, the type of hyperscanning performed is not specified (EEG, NIRS or fMRT?), and several terms are not clearly defined (e.g. interpersonal neural synchrony; what does deception mean in this context?). Furthermore, while some sections provide methodological details (e.g. least squares regression), others lack key information (e.g. sample size).

Re: Thank you very much for your constructive comments regarding the clarity and structure of the abstract.

In response to your suggestion, we have revised the abstract accordingly. First, at the methodological level, we now explicitly state the neuroimaging modality employed to prevent confusion with EEG- or fMRI-based hyperscanning approaches. The revised manuscript **(page 1, lines 8-11)** now reads: "we recruited 34 same-gender stranger dyads (18 female dyads) and employed functional Near-infrared Spectroscopy hyperscanning to simultaneously record Prefrontal activity while participants engaged in a face-to-face sender (cheater)-receiver (detector) paradigm under gain and loss contexts."。

Second, we have provided clear operational definitions of the core constructs to enhance conceptual transparency. For example, deception is now explicitly defined as: "Deception was operationalized as a cheater's recommendation that was objectively disadvantageous to the detector" **(see the revised manuscript, page 8, lines 11-12)**. This formulation avoids framing deception as a purely subjective intention and instead anchors it within the objective gain-loss structure of the task. Likewise, interpersonal neural synchrony is no longer presented as an abstract construct; rather, it is defined in relation to task-related inter-brain coupling within specific neural regions and contextual conditions. As stated in the revised abstract**(see the revised manuscript, page 1, lines 14-17)**: "during successful deception trials, loss contexts were associated with significantly higher task-related increases in interpersonal neural synchrony (ΔINS) between bilateral DLPFC regions, whereas honesty and failed deception showed no comparable contextual modulation."

Additionally, to address the issue of uneven presentation of methodological details, we have optimized the revision. We retained the predictive modeling information that is directly relevant to the study's contributions and ensured that it logically connects with the main findings, rather than isolating technical terms. For example, in the revised manuscript **(page 1, lines 17 to 19)**, you can find the following text: "Across trial-level analyses, brain-behavior correlations, and machine-learning models, DLPFC ΔINS robustly predicted individual differences in successful deception." This formulation emphasizes the consistency across methodologies and the robustness of the predictions, rather than focusing on any single algorithm, thereby enhancing the logical coherence and focus of the abstract.

Finally, thank you once again for your detailed review of the abstract's precision and structural balance. Your feedback has significantly improved the clarity and self-consistency of the abstract, making it better suited to meet the expectations of interdisciplinary readers.

---

(2) Power analysis: How do the authors justify their assumption of an effect size of $f = 0.25$? I also wondered what the final sample size analysed was. It states that 34 pairs of participants were analysed. However, it is later stated that dyads with deception rates greater than 85% were excluded.

How many participants were excluded and for which reasons?

Re: Thank you for raising these important questions regarding the power analysis and sample composition. These issues are directly related to the statistical validity and transparency of the study design, and we have clarified them more explicitly and systematically in the revised manuscript.

Regarding the choice of effect size (f = 0.25), this value corresponds to a medium effect size as defined by Cohen (1988), which is commonly adopted in repeated-measures or paired designs when prior evidence suggests moderate effects. Before initiating the study, we consulted previous hyperscanning research using comparable sender–receiver deception paradigms (e.g., Chen et al., 2020; Huang et al., 2025), in which both behavioral and neural indices typically showed medium to relatively large effects. Considering that (1) the present study employed a dyadic paired design and (2) the primary analyses focused on within-dyad contextual differences (gain vs. loss), we selected f = 0.25 in G*Power as a conservative and theoretically justified estimate.

Please see the revised manuscript, page 9, lines 168 to 170, or the highlighted blue text:

A priori power analysis was conducted using G*Power 3.1.9.7 (Faul et al., 2007) (medium effect size f=0.25, α=0.01, 1-β=0.80, paired-samples t-test), indicating a minimum required sample size of 34 dyads.

With respect to the final sample size and exclusions, we have clearly distinguished between the initially recruited sample and the final analyzed sample in the revised manuscript. The exclusion criterion was predefined as a deception rate < 85%. Two dyads were excluded because, in the vast majority of trials, the sender selected options objectively beneficial to the receiver, indicating that they rarely engaged in active deceptive interaction. Because the core analyses of this study—particularly task-level INS and trial-level INS prediction—require a sufficient number of deceptive trials to obtain stable neural estimates, these dyads could not provide reliable data for modeling deception-related processes and were therefore excluded according to the predefined criterion. Importantly, this criterion was established prior to data analysis and applied uniformly across conditions, rather than being introduced post hoc. In total, 37 dyads were initially recruited. Three dyads were excluded: one due to insufficient fNIRS data quality and two due to deception rates below 85%. The final analyzed sample therefore consisted of 34 dyads. We have now explicitly reported the exclusion process, the number of excluded dyads, and the corresponding reasons in the revised manuscript.

Please see the revised manuscript, page 9, lines 170 to 175, or the highlighted blue text:

Initially, 37 same-gender dyads composed of previously unacquainted individuals were formed from eligible participants. One dyad was excluded due to insufficient fNIRS data quality, and two dyads were excluded based on a predefined high-honesty criterion (deception rate<85%; Chen et al., 2020). The final

sample therefore consisted of 34 same-gender stranger dyads, including 18 female dyads (age: *M*=21.33, *SD*=2.07) and 16 male dyads (age: *M*=20.56, *SD*=1.29).

We sincerely appreciate your attention to the assumptions underlying the power analysis and the completeness of sample reporting. Your comments have helped us further improve the methodological clarity and rigor of the manuscript.

Reference

[1] Huang, R., Gao, X., Zhang, C., …, & Liu, Y. (2025). Forewarned Is Forearmed: The single- and dual-brain mechanisms in Detectors from Dyads of Varying Social Distance During Deceptive Outcomes Evaluation. The *Journal of neuroscience*, *45*(43): e2129242025.

[2] Chen, M., Zhang, T., Zhang, R., ..., & Li, X. (2020). Neural alignment during face-to-face spontaneous deception: does gender make a difference? *Human brain mapping*, *41*(17), 4964-4981.

---

(3) Subjective ratings: Were any ratings collected on impressions of the interaction partners (e.g. interpersonal closeness or sympathy) or on subjective states (e.g. arousal)? These ratings could corroborate the authors' interpretations of their findings, for example by showing that decisions about gain contexts are associated with higher levels of moral conflict.

Re: Thank you for this constructive suggestion. With regard to subjective ratings, we did collect measures related to interpersonal perceptions, although we did not systematically assess trial-level subjective states such as momentary arousal or perceived moral conflict.

Specifically, perceived interpersonal closeness (IOS) was measured both before and after the experiment to verify the success of the stranger-dyad manipulation and to examine whether the interaction altered relational perceptions. IOS scores remained consistently low and showed no significant changes across gain and loss contexts. This indicates that the observed differences in interpersonal neural synchrony were unlikely to be driven by changes in relational closeness or emotional bonding, and instead more plausibly reflect contextual modulation of strategic regulation.

In addition, prior to the experiment, detectors completed validated questionnaires assessing gullibility (George et al., 2020) and vigilance-related traits (Pettersson et al., 2014) to control for individual differences associated with susceptibility to deception. Preliminary analyses revealed no significant differences across dyads (including gender composition), reducing the likelihood that trait-level variability confounded the behavioral or neural findings.

We acknowledge that trial-level self-reports of moral conflict, tension, or arousal were not collected. This

decision was motivated by the continuous interactive design of the task (approximately 45 minutes). Inserting subjective ratings after each trial would have disrupted the natural flow of interaction and potentially altered the temporal dynamics of interpersonal neural synchrony (De Felice et al., 2024). Given that the primary aim of the study was to characterize real-time neural regulatory processes during spontaneous interaction, we prioritized preserving ecological validity over introducing intermittent self-report measures.

We appreciate the reviewer's suggestion, as it has allowed us to clarify the scope of our measurements and the theoretical boundaries of our inferences. **Relevant details regarding the subjective measures can be found in the revised manuscript (page 11, lines 209 to 220) or the highlighted blue text:**

Subjective Measures. **(1) IOS.** Perceived interpersonal closeness toward the interaction partner was assessed and administered both before and after the experiment. Given that all dyads consisted of previously unacquainted individuals, IOS scores were expected to be low prior to the experiment (i.e., below 2), an expectation that was confirmed by the present data. **(2) Measures for detectors.** Previous research has identified higher gullibility (George et al., 2020) and lower vigilance (Pettersson et al., 2014) as key personality traits associated with increased susceptibility to deception. Accordingly, detectors completed two questionnaires prior to the experiment. **(a)** Gullibility and susceptibility to deception were assessed using the **Social Vulnerability Scale** (SVS-15; Pinsker, 2011), a 0–4 Likert-type scale in which higher scores indicate a higher frequency of vulnerable behaviors. **(b)** Vigilance-related traits were assessed using the introspection and reactivity subscales of the **Hyperarousal Scale** (HAS; Ayabe et al., 2022). Items were rated on a 0–3 scale, with higher scores indicating a higher tendency toward heightened vigilance.

Reference

[1] George, M. S., Teunisse, A. K., & Case, T. I. (2020). Gotcha! Behavioural validation of the gullibility scale. *Personality and Individual Differences*, *162*, 110034.

[2] Pettersson, E., Mendle, J., Turkheimer, E., Horn, E. E., Ford, D. C., Simms, L. J., & Clark, L. A. (2014). Do maladaptive behaviors exist at one or both ends of personality traits?. *Psychological assessment*, *26*(2), 433.

[3] De Felice, S., Chand, T., Croy, I., …, & Vrtička, P. (2024). Relational neuroscience: Insights from hyperscanning research. Neuroscience & Biobehavioral Reviews, 169, 105979.

---

(4) Behavioural paradigm: Several points regarding the behavioural paradigm remained unclear to me after reading the manuscript. Did participants play both the role of sender and receiver? Furthermore, I was confused by the terms 'honesty rate' and 'deception rate'. How exactly were these variables computed? The terms sound as if one variable is the opposite of the other.

Re: Thank you for pointing out the lack of clarity in the description of the behavioral paradigm. In the revised manuscript, we have further clarified the role assignment and the calculation of behavioral metrics to avoid ambiguity.

Firstly, regarding the role assignment, this study did not use a role-switching design. Each dyad was randomly assigned to the roles of sender (cheater) and receiver (detector) at the start of the experiment, and these roles remained fixed throughout the entire experiment. Therefore, each participant only played one role, rather than switching between sender and receiver across different blocks. This design was implemented to maintain continuity in the interactive strategies and to avoid introducing additional learning or strategy reset effects that could arise from role-switching.

Please see the revised manuscript, page 10, lines 181 to 182, or the highlighted blue text:

Roles (sender/cheater vs. receiver/detector) were randomly assigned within each dyad and remained fixed throughout the experiment.

Secondly, regarding the definitions of honesty rate and deception rate, we have clarified that these variables are computed at the sender's decision level and represent two distinct behavioral proportions serving different analytical purposes. In each trial, the sender made a binary choice: (1) to recommend the option that was objectively beneficial to the receiver (truthful / detector-beneficial recommendation), or (2) to recommend the option that was objectively disadvantageous to the receiver but more beneficial to themselves (deceptive recommendation). Accordingly: Honesty rate refers to the proportion of trials in which the sender provided truthful, detector-beneficial recommendations. Deception rate refers to the proportion of trials in which the sender provided objectively detector-disadvantageous recommendations. Importantly, we further distinguish deception rate from successful deception rate. The latter is computed only within deceptive trials and reflects the proportion of those trials in which the receiver ultimately adopted the sender's recommendation, thereby resulting in a more favorable outcome for the sender.

Please see the revised manuscript, page 17, lines 331 to 337, or the highlighted blue text:

(1)Honesty rate. The proportion of trials in which the sender provided truthful and detector-beneficial information. To avoid bias in subsequent INS analyses caused by an insufficient number of deceptive trials, dyads with an honesty rate exceeding 85% were excluded (Chen et al., 2020). **(2) Deception rate.** The proportion of trials in which the sender provided information that was objectively disadvantageous to the receiver. **(3) Successful deception rate.** The proportion of deceptive trials in which the receiver ultimately obtained a lower gain or incurred a higher loss.

Thank you for pointing out the potential ambiguity at the terminology level, which will help improve the transparency and readability of the paradigm description.

(5) fNRIS analysis: While I am not an expert in fNRIS analysis, I wondered if information on the temporal dynamics of brain activation was available, and if the authors could illustrate this?

Re: We thank the reviewer for raising this important question regarding the temporal dynamics of brain activation. We agree that understanding the temporal evolution of neural processes in social decision-making and deception evaluation is crucial. Here, we address this issue from both methodological feasibility and theoretical rationale, explaining why we have chosen to apply trial-level interpersonal neural synchrony (INS) time-series analysis to capture these dynamic processes. From the physiological and technical characteristics of fNIRS signals, it is important to note that fNIRS is not well-suited for fine-grained analysis of "single-brain activation time dynamics." fNIRS measures the hemodynamic response to neural activity, which inherently has a significant time delay (typically 5-7 seconds) compared to neural electrical activity, and exhibits time-smoothing properties. This means that performing dynamic comparisons of brain activation at a sub-second or higher time resolution within individual trials is challenging because it is difficult to separate true neural events from delayed and overlapping blood flow responses. Therefore, in fNIRS research, single-brain activation analyses typically focus on trial-level averages or between-condition differences rather than fine-grained temporal processes, which is a common practice in fNIRS studies related to decision-making and social interactions (Zhang et al., 2025; Li et al., 2025; Xu et al., 2024; Filippetti et al., 2022).

To address the reviewer's concern about "temporal dynamics," we propose that the time evolution of INS provides a more theoretically meaningful and methodologically robust alternative within the fNIRS framework. Unlike single-brain activation, INS reflects the time-based synchronized changes in brain activity between interacting individuals. The calculation of INS relies on coherence or correlation structures between signals, which is less dependent on absolute blood flow amplitudes and more focused on the consistency of cross-brain signals over time. Therefore, INS is particularly suited to capture the dynamic characteristics of information exchange, alignment of understanding, and strategic adjustment in social interactions. Based on this rationale, we have included a trial-level time-series analysis of ΔINS in the revised manuscript.

Please see the revised manuscript, page 21, lines 415 to 421, or the highlighted blue text:

Trial-level ΔINS Time-series Analysis. To further characterize the temporal dynamics underlying evaluative and behavioral modulation during deceptive information processing, a time-series analysis of trial-level ΔINS was conducted for successful and unsuccessful deception trials. Following the approach described by Jiang et al. (2015), trial-level ΔINS coherence was computed at 15 consecutive time points under each condition. Paired-samples t-tests were then performed to compare ΔINS values between successful and failed deception trials at each time point. To control for multiple comparisons across time points, FDR correction was applied.

The results indicate that although fNIRS is not well-suited to provide high–temporal resolution dynamics of single-brain activation, the time-series evolution of INS clearly captures the dynamic divergence between different types of deception trials during evaluative processing. Specifically, in the DLPFC, the differentiation in INS between successful and failed deception emerged as early as approximately 7 seconds after the onset of verbal interaction in the gain context. In contrast, in the loss context, this divergence appeared later—around the 10th second of the verbal statement—and persisted until the end of the trial. These findings suggest that interpersonal neural synchrony not only reflects overall differences in interaction states but also reveals critical temporal inflection points in the unfolding of strategic social exchanges.

Please see the revised manuscript, page 35, lines 648 to page 36, line 661, or the highlighted blue text:

Trial-by-Trial ΔINS Time-Series Dynamics

To further characterize the dynamic neural mechanisms underlying evaluative processes during deception, we conducted time-series analyses of ΔINS for successful and failed deception trials (**Figure 8**). Results revealed that: **(1)** In the DLPFC, significant difference in INS between successful and failed deception trials emerged approximately 7 seconds after the onset of the verbal interaction in the gain context. This suggests that INS begins to differentiate deception outcomes relatively early during interpersonal interaction. **(2)** In the loss context, significant ΔINS differences emerged later, around the 10th second of the verbal statement, and persisted through the end of the trial.

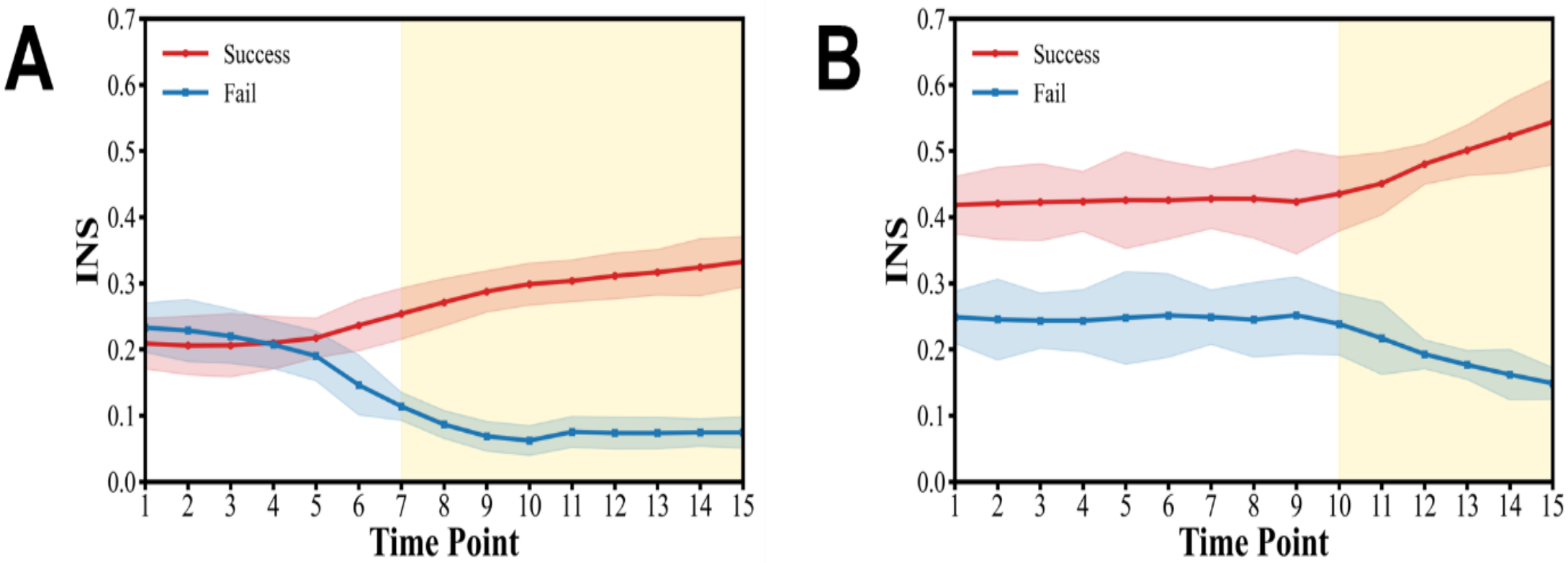

Figure 3. Trial-by-trial ΔINS time-series dynamics during successful and failed deception across contextual. (A) Gain context; **(B)** Loss context. The red line represents ΔINS during successful deception trials, and the blue line represents ΔINS during failed deception trials. Shaded regions indicate 95% confidence intervals. Yellow-highlighted indicating significant ΔINS differences.

In summary, we did not overlook temporal dynamics. Rather, after carefully considering the physiological characteristics of fNIRS signals, we adopted a methodologically feasible and theoretically appropriate INS time-series approach to characterize dynamic neural processes during social interaction. We have now clarified this analytical rationale in the revised *Methods* and *Results* sections to enhance transparency and interpretability. We

sincerely thank the reviewer for prompting us to articulate the methodological boundaries and justification more clearly.

Reference

[1] Li, C., Guo, X., Wang, J., & Wang, S. (2025). Single video games improve cognitive functioning in college students: evidence from behavioral and fNIRS assessments. *Frontiers in Psychiatry*, *16*, e1640142.

[2] Xu, G., Zhou, M., Chen, Y., Song, Q., Sun, W., & Wang, J. (2024). Brain activation during standing balance control in dual-task paradigm and its correlation among older adults with mild cognitive impairment: a fNIRS study. *BMC Geriatrics*, *24*(1), 04772-4775.

[3] Filippetti, M., Andreu-Perez, J., De Klerk, C., Richmond, C., & Rigato, S. (2022). Are advanced methods necessary to improve infant fNIRS data analysis? An assessment of baseline-corrected averaging, general linear model (GLM) and multivariate pattern analysis (MVPA) based approaches. *NeuroImage*, *265*, 119756.

[4] Jiang J, Chen C, Dai B, Shi G, Ding G, Liu L, Lu C (2015) Leader emergence through interpersonal neural synchronization. *Proceedings of the National Academy of Sciences of the United States of America*, *112*, 4274-4279.

---

(6) Relationship between deception and INS: The authors report a significant negative correlation in the gain context, but not in the loss context. They could report the correlation in the loss context and then statistically compare these correlations to support the idea that the relationship is specific to the gain context.

Re: We sincerely thank the reviewer for raising this important statistical concern. As you correctly noted, demonstrating significance in one context but not in another is insufficient to substantiate claims of contextual specificity. In the revised manuscript, we have now reported the correlation coefficients between ΔINS and successful deception rate separately for both gain and loss contexts. This additional analysis allows us to present a more rigorous and nuanced conclusion: the brain–behavior coupling between ΔINS and successful deception is present in both contexts, but it is stronger and more stable in the gain condition. Importantly, no significant contextual differences were observed for failed deception trials. This clarification strengthens the statistical basis of our interpretation and avoids over-reliance on differential significance testing as evidence for context specificity.

Please see the revised manuscript, page 36, line 663 to page 37, line 678, or the highlighted blue text:

To examine whether ΔINS was associated with deception behavioral performance, Pearson correlation analyses were conducted separately for gain and loss contexts. Results revealed that ΔINS during successful deception was significantly associated with successful deception rate in both contexts. In the gain context, a negative correlation was observed (r=-0.934, p<0.001; **Figure 9A**). In the loss context, ΔINS was also

significantly correlated with successful deception rate (*r*=−0.442, *p*=0.009; **Figure 9B**). In contrast, ΔINS during failed deception trials was not significantly associated with failed deception rate in either context (gain: *r*=0.264, *p*=0.131, **Figure 9C**; loss: *r*=0.074, *p*=0.676, **Figure 9D**).

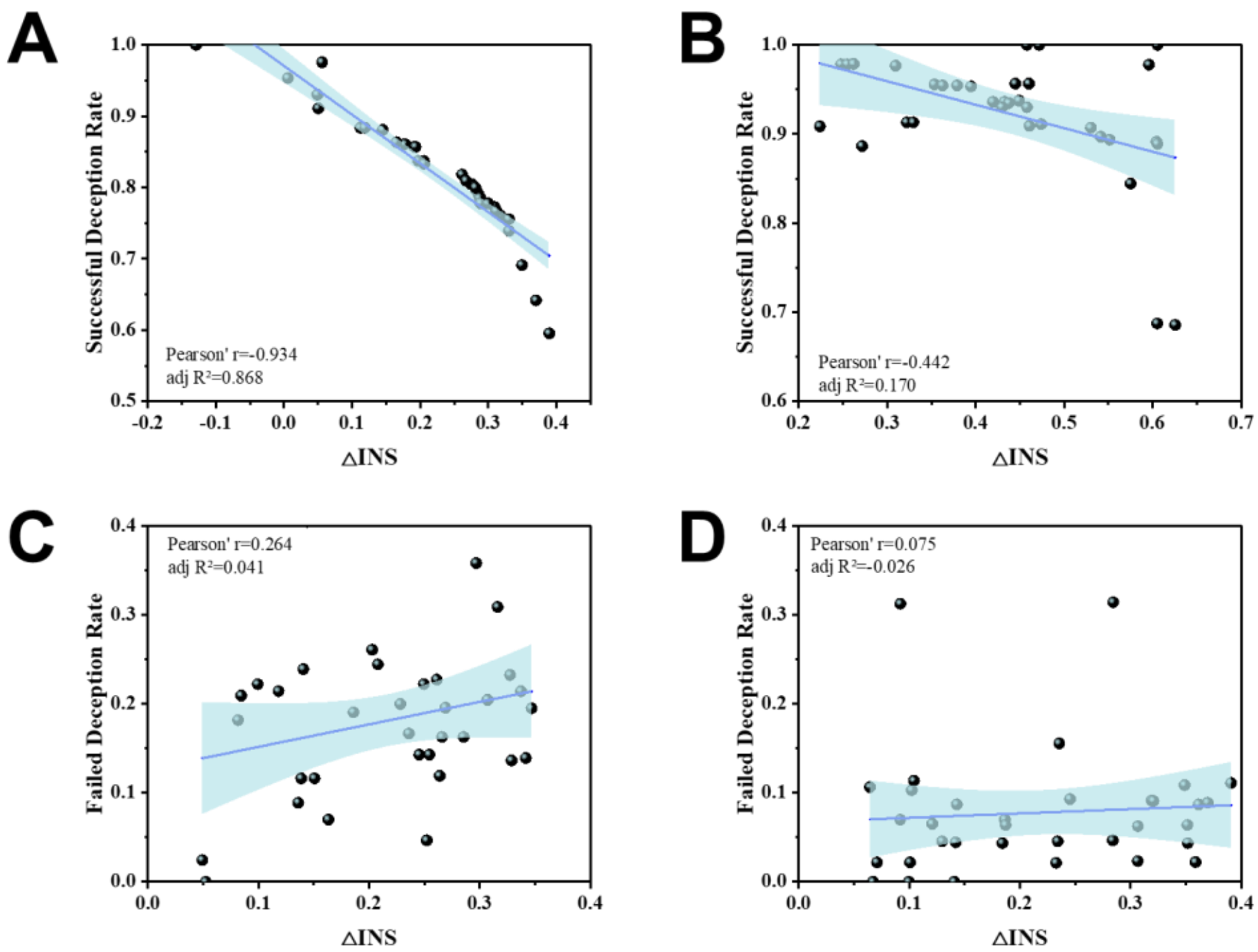

Figure 9. Brain–behavior correlations between ΔINS and deception rates across contexts. (A) Correlation between ΔINS during successful deception and successful deception rate in the gain context. **(B)** Correlation between ΔINS during successful deception and successful deception rate in the loss context. **(C)** Correlation between ΔINS during failed deception and failed deception rate in the gain context. **(D)** Correlation between ΔINS during failed deception and failed deception rate in the loss context. Lines represent linear regression fits; shaded areas denote 95% confidence intervals. Reported statistics include Pearson's *r* and adjusted R^2.

---

(7) Machine learning algorithm: Again, I am not an expert in applying machine learning algorithms. However, I wondered what constitutes an increase in knowledge when applying this algorithm? Is it merely to demonstrate a relationship between behaviour and brain activity? This has already been demonstrated by the correlation, hasn't it? Perhaps the authors could explain this in more detail. Moreover, given that the sample size is quite small, I am concerned about the validity of these findings, as it is not possible to separate the test and validation samples.

Re: We sincerely thank the reviewer for this constructive suggestion. We agree that if machine learning analysis is only aimed at "demonstrating the relationship between behavior and brain activity," its methodological value would indeed be limited. Therefore, it is necessary to clearly articulate the specific purpose of incorporating machine learning analysis into this study and how it provides added knowledge beyond traditional correlational analyses.

First, the core goal of machine learning analysis in this study is not to re-examine the correlation between variables, but to assess the predictive power of brain-behavior indicators at the individual level. While correlational analysis addresses "whether variables co-vary," machine learning models ask: when given new, previously unmodeled individuals or trials, can the INS measure predict behavior outcomes (such as successful or failed deception)? This "predictability" dimension provides information that correlational analysis alone cannot offer.

Second, in practical terms, machine learning analysis allows us to simultaneously integrate multidimensional features (e.g., INS measures from different time scales or channels) and evaluate whether these features constitute a discriminative pattern for behavior outcomes through model fitting and performance assessment. Therefore, the knowledge gained from machine learning does not rest on the importance of a single variable but instead reveals whether cross-brain neural synchrony can serve as a functional indicator for systematically predicting complex social decision-making outcomes. This is especially critical for evaluating the potential application value of INS, such as in behavior prediction or risk identification.

Regarding the reviewer's concern about the small sample size and the lack of a test-validation separation, we fully understand the rationale behind this concern. To address it, we did not rely on a simple training/test split but instead used LOOCV as our model evaluation strategy. Specifically, in each iteration, we used one participant (or dyad) as the validation sample, and the remaining samples for training the model. This process was repeated until every sample was independently validated once. This approach has two important advantages in small-sample contexts: (1) it maximizes the use of available training data, avoiding information loss due to sample partitioning, and (2) it ensures that each sample's prediction comes from a model that did not use it in training, thus providing a more conservative and robust estimate of generalization performance.

We have significantly revised the introduction to reflect this modeling strategy and have explicitly distinguished our approach from traditional regression analyses aimed at describing brain-behavior relationships. The focus of the study now emphasizes the use of neural signals to predict deceptive behavior and validate model robustness. These revisions can be found in **the revised introduction (page 7, line 132 to page 8, line 150, marked in blue)**.

However, while conventional correlational (Fu et al., 2025) or regression-based analyses (Helmer et al., 2024) are effective for identifying brain-behavior associations within specific contexts, they are limited in evaluating whether such relationships generalize across contexts or remain robust under different modeling assumptions. Addressing these questions requires analytical approaches that move beyond descriptive fitting toward prediction-oriented validation (Zhuang et al., 2020). Machine-learning (ML) methods provide a suitable framework for this purpose by treating neural measures as feature variables and behavioral outcomes as prediction targets, thereby enabling direct tests of whether brain-based signals carry reliable predictive information (Shamout et al., 2020; Wang et al., 2020). Importantly, robustness of prediction should not depend on a single algorithm but should be evaluated

across multiple models with different inductive biases (Huang et al., 2025). Accordingly, the present study adopts a cross-validated modeling framework in which several commonly used algorithms—including Logistic Regression, least-squares regression (LSR), Decision Trees (DT), Naive Bayes, and Support Vector Regression (SVR)— are systematically compared. Within this framework, ΔINS are used to predict individual differences in cheaters' successful deception rates under gain-loss contexts. Model performance is evaluated using leave-one-out cross-validation, allowing each participant's deception rate to be predicted from models trained on independent data. By emphasizing cross-validated prediction and model comparison, this approach enables us to assess the generalizability and robustness of the neural–behavioral relationship, rather than relying on parameter estimates from a single regression model.

Regarding the concern about the relatively small sample size (N=34), we acknowledge that this limits the use of traditional train-test split approaches. To mitigate this issue, we have employed LOOCV, a method that is commonly recommended in small-sample neuroimaging and computational modeling studies (Zhu et al., 2023; Poldrack et al., 2019) to provide an unbiased estimate of generalization performance. We have provided a detailed explanation of this procedure in the "Methods" section, including how model performance is assessed and validated.

Additionally, in response to the reviewer's suggestion, we have expanded our analysis by incorporating comparisons across various machine learning algorithms, including both linear and nonlinear models, to demonstrate that the observed predictive patterns are not reliant on a single modeling choice. These additional analyses, combined with performance metrics from cross-validation, are now reported to provide greater transparency and robustness to our findings.

You can find the detailed machine learning modeling methods in the revised manuscript, pages 22, line 438 to page 24, line 473, marked in blue.

Predicting Successful Deception Using ΔINS: a ML Approach

Given the relatively small sample size of the present study (N=34), conventional train–test splits may yield performance estimates that are highly sensitive to sample partitioning, thereby compromising the stability of generalization assessment. To address this issue, we adopted a leave-one-out cross-validation (LOOCV) strategy. LOOCV has been widely recommended in small-sample neuroimaging studies, as it maximizes the use of available training data while providing an approximately unbiased estimate of model generalization performance (Zhu et al., 2023; Poldrack et al., 2019). In each cross-validation iteration, data from one dyad were held out as the test set, whereas data from the remaining N-1 dyads were used for model training. This procedure was repeated N times, ensuring that each dyad served exactly once as an independent test sample.

Model Construction. ΔINS values were extracted from fNIRS channel pairs that exhibited significant

contextual differences in the preceding statistical analyses and were used as input features for model construction. The successful deception rate served as the prediction target, capturing individual differences in deceptive performance across gain and loss contexts.

Model Evaluation. To avoid dependence on a single modeling assumption, multiple commonly used ML algorithms were systematically compared within a unified LOOCV framework. These algorithms included Logistic Regression, LSR, DT, Naive Bayes, and SVR. Linear models (e.g., Logistic Regression and LSR) were primarily used to assess linear predictive relationships between ΔINS and successful deception rates, whereas nonlinear models (e.g., DT and SVR) were employed to capture potential nonlinear patterns. We further evaluated classification-related performance metrics derived from model predictions, including Accuracy, Precision, Recall, and the F1 score. Accuracy reflects the overall proportion of correct predictions; Precision indexes the reliability of predicted deceptive outcomes; Recall quantifies sensitivity to true deceptive behavior; and the F1 score represents the harmonic mean of Precision and Recall, providing a balanced measure that is particularly suitable for imbalanced class distributions or asymmetric misclassification costs (Altalhan et al., 2025; Chen & Cheng, 2023).

Model Robustness Assessment. Model robustness was further quantified by computing the Pearson's r between LOOCV-based predicted values and observed successful deception rates. This metric captures the model's ability to account for individual differences in deceptive behavior at the continuous level. To assess the statistical significance of the observed prediction performance, permutation testing was conducted to construct a null distribution. In each permutation, deception-rate labels were randomly shuffled while feature values were held constant, and the entire prediction procedure was repeated. This process was iterated 1,000 times to generate a distribution of correlation coefficients expected under chance-level prediction. The empirical r obtained from the true model was then compared against this null distribution to determine whether predictive performance exceeded random levels (Huang et al., 2025).

In addition, detailed evaluation results of the ML models are provided in the *Results* section of the revised manuscript (pages 40, line 700 to page 42, line 723, highlighted in blue).

Predicting Successful Deception from ΔINS: Comparative ML Analyses

To further characterize the brain–behavior relationship, we implemented exploratory ML approaches. Based on the preceding findings, ΔINS during failed deception and single-brain activation measures neither correlated significantly with deception outcomes nor contributed incremental explanatory power in hierarchical regression models. We therefore hypothesized that ΔINS during successful deception constitutes the critical neural predictor of deceptive performance.

Table 11. Performance evaluation of different ML models.

Model	Condition	ROC-AUC	Accuracy	Precision	Recall	F1 Score	MCC
LSR	Gain	0.929	0.727	0.750	0.857	0.800	0.386
	Loss	0.867	0.727	0.800	0.667	0.727	0.467
Logistic Regression	Gain	0.893	0.909	0.875	0.796	0.733	0.810
	Loss	0.800	0.636	0.667	0.667	0.667	0.817
Naive Bayes	Gain	0.768	0.727	0.650	0.757	0.700	0.386
	Loss	0.733	0.727	0.800	0.667	0.727	0.467
DT	Gain	0.732	0.677	0.833	0.714	0.669	0.449
	Loss	0.817	0.818	0.833	0.833	0.833	0.633
SVR	Gain	0.821	0.818	0.857	0.857	0.610	0.607
	Loss	0.667	0.545	0.600	0.500	0.545	0.100

Separate predictive models were constructed for gain and loss contexts, with ΔINS during successful deception entered as input features and successful deception rate as the target variable. To identify the optimal predictive framework, we systematically compared multiple ML algorithms encompassing both linear and non-linear approaches. Across contexts, LSR demonstrated superior predictive performance[gain context: ROC-AUC=0.929, Accuracy=0.727, **Figure 10A**; loss context: ROC-AUC=0.973, Accuracy=0.933, **Figure 10B**]. Detailed performance metrics for all models are reported in **Table 11**. Using the LSR model, ΔINS within the significant channel pair reliably predicted successful deception across contexts (gain: R^2=0.870, MSE=0.001; loss: R^2=0.266, MSE=0.004). To assess statistical significance, we conducted 5,000 label-shuffled permutation tests. In both contexts, the observed predictive performance exceeded chance levels (gain: r=0.933, p<0.001, **Figure 10C**; loss: r=0.516, p<0.001, **Figure 10D**).

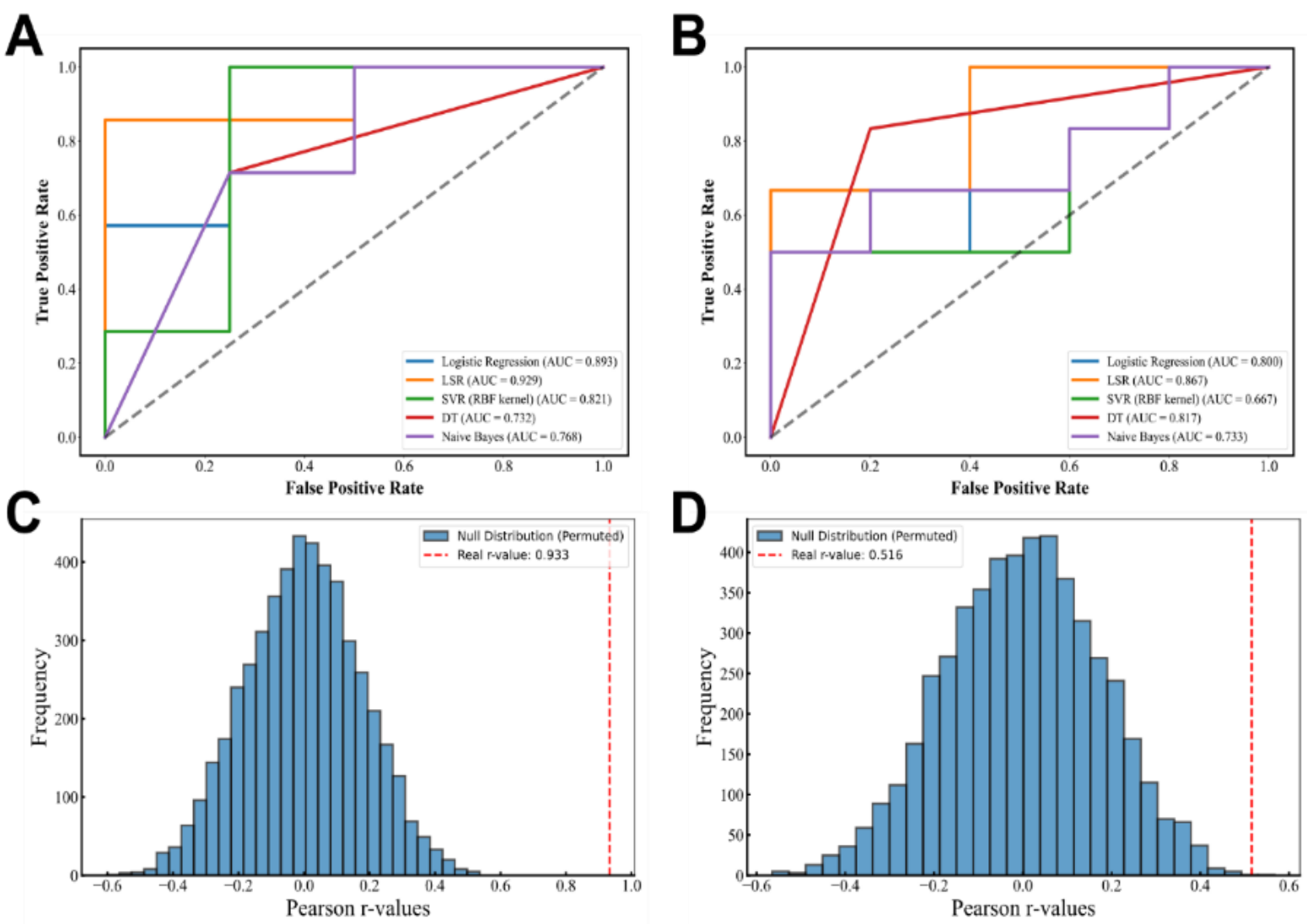

Figure 10. Comparative predictive performance of ML models across contexts. (A) ROC curves comparing ML models in the gain context. **(B)** ROC curves comparing ML models in the loss context. **(C)** Results of the 5,000-permutation test for predicting successful deception using ΔINS features in the gain context. **(D)** Results of the 5,000-permutation test for predicting successful deception using ΔINS features in the loss context.

Reference

[1] Fu, H., Tang, S., & Zhao, X. (2025). Limitations of Correlation Coefficients in Research on Functional Connectomes and Psychological Processes. Human Brain Mapping, 46(10), hbm70287.

[2] Helmer, M., Warrington, S., Mohammadi-Nejad, A., …, & Murray, J. (2024). On the stability of canonical correlation analysis and partial least squares with application to brain-behavior associations. Communications Biology, 7(1), 5869-5873.

[3] Shamout, F., Zhu, T., & Clifton, D. (2020). Machine Learning for Clinical Outcome Prediction. IEEE Reviews in Biomedical Engineering, 14, 116-126.

[4] Wang, S., McCormick, T., & Leek, J. (2020). Methods for correcting inference based on outcomes predicted by machine learning. Proceedings of the National Academy of Sciences of the United States of America, 117(48), 30266-30275.

[5] Huang, R., Gao, X., Zhang, C., …, & Liu, Y. (2025). Forewarned Is Forearmed: The single- and dual-brain mechanisms in Detectors from Dyads of Varying Social Distance During Deceptive Outcomes Evaluation. The Journal of neuroscience, 45(43): e2129242025.

[6] Zhu, X., Kim, Y., Ravid, O., …, & Morey, R. A.(2023). Neuroimaging-Based Classification of PTSD Using Data-Driven Computational Approaches: A Multisite Big Data Study from the ENIGMA-PGC PTSD Consortium. NeuroImage, 283, 120412.

[7] Poldrack, R., Huckins, G., & Varoquaux, G. (2019). Establishment of Best Practices for Evidence for Prediction: A Review. JAMA psychiatry, 77(5), 534.

[8] Altalhan, M., Algarni, A., & Alouane, T. (2025). Imbalanced Data Problem in Machine Learning: A Review. IEEE Access, 13, 13686-13699.

[9] Chen, H., & Cheng, Y. (2023). Travel Mode Choice Prediction Using Imbalanced Machine Learning. IEEE Transactions on Intelligent Transportation Systems, 24(4), 3795-3808.

---

(8) Response times: As the authors assume that deception in loss contexts might be associated with more automated cognitive processing, I wondered if they had analysed response times. This would support their assumption if they showed that response times were shorter for deception decisions in loss contexts.

Re: Thank you for raising this important behavioral question. Regarding the assumption that deception in loss contexts may be more automatic, we have systematically analyzed response times (RTs) in the revised manuscript and incorporated them as key behavioral evidence. Specifically, for the sender's initial decision phase (i.e., whether

to choose deception or honesty), we conducted a 2 (Context: gain vs. loss)×2 (Behavior type: honesty vs. deception) repeated-measures ANOVA. The results revealed a significant main effect of context, with overall shorter RTs in loss compared to gain contexts, indicating that contextual framing influenced decision speed. Crucially, a significant Context×Behavior Type interaction emerged: in loss contexts, deceptive decisions were executed significantly faster than honest decisions, whereas in gain contexts no significant RT difference was observed between deception and honesty. This pattern is consistent with our theoretical account, suggesting that under loss framing, deception may be implemented as a faster, lower-cost strategy, reflecting processing that is closer to automatized responding. In contrast, deception in gain contexts did not exhibit this temporal advantage.

In addition, we analyzed detectors' RTs during the final decision phase. Results showed that decisions were generally faster in loss contexts, and RTs were significantly shorter when detectors adopted the sender's recommendation under loss framing. This further supports the interpretation that loss contexts may facilitate more rapid value integration and behavioral execution. Thus, RT analyses were not only included but also provide convergent behavioral evidence supporting our interpretation that deception under loss contexts is associated with more automatic processing dynamics.

Please see the revised manuscript, page 27, line 533 to page 29, line 562, or the highlighted blue text:

To examine the RT underlying cheaters' first decisions (i.e., the first behavioral expression of deception versus honesty), a 2 (Context: gain vs. loss)×2 (Behavior type: honesty vs. deception) repeated-measures ANOVA was conducted (**Figure 3A**). A significant main effect of contextual framing was observed[loss vs. gain: 3144.11±139.16 vs. 3541.29±174.11, $F_{(1,33)}$=35.132, p<0.001, η_p^2=0.516], indicating that RT were significantly shorter under loss compared to gain contexts. A significant interaction between context and behavior type also emerged [$F_{(1,33)}$=5.0422, p=0.032, η_p^2=0.133]. Simple-effects analyses revealed that under loss contexts, deception were executed significantly faster than honesty (deception vs. honesty: 3047.40±127.74 vs. 3240.82±159.51; post hoc p= 0.018, *95% CI* [−351.727, −35.111]). Contrastly, under gain contexts, no significant difference was observed between deceptive and honest responses (deception vs. honesty: 3589.68 ± 187.97vs. 3492.91±165.10; post hoc p= 0.132, *95% CI* [−30.630, 224.161]). No significant main effect of behavior type was detected [deception vs. honesty: 3318.54±151.49 vs. 3366.87±157.77, $F_{(1,33)}$=2.876, p=0.099, η_p^2=0.080].

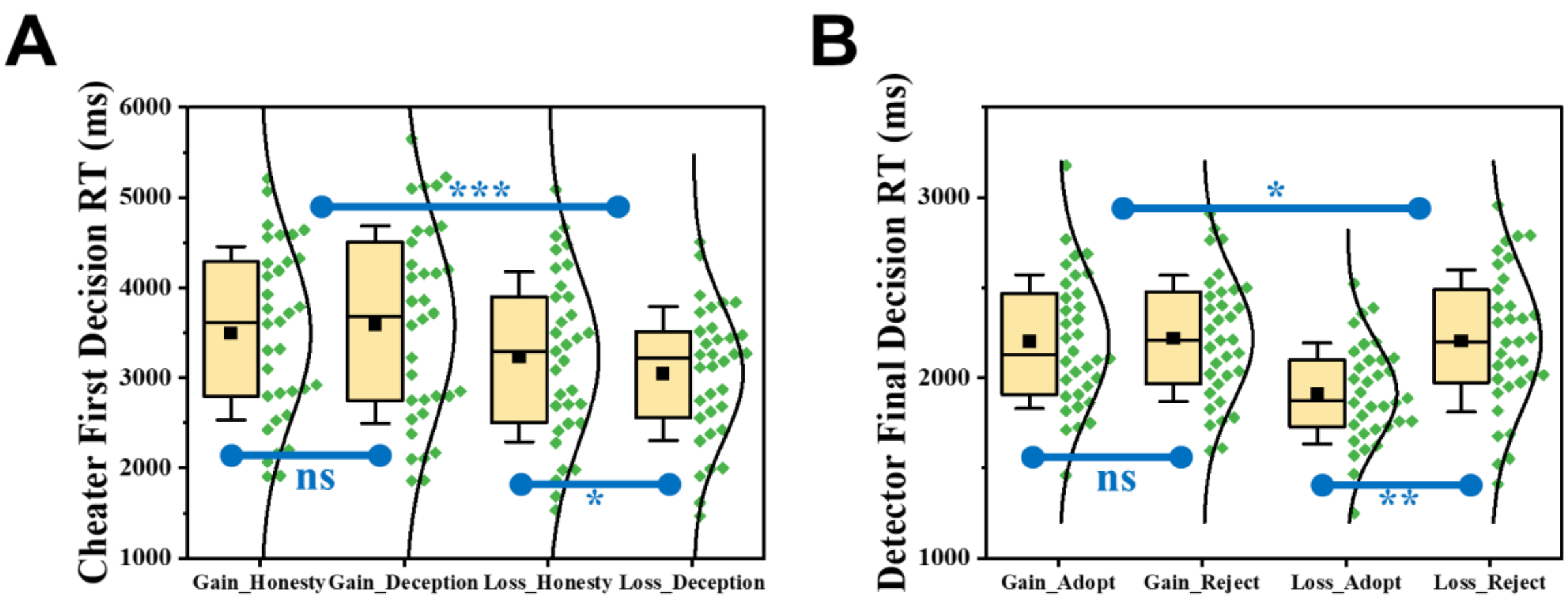

Figure 3. RT results. (A) Cheater RT at the first decision phase. **(B)** Detector RT at the final decision phase . **$p<$ 0.01, *$p<$ 0.05, ns= non-significant. The same conventions apply in subsequent figures.

We further examined detectors' RT during the final decision phase to characterize their decision-making dynamics under different contextual and recommendation conditions. A 2 (context: gain vs. loss)×2 (recommendation adoption: adopted vs. rejected) repeated-measures ANOVA revealed a significant main effect of context, with overall shorter RT in loss compared to gain contexts [loss vs. gain: 2058.96±38.67 vs. 2209.68±46.18, $F_{(1,33)}$=5.125, p=0.030, η_p^2=0.134]. A significant main effect of recommendation adoption was also observed, indicating that RT were shorter when detectors adopted the cheater's recommendation (2056.82±39.34) than when they rejected (2211.82±43.87) it [$F_{(1,33)}$=5.829, p=0.021, η_p^2=0.150]. Importantly, the interaction between context and recommendation adoption was significant [$F_{(1,33)}$=6.024, p=0.020, η_p^2 =0.154]. Simple-effects analyses showed that in loss contexts, RT were significantly shorter when recommendations were adopted compared to rejected (adopted vs. rejected: 1912.96±47.80 vs. 2204.96±67.52, post hoc p=0.002, *95%CI* [113.406, -470.599]). No significant difference between adoption (2200.68 ± 63.69) and rejection (2218.68± 59.93) was observed in gain contexts (post hoc p= 0.828, *95% CI* [−149.387, 185.398]). These results are illustrated in **Figure 3B**.

---

(9) Discussion: Overall, I found that the structure of the discussion could be improved by shortening or combining some paragraphs. Adding some transitions and summary sentences would help to clarify to the reader what each paragraph is about.

Re: Thank you very much for this thoughtful and constructive suggestion. We agree that, in the original version, the Discussion section—while comprehensive—contained several relatively long paragraphs and dense theoretical

elaborations that may have obscured the hierarchical logic and made it more difficult for readers to clearly follow the progression of arguments. In response to your comment, we have undertaken a structural reorganization of the Discussion with three primary goals: (1) reducing redundancy, (2) improving logical transitions, and (3) clarifying the functional role of each paragraph.

First, we shortened and consolidated several paragraphs that previously addressed closely related themes (e.g., behavioral findings and Prospect Theory implications; single-brain DLPFC activation and control-demand interpretation). These sections are now integrated into more concise thematic units, each centered around a clearly articulated claim. Redundant theoretical restatements were removed, and overlapping citations were streamlined to enhance readability without weakening theoretical grounding.

Second, we introduced explicit transition and signposting sentences at the beginning and end of major sections. For example, we now structure the Discussion into clearly delineated levels: (1) Behavioral contextual modulation; (2) Single-brain control mechanisms; (3) Inter-brain synchronization as an interactive mechanism; (4) Brain–behavior coupling and predictive modeling; (5) Limitations and boundary conditions. Each section now begins with a brief orienting statement and ends with a short integrative sentence summarizing the conceptual takeaway before transitioning to the next level of analysis. This restructuring makes the theoretical progression—from individual execution to interactive alignment to predictive modeling—more explicit and easier to follow.

Third, we added concise summary sentences in key locations to reinforce the central argument of the paper—namely, that gain-loss contexts "reparametrize" deception across motivational, control-related, and interactive neural levels. These integrative sentences help prevent the Discussion from appearing as a sequence of loosely connected interpretations and instead present it as a unified mechanistic narrative. Importantly, while we reduced length and tightened structure, we ensured that the core theoretical contributions—contextual modulation, DLPFC control asymmetry, success-specific ΔINS, temporal dynamics, and predictive robustness—remain fully represented.

We sincerely appreciate your comment, as it prompted us to substantially improve the clarity, coherence, and narrative flow of the Discussion. We believe the revised version now presents the findings in a more logically organized and reader-friendly manner, while preserving the depth of theoretical interpretation.

Please see the revised manuscript, page 42, line 725 to page 49, line 883, or the highlighted blue text:

The present study demonstrates that deception is not a fixed individual trait but rather a energetic, interactive decision-making process that is "reparametrized" by gain-loss contexts. At the behavioral level, loss contexts significantly increased the frequency and success rate of deception, accompanied by shorter response times. Accordingly, at the single-brain level, only the DLPFC exhibited higher activation levels during deception trials in the gain contexts, suggesting that deception in these contexts relies more heavily on high-

cognitive-load strategic monitoring. The more crucial neural mechanisms emerge from our investigations at the dual-brain level, the inter-brain analysis revealed a key neural mechanism: the change in ΔINS between dyads' DLPFCs showed significant contextual differences only during "successful deception." No such effect was observed during honest or failed deception conditions. This indicates that gain-loss contexts do not universally enhance interaction intensity but selectively amplify △INS during successful deception interactions. This conclusion is further supported by trial-level analyses, where ΔINS significantly distinguished successful from failed deception in both contexts, albeit with distinct temporal dynamics (in gain contexts, differentiation occurs earlier; in loss contexts, it occurs later but is more persistent). Furthermore, hierarchical regression and cross-model validation confirmed that ΔINS provides unique incremental predictive power beyond single-brain activation and eye contact, offering a new dimension for understanding the interactive neural mechanisms of deception.

The behavioral results clearly indicate that gain-loss contexts systematically modulate the probability and execution of deception in interpersonal interactions. Compared to gaining benefits, individuals were more inclined to employ deception to maintain their interests when facing potential losses. This aligns with prior research suggesting that individuals are more likely to adopt strategic or norm-violating behaviors to avert negative outcomes rather than to obtain equivalent gains (Grolleau et al., 2016). This implies that deception is not merely an incidental behavioral deviation but a functional strategy activated by contextual pressure. Our findings also directly support the core tenets of Prospect Theory, which posits that gain-loss contexts significantly influence deceptive behavior (Kahneman et al., 1990). Furthermore, decision-making was significantly faster in loss contexts—deceivers executed deception faster than honesty, and receivers accepted deceptive offers faster than they rejected them. This is consistent with evidence that loss contexts trigger rapid, threat-sensitive processing pathways (Kern & Chugh, 2009), suggesting that approach-avoidance motivation compresses deliberation time, facilitating deception automaticity. In contrast, gain contexts showed no significant differences in response times between deception and honesty, nor between acceptance and rejection. This pattern accords with research indicating that gain frames induce more detailed value comparison and delayed decision-making (Levin et al., 1998), suggesting possibly exist a more balanced information integration in gain contexts.

At the single-brain level, contextual differences were concentrated in the DLPFC and were specific to deception trials, with no such regulation observed in honest trials. While loss contexts behaviorally elicited more frequent and successful deception with faster decision rhythms, neurally, it was the gain context that necessitated greater DLPFC recruitment, supporting high-cost strategic monitoring and executive control. This interpretation aligns with a large body of research that the DLPFC is not continuously participate in all value

assessments, but is recruited more strongly when maintaining goals, suppressing dominant responses, and performing strategic comparisons and monitoring. (Hutcherson & Tusche, 2022; Zoh et al., 2021; Turnbull et al., 2019). In social decision-making, the role of the prefrontal cortex is often interpreted as imposing "top-down constraints" on contextual and rule-based processing, particularly in regulating value signals during multi-cue tradeoffs and norm-benefit conflicts (Phillips et al., 2021; Fascianelli et al., 2020). Moreover, under loss frames, approach-avoidance motivation and threat sensitivity drive decisions toward rapid, relatively automated tracks (Kern & Chugh, 2009; Mäki-Marttunen et al., 2019), manifesting behaviorally as faster deception. Conversely, while deception in gain contexts is less frequent, it appears more cognitively demanding—requiring detailed comparison of potential gains, maintenance of deceptive goals, monitoring of the partner's reactions, and suppression of intuitive response—thereby intensifying DLPFC engagement (Corbi & Soutschek, 2024; Kim et al., 2023; Zoh et al., 2021). In other words, neural evidence suggests that gain-loss contexts alter not only the motivation for deception but also its execution pathway: loss contexts trigger a "low-threshold, high-probability" strategy, whereas gain contexts necessitate a "high-threshold, strong-control" execution. Importantly, if contextual differences stemmed merely from general arousal, speech intensity, or interaction engagement, PFC differences should also appear in honest conditions; however, the restriction of DLPFC differences to deception conditions supports a mechanism of "control processing required for deception" rather than general verbal activity. This conclusion should be interpreted cautiously within the boundary conditions of fNIRS naturalistic measurement: open verbal interaction may introduce physiological components such as head motion, facial expressions, and articulation. Since fNIRS is sensitive to superficial cortical blood flow, fully isolating these sources is challenging. Therefore, we relied on multi-step preprocessing and trial-level quality screening to minimize non-neural noise, emphasizing the "pattern evidence" of the results—spatially focused on the DLPFC, specific to deception conditions, and theoretically consistent with the higher control demands of gain frames.

While single-brain results reveal the "execution pathways" of deception across contexts, the inter-brain INS results uncover the "interactive mechanisms" of deception success. In the task, the ΔINS between dyads' DLPFCs was significant only during successful deception conditions. This condition-specificity carries important theoretical implications. First, it suggests that contextual frames do not simply increase interaction intensity or communication effort; rather, they selectively amplify the cross-brain alignment of control networks at the critical moment of "successfully altering another's decision." This parallels research on the role of interpersonal neural synchronization in strategic coordination, competitive gaming, and negotiation success (Cui et al., 2012; Wang et al., 2020; Huang et al., 2025). Prior studies indicate that DLPFC-related INS often reflects shared regulation of rule understanding, strategy adjustment, and goal maintenance, rather than

emotional resonance (Jiang et al., 2015; Baker et al., 2021). Therefore, the stronger DLPFC–DLPFC synchronization observed in loss contexts in this study may imply that in high-threat, fast-paced context, successful deception relies heavily on a “dynamic locking” of control-related processing—where the deceiver maintains manipulation targets while the detector integrates information within a limited time window, and the synchronized regulation of rhythm enhances the successful deception rate.

Furthermore, ΔINS not only distinguished successful from failed deception statistically but also demonstrated significant predictive power in both contexts, with higher classification accuracy in loss contexts. This suggests that INS is not a post-hoc averaged group effect but carries real-time information about decision outcomes within single interactions (Reinero et al., 2021; Jiang et al., 2025). More importantly, time-series analysis revealed distinct temporal dynamics: in gain contexts, INS differences between success and failure emerged early (approximately 7 seconds), suggesting that in context requiring higher strategic monitoring, interacting partners enter a state of control alignment earlier (Long & Chen, 2022). In contrast, in loss contexts, differences appeared later (approximately 10 seconds) but persisted until the end of the trial, indicating that synchronization may not be a prerequisite for the initiation phase but rather strengthens progressively to consolidate decisions and maintain influence (Gordon et al., 2024; Pan et al., 2017). Taken together, the task-level and trial-level INS results jointly indicate that successful deception relies on the cross-brain synergy of control networks between interacting partners rather than merely individual control resource investment (Shao et al., 2023). Gain and loss contexts shape the timing and intensity of this synergy by modulating decision rhythms and motivations, reflecting differentiated interactive regulation mechanisms at the neural level.

Building on this, brain-behavior correlations indicate that the ΔINS of the DLPFC–DLPFC is not an “epiphenomenon” but a functional indicator closely related to individual deception success rates (Reinero et al., 2021; Jiang et al., 2025). In both gain and loss contexts, ΔINS during successful deception was significantly negatively correlated with the success rate of deception, whereas ΔINS during failed deception showed no such relationship. This pattern, sensitive only to successful deception, aligns with the task-level results—Cross-brain synchronization reflects not the occurrence of interaction, but whether the interaction effectively influenced the other’s decision. ΔINS more likely represents “regulatory investment/coordination demand”: individuals with high deception success rates may rely on more efficient, automated, or stable strategy scripts, thus requiring less cross-brain regulatory alignment to sustain influence over the partner. This interpretation fits with perspectives in interpersonal synchronization research, which posit that synchronization strength does not always linearly predict better performance but often reflects task difficulty, conflict levels, and online adjustment loads, implying a context-dependent meaning of “higher synchronization = higher coordination demand” (Cui et al., 2012; Wang et al., 2020). Hierarchical regression analysis further supported this

mechanism: regardless of the context, only ΔINS during the success phase significantly improved model explanatory power, while demographic variables, eye contact, and single-brain activation offered no significant incremental validity. Reverse entry validation confirmed that after including success-phase ΔINS, other variables provided almost no additional explanatory power. These results suggest that while traditional behavioral cues (e.g., eye contact frequency) and single-brain activation describe "individual behavior," they fail to capture the critical variance of "how both parties jointly influence the outcome," a process uniquely characterized by ΔINS (Nozawa et al., 2016).

ML analysis advanced these associations from "explanation" to "generalizable prediction." Cross-algorithm comparisons showed that the LSR model performed robustly in both contexts, indicating that ΔINS contains reproducible, cross-model robust behavioral information rather than accidental fitting. Simultaneously, the higher explanatory power and stronger negative correlation observed in gain contexts align with the aforementioned dynamic evidence that "deception in gain frames relies more on high-cost strategic monitoring and earlier synchronization differentiation." In gain contexts, INS appears to be a core variable determining individual differences, whereas in loss contexts, where behavior is driven more by approach-avoidance motivation, ΔINS remains predictive but has more limited explanatory power. This confirms that gain-loss contexts modulate not only the pathway of deception execution but also the efficiency of interactive alignment that influences successful deception.

This study has several important limitations, and interpretations should be made with caution. Firstly, the task design included a 15-second open verbal interaction phase. While this high ecological validity setting helps simulate real social exchange, it introduces semantic uncertainty that cannot be fully controlled. We operationally defined "deceptive recommendation" based on the outcome structure (payoff distribution favorable to sender/unfavorable to receiver) rather than a sentence-by-sentence semantic analysis. In the absence of verbatim transcription and coding, we cannot strictly confirm whether the sender implemented intentional misleading at the linguistic level. Future research could improve conceptual precision through voice recording, semantic coding, or explicit truth-statement tasks to distinguish "interest-biased recommendations" from "linguistic falsehoods." Secondly, despite employing multi-step preprocessing to control for motion artifacts, fNIRS signals in naturalistic interactions may still be influenced by speech-related muscle activity and head motion. Therefore, interpretations of prefrontal activation and INS should be viewed as inferences of functional patterns in ecological contexts rather than direct localization of fine-grained neural circuits. Finally, this study focused on a binary gain-loss framework and did not address complex social variables such as long-term relationships, reputation costs, or normative constraints; thus, the mechanistic explanation is limited to controlled game structures.

Summarily, by integrating behavioral data, single-brain activation, and inter-brain synchronization metrics within a high-ecological interaction setting, this study proposes and empirically validates a “Contextual Reparameterization” interactive neural model, breaking from the traditional view of deception as a static individual trait or isolated single-brain process. By revealing how gain-loss frameworks distinctly shape strategic initiation and control execution pathways, and further demonstrating that successful deception relies on the cross-brain synergy of prefrontal control networks and its predictability, this research not only deepens our understanding of the neural underpinnings of deceptive behavior but also provides a methodological basis for using interpersonal neural synchronization as a core metric for explaining and predicting interactive influence. Importantly, identifying neural indicators with incremental explanatory power and cross-model robustness under natural verbal interaction paradigms promotes the frontier transformation of social neuroscience from “correlational description” to “mechanistic modeling and behavioral prediction”.

COMMSBIO-25-4921B R2

Interpersonal Neural Synchrony in Deceptive Behavior Regulated by Gain-Loss Contexts: A fNIRS-based Hyperscanning Study

Dear Editor and Reviewers,

We sincerely appreciate the opportunity to resubmit our revised manuscript entitled **"Interpersonal Neural Synchrony in Deceptive Behavior Regulated by Gain–Loss Contexts: A fNIRS-based Hyperscanning Study"** for further consideration by *Communications Biology*. We are very grateful to Reviewer #2 and Reviewer #3 for their careful evaluation, constructive comments, and helpful suggestions. These comments have been invaluable in improving the theoretical framing, methodological clarity, statistical rigor, and overall presentation of our manuscript.

In this revision, we have carefully considered each comment and made targeted revisions throughout the manuscript. In particular, we further clarified the participant compensation procedure and ethical safeguards, corrected the description of the high-honesty exclusion criterion, refined the interpretation of the high deception rates in relation to the task incentive structure, and narrowed claims regarding deception-specific neural mechanisms. We also added additional robustness analyses for the brain–behavior correlation results, including control analyses for trial count and verbal-statement characteristics, Spearman correlation, bootstrap confidence intervals, robust regression, leave-one-dyad-out sensitivity analysis, and a direct comparison of the gain- and loss-context correlation coefficients using the cocor package in R. In addition, we carefully proofread the revised manuscript and corrected typographical, grammatical, formatting, and notation-related errors throughout the text.

All changes have been highlighted in blue in the revised manuscript. Below, we provide a point-by-point response to the reviewers' comments and indicate where the corresponding revisions can be found in the manuscript. We hope that the revised manuscript and our responses satisfactorily address the remaining concerns raised by the reviewers and meet the standards of *Communications Biology*.

Thank you again for your time, consideration, and constructive guidance. We look forward to your further evaluation of our manuscript.

Sincerely.

Author. (Double-blind review has been hidden)

Reviewer #2:

SUMMARY: *The revised version of this manuscript differs significantly from the previous submission, with an almost re-written introduction and significant changes to the analysis strategy. Therefore, this review is essentially a new review of the manuscript, rather than just checking previous concerns have been addressed. There are significant improvements to the theoretical background, the formulation of hypotheses, and the analysis approaches. However, some original concerns remain and the newly added methodological details & results raise new, substantial concerns.*

Re: We sincerely thank the reviewer for the careful and constructive re-evaluation of our substantially revised manuscript. We appreciate the reviewer's recognition that the revised version contains significant improvements in the theoretical background, hypothesis formulation, and analytical strategy. We also fully acknowledge the reviewer's concern that several original issues remain unresolved and that some newly added methodological details and results raise additional questions requiring further clarification. In response, we have carefully re-examined the manuscript and made further revisions in the following major respects:

1. **We clarified the ethical and procedural details of participant compensation**, explicitly distinguishing the guaranteed base payment from the performance-based bonus and adding a supplementary table reporting the actual bonus received by each participant.

2. **We corrected and clarified the high-honesty exclusion criterion**, making clear that the exclusion was based on an honesty rate exceeding 85%, not a deception rate below 85%.

3. **We substantially narrowed the interpretation of high deception rates**, acknowledging that they should be interpreted in light of the task incentive structure, random-payment rule, and absence of trial-by-trial feedback.

4. **We removed overstatements regarding deception-specific neural mechanisms**, because the low number of honesty trials precluded a sufficiently powered deception > honesty neural comparison.

5. **We reinterpreted honesty-trial analyses as exploratory checks only**, rather than as evidence supporting deception-specificity.

6. **We added robustness analyses for the unusually high brain–behavior correlation**, controlling for valid trial count, verbal-statement duration, and verbal-duration variability, and further testing robustness using Spearman correlation, bootstrap confidence intervals, robust regression, and leave-one-dyad-out sensitivity analysis.

7. **We expanded the Limitations section**, explicitly acknowledging the constraints imposed by the incentive structure, lack of trial-by-trial feedback, imbalance between deception and honesty trials, and limited ability to quantify semantic variability in verbal statements.

You can refer to the specific revisions and responses in the following sections.

(1) *Firstly, P10: 'Final payment was calculated as 15RMB+Rgain-Rloss, yielding a total compensation range of 0-45RMB.' – so participants could end up with not being reimbursed for their time? This would be a highly unethical practice. In itself, performance-based rewards being up to 2x the base payment is ethically questionable.*

Re: We sincerely thank the reviewer for raising this important ethical concern. We fully agree that an experimental payment scheme should not leave participants uncompensated for their time and effort. We apologize that the wording in the original manuscript did not clearly distinguish between the guaranteed base payment and the performance-based bonus.

To clarify, all participants received a guaranteed base payment of 15 RMB immediately after completing the experiment. The outcomes of the selected payoff matrices were used only to determine an additional task bonus; they were never used to reduce, cancel, or reclaim the base payment. Thus, no participant was required to repay any amount or forfeit any part of the guaranteed compensation. For the task bonus, the experimenter randomly selected one trial from the gain context and one trial from the loss context among the payoff matrices actually chosen by each dyad. The bonus was then calculated as R_{gain}-R_{loss} for each participant. If this value was 0 or less, participants received a uniform minimum bonus of 1 RMB; otherwise, they received the corresponding positive bonus. Therefore, the minimum actual total payment was 16 RMB, consisting of the 15 RMB base payment plus the 1 RMB minimum bonus, rather than 0 RMB.

To address the reviewer's concern more transparently, we have revised the manuscript to separate the guaranteed base payment from the performance-based bonus explicitly. The revised text now states that final compensation consisted of a fixed participation payment of 15 RMB and a bounded task bonus, with a minimum bonus of 1 RMB applied when the calculated bonus was non-positive. We also added a supplementary payment-distribution table reporting the actual bonus received by each participant. As shown in Table S3, the observed task bonuses ranged from 1 to 20 RMB, with a mean of 10.79 RMB and a median of 10.00 RMB. Thus, although the original formula may have implied a broader theoretical range, the actual implementation ensured that all participants were compensated within a reasonable and ethically acceptable range. No participant received 0 RMB, and the maximum total payment of 45 RMB did not occur; this value reflected a possible misunderstanding of the original formula rather than an actual payment.

According to the predefined payoff matrices used in this study, the probability of receiving the maximum task bonus was extremely low. Across all possible payoff outcomes, only 8 of 192 entries yielded 30 RMB in the gain context, whereas only 24 of 192 entries yielded 0 RMB in the loss context. Therefore, when the task bonus was calculated as R_{gain}-R_{loss}, receiving a 30 RMB bonus required both a 30 RMB gain and a 0 RMB loss to be selected

simultaneously. The joint probability of this outcome was only 0.521%. This indicates that the maximum task bonus represented a theoretical upper bound that was highly unlikely to occur in practice and therefore did not constitute an excessive performance-based incentive. Consistent with this calculation, no participant in the present study received a task bonus exceedingly twice the fixed base payment.

We also clarified that the "loss" component in the task was used only as an experimental outcome for calculating the task bonus; it did not constitute a real financial debt or repayment obligation. This design preserved the incentive structure of the gain–loss task while protecting participants guaranteed base compensation and minimizing financial risk. All participants provided informed consent before the experiment, were informed that they would receive compensation for their participation, and the study was approved by the relevant ethics committee. Moreover, the performance-based compensation structure was consistent with protocols used in previous related studies (Huang et al., 2025; Chen et al., 2020). Accordingly, we have revised the relevant sentence in the Methods section and added a supplementary payment-distribution table (Table S3) to avoid any ambiguity.

Please see the revised manuscript, page 7, line 184 to page 8, line 202, or the highlighted blue text.

Participant compensation consisted of two components. Each participant received a fixed base payment of 15 RMB upon completion of the experiment. To enhance the ecological validity of gain and loss contexts, one trial was randomly selected from each context (gain and loss) at the end of the task to determine performance-based monetary rewards or penalties. The performance-based bonus was defined as R_{gain}-R_{loss}. If this value was less than or equal to 0, participants received a uniform minimum bonus of 1 RMB; otherwise, they received the corresponding positive bonus. Under this rule, all participants received compensation for their time in addition to the fixed base payment of 15 RMB, and no participant was required to repay or forfeit any part of the base payment. In the present study, the actual task bonus ranged from 1 to 20 RMB, with a mean of 10.79 RMB and a median of 10.00 RMB. Accordingly, no participant received 0 RMB, and the extreme total payment of 45 RMB implied by the original theoretical range did not occur; the highest actual total payment was 35 RMB, received by 12 participants. All experimental procedures and payment rules were clearly explained to participants prior to the experiment, and no participant was required to incur any additional costs. The performance-based compensation structure followed the protocol established in previous studies (Huang et al., 2025; Chen et al., 2020). The final bonus received by each participant in the present study is reported in **Table S3**.

The detailed payment distribution is reported in Table S3 of the Supplementary Materials and is also provided below for reference.

Table S3. The final bonuses received by participants in the present study.

Dyad_ID	Role	R_gain	R_loss	Raw_Bonus	Adjusted_Bonus	Final_Bonus
**1**	Sender	1	1	0	1	1
**1**	Receiver	0	0	0	1	1
**2**	Sender	20	20	0	1	1
**2**	Receiver	25	21	4	4	4

**3**	Sender	15	11	4	4	4
**3**	Receiver	10	10	0	1	1
**4**	Sender	16	11	5	5	5
**4**	Receiver	15	10	5	5	5
**5**	Sender	0	0	0	1	1
**5**	Receiver	10	1	9	9	9
**6**	Sender	30	21	9	9	9
**6**	Receiver	20	20	0	1	1
**7**	Sender	10	0	10	10	10
**7**	Receiver	15	10	5	5	5
**8**	Sender	15	10	5	5	5
**8**	Receiver	10	0	10	10	10
**9**	Sender	20	25	-5	1	1
**9**	Receiver	30	15	15	15	15
**10**	Sender	25	10	15	15	15
**10**	Receiver	20	20	0	1	1
**11**	Sender	30	10	20	20	20
**11**	Receiver	20	20	0	1	1
**12**	Sender	10	10	0	1	1
**12**	Receiver	20	0	20	20	20
**13**	Sender	5	1	4	4	4
**13**	Receiver	6	0	6	6	6
**14**	Sender	16	10	6	6	6
**14**	Receiver	15	11	4	4	4
**15**	Sender	10	1	9	9	9
**15**	Receiver	11	0	11	11	11
**16**	Sender	16	5	11	11	11
**16**	Receiver	15	6	9	9	9
**17**	Sender	15	1	14	14	14
**17**	Receiver	5	0	5	5	5
**18**	Sender	5	0	5	5	5
**18**	Receiver	15	1	14	14	14
**19**	Sender	25	10	15	15	15
**19**	Receiver	20	11	9	9	9
**20**	Sender	10	1	9	9	9
**20**	Receiver	15	0	15	15	15
**21**	Sender	30	10	20	20	20
**21**	Receiver	20	15	5	5	5
**22**	Sender	15	10	5	5	5
**22**	Receiver	25	5	20	20	20
**23**	Sender	21	5	16	16	16
**23**	Receiver	20	10	10	10	10
**24**	Sender	15	5	10	10	10
**24**	Receiver	16	0	16	16	16

**25**	Sender	15	0	15	15	15
**25**	Receiver	20	5	15	15	15
**26**	Sender	25	5	20	20	20
**26**	Receiver	20	10	10	10	10
**27**	Sender	15	5	10	10	10
**27**	Receiver	20	0	20	20	20
**28**	Sender	25	6	19	19	19
**28**	Receiver	20	5	15	15	15
**29**	Sender	15	0	15	15	15
**29**	Receiver	20	1	19	19	19
**30**	Sender	20	0	20	20	20
**30**	Receiver	25	10	15	15	15
**31**	Sender	15	0	15	15	15
**31**	Receiver	25	5	20	20	20
**32**	Sender	20	0	20	20	20
**32**	Receiver	21	5	16	16	16
**33**	Sender	21	5	16	16	16
**33**	Receiver	20	0	20	20	20
**34**	Sender	20	0	20	20	20
**34**	Receiver	21	1	20	20	20

Note: The final amounts reported in this table do not include the fixed base participation compensation of 15 RMB received by each participant.

Reference

[1] Huang, R., Gao, X., Zhang, C., ..., & Liu, Y. (2025). Forewarned Is Forearmed: The Single-and Dual-Brain Mechanisms in Detectors from Dyads of Varying Social Distance during Deceptive Outcome Evaluation. *Journal of Neuroscience*, *45*(43), e2129242025.

[2] Chen, M., Zhang, T., Zhang, R., ..., & Li, X. (2020). Neural alignment during face-to-face spontaneous deception: does gender make a difference? *Human Brain Mapping*, *41*(17), 4964-4981.

---

(2) P9: 'two dyads were excluded based on a predefined high-honesty criterion (deception rate<85%)' – is this an error, i.e. did the authors mean an HONESTY rate of >85%? Otherwise, if to be included in analysis, dyads had to show deception on 85% of trials, this contradicts the author's claim of deception being a cognitively demanding, deliberate, goal-oriented decision-making process; or at least it wouldn't be in the context of the task used. Deception would be the default response, and Honesty a rare occurrence either done by mistake or to introduce some variability.

Re: We thank the reviewer for identifying this important inconsistency. The reviewer is correct that the original wording was erroneous. The exclusion criterion was not “deception rate < 85%.” Rather, the predefined criterion was a high-honesty criterion: an honesty rate > 85%, equivalent to a deception rate < 15%. We apologize for this incorrect wording.

The rationale for this criterion was to exclude dyads that rarely made objectively disadvantageous recommendations to the receiver. Such dyads provided too few deception trials to support reliable behavioural, fNIRS activation, and interpersonal neural synchrony analyses of deceptive interaction. Importantly, this exclusion rule was not intended to select “high-deception” dyads or to make deception the default response. Instead, it was applied to remove cases of extremely high honesty in which the psychological and neural processes underlying deception could not be meaningfully modelled. The corresponding revision in the Participants **(page 7, lines 170 to 172)** reads as follows: “two dyads were excluded based on a predefined high-honesty criterion, defined as an honesty rate exceeding 85% across the experiment, equivalent to a deception rate below 15% (Chen et al., 2020).”

To avoid ambiguity, we have revised the Participants and Behavioural Data Analysis sections. The revised manuscript now states that two dyads were excluded because their honesty rates exceeded 85% across the experiment. We also clarified that deception was operationalized as an objectively receiver-disadvantageous recommendation under the payoff structure, rather than inferred directly from the sender’s subjective intention.

Please see the revised manuscript, page 14, lines 344 to 348, or the highlighted blue text.

To ensure that deception-related behavioral and neural analyses were based on a sufficient number of deceptive trials, dyads with an honesty rate exceeding 85% across the experiment were excluded according to the predefined high-honesty criterion (Chen et al., 2020). This criterion was used only to remove extreme high-honesty cases and was not intended to select dyads with unusually high deception rates.

We further agree with the reviewer that deception should not be described as uniformly cognitively demanding in all contexts. We have therefore refined our theoretical framing to emphasize that deception is a context-sensitive decision process whose cognitive demands vary across gain and loss contexts. In the present task, the relatively high deception rate reflects the direct conflict-of-interest payoff structure. However, the response-time and neural results indicate that deception was not a simple default response: loss contexts were associated with faster and more frequent deception, whereas gain contexts elicited stronger DLPFC activation during deception, suggesting greater strategic monitoring and executive control under gain framing.

(3) Deception rates are extremely high (>90%). This is likely linked to the payment structure, i.e. one trial being selected randomly for payoff. In a directly relevant setting, where every decision affects final payoff, participants can be expected to make trial-wise decisions, for example aiming for equity by sometimes recommending a choice disadvantageous to themselves. If only one trial is counted for the payoff, the rational thing to do is to determine the best strategy on the first trial and stick with that strategy throughout the task. This is particularly relevant given no feedback was provided, such that participants had no opportunities to learn about their partner's behaviour. As such, the deceiver's behaviour likely is not reflecting a trial-wise decision-making process as to whether to deceive or not.

Re: We sincerely thank the reviewer for this important comment. We agree that the high deception rates observed in the present study should be interpreted in relation to the task incentive structure, including the direct conflict-of-interest payoff matrices, the random selection of one trial from each context for payment, and the absence of trial-by-trial feedback. We also agree that these design features limit the extent to which sender behavior can be interpreted as a fully independent trial-wise decision process in which participants repeatedly reconsider whether to deceive or act honestly on each trial.

In response, we have revised the manuscript to avoid overinterpreting the behavioral data as evidence for independent trial-by-trial decisions to deceive. Specifically, we now clarify that the present paradigm was designed to elicit receiver-disadvantageous recommendations under controlled gain–loss contexts, rather than to model adaptive learning or repeated strategy updating. Although senders responded to different payoff matrices on each trial, the random-payment rule and absence of feedback mean that the observed behavior is more appropriately interpreted as the trial-level implementation and outcome of a generally adopted strategic tendency.

We made several revisions accordingly. In the Methods section, we clarified that the random-payment rule preserved task-level incentive compatibility while avoiding excessive variability in participant compensation, but that the task was not designed to assess learning-based adjustment of deception strategies. In the task procedure, we revised the no-feedback description to state that the design minimized explicit punishment, reputation management, and feedback-based learning, while precluding conclusions about adaptive trial-by-trial updating. In the Results section, we replaced wording such as "trial-wise decision-making process" with more precise terms such as "trial-level implementation" and "outcomes of receiver-disadvantageous recommendations." Finally, in the Discussion and Limitations sections, we explicitly acknowledge that the high deception rate may partly reflect the incentive structure, and we note that future studies should incorporate cumulative trial-wise payment and trial-by-trial feedback to examine dynamic updating of deceptive and honest strategies across repeated interactions.

Please see the revised manuscript, page 33, line 805 to page 34, line 814, or the highlighted blue text.

However, the high deception rates observed in the present study should be interpreted in light of the task incentive structure. The payoff matrices created a direct conflict of interest between the sender and receiver, and only one trial from each context was randomly selected for final payment. This design was useful for eliciting sufficient receiver-disadvantageous recommendations for neural analysis while limiting excessive variability in participant compensation. However, it also means that senders may have adopted a relatively stable strategic tendency across trials, rather than independently reconsidering honesty versus deception on every trial. Thus, the present behavioral results should not be interpreted as evidence for fully independent trial-wise decisions to deceive. Instead, they indicate that gain–loss framing modulated the frequency, execution, and success of receiver-disadvantageous recommendations under a high-conflict incentive structure.

Please see the revised manuscript, page 37, line 925 to page 38, line 931, or the highlighted blue text.

Another limitation concerns the incentive, feedback, and trial-distribution structure of the task. In the present design, one trial from each context was randomly selected for payment, and senders did not receive trial-by-trial feedback regarding whether their recommendations influenced receivers' decisions. Although this design reduced explicit reputation management and prevented feedback-based learning from confounding the neural measures, it may also have encouraged a relatively stable response strategy and limited the extent to which deception can be interpreted as an adaptive trial-wise decision process.

---

(4) P18: 'Because the primary objective of the present study was to characterize neural response patterns associated with deceptive interaction, fNIRS analyses were restricted to trials in which senders chose to engage in deception.' This was raised in my original review and has not been sufficiently addressed. To draw any conclusions regarding deception, all analyses must be based on the deception>honesty comparison. Without this, all the authors can say is how brain activity differs between gain vs. loss contexts, but this may be true for any decision-making, involving deception or not. Of course, the extremely low honesty rate in the given paradigm would make such an analysis difficult. This, however, reflects the limiting nature of the task used, as it does not allow for testing the neural basis of deception (given deception is essentially present on every trial).

Re: We sincerely thank the reviewer for raising this important concern again. We fully agree that a rigorous test of deception-specific neural mechanisms would require a direct and sufficiently powered deception> honesty comparison. We also agree that, because honesty trials were extremely sparse in the present paradigm, the current data cannot provide a definitive test of the neural basis of deception per se. The reviewer's comment helped us

recognize that our previous wording overstated the scope of the neural conclusions and did not sufficiently acknowledge the inferential limitation imposed by the imbalance between deceptive and honest trials.

In the present study, deception occurred on more than 90% of trials, whereas honesty accounted for only approximately 8% of trials. This imbalance made it statistically unreliable to estimate fNIRS activation, task-level INS, trial-level ΔINS, or predictive models for honesty trials with the same reliability as for deceptive trials. Therefore, the original statement that "fNIRS analyses were restricted to trials in which senders chose to engage in deception" was methodologically accurate but theoretically insufficiently qualified. We agree that the resulting neural effects should not be interpreted as evidence for deception-specific activation or as identifying the neural basis of deception in general. Rather, these analyses can support a more limited conclusion: they characterize how gain–loss contexts modulate neural activity and interpersonal neural synchrony during receiver-disadvantageous recommendations in a high-conflict interaction task.

To address this concern, we have substantially revised the manuscript to narrow the scope of our claims.

First, in the **Abstract**, we replaced the broad statement that "deception elicited higher DLPFC activation in gain compared with loss contexts" with a more precise formulation (**in the revised manuscript page 1, lines 12-14**): **"**During receiver-disadvantageous recommendations, gain contexts were associated with higher dorsolateral prefrontal cortex (DLPFC) activation than loss contexts.**"** This revision avoids implying that we tested a deception> honesty neural contrast.

Second, in the **Introduction**, we revised the statement of the study aim. Rather than stating that the study identifies neural mechanisms of deception broadly, we now specify that the study examined how control-related PFC dynamics at both individual and dyadic levels were modulated by gain–loss context during receiver-disadvantageous recommendations, and how these dynamics were associated with the success of such recommendations. Specifically, the revised manuscript (**page 6, lines 154-158**) states: **"**Using fNIRS hyperscanning, we focused on the interaction phase to examine how control-related PFC dynamics—at both individual and dyadic levels—were modulated by gain–loss context during receiver-disadvantageous recommendations and how these dynamics were associated with the success of such recommendations.**"** This change better aligns the theoretical framing with the actual design and analysis.

Third, in the **Methods section**, we revised the fNIRS analysis rationale. The original sentence suggested that deception trials were selected simply because the primary objective was to characterize neural responses associated with deceptive interaction. We now explicitly state that honesty trials were too sparse to provide sufficient observations for reliable estimation of fNIRS activation, task-level INS, or trial-level predictive models. Accordingly,

the revised manuscript (**page 14, lines 359-364**) states: **"**Because honesty trials were extremely sparse in the present paradigm, they did not provide sufficient observations for reliable estimation of fNIRS activation, task-level INS, or trial-level predictive models. Therefore, the primary fNIRS analyses focused on trials in which senders provided receiver-disadvantageous recommendations. These analyses were intended to examine context-dependent neural modulation within deceptive interactions, rather than to provide a direct deception > honesty contrast.**"**

Fourth, in the **Results section**, we revised the wording of the intra-brain activation results. Instead of stating that we examined whether "deception interactions" were associated with context-dependent PFC modulation, the revised manuscript (**page 23, lines 603-606**) now states: **"**We next examined whether PFC hemodynamic responses during receiver-disadvantageous recommendations were modulated by gain–loss context. Given the low number of honesty trials, the primary analysis focused on deceptive recommendation trials, with HbO responses extracted from the 15-s verbal statement phase.**"**

We also added a cautionary statement regarding the exploratory honesty-trial analysis (**see the revised manuscript page 24, lines 609-613**): **"**As an exploratory check, we also examined contextual differences during honesty trials. No significant contextual differences were observed in any channel (ps> 0.162; Table S2). However, given the small number of honesty trials, this analysis should be interpreted cautiously and was not treated as a sufficiently powered deception> honesty comparison.**"**

Fifth, in the **Discussion**, we revised several passages to avoid overclaiming deception-specificity. For example, the revised manuscript states (**page 32, lines 772-776**): **"**At the single-brain level, DLPFC activation was higher during receiver-disadvantageous recommendation trials in gain contexts than in loss contexts. This finding indicates context-dependent modulation of PFC responses within deceptive interactions, but it should not be interpreted as a deception-specific effect because the low number of honesty trials precluded a sufficiently powered deception> honesty comparison.**"**

We also replaced the earlier claim that contextual differences were "specific to deception trials" with the following more cautious wording (**see the revised manuscript page 34, lines 815-818**): **"**At the single-brain level, contextual differences during receiver-disadvantageous recommendation trials were concentrated in the DLPFC. Although no significant contextual modulation was detected in honesty trials, the very small number of honesty trials precludes strong claims about deception-specificity.**"**

Finally, in the **Limitations**, we added an explicit limitation addressing this issue. The revised manuscript (**page 38, lines 931-943**) states: **"**Moreover, the payoff structure elicited very high deception rates, leaving too few honesty

trials to support reliable fNIRS activation, INS, or predictive modeling analyses. Consequently, the present study cannot provide a sufficiently powered deception > honesty neural comparison and should not be interpreted as identifying the neural basis of deception per se. Rather, the findings characterize how gain–loss contexts modulate neural activity and interpersonal neural synchrony during receiver-disadvantageous recommendations in a high-conflict interaction task. Future studies should incorporate task designs that yield a more balanced distribution of honest and deceptive choices, such as cumulative trial-wise payment, trial-by-trial feedback about whether the receiver adopted the sender's recommendation and how the outcome affected both players' payoffs, stronger incentives for honest recommendations, or experimentally instructed honesty and deception conditions. Such designs would allow researchers to examine dynamic strategy updating across repeated interactions and directly test deception-specific neural mechanisms.**"**

---

(5) The authors now include effects for analyses focusing exclusively on honest trials. However, it is unclear if they had enough trials to run this analysis (presumably, with ~5-10 trials per dyad & condition, this would have been very noisy data), and it does not adequately support the claims made as to the specificity of the effects for deception trials. Without using the correct difference score (deception > honesty) as the basis for the gain > loss comparison, it is not valid to conclude that effects are specific to deception trials only.

Re: We thank the reviewer for further clarifying this issue. We agree that the honesty-trial analyses were underpowered because honesty trials were very sparse, and therefore these analyses cannot provide decisive evidence for deception-specificity. We also agree that, without using a deception> honesty difference score as the basis for the gain> loss comparison, it is not valid to conclude that the observed effects are specific to deception trials.

Accordingly, we have removed statements implying a deception-specific neural basis. The honesty-trial analyses are now described only as exploratory checks. We now interpret the findings more conservatively as evidence that gain–loss contexts modulated neural activity and interpersonal neural synchrony during receiver-disadvantageous recommendations, particularly when such recommendations were adopted. In the **Methods** section, we further clarified that the small number of honesty trials precluded reliable estimation of fNIRS activation, task-level INS, or trial-level predictive models; thus, the primary neural analyses examined context-dependent modulation within deceptive interactions rather than a direct deception> honesty contrast.

Please see the revised manuscript, page 14, lines 359-364, or the highlighted blue text.

Because honesty trials were extremely sparse in the present paradigm, they did not provide sufficient

observations for reliable estimation of fNIRS activation, task-level INS, or trial-level predictive models. Therefore, the primary fNIRS analyses focused on trials in which senders provided receiver-disadvantageous recommendations. These analyses were intended to examine context-dependent neural modulation within deceptive interactions, rather than to provide a direct deception> honesty contrast.

In the **INS Results** section, we removed the original statement implying deception-specificity and replaced it with a more cautious interpretation of gain–loss modulation during successful receiver-disadvantageous recommendations.

Please see the revised manuscript, page 26, lines 660-664, or the highlighted blue text:

These results indicate that gain–loss framing modulated ΔINS during successful receiver-disadvantageous recommendations. However, given the limited number of honesty trials and the absence of a sufficiently powered deception> honesty contrast, this pattern should not be interpreted as demonstrating deception-specificity.

In the **Discussion** section, we further refined the interpretation of the single-brain findings to avoid overstating deception-specificity.

Please see the revised manuscript, page 32, lines 772-776, or the highlighted blue text.

At the single-brain level, DLPFC activation was higher during receiver-disadvantageous recommendation trials in gain contexts than in loss contexts. This finding indicates context-dependent modulation of PFC responses within deceptive interactions, but it should not be interpreted as a deception-specific effect because the low number of honesty trials precluded a sufficiently powered deception> honesty comparison.

Through these revisions, the revised manuscript no longer uses the potentially noisy honesty-only analyses to support claims of deception-specificity. Instead, the interpretation is strictly limited to gain–loss modulation of neural activity and interpersonal neural synchrony during receiver-disadvantageous recommendations in a high-conflict interaction paradigm.

---

(6) P36, Brain-Behaviour Correlation: the observed correlation between inter-brain connectivity in successful deception gain trials and successful deception rate is suspiciously high, showing an almost perfect (negative) relationship. This suggests it may be driven by methodological confounds, e.g. number of trials included in the analysis, length of verbal statements made, trial-to-trial variability in statements made, etc..

Re: We thank the reviewer for this important and well-justified concern. We agree that the zero-order correlation observed in the gain context was unusually large and should not be interpreted without further robustness checks. In particular, we recognized that this association could potentially be inflated by methodological or behavioral factors, including the number of valid trials contributing to the ΔINS estimate, the duration of verbal statements, variability in verbal-statement duration across trials, or the influence of one or a few dyads with extreme values.

To address this concern, we revised the analysis strategy rather than relying solely on the original Pearson correlation. Specifically, we first quantified, for each dyad and condition, the number of valid trials included in the ΔINS estimate, the mean duration of verbal statements, and the within-condition variability in verbal-statement duration. We then repeated the brain-behavior analysis using partial correlations that controlled for these factors. This analysis allowed us to test whether the association between DLPFC ΔINS and successful deception rate remained after accounting for the most direct methodological confounds identified by the reviewer.

Please see the revised manuscript, page 17, lines 441-449, or the highlighted blue text.

For each dyad and condition, we calculated the number of valid trials, mean verbal-statement duration, and within-condition variability in verbal-statement duration, and then repeated the brain–behavior analyses using partial correlations controlling for these variables. Because verbatim transcripts were unavailable, semantic variability in verbal content could not be directly quantified; therefore, verbal-duration variability and direct eye-contact frequency were used as behavioral proxies for interaction variability. Spearman correlations, bootstrap confidence intervals, robust regression, and leave-one-dyad-out analyses were further conducted to assess whether the associations were driven by distributional assumptions, outliers, or influential dyads.

The association remained significant after these controls were included. Specifically, the relationship between DLPFC ΔINS during successful receiver-disadvantageous recommendations and successful deception rate in the gain context remained significant after controlling for valid trial count, mean verbal-statement duration, and verbal-duration variability (partial r=−0.731, p<0.001). Thus, the association was not fully explained by the number of trials entering the ΔINS estimate or by basic temporal characteristics of the verbal interaction. We further examined whether the result depended on distributional assumptions or influential observations. The association was supported by Spearman rank correlation (ρ=−0.774, p<0.001), indicating that the effect was not limited to the assumptions of Pearson correlation. Bootstrap resampling also yielded a confidence interval that did not include zero (95% CI [-0.888, -0.571]). In addition, robust regression produced a significant negative association (β=−0.491, p<0.001). Finally, leave-one-dyad-out sensitivity analysis showed that the correlation remained stable when each dyad was removed in turn, with r values ranging from −0.952 to −0.929. These results indicate that the original association was not driven by a single influential dyad.

Please see the revised manuscript, page 29, line 713-722, or the highlighted blue text.

Because the zero-order correlation in the gain context was unusually large, we conducted additional robustness analyses to examine whether this association was driven by methodological or behavioral confounds. After controlling for the number of valid trials, mean verbal-statement duration, and variability in verbal-statement duration, the association between DLPFC ΔINS during successful receiver-disadvantageous recommendations and successful deception rate remained significant (partial r =-0.731, p<0.001). This association was further supported by Spearman rank correlation (ρ=-0.774, p<0.001), bootstrap resampling (95% CI [-0.888, -0.571]), and robust regression (β=-0.491, p<0.001). Leave-one-dyad-out sensitivity analysis further showed that the correlation remained stable after each dyad was excluded in turn, with r values ranging from -0.952 to -0.929.

We have revised both the Methods and Results sections accordingly. In the Methods, we now describe the additional robustness analyses, including control of trial count and verbal-statement characteristics, as well as Spearman correlation, bootstrap confidence intervals, robust regression, and leave-one-dyad-out sensitivity analysis. In the Results, we now report these robustness checks directly alongside the original brain–behavior correlation. We also clarified that verbatim transcripts were not available, so trial-to-trial semantic variability in verbal content could not be directly quantified; therefore, verbal-duration variability and direct eye-contact frequency were used as available behavioral proxies for interaction variability.

Reviewer #3:

SUMMARY: *In my opinion, the authors have adequately and thoroughly addressed most of my concerns. Overall, however, I noticed a few minor typographical errors in the revised manuscript; it would be advisable to proofread the text once more.*

Re: We thank the reviewer for the positive evaluation and for noting the remaining typographical issues. We have carefully proofread the revised manuscript again and corrected typographical, grammatical, formatting, and notation-related errors throughout the text. These include corrections to hyphenation and spacing, subject–verb agreement, statistical notation, figure captions, formula presentation, and several minor wording inconsistencies. We have also ensured that the terminology used in the Abstract, Methods, Results, and Discussion is consistent with the revised interpretation of the findings.

---

(1) Regarding my point 6, the authors could consider adding a statistical comparison of the two regression coefficients, which is relatively straightforward to conduct (e.g., using appropriate R packages or the procedure described here: https://www.psychometrica.de/correlation.html).

Re: We sincerely thank the reviewer for this important and constructive suggestion. We agree that our previous revision, although it reported the brain-behavior correlations separately for gain and loss contexts, did not yet provide a formal statistical test of whether the two context-specific associations differed in strength. We also agree that simply observing a stronger correlation in the gain context than in the loss context is not sufficient to support a context-difference interpretation. A direct comparison of the two coefficients is therefore necessary.

In response, we added a formal comparison of the gain- and loss-context brain–behavior associations. Because both correlations were computed from the same dyads, they are statistically dependent rather than independent. Moreover, the two correlations do not share identical variables: the gain-context coefficient reflects the association between gain-context DLPFC ΔINS and gain-context successful deception rate, whereas the loss-context coefficient reflects the association between loss-context DLPFC ΔINS and loss-context successful deception rate. Therefore, we treated the two coefficients as dependent, non-overlapping correlations and compared them using the cocor package in R. This procedure uses the full correlation structure among the four relevant variables and provides a direct test of whether the two context-specific brain–behavior associations differ significantly.

The newly added analysis showed that the association between DLPFC ΔINS and successful deception rate was significantly stronger in the gain context than in the loss context. Specifically, the dependent-correlation comparison

yielded Pearson and Filon's z=−3.487, p<0.001, and Zou's 95% confidence interval for the difference between the two correlations excluded zero, 95% CI [-0.816, -0.243]. Thus, the stronger gain-context association is supported by a direct statistical comparison, rather than by separate within-context significance tests alone.

We have revised both the Methods and Results sections accordingly. In the Methods section, we added the following description of the coefficient-comparison procedure, **please see the revised manuscript, page 17, lines 450-455, or the highlighted blue text.**

To test whether the brain–behavior association differed between gain and loss contexts, we compared the two context-specific correlations using the cocor package in R. Because both correlations were derived from the same dyads, they were treated as dependent correlations. We therefore used the non-independent correlation comparison implemented in cocor to examine whether the correlation between DLPFC ΔINS and successful deception rate differed significantly between the gain and loss contexts.

In the Results section, we now report the direct comparison as follows **(the revised manuscript, page 28, line 706 to page 29, line 712)**: "We further directly compared the two context-specific brain–behavior correlations. Because the two correlations were obtained from the same dyads but did not share variables, they were compared as dependent, non-overlapping correlations using the cocor procedure. The gain-context association was significantly stronger than the loss-context association, Pearson and Filon's z=−3.487, p<0.001, with Zou's 95% confidence interval for the difference excluding zero, 95% CI [-0.816, -0.243]. This result indicates that the DLPFC ΔINS–successful deception rate association was significantly stronger in the gain context than in the loss context."

This revision strengthens the statistical basis of our interpretation. In the previous version, we could only state that the gain-context association appeared numerically stronger. After adding the coefficient-comparison analysis, we can now conclude more appropriately that the gain-context brain–behavior coupling was statistically stronger than the corresponding loss-context coupling. At the same time, we have retained a cautious interpretation: the association was present in both contexts, but its strength was significantly greater in the gain context.

---

(2) From my side, after these minor revisions, I would not recommend another round of review.

Re: We sincerely thank the reviewer for this encouraging assessment and for indicating that another round of review would not be necessary after these minor revisions. We greatly appreciate the reviewer's constructive and efficient evaluation of the revised manuscript. In response, we carefully proofread the entire manuscript once more and corrected the remaining typographical, grammatical, formatting, and notation-related errors. We also added the

requested statistical comparison of the gain- and loss-context brain–behavior coefficients using the cocor package in R, thereby strengthening the statistical basis for our interpretation of context differences. These revisions were made to ensure that the manuscript is clearer, more internally consistent, and methodologically more rigorous.

COMMSBIO-25-4921B R2

Original Title: Interpersonal Neural Synchrony in Deceptive Behavior Regulated by Gain-Loss Contexts: A fNIRS-based Hyperscanning Study

Revised title: Loss Contexts Enhance Dorsolateral Prefrontal Interpersonal Neural Synchrony During Successful Human Deceptive Recommendations

Dear Editor and Reviewers,

We sincerely appreciate the opportunity to submit our revised manuscript entitled "**Loss Contexts Enhance Dorsolateral Prefrontal Interpersonal Neural Synchrony During Successful Human Deceptive Recommendations**" for further consideration by *Communications Biology*. We are especially grateful to the Editorial Office for the continued consideration of our work for publication, and to the reviewer for the careful evaluation and constructive comments. The reviewer's remaining concern has been highly valuable in helping us further improve the clarity, rigor, and presentation of the manuscript.

In this revision, we have carefully addressed the reviewer's comment and made the corresponding changes in the revised manuscript. We believe that this issue has now been satisfactorily resolved. All relevant changes have been highlighted in blue in the revised manuscript, and a point-by-point response is provided below.

Thank you again for your time, consideration, and constructive guidance. We greatly appreciate the opportunity to have our work considered for publication in *Communications Biology*.

Sincerely.

He Wang (Co-corresponding author 1)

Yingjie Liu (Co corresponding author 2)

Reviewer #2:

SUMMARY: *In their revisions, the authors have addressed my concerns raised in the previous review. Their interpretation of findings is now more in line with the presented data. It should be noted that this somewhat reframes the implications of this research, as conclusions can be drawn about differences between gain-loss contexts, but little can be said about any specificity to the social context of deception. I have some minor comments below, for the authors' consideration.*

Re: We sincerely thank the reviewer for the positive and constructive assessment of our revised manuscript. We appreciate the reviewer's recognition that our revisions have addressed the major concerns raised in the previous review and that our interpretation is now more closely aligned with the presented data.

In response to the reviewer's summary, we have further clarified the scope and implications of the study in three ways:

1. We have reframed the main conclusion more cautiously. The revised manuscript no longer presents the findings as evidence for deception-specific neural mechanisms. Instead, we now frame the study as showing how gain-loss contexts modulate behavior, prefrontal activation, and interpersonal neural synchrony during receiver-disadvantageous recommendations in a high-conflict interactive task.

2. We have further limited speculative interpretations. Given the absence of speech recordings and verbatim semantic coding, we clarified that deception was operationally defined by the payoff structure rather than by confirmed linguistic falsehoods or direct inference of subjective intent.

3. We have made additional minor revisions following the reviewer's comments. Specifically, we corrected the cross-reference error, rechecked all figure and table citations, removed the over-interpretive sentence "deception is not a fixed individual trait," and made the Discussion section more concise and focused.

We are grateful for the reviewer's careful reading and helpful suggestions. Below, we provide point-by-point responses to the remaining comments.

---

(1) On page 27, there is a referencing error ('Performance metrics for all conditions and 679 models are summarized in Table 6 and Error! Reference source not found.')

Re: We thank the reviewer for this careful and important comment. Following the reviewer's comments and the editorial office's requirements, we have thoroughly checked all figure and table citations throughout the revised manuscript. We carefully examined the correspondence between the in-text citations, figure panels, table numbers, figure legends, and supplementary materials, and corrected any inconsistent or unclear references where necessary.

Specifically, we verified that each main-text figure and table is cited in the appropriate section and that the cited panels correspond to the results described in the text. We also checked the figure legends and table titles to ensure that they accurately describe the corresponding analyses, including the behavioral results, reaction-time analyses, intra-brain activation results, task-level ΔINS results, trial-level ΔINS dynamics, experimental design, direct eye-contact control analysis, machine-learning model performance, and anatomical correspondence of fNIRS channels. In addition, we reviewed the citations of supplementary figures and supplementary tables to ensure consistency between the main text and supplementary materials.

We appreciate the reviewer's careful reading, which helped us improve the accuracy and clarity of the manuscript presentation.

---

(2) In terms of discussing the results, a major limitation is that speech recordings are not available. As such, much of the interpretation of the fNIRS & hyperscanning results remains speculative (e.g. did dyads with high deception rates have a consistent strategy; are there (m)any cases of logged deception actually based on honest communication, e.g. 'I will recommend the better choice for myself now and you select that, next trial we'll switch'). This is acknowledged in the limitations section, but combined with the above limitations of the task design, some statements such as 'deception is not a fixed individual trait' could be phrased more cautiously.

Re: We thank the reviewer for this important and constructive comment. We have removed the over-interpretive sentence "deception is not a fixed individual trait" from the revised manuscript. We agree that, because speech recordings and verbatim transcripts were unavailable, we cannot determine the exact semantic content of the sender's statements or distinguish all possible communicative strategies at the trial level. Therefore, we have substantially tempered our interpretation of the fNIRS and hyperscanning findings and clarified that our task captures receiver-disadvantageous recommendations under a controlled payoff structure, rather than verified linguistic falsehoods or fully confirmed subjective deception.

To address this concern, we revised the Methods section to make the operational definition of deception explicit. Specifically, we now state (**see revised manuscript page 24, lines 643-648, or subsequent blue marked text**): "Because the semantic content of verbal statements was not constrained, deception could not be conclusively identified at the single-trial level. Accordingly, deception was operationally defined based on whether the sender's recommendation was objectively disadvantageous for the receiver given the payoff structure, rather than on direct inference of the sender's subjective intent." We believe this clarification directly addresses the reviewer's concern

that some trials classified as deception could have involved communicative strategies that were not linguistically false in a strict sense.

We also clarified that the task design was not intended to model adaptive trial-by-trial strategy updating. In the revised manuscript (**page 24, lines 639-643, or subsequent blue marked text**), we state: "This design minimized explicit punishment, reputation management, and feedback-based learning. Accordingly, the present task was intended to capture how receiver-disadvantageous recommendations were implemented under controlled gain-loss contexts, rather than to model adaptive trial-by-trial strategy updating based on outcome feedback." In the Discussion (**see revised manuscript page 16, lines 420-423, or subsequent blue marked text**), we further acknowledge that "because the payoff matrices created direct conflicts of interest and only one trial per context was selected for payment, senders may have developed relatively stable strategic tendencies across trials." We therefore revised the relevant conclusion to a more cautious form (**see revised manuscript page 16, lines 423-425, or subsequent blue marked text**): "Thus, the results indicate that gain-loss framing modulated the frequency, speed, and success of receiver-disadvantageous recommendations, rather than providing evidence for fully independent trial-wise decisions to deceive."

Regarding the interpretation of the hyperscanning results, we have also narrowed our claims. The revised Discussion now states (**page 17, lines 450-455, or subsequent blue marked text**): "Because speech recordings were unavailable, however, we cannot determine whether enhanced DLPFC ΔINS reflected specific verbal tactics, stable dyadic strategies, or moment-to-moment persuasive adjustments. Therefore, DLPFC ΔINS should be interpreted as evidence for dyadic control alignment during successful receiver-disadvantageous influence, rather than as a marker of a specific communicative strategy." This revision explicitly avoids attributing the observed ΔINS effect to a particular linguistic or strategic mechanism.

Finally, we strengthened the limitation section to make this caveat clear. We now state (**see revised manuscript page 18, lines 486-489, or subsequent blue marked text**): "Because speech recordings and verbatim coding were unavailable, deception was defined operationally by the payoff structure—that is, receiver-disadvantageous recommendations—rather than by confirmed linguistic falsehoods." We also added a broader caution that "this study should not be interpreted as identifying the neural basis of deception per se, but as showing how gain-loss contexts modulate prefrontal activity and INS during receiver-disadvantageous recommendations in a high-conflict interaction task." **(page 18, lines 497-500)** We further note that "future studies should incorporate speech recording, semantic coding, trial-wise feedback, and more balanced honest/deceptive choices to test deception-specific mechanisms more directly." **(page 18, lines 500-502)**

Together, these revisions remove the overly broad trait-level interpretation, make the behavioral classification more transparent, and constrain the neural interpretation to the evidence directly supported by the present design.

---

(3) It may also be useful to check whether the discussion section can be written somewhat more concisely.

Re: We thank the reviewer for this helpful comments. We have carefully revised the Discussion section to improve concision and readability. In the revised manuscript, we removed redundant explanations, shortened speculative interpretations, and reorganized the Discussion around the main empirical findings: behavioral modulation by gain-loss contexts, context-dependent DLPFC activation, DLPFC △INS during successful receiver-disadvantageous recommendations, and the predictive value of △INS.

In particular, we condensed repeated descriptions of the behavioral and neural results and avoided restating statistical details that were already reported in the Results section. We also shortened the interpretation of the hyperscanning findings and made the relevant claims more cautious. For example, the revised Discussion now states that "gain-loss contexts shape deception-related outcomes not only through individual prefrontal regulation, but also through the alignment of control-related processes between interaction partners."

We also integrated the limitations more directly into the interpretation rather than presenting extensive speculative discussion. For example, we now explicitly state that "this study should not be interpreted as identifying the neural basis of deception per se, but as showing how gain-loss contexts modulate prefrontal activity and INS during receiver-disadvantageous recommendations in a high-conflict interaction task." These revisions make the Discussion more concise while preserving the central theoretical contribution and the necessary methodological cautions.

We appreciate the reviewer's suggestion, which helped us improve the clarity, focus, and balance of the Discussion section.

Please see the revised manuscript, page 15, line 388 to page 19, line 511, or the highlighted blue text.

However, The present study provides evidence that deception-related behavior, as operationalized by receiver-disadvantageous recommendations in the present task, is modulated by gain-loss contexts during social interaction. Behaviorally, loss contexts increased the frequency and success rate of such recommendations and were associated with shorter response times, suggesting that potential losses may facilitate more rapid selection of detector-disadvantageous options. At the single-brain level, DLPFC activation was higher during receiver-disadvantageous

recommendation trials in gain contexts than in loss contexts, indicating context-dependent modulation of prefrontal control processes. However, this effect should not be interpreted as deception-specific activation, because the limited number of honesty trials precluded a sufficiently powered deception-versus-honesty comparison. More critically, the inter-brain results showed that contextual modulation of DLPFC ΔINS emerged selectively during successful deception, but not during honest or failed deception trials. Trial-level analyses further showed that DLPFC ΔINS distinguished successful from failed deception in both gain and loss contexts, with earlier differentiation in gain contexts and more sustained differentiation in loss contexts. Finally, hierarchical regression and cross-validated model comparison indicated that DLPFC ΔINS explained unique variance in deception success beyond single-brain activation and direct eye contact. Together, these findings suggest that gain-loss contexts shape deception-related outcomes not only through individual prefrontal regulation, but also through the alignment of control-related processes between interaction partners.

Behaviorally, gain-loss contexts modulated deception-related choices during interpersonal interaction. Senders made more receiver-disadvantageous recommendations in loss contexts than in gain contexts, consistent with Prospect Theory's claim that losses carry greater psychological weight than equivalent gains[8] and with evidence that potential losses can increase dishonest or norm-violating behavior aimed at avoiding negative outcomes[12]. Response-time results further showed that loss contexts were associated with faster decision implementation: senders made receiver-disadvantageous recommendations faster than honest recommendations, and receivers accepted such recommendations faster than they rejected them. This pattern accords with the view that loss contexts can promote rapid, threat-sensitive processing[35], whereas gain frames often involve more deliberative value comparison[7]. Nevertheless, these effects should be interpreted within the task incentive structure. Because the payoff matrices created direct conflicts of interest and only one trial per context was selected for payment, senders may have developed relatively stable strategic tendencies across trials. Thus, the results indicate that gain-loss framing modulated the frequency, speed, and success of receiver-disadvantageous recommendations, rather than providing evidence for fully independent trial-wise decisions to deceive.

The neural findings suggest that gain-loss contexts shaped deception-related outcomes through both individual prefrontal control and dyadic control alignment. At the individual level, DLPFC activation during receiver-disadvantageous recommendation trials was stronger in gain contexts than in loss contexts. Given the limited number of honesty trials, this effect should not be interpreted as deception-specific activation. Rather, it suggests that gain contexts increased the control demands required to implement receiver-disadvantageous recommendations. This interpretation is consistent with evidence that the DLPFC is preferentially recruited when decisions require goal maintenance, response inhibition, strategic monitoring, and contextual regulation[21,24,26], as well as with accounts of prefrontal control in multi-cue tradeoffs and norm–benefit conflicts[17,22]. Thus, whereas loss contexts promoted more frequent and faster receiver-disadvantageous recommendations, gain contexts appeared to require greater individual-level control.

Inter-brain results extend this account by showing that deception success was associated with the alignment of

control-related processes between partners. DLPFC ΔINS showed contextual modulation selectively during successful deception, but not during honest or failed deception trials, arguing against a simple explanation based on general interaction intensity or verbal engagement. Instead, this pattern suggests that gain-loss contexts modulated the degree to which control-related processes became synchronized when receiver-disadvantageous recommendations successfully influenced the receiver's choice. This interpretation is consistent with hyperscanning evidence linking DLPFC synchrony to strategic coordination, negotiation, competition, and shared behavioral adjustment[6,34], and with the view that DLPFC-related INS reflects shared regulation of rules, goals, and strategies rather than emotional resonance alone[32]. Because speech recordings were unavailable, however, we cannot determine whether enhanced DLPFC ΔINS reflected specific verbal tactics, stable dyadic strategies, or moment-to-moment persuasive adjustments. Therefore, DLPFC ΔINS should be interpreted as evidence for dyadic control alignment during successful receiver-disadvantageous influence, rather than as a marker of a specific communicative strategy.

Furthermore, ΔINS not only distinguished successful from failed deception statistically but also demonstrated significant predictive power in both contexts, with higher classification accuracy in loss contexts. This suggests that INS is not a post-hoc averaged group effect but carries real-time information about decision outcomes within single interactions[44,45]. More importantly, time-series analysis revealed distinct temporal dynamics: in gain contexts, INS differences between success and failure emerged early (approximately 7 seconds), suggesting that in context requiring higher strategic monitoring, interacting partners enter a state of control alignment earlier[46]. In contrast, in loss contexts, differences appeared later (approximately 10 seconds) but persisted until the end of the trial, indicating that synchronization may not be a prerequisite for the initiation phase but rather strengthens progressively to consolidate decisions and maintain influence[47,48]. Taken together, the task-level and trial-level INS results jointly indicate that successful deception relies on the cross-brain synergy of control networks between interacting partners rather than merely individual control resource investment[49]. Gain and loss contexts shape the timing and intensity of this synergy by modulating decision rhythms and motivations, reflecting differentiated interactive regulation mechanisms at the neural level.

ML analysis advanced these associations from "explanation" to "generalizable prediction." Cross-algorithm comparisons showed that the LSR model performed robustly in both contexts, indicating that ΔINS contains reproducible, cross-model robust behavioral information rather than accidental fitting. Simultaneously, the higher explanatory power and stronger negative correlation observed in gain contexts align with the aforementioned dynamic evidence that "deception in gain frames relies more on high-cost strategic monitoring and earlier synchronization differentiation." In gain contexts, INS appears to be a core variable determining individual differences, whereas in loss contexts, where behavior is driven more by approach-avoidance motivation, ΔINS remains predictive but has more limited explanatory power. This confirms that gain-loss contexts modulate not only the pathway of deception execution but also the efficiency of interactive alignment that influences successful deception.

Several limitations should be noted. First, the 15-s open verbal interaction phase enhanced ecological validity

but introduced semantic uncertainty. Because speech recordings and verbatim coding were unavailable, deception was defined operationally by the payoff structure—that is, receiver-disadvantageous recommendations—rather than by confirmed linguistic falsehoods. Second, although multi-step preprocessing and trial-level quality screening were applied, fNIRS signals during naturalistic speech may still be influenced by head motion, facial movement, articulation, and superficial blood flow. Accordingly, prefrontal activation and INS effects should be interpreted as ecological functional patterns rather than fine-grained localization of deception-specific neural circuits. Third, the incentive structure may have encouraged relatively stable response tendencies, and the high rate of receiver-disadvantageous recommendations left too few honesty trials for reliable deception-versus-honesty neural comparisons. Therefore, this study should not be interpreted as identifying the neural basis of deception per se, but as showing how gain-loss contexts modulate prefrontal activity and INS during receiver-disadvantageous recommendations in a high-conflict interaction task. Future studies should incorporate speech recording, semantic coding, trial-wise feedback, and more balanced honest/deceptive choices to test deception-specific mechanisms more directly.

In summary, by integrating behavioral measures, single-brain activation, and inter-brain synchronization within an ecologically valid interaction paradigm, this study provides evidence for a context-sensitive interpersonal neural account of receiver-disadvantageous recommendations. The findings suggest that gain–loss framing shapes both the behavioral implementation of such recommendations and the prefrontal interpersonal synchrony associated with their success. More broadly, this work advances the use of INS as a core metric for explaining and predicting interactive social influence, supporting a shift in social neuroscience from correlational description toward mechanistic modeling and behavioral prediction.

(None)
